# A Machine Learning-Based Genome Mining Approach Reveals Unprecedented Biarylitide Diversity

**DOI:** 10.1021/jacsau.6c00500

**Published:** 2026-07-13

**Authors:** Leo Padva, Jemma Gullick, Friederike Biermann, Laura J. Coe, Yongwei Zhao, Sam Tucker, Lukas Zimmer, Julien Tailhades, Stefan Kehraus, Ralf B. Schittenhelm, James J. De Voss, Eric J.N. Helfrich, Max J. Cryle, Max Crüsemann

**Affiliations:** † Institute of Pharmaceutical Biology, 9374 University of Bonn, Bonn 53115, Germany; ‡ Department of Biochemistry and Molecular Biology, The Monash Biomedicine Discovery Institute, 2541 Monash University, Clayton, Victoria 3800, Australia; § 1974 ARC Centre of Excellence for Innovations in Peptide and Protein Science, Clayton, Victoria 3800, Australia; ∥ Institute of Molecular Biosciences, 9173 Goethe University Frankfurt, Max-von-Laue Strasse 9, Frankfurt am Main 60438, Germany; ⊥ School of Chemistry and Molecular Biosciences, The University of Queensland, Brisbane, Queensland 4067, Australia; # Institute of Pharmaceutical Biology, 9173 Goethe University Frankfurt, Frankfurt am Main 60438, Germany; ∇ The Monash Proteomics and Metabolomics Platform, Monash University, Clayton, Victoria 3800, Australia; ○ Senckenberg Society for Nature Research, Senckenberganlage 25, Frankfurt am Main 60325, Germany

**Keywords:** biarylitides, RiPPs, genome mining, biosynthesis, P450, cross-linking, machine learning

## Abstract

Biarylitides are a group of bacterial ribosomally synthesized and post-translationally modified peptides (RiPPs) that contain a biaryl bridge formed by dedicated cytochrome P450 enzymes that can introduce different cross-links. The biarylitides are produced via a five-amino-acid precursor peptide, encoded by a minimal 18 bp gene that evades automatic detection. Previous genome mining approaches for biarylitides do not capture their full biosynthetic space. We therefore repurposed a machine learning algorithm to comprehensively chart the biosynthetic space of the biarylitides, including variation of precursor motifs, P450, and additional modifying enzymes, which yielded 277 biarylitide biosynthetic gene clusters (BGCs). We experimentally investigated biaryl formation with previously uninvestigated core peptide motifs, including YWH, YVH, and YWY, and elucidated the nature of these cross-links. This study significantly expands the biarylitide precursor and BGC diversity and provides directions for the systematic exploration of other RiPP families.

## Graphical abstract

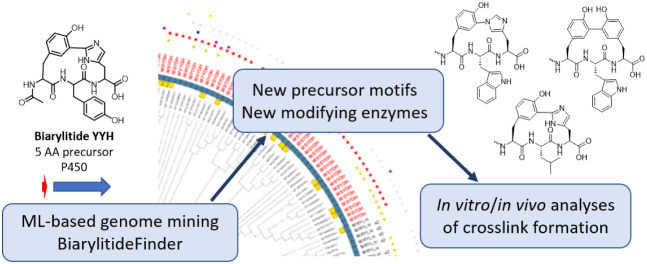

## Introduction

Natural products are a fascinating source of chemical complexity that enables these diverse classes of molecules to possess a wide range of biological functions, providing the producing organism with a competitive advantage.^1^ Peptides are an important natural product class, particularly for human health, given the number of clinical and preclinical antibiotics that are natural or second-generation peptides.^2,3^ This includes licensed drugs with broad-ranging activity, such as the penicillins, lipopeptides (such as daptomycin), and glycopeptide antibiotics (such as vancomycin). Additional examples of preclinically evaluated molecules from this group are darobactin, which has activity toward Gram-negative bacteria,^4^ and rufomycin, which acts against *Mycobacterium tuberculosis*.^5^ Within peptide natural products, both nonribosomal as well as ribosomal biosynthetic pathways contribute to the pool of available antibiotics, with ribosomally synthesized and posttranslationally modified peptide (RiPP) pathways growing in prevalence.^6^ Many of these peptide antibiotics display some form of side-chain cross-linking within their structures, and many of these cross-links are installed by the actions of cytochrome P450 (P450) enzymes, powerful oxidative hemoproteins capable of catalyzing a wide range of transformations.^7^

The importance of cross-linked peptides as antibiotics has made the identification of RiPP pathways containing these enzymes a priority for the field. In this regard, key discoveries that have supported the rapid diversification of known P450-cross-linked RiPP pathways were the identification of the biosynthetic pathways of cittilin^8^ and tryptorubin A,^9^ and the discovery of the biarylitides (Figure 1).^10^ The elucidation of atropopeptide biosynthesis (exemplified by tryptorubin A)^9,11^ —highly complex, multicyclic cross-linked RiPPs, in which P450 enzymes install the cross-links between aromatic side chains and between aromatic side chains and the peptide backbone, respectively—provided a key advance in supplying the template that enabled a series of computational works to identify P450 cross-linking in RiPP pathways.^12–14^ Biarylitides are cross-linked tripeptides produced from a pentapeptide precursor by P450-mediated aromatic cross-linking. They form a separate group within P450-mediated cross-linked RiPPs, due to their minimal precursor sizes (the smallest known RiPP precursors and, with 18 bp, the smallest known genes across the tree of life), in which the leader peptide sequence consists of only two amino acids (Figure 1).^10,15^ This is distinct from other P450-cross-linked RiPP pathways, where the final product of the pathway is significantly shorter than the complete leader-core­(follower) precursor peptide.^8,9,12–14^

**Figure 1:**
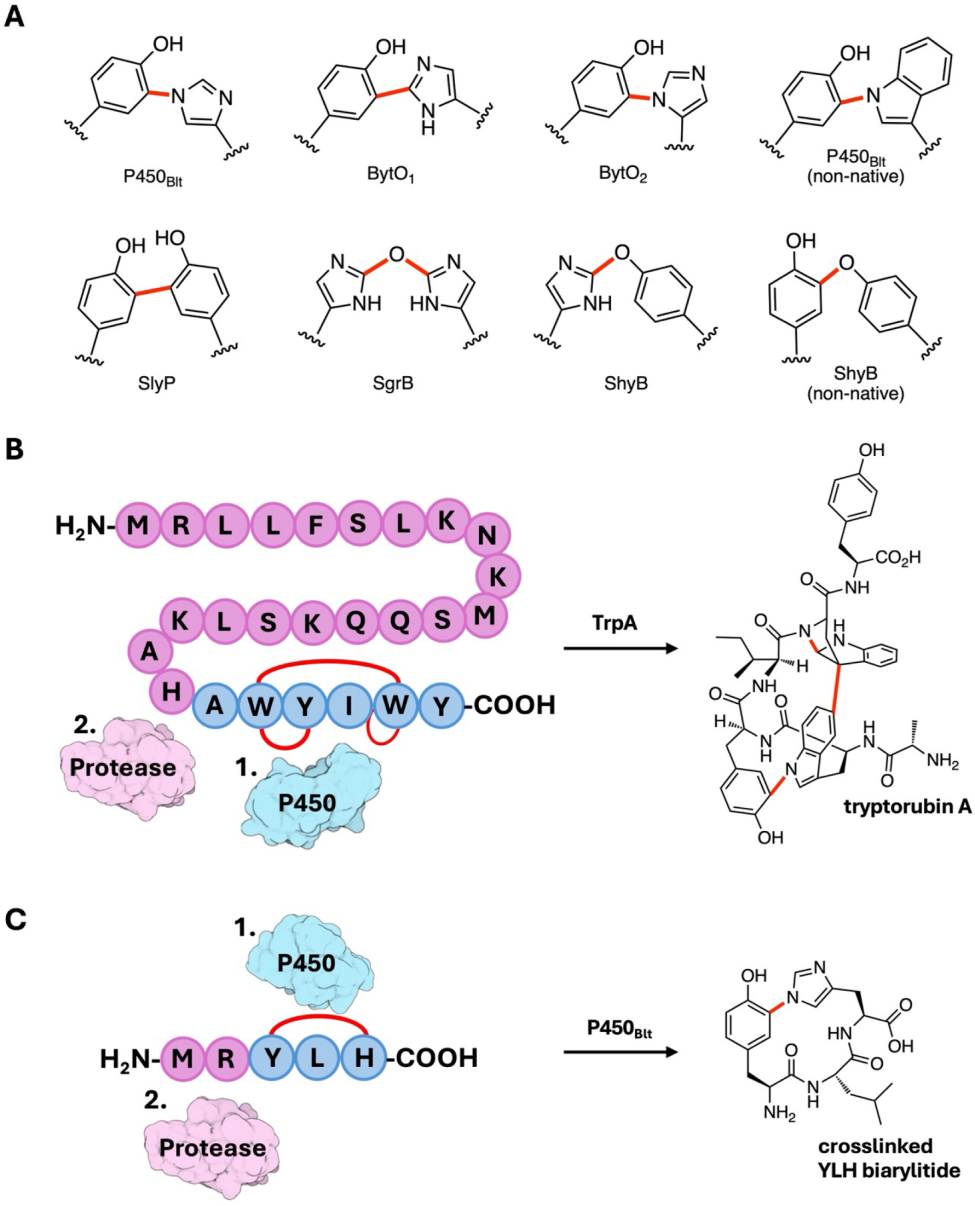
Structures and biosynthetic models of selected side-chain cross-linked peptide natural products. **A)** Structural diversity of biarylitides with cross-links shown in red, including the native examples of P450_Blt_ (*Micromonospora* sp. MW-13), BytO_1_, BytO_2_, SlyP, SgrB, and ShyB, as well as non-native examples from in vitro studies with P450_Blt_ and ShyB. **B)** RiPP biosynthetic pathway for tryptorubin A, numbers indicate order of enzyme activity. **C)** RiPP biosynthetic pathway for the biarylitides, numbers indicate order of enzyme activity (example shown for P450_Blt_).

The biarylitides have also shown promise in their potential use in the biocatalytic production of cross-linked tripeptides,^16–22^ which themselves are important precursors to antibiotics such as arylomycin.^23^ While the majority of the P450-cross-linked RiPPs identified to date have little or no activity in typical antimicrobial screens,^10,24^ peptides related to the biarylitides have been shown to have potential effects on plants,^25^ but other biological activities remain elusive. Furthermore, the P450s that cross-link the biarylitides have been identified to form links between different combinations of His, Tyr, and Trp residues. Importantly, from a building block perspective, the types of cross-links installed within the same precursor peptide can differ depending on the P450 (or engineered mutant) utilized.^16–22^ Biarylitide pathways have also been identified as the source of the 3-nitrotyrosine building block that is incorporated into the nonribosomal peptide antibiotic rufomycin.^26^ Here, subtle changes in the active site to one specific clade of C–N YxH cross-linking biarylitide P450 enzymes have completely altered their function to specifically catalyze nitration of tyrosine within their pentapeptide substrate, providing nitrotyrosine, which is released from the pentapeptide, as a precursor for nonribosomal peptide biosynthesis.^26^ Given the diverse range of activity and substrates identified, it is important to identify further examples of biarylitide pathways to explore the scope of the substrates and catalyzed cross-links. Therefore, in this work, we used a machine learning-based approach to explore the prevalence of biarylitide pathways that lie outside of those in which the precursor peptide and P450 are tightly clustered, expanding substrate diversity and catalytic potential within this fascinating peptide natural product family.

## Results and Discussion

### Bioinformatic Discovery of a Novel Subgroup within the Biarylitide Family

Biarylitide precursor diversity, first described by Zdouc et al.,^10^ has been established based on two key characteristics: a conserved five-amino acid precursor peptide containing specific aromatic residues (MxYxY/H) and tight genomic coupling of the precursor gene (*bytA*) and the gene encoding its cognate cross-linking enzyme (*bytO*). This idea remained consistent, as subsequent studies identified BytA variants with alternative cross-linked aromatic residue patterns, while maintaining the characteristic proximity between *bytA* and *bytO* homologues.^12–14,20,24^

However, during manual analyses of biarylitide BGCs, we identified a *bytO* homologue in*Stackebrandtia nassauensis* that lacked its expected precursor peptide partner within the conventional 500-base-pair window, which was empirically determined to be sufficient to find the precursor gene in previous studies. A detailed reinvestigation of the surrounding genomic region revealed an unexpected finding: a *bytA* candidate encoding an MRYWY motif was located approximately 1400 bp downstream of *bytO* (Figure S1). This discovery represented a significant deviation from the established spatial arrangement of biarylitide BGCs while also introducing a novel precursor sequence motif. The identification of this unusual cluster architecture, combined with mounting evidence of greater biarylitide diversity than previously anticipated,^12–14,20,24^ prompted us to develop a machine-learning-based genome mining strategy to capture the full biarylitide biosynthetic diversity.

### Machine-Learning-Based Genome Mining of Biarylitides

Traditional sequence-based approaches for identifying biarylitide biosynthetic genes have their inherent limitations. While BLAST searches^27^ excel at identifying closely related homologues, they prove less effective for charting the full diversity of biarylitide-modifying P450s due to their characteristically low sequence conservation. PSI-BLAST,^28^ despite offering greater flexibility through its iterative search approach, often yields a large proportion of false positives resulting from the identification of P450s unrelated to biarylitide biosynthesis. Furthermore, the detection of *bytA* homologues is hindered by their exceptionally small size (18 bp), which prevents reliable annotation and, consequently, excludes them from current gene detection and annotation pipelines.

We thus adapted the AtropoFinder genome mining pipeline,^29^ originally developed for the identification of atropopeptide BGCs, for the detection of biarylitide BGCs. Briefly, AtropoFinder uses a random Forest classifier todifferentiate atropopeptide-modifying P450s from P450s that modify any other substrate. For the training of the classifier, the P450s were divided into functional regions, the amino acid code simplified based on their physicochemical properties and a k-mer-based featurization strategy employed.^30^ In a second step, AtropoFinder uses a hard-coded, rule-based approach to localize the corresponding precursor gene in the vicinity of the gene encoding the atropopeptide-modifying P450. When applied to the RefSeq database, AtropoFinder identified more than 680 putative atropopeptide BGCs—thus more than doubling the number of atropopeptide BGCs identified when compared to traditional sequence similarity searches.^29^

To chart the biosynthetic diversity of the biarylitides, we adapted the AtropoFinder algorithm. We trained the BiarylitideFinder on carefully curated positive and negative data sets. The positive training set initially combined six experimentally validated biarylitide P450 sequences^10,12–14,19,24^ with 181 bioinformatically identified putative biarylitide P450s from previous studies (55 from Zdouc et al.^10^ and 126 from Nam et al.^12^). These 181 putative biarylitide-modifying P450s in the positive training data set were identified by BLAST-based searches, and the corresponding putative biarylitide precursor genes were identified in close proximity to the P450 gene. The negative data set was assembled from diverse sources: the MIBiG database,^31^ verified BGCs not yet incorporated into the MIBiG database, predicted atropopeptide P450s,^9^ other putative RiPP-modifying P450s,^12^ as well as P450s from the antiSMASH database.^32^ Following dereplication, the final training sets used for the classifier contained 106 positive and 9,761 negative P450 sequences. For precursor peptide detection, CoreFinder was adapted for the detection of the unusually short biarylitide precursor peptides, specifically to search for precursor sequences between 5 and 8 amino acids in length that contain aromatic amino acids at positions 3 and 5.

Application of this integrated approach to the NCBI GenPept database^33^ identified 1,128 putative biarylitide-modifying P450s. After the removal of duplicates and manual curation, 277 unique BGCs were identified, each containing genes encoding both a P450 and one or more corresponding precursor peptides. The genomic context of these clusters was further analyzed using RODEO2 to detect coencoded tailoring enzymes through Pfam domain identification.^34^

This workflow substantially expanded the known diversity of biarylitide biosynthesis by identifying 124 novel P450s and bringing the total number to 277 putative members (Figure 2). A complete list with all identified P450 IDs, species names, and coclustered genes can be found in Table S1. This analysis provided an overview of the phylogenetic distribution of biarylitide BGCs across bacterial phyla, with predominant representation in Actinomycetota and additional presence in Bacillota and Pseudomonadota. Notably, the analysis revealed potential biarylitide biosynthetic capability in the human microbiome-associated bacteria, specifically identifying *bytO* homologues and MQYAH precursor peptides in*Rothia dentocariosa* and*Rothia* *sp*. HMSC058E10 both of these Gram-positive bacteria are naturally present in the oral and respiratory microbiota,^35^ representing the first identification of potential biarylitide production in human-associated microorganisms.

**Figure 2:**
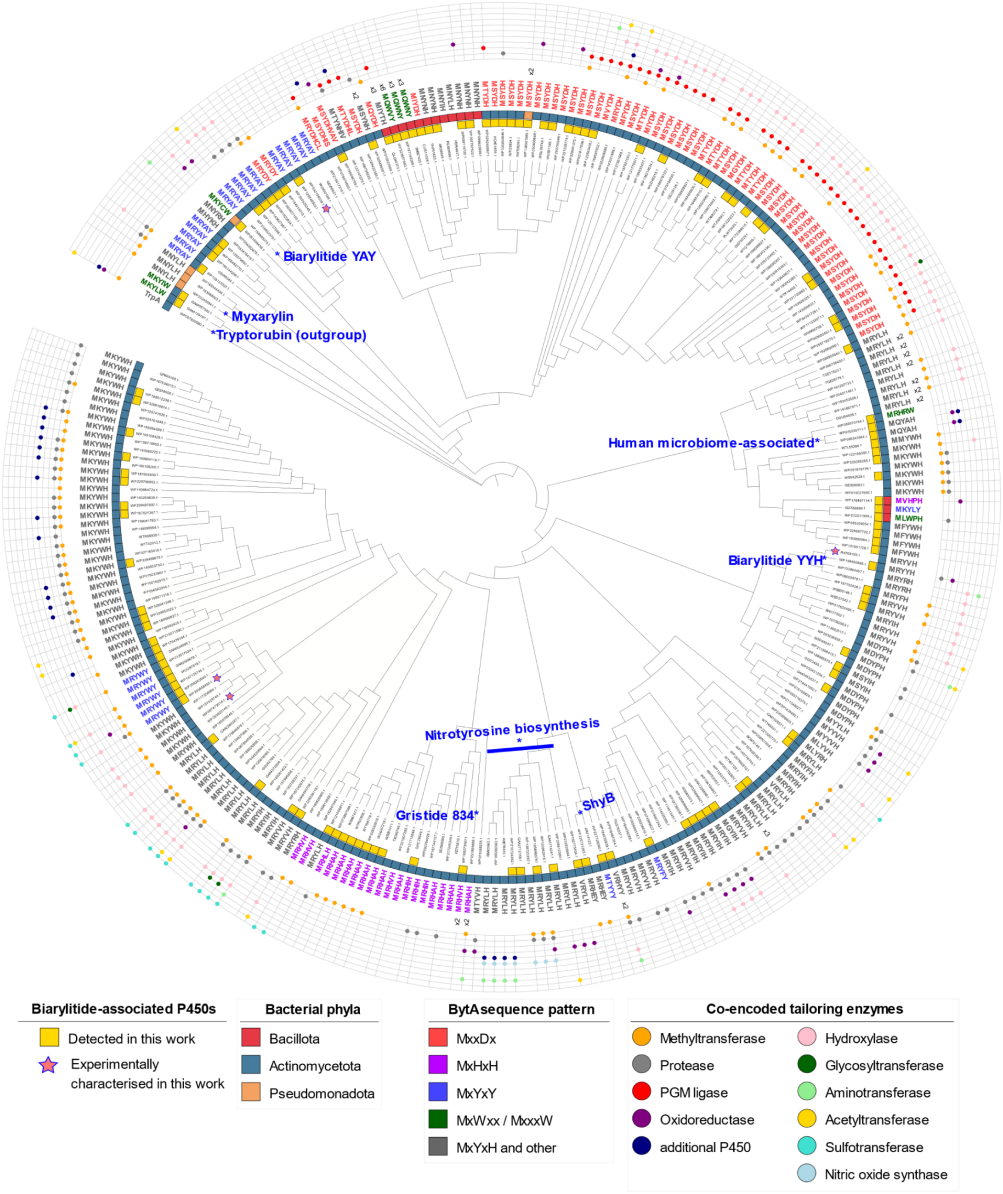
A phylogenetic tree showing the diversity and distribution of putative biarylitide-modifying P450s and their BGCs, identified through BiarylitideFinder. The integrated visualization highlights the taxonomic distribution across bacterial phyla, characteristic BytA sequence patterns, already published examples, systems targeted in this work, and the variety of coencoded tailoring enzymes, revealing distinct clades of potential biarylitide biosynthetic systems.

The phylogenetic analysis revealed several distinct precursor sequences and motif groups. The previously identified *S. nassauensis* *byt* BGC, notable for both its MRYWY motif and its unusual 1,400 base pair separation between *bytA* and *bytO* homologues, emerged as the founding member of a conserved clade comprising six BGCs sharing identical BytA motifs and similar tailoring enzyme compositions (Figure S2). All six BGCs in this clade show physical separation of *bytA* and *bytO*, which, however, turned out to be a unique phenomenon in the 277 BGC data set. Five BGCs in this clade, in addition to 12 other BGCs in our data set, encode sulfotransferases, a relatively understudied enzyme class in RiPP biosynthesis with few characterized members.^36^ Furthermore, our analysis revealed nine novel precursor peptide motifs containing tryptophan at cross-linking positions, including MKYLW and MKYIW variants in *Actinomadura* strains. These findings are significant given that tryptophan at position 5 has been experimentally validated to lie within the catalytic scope of P450_Blt_ from *Micromonospora* MW-13 and of myxarylin P450_BytO_ by in vitro studies.^19,21^ A particularly noteworthy discovery was made in four *Lysinibacillus* strains (phylum Bacillota), which harbor the MQWNY motif with tryptophan at position 3. These strains exhibited remarkable *bytA* homologue copy number variation, with one strain containing six copies. Multiple precursor peptides have been reported for various RiPP classes, such as cyanobactins, with reports of up to 10 precursor peptides leading to an increased production of the compounds.^37^

Our analysis also revealed distinct patterns in the co-occurrence of tailoring enzymes and specific precursor sequences. A striking example is the frequent association between Pgm1 ligase homologues and precursor motifs containing aspartic acid at position 4, observed in 47 BGCs. The Pgm1 ligase is an ATP-dependent amide bond-forming enzyme of the ATP-grasp superfamily, first discovered in pheganomycin biosynthesis.^38^ Pgm1 catalyzes the conjugation of ribosomal peptides with nonproteinogenic amino acids, suggesting another case of biarylitide pathway integration with distinct biosynthetic logic. This integration of different biosynthetic pathways is further exemplified by BGCs involved in 3-nitrotyrosine biosynthesis, which contain coencoded nitric oxide synthases and form a distinct phylogenetic clade. This clade represents the recently characterized rufomycin-associated biarylitide pathways, in which subtle changes in the active sites of the P450_Blt_-related RufO enzymes enabled them to gain the ability to nitrate the central tyrosine of the MRYLH peptide for the production of nitrotyrosine, a noncanonical precursor for nonribosomal peptide antibiotic biosynthesis.^26,39^

### Experimental Validation of P450 Enzyme Cross-Links

#### MKYWH Motif

To validate our bioinformatic findings, we experimentally investigated selected candidate biarylitide BGCs. The first major focus was the characterization of BytO homologues associated with the MKYWH motif (**1**), one of the most widely distributed sequence patterns across bacterial genomes, that had not previously been experimentally explored. The prevalence of this motif, combined with the observation in a previous study that P450_Blt_ did not tolerate large residues such as tryptophan at position 4 of the substrate peptide,^19^ made structural characterization and the exploration of tolerance for peptide modifications particularly intriguing.

With initial *in vivo* studies aiming to explore the activity of the BytO homologue from *Actinomadura hibisca* (AchB) unsuccessful, we turned to in vitro experiments to assess the promiscuity of this enzyme (AchB) for different amino acids in position 4 of peptide **1**(Figures S7–S22, Figures S51–S66). While the activity of AchB toward the “native” Nle-KYWH substrate [**Nle-1**; norleucine (Nle) preferred over Met to avoid off-pathway sulfoxidation of Met] was relatively high (70%), as shown by the formation of a −2 Da cross-linked product (**Nle-2**; *m*/*z*: 744.4) relative to the starting peptide (*m*/*z*: 746.4) (Figure 3, Figures S83, S84), a screen of amino acids in position 4 showed a significant preference for W across all potential substitutions tested. Aliphatic and aromatic residues with large, nonpolar side chains (leucine, valine, and tyrosine) were accepted to some extent by AchB (<50% relative conversion to **Nle-1**), but no tolerance was demonstrated toward smaller aliphatic (alanine) or charged (glutamic acid) residues, making AchB one of the more specific biarylitide P450 examples studied to date (Figure 3, Figures S85–S89).^18–21,40^ We further explored the promiscuity of AchB by altering the position 5 amino acid to Tyr and Trp (Figures S90, S91). These had <2% conversion, highlighting the specificity of this enzyme for Tyr-His cross-linking.

**Figure 3:**
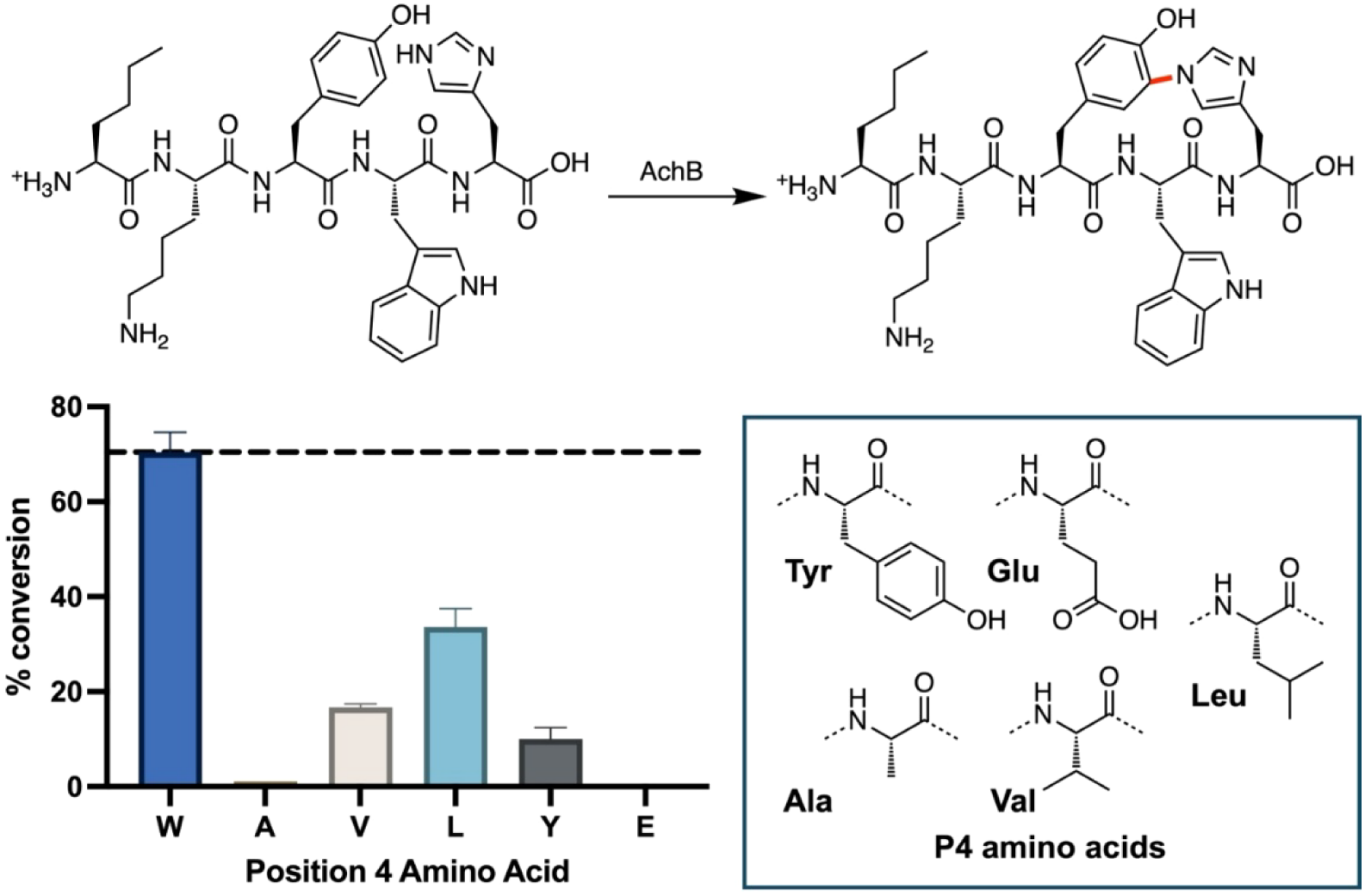
In vitro enzymatic assay data of the substrate promiscuity of AchB indicate the cross-linking outcome along with position 4 variants of Nle-KYWH (**Nle-1**) tested. Data is represented as the mean % conversion of linear peptide to cross-linked peptide, with error bars showing SD, *n* = 3.

To enable structural characterization of the cross-link type installed by AchB, an in vitro reaction was scaled up 100-fold using the native Nle-KYWH substrate (**Nle-1**). Reaction products were purified by reverse-phase HPLC. Once isolated, the structure of the cross-linked pentapeptide Nle-KYWH (**Nle-2**) was determined by one- and two-dimensional NMR spectroscopy (Figures S92–S99). Analysis was performed using DMF-*d*_7_, consistent with our prior reports^15^ and resulted in a well-resolved ^1^H spectrum. Five α-protons were readily identified at δ_H_ 4.18, 4.48, 4.77, 5.12, and 4.73, consistent with the five expected residues. Corresponding α-carbon resonances were identified by ^13^C-HSQC at δ_C_ 54.3, 54.2, 54.8, 54.9, and 53.2, respectively. Five carbonyl resonances were observed in the ^13^C spectrum at δ_C_ 170.3, 171.6, 170.9, 173.1, and 173.9, once again consistent with a pentapeptide (Figure S98). A TOCSY spectrum, in addition to COSY, ^13^C-HSQC, ^13^C-HMBC and ^15^N-HSQC spectra, facilitated assignment of the pentapeptide backbone and the Nle, Lys, and Trp residues (Figure 4, Figure S95).

**Figure 4:**
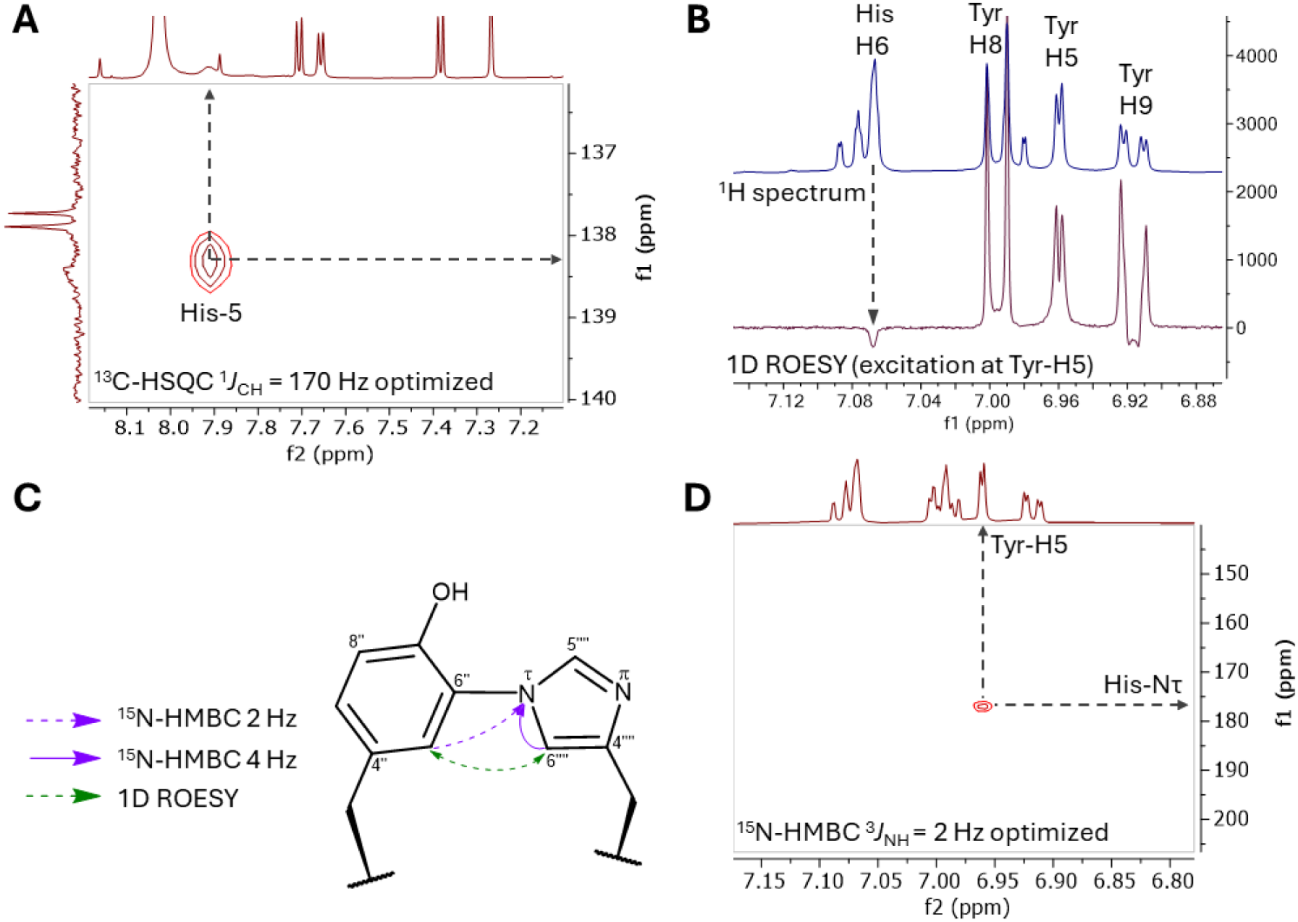
NMR characterization of the Tyr-His cross-link in cross-linked Nle-KYWH (**Nle-2**). (A) Extract of ^13^C-HSQC spectrum optimized for^1^ *J*_CH_ = 170 Hz. (B) Extract of 1D ROESY spectrum with irradiation of Tyr-H5 at δ_H_ 6.97. The NOE relationship to His-H6 results in a negatively phased signal, while signals resulting from scalar relationships appear antiphase/positively phased. (C) Key through cross-link ^15^N-HMBC and ROESY correlations in the Tyr-His biaryl fragment. (D) Extract of ^15^N-HMBC spectrum optimized for observation of *J*_NH_ 2 Hz coupling, highlighting the correlation between Tyr-H5 and His-N­(τ).

We then turned our attention to the critical Tyr and His side-chain resonances. ^1^H resonances at 6.99 (d, *J* = 8.2 Hz, Tyr-H8) and δ_H_ 6.93 (dd, *J* = 8.2, 2.2 Hz, Tyr-H9) demonstrated a typical *J* = 8.2 Hz *ortho* coupling. A third signal, δ_H_ 6.97 (d, *J* = 2.2 Hz, Tyr-H5), demonstrated a typical *J* = 2.2 Hz *meta* coupling. The corresponding carbon resonances were located by ^13^C-HSQC correlations at δ_C_ 117.3, 130.6, and 129.7. A peak was identified in the downfield region of the proton spectrum at δ_H_ 10.48, consistent with a Tyr phenolic proton. These signals suggested the presence of an AMX system, consistent with a C6-substituted Tyr residue in **Nle-2** (Figure S96).

One final aromatic resonance remained unassigned in the ^13^C-HSQC spectrum, identified at δ_H_ 7.09 (s, br, ov), δ_C_ 120.2, the latter of which was correlated by HMBC with the His-H3 β-protons (δ_H_ 3.22 dd, 14.7, 4.9 Hz), 3.13 (*J* = 14.7, 8.7 Hz). The former was therefore assigned as His-H/C6. The resonance corresponding to His-C4 was identified at δ_C_ 137.9 by HMBC correlation from His-H6. Prior experience examining spectra containing His residues suggested that any His-H5 to His-C5 correlation was unlikely to be observed in a standard ^13^C-HSQC experiment optimized for^1^ *J*_CH_ = 145 Hz.^15,17^ A second ^13^C-HSQC experiment was therefore undertaken, increasing the^1^ *J*_CH_ parameter value from 145 to 170 Hz. The modified experiment was run band-selectively, analyzing only the 105 – 165 ppm region in the F1 dimension, thereby improving resolution in this region of interest. This experiment revealed a ^13^C-HSQC correlation between a very broad resonance at δ_H_ 7.91 (s, br) and δ_C_ 138.2, which was assigned as His-H/C5 (Figure 4A). HMBC correlations were observed to His-C5 (δ_C_ 138.2) from His-H6 and His-H3 (Figure 4, Figure S97), consistent with these assignments. Identification of His-H5 and His-H6 suggested that the His residue was therefore cross-linked to the Tyr residue through either the N -tele (τ) or -pros (π) position. Differentiation between these two isomers in His-N cross-linked peptides can be challenging by NMR.^15^ No NOE correlation was observed between His-H6 and Tyr-H5 using a 1D NOESY experiment. However, a 1D ROESY experiment facilitated observation of a through-space correlation between these protons that would be consistent with a Tyr-C6 to His-N (τ) cross-link (Figures 4B, S93). We then focused on the observation of correlations through the proposed cross-link by ^15^N-HMBC. In order to optimize experimental sensitivity, we sought to determine the expected *J*_HN_ coupling constant between Tyr-H5 and His-N­(τ) by analysis of 1-phenylimidazole as a simplified analogue of the region of interest. Using this data as a guide, a^3^ *J*_NH_ coupling was observed between Tyr-H5 and His-N­(τ) using a ^15^N-HMBC experiment optimized for *J*_NH_ = 2 Hz (Figure 4C, D). When optimized for *J*_NH_ = 4 Hz, a correlation was observed between His-H6 and His-Nτ (Figure 4C, D). Key NMR correlations used to determine the structure of cross-linked **Nle-2** are illustrated in Figure 4 and Figure S100. NMR assignments are per Table S2.

#### MRYWY Motif

We next focused on characterizing the initially discovered BGC from*Stackebrandtia nassauensis* and a homologous example from *A. rupis*. These BGCs belong to a previously overlooked subgroup of biarylitide BGCs that encode an unusual MRYWY peptide precursor (**3**), which is intriguing both for the presence of the large Trp residue at position 4 of the substrate peptide and for the presence of tyrosine at position 5 in place of the more common histidine residue, suggesting different cross-linking chemistry. Since cultivation of *S. nassauensis* and *A. rupis*, as well as heterologous expression of the *S. nassauensis* and *A. rupis* BGCs in *S. coelicolor*, failed to afford detectable biarylitide-like products, we turned to in vitro characterization of the *A. rupis* P450 AciB. Using a synthetic Nle-RYWY substrate (**Nle-3**, Figures S23, S24, S67, S68), LC-MS analysis revealed 36% formation of a product with a −2 Da mass shift (**Nle-4**; *m*/*z* = 798.4) relative to the pentapeptide substrate (*m*/*z* = 800.4) (Figure S101). While three possible cross-links exist for such a YxY system, only a single product was observed. This specificity stands in contrast to the mixed products seen when employing such a YxY peptide as a substrate for P450_Blt_ that typically cross-links the YxH motif.^16,17^ Given this specificity, we undertook in vitro experiments using deuterated tyrosine-containing Nle-RYWY (**Nle-3**) peptides (Figures S31–S36), to determine the exact nature of the cross-linking performed by AciB (Figure 5, Figures S102–S104). Determining the loss of deuterium atoms from these three deuterated **Nle-3** probes revealed that the cross-link formed by AciB is a Tyr_3_C-Tyr_5_C C–C bond.^16^

**Figure 5:**
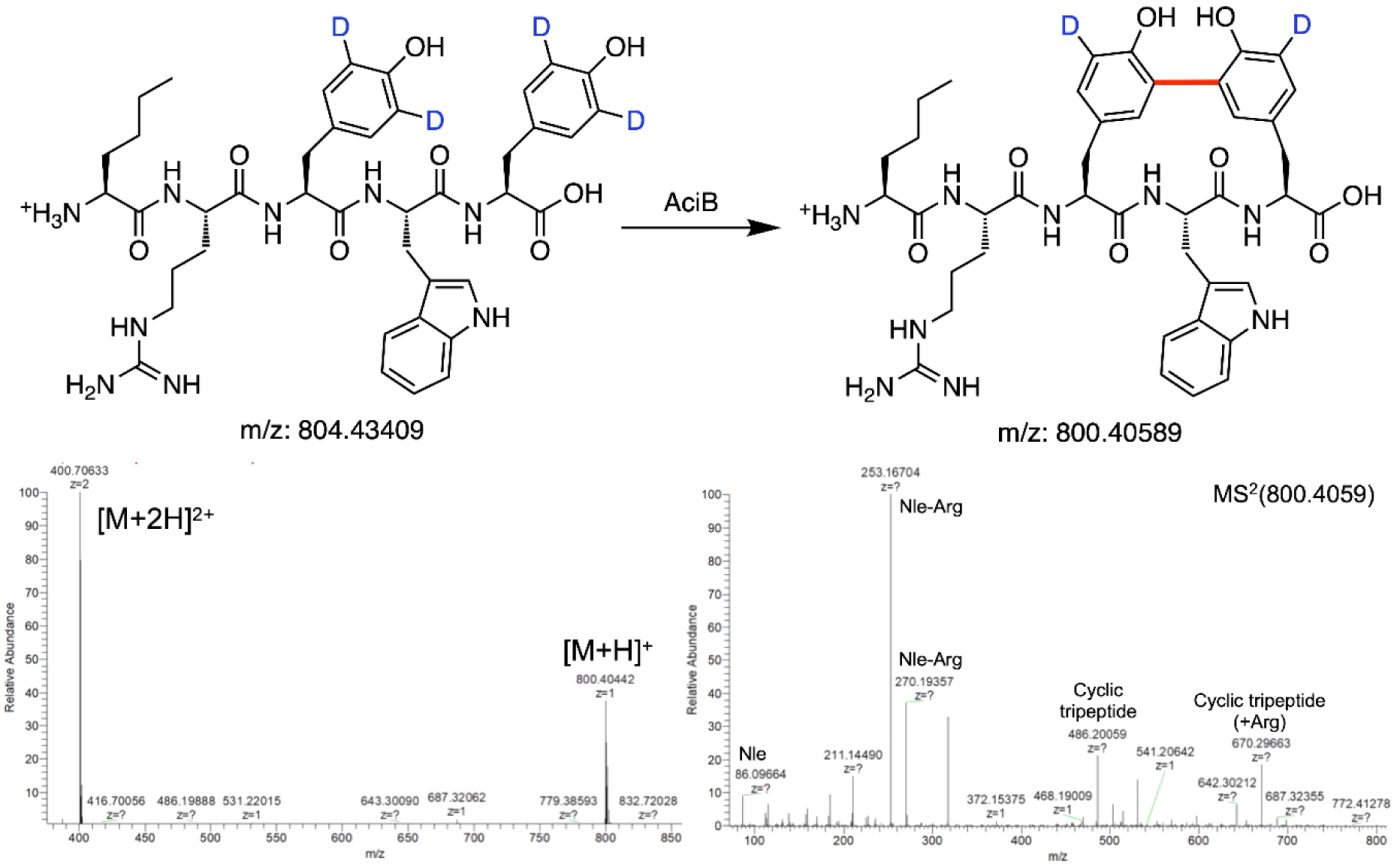
HRMS and MS^2^ spectra of the cross-linked peptide from the in vitro enzymatic assay data for the reaction of AciB from *Actinocatenispora rupis* with a double deuterated Tyr variant of **Nle-3**; Nle-R-Y­(3,5-d_2_)–W-Y­(3,5-d_2_).

To assess the tolerance of AciB for the P4 position, we next employed a panel of peptides as potential substrates (Figures S25–S30, S69–S74), which revealed that AciB is tolerant for a wide range of alternate amino acid residues in P4, and showed even higher turnovers for all three tested examples than for the native substrate with Trp in P4 (Figure 6, Figures S105–S107). These results might be at least partly attributed to the higher hydrophobicity and impaired solubility of the Trp-containing peptide vs the others tested, causing impaired turnovers in in vitro assays, as similar trends were observed in previous studies on glycopeptide antibiotics and diketopiperazines.^41–43^ The high tolerance for P4 alterations stands in contrast to the results obtained with AchB in the previous section, further suggesting that differences in P4 acceptance by biarylitide cross-linking P450s are influenced by the type of peptide cross-link that is installed (i.e., YxY vs YxH), likely due to constraints around side-chain positioning in the active site.

**Figure 6:**
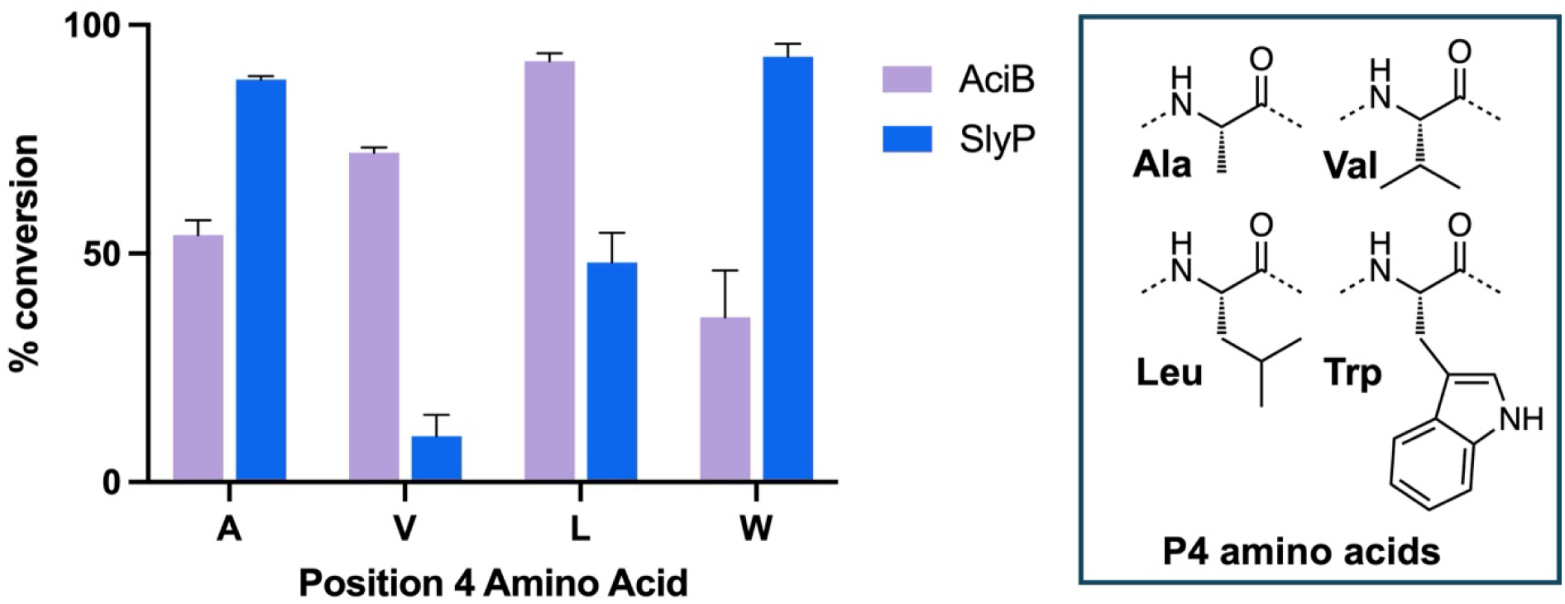
In vitro enzymatic assay results of a position 4 screen of **Nle-3** with AciB and SlyP. Data is represented as mean % conversion of linear peptide to cross-linked peptide with error bars showing SD, *n* = 3.

We next explored the in vitro activity of another biarylitide P450, SlyP, from *Streptomyces lydicamycinicus* NBRC 110027,^13^ as its native substrate, the MRYAY precursor, contains a much smaller alanine residue in the central P4 position. As with AciB, SlyP installs a carbon–carbon cross-link between the positions 3 and 5 tyrosine residues of its **Nle-3-(Ala4)** substrate (Figure S110). Interestingly, SlyP accepted the larger amino acid residues tryptophan and leucine in P4 well, showing overall a similar substrate tolerance to that of AciB for the P4 position (Figure 6, Figures S111–S115).

To extend our substrate tolerance studies for these two examples, we further investigated the enzymatic actions of AciB and SlyP on alternative substrates with His and Trp at position 5. Both AciB and SlyP did not install a Tyr-His cross-link (Figures S108 and S116), but surprisingly, both showed tolerance for Trp in position 5. SlyP installed a single cross-link with 7% conversion, while, impressively, AciB had 100% conversion to two cross-linked products (Figures S109 and S117).

#### MRYVH Motif

Finally, we focused on a *byt* BGC from *Saccharothrix variisporea* encoding a MRYVH precursor peptide and two tailoring enzymes. Beyond the canonical *bytA (savA)* and *bytO* (*savB*) genes, the gene cluster contains *savT*, encoding an amidinotransferase, and *savH*, predicted to encode an aspartyl/asparaginyl β-hydroxylase (Figure 7A). We cloned *savAB* with *savT* and *savH* in different combinations, expressed them heterologously in *Streptomyces coelicolor*, and also analyzed extracts of the native producer in different culture conditions. Somewhat unexpectedly, only an ion corresponding to the cross-linked biarylitide YVH without further modifications was detected in extracts from the wild-type or heterologous expression strains. We performed a large-scale cultivation (15 L) of *Streptomyces coelicolor* pSET_ermE*-*savAOHT* and, after HPLC-based purification, isolated 8.7 mg of cross-linked biarylitide YVH product (**5**) (Figure 7 B, C) (*m*/*z*: 458). We then analyzed the structure of **5** through a series of 1D and 2D NMR experiments (Figures S118–S123, Table S3). Structure elucidation was facilitated through direct comparison and correlation with the previously reported data for biarylitide YYH^10,15^ with predictable variations observed only at positions corresponding to the valine substitution relative to the original tyrosine. Critical to the structural elucidation of **5** was the determination of the Tyr-His cross-link configuration. Based on the 68.8% sequence homology between BytO from *Planomonospora sp*. and SavB, including crucial I-helix residues,^16,17^ a C–C cross-link was anticipated to be formed by SavB. This is supported by the chemical shift of tyrosine C6 at δ_C_ 110.6 ppm, which is in the diagnostic range (δ_C_ 108–115 ppm) characteristic of C–C cross-links in these systems,^15^ matching the signal observed in the original biarylitide cross-linked YYH product and upfield from the chemical shift range associated with C–N cross-links (δ_C_ 125–130 ppm) (Table S3).^19,21,24^ We experimentally confirmed this assignment using in vitro cross-linking of an alternative Nle-RYLH substrate (**Nle-6**, Figures S45, S46, S79, S80) and making use of various deuterated Tyr/His versions (Figures S47–S50, S81,S82) that could confirm the nature of the C–C cross-link in the product **Nle-7** from this system (Figures S124–S127).^16^ As with the previously investigated examples, we explored the tolerance of SavB for Tyr and Trp in position 5. Both alternative peptides were cross-linked in moderate amounts, with two Tyr-Tyr cross-links, the C–C and C–O being formed, and one Tyr-Trp cross-link (Figures S128–S131).

**Figure 7:**
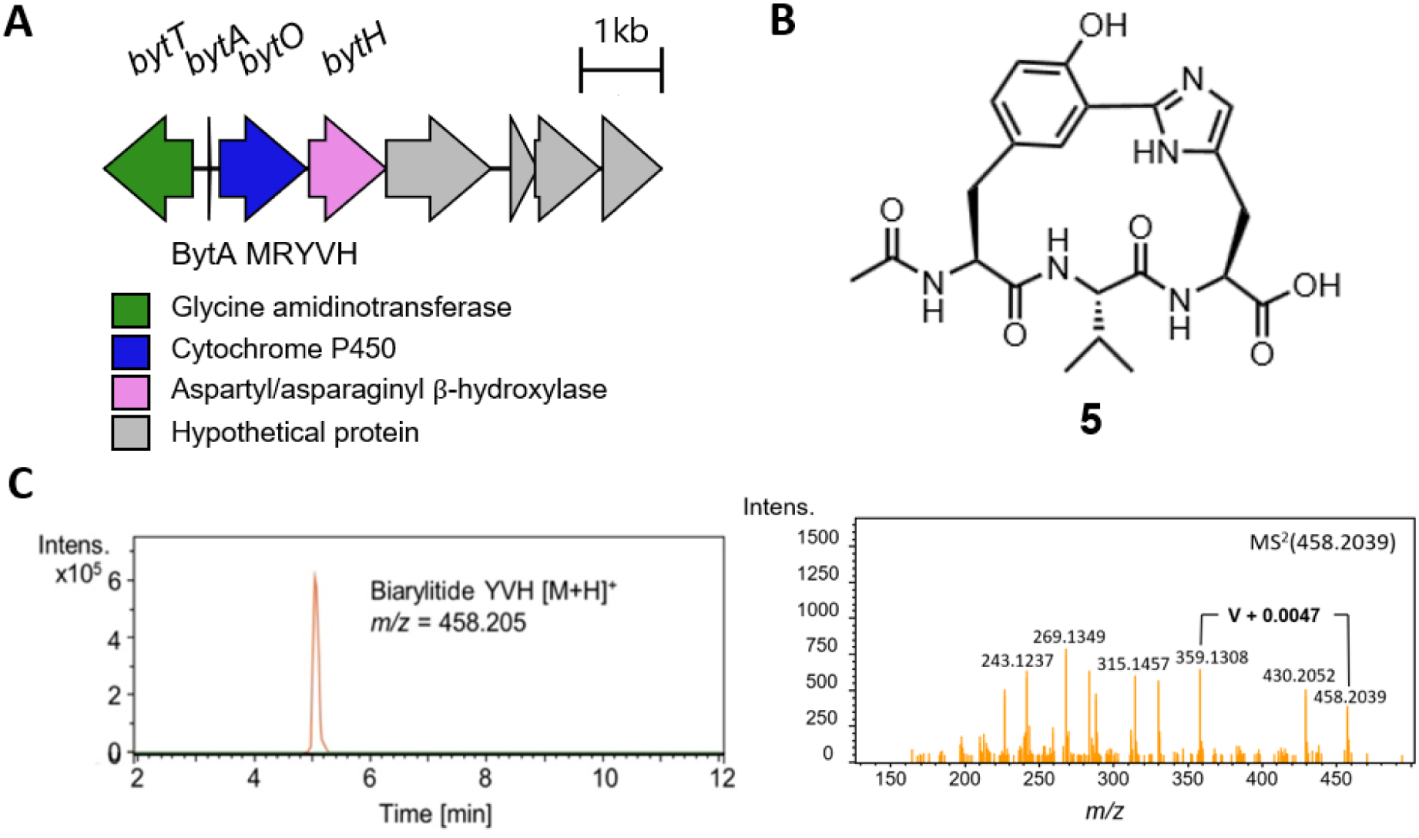
(A) Biosynthetic gene cluster from *Saccharothrix variisporea* for biarylitide YVH (**5)**. The pentapeptide precursor *bytA* is shown, due to its small size, as a dash. (B) Structure of **5**. (C) MS and MS^2^ spectra from in vivo production of **5**. In the MS^2^ spectrum, the loss of the central valine fragment (*m*/*z*: 99.026) is indicated.

We finally evaluated the bioactivity of **5** in antibacterial, antifungal, and cytotoxicity assays but observed no detectable activity in these experiments. This is in accordance with previous studies, where no bioactivity was detected for biarylitides in various assays,^10,24^ leaving the biological function of biarylitides unknown. While heterologous expression of the *sav* BGC yielded only unmodified biarylitide **5**, the role of the tailoring enzymes in the BGC remains unclear. We hypothesize that the accumulated compound is not the final metabolite of this pathway but rather a biosynthetic intermediate that may have accumulated due to impaired expression or folding of the tailoring enzymes, or the lack of appropriate enzyme substrates in the heterologous system. While the role of these additional biosynthetic enzymes remains enigmatic, the identification of the cross-linking type in **5** will aid in future investigations of the roles of these enzymes in biarylitide biosynthesis.

## Conclusions

The systematic study of biarylitide biosynthesis using a combined computational and experimental approach, provided significant insights into both precursor sequence variation and overlooked motifs and enzyme functions. An adapted machine-learning pipeline based on AtropoFinder^29^ enabled the identification of 277 unique biarylitide BGCs, including 124 novel members, not detected in previous genome mining studies.^10,12–14,20^ This analysis extended the known distribution of biarylitide producers to include human microbiome-associated bacteria, specifically identifying potential production capabilities in *Rothia* species.^35^ The computational analysis identified several novel precursor peptide motifs, particularly variants containing tryptophan at the cross-linking positions. Modeling of their associated P450s with their pentapeptide substrates (Figure S132) showed that all systems binding to MxYxW peptides adopted similar poses. In these, the Tyr-3 residue phenol presents the closest abstractable hydrogen to the heme iron, while the Trp-5 residue adopts similar poses suggestive of either C–N cross-linking with the indole nitrogen or linking via an adjacent carbon in the indole ring (Figure S132A,C,D). The model of the MxWxY complex also places the Trp-3 indole nitrogen in a similar orientation to that of Tyr-3 residues in typical MxYxH peptides, again suggesting that initial H-abstraction proceeds from residue 3 and likely affords a C–N linkage in such cases (Figure S132E). The models of peptides with a His-3 residue show that the reduced size of the His-3 side chain prevents the close approach of the imidazole ring to the heme due to the binding of the peptide backbone, and in doing so also prevents unwanted inhibition via coordination of the heme iron by the imidazole side chain (Figure S132B,F). These biarylitide systems, should they prove tractable in vitro, will no doubt prove to be important targets for future characterization to understand the regiospecificity of ring formation in these different tripeptide cross-linking pathways.

Another interesting finding was the discovery of multiple precursor copy variants in *Lysinibacillus* strains, some containing up to six copies of the precursor gene. The analysis also revealed systematic patterns in the distribution of tailoring enzymes, with 47 gene clusters encoding both a Pgm1 ligase homologue and precursor motifs with aspartic acid at position 4. This widespread occurrence of hybrid clusters combining biarylitide and pheganomycin biosynthetic machineries suggests a significant evolutionary relationship between these pathways and indicates broader implications for understanding the evolution of peptide natural product biosynthesis.

Taken together, our findings substantially expand our understanding of biarylitide biosynthetic diversity and reveal systematic patterns in the distribution of precursor sequences and tailoring enzymes across bacterial phyla. The discovery of novel precursor motifs and the structural elucidation of their cross-links through detailed NMR and MS labeling studies, combined with the identification of conserved relationships between specific precursor sequences and tailoring enzymes, provides new and comprehensive insights into the biosynthetic diversity and potential of this natural product family.

## Materials and Methods

### Generation of the Training Data Set

For the development of the ML classifier, a negative data set was assembled from P450s from the MIBiG database (*n* = 277), additional P450s from verified BGCs not in the MIBiG database (*n* = 12), predicted atropopeptides a BLAST search on WP_007820080.1 of tryptorubin A (*n* = 49) and P450s predicted to belong to other cyptide classes by Nam et al. (*n* = 787).^12^ The negative training data set was supplemented with P450s from the antiSMASH database,^32^ comprising 14,665 proteins annotated as ‘cytochrome P450. Concurrently, a positive data set was meticulously curated by combining 5 characterized known biarylitide P450s with 181 predicted putative biarylitide P450s from Zdouc et al.^10^ (*n* = 55) and Nam et al. (*n* = 126).^12^ To dereplicate both data sets, cdhit V4.8.1 was employed.^44^ Parameters were configured to cluster sequences with a minimum of 95% sequence similarity and to utilize a word size of 5. As a result of this dereplication process, the negative and positive data sets were refined to 9,761 and 106 sequences, respectively.

### Assembly of the Classification Data Set

For the assembly of the classification data set, 299,885 protein sequences were retrieved from the NCBI genpept database (18.03.2024).^33^ These sequences, which encompassed the term “p450” in their names and which had lengths ranging from 300 to 500 amino acids, were procured using the following query parameters: (P450­[Title] OR P450­[Protein Name]) NOT “Mycobacteroides abscessus”[Organism] NOT “Mycobacterium tuberculosis”[Organism] AND bacteria­[filter] AND (“300”[SLEN]: “500”[SLEN]).^33^ Proteins from *Mycobacteroides abscessus* and *Mycobacterium tuberculosis* were removed after examination of around 20 strains of the organisms did not yield to any results, because their inclusion would have doubled the size of the data set. To ensure the inclusion of all known biarylitide BGCs, the positive training data set was added to the classification data set. The method employed to assemble the data set mirrored that of the training data set.

### Data Preprocessing

For data preprocessing, sequences were aligned using Muscle version 3.8 ^45^ (gap open = −2, gap extend = −1, and center = −1) with a reference cytochrome P450 with a known crystal structure, AmphL, chain A from *Streptomyces nodosus* (PDB: 7SHI) in a multiple sequence alignment. The sequences were fragmented at positions 69, 78, 194, 228, 235, 377, and 383 of the reference to resemble the different functional regions of the cytochrome. To prevent overfitting, the sequences were translated into a simplified amino acid code.

### Training of the Classifier and Hyperparameter Optimization

The training of the classifier and hyperparameter optimization was modified from Biermann et al.^29^ The machine learning model was implemented using scikit-learn version 1.0.2 ^46^ for Python. The data set was balanced using the Random Over Sampler from the Python package Imbalanced-learn 0.9.0.^47^ A 60:40 split for the training data set and internal validation set was used. The number of overlapping *k*-mers of motifs with the length of four that occurred in at least half of the putative biarylitide sequences in each segment were used as features. Different classifiers were compared, and the Random Forest classifier was chosen because of its high balanced accuracy score (balanced accuracy = (recall+ specificity)/2) for biarylitide P450s (0.89). Hyperparameter tuning was performed to optimize the maximum amount of samples per leaf and the maximum tree depth, assessed on the obtained maximum balanced accuracy (optimal parameters: 3 and 12, respectively). Every hit with a score >0.25 was considered “positive”.

### CoreFinder

The Corefinder was adapted from Biermann et al.^29^ Potential ORFs encoding precursor and core peptides were investigated in the 3 kb genetic neighborhood, both upstream and downstream of the putative biarylitide-modifying cytochrome P450 genes in all relevant nucleotide records. Open reading frames (ORFs) ranging between 5 and 20 amino acids that encoded aromatic amino acids in positions 3 and 5 and had a ribosomal binding site up to 20 bp in front of the gene were selected. ORFs that were within completely annotated coding sequences (CDSs) were ignored. Overlapping cores were sorted out to remove duplicates with slightly longer putative leader peptide-encoding genes. If multiple hits were obtained, then the shortest hits were retained. Corefinder uses .fasta files containing protein identifiers and outputs a GenBank file containing the region 3 kb upstream and downstream with annotated precursor peptides.

### BGC Analysis and Phylogeny

The genomic context of the detected BGCs was further analyzed using RODEO2 to detect coencoded tailoring enzymes through Pfam domain identification.^34^ For phylogenetic analysis, protein sequences were aligned using MUSCLE,^45^ and the alignment was submitted to the IQ-tree Web server using standard settings.^48^ The resulting treefile was visualized and annotated using the iTOL Web server (Figure 2).^49^

### Bacterial Strains

*Actinocatenispora rupis* DSM45178, *Saccharothrix variisporea* DSM40234 were obtained from the German Collection of Microorganisms and Cell Cultures (DSMZ), and *Actinomadura hibisca* was a kind gift from Naicons, Milano, Italy.

### Synthesis ofl-tyrosine-(phenyl-3,5-d_2_) and Fmoc-l-tyrosine-(phenyl-3,5-d_2_)

The syntheses of l-tyrosine-(phenyl-3,5-d_2_) and Fmoc-l-tyrosine-(phenyl-3,5-d_2_) were performed according to previously reported protocols.^16^ To a solution of l-tyrosine (500 mg, 2.76 mmol) in D_2_O (20 mL), DCl (35% in D_2_O, 2 mL) was added. The mixture was refluxed overnight at 120 °C before being lowered to 40 °C and the solvent was evaporated under nitrogen to afford the crude product as a yellow solid. l-tyrosine-(phenyl-3,5-d_2_) (506 mg, 2.76 mmol) was dissolved in a solution of THF (4.1 mL), water (4.1 mL), and sodium bicarbonate (696 mg, 8.28 mmol) at room temperature. Fmoc-succinimide (1.12 g, 3.32 mmol) was added to the solution and stirred at room temperature overnight. The solvent was removed under reduced pressure. Flash chromatography yielded the desired product as light-yellow crystals.

### Peptide Synthesis and Deuteration of Histidine-(imidazole-2-d)

Peptide synthesis was performed using typical Fmoc-protected solid-phase peptide synthesis (SPPS) protocols. In brief, 2-chlorotrityl chloride resin (150 mg, Merck) was swelled in dichloromethane (DCM, 2 mL, 30 min). The resin was then washed three times with dimethylformamide (DMF, 2 mL, 30 s) before the first Fmoc-protected amino acid (0.05 mmol) was coupled to the resin overnight with *N,N*-diisopropylethylamine (DIPEA, 0.12 mmol) in DMF (3 mL). The resin was washed three times with DMF (2 mL, 30 s) before unreacted chlorotrityl groups were capped with methanol using a DMF/DIPEA/methanol (18:1:1) solution (3 mL). Deprotection of Fmoc protecting groups was achieved with 20% piperidine in DMF for 5 min, followed by coupling of subsequent Fmoc-protected amino acids (3 eq, 0.15 mmol) with COMU (0.15 mmol) and triethylamine (TEA, 0.3 mmol) for 1 h at room temperature. The peptide was cleaved using TFA/TIS/H_2_O (5 mL, 95:2.5:2.5, v:v′:v″) for 1 h at room temperature (2 h for sequences containing Arg­(Pbf)). The filtrate was concentrated under N_2_ gas, precipitated with cold diethyl ether (12 mL), centrifuged in a spark-free centrifuge (Spintron, GT-175), and washed three times with diethyl ether (5 mL). For peptides with deuterated histidine-(imidazole-2-d), deuteration was performed as previously reported.^50^ Briefly, the peptide was incubated in D_2_O at pH 8 at 50 °C for 72 h prior to lyophilization in DCl.^19^ NMR spectra of all synthesized peptides are provided in Figures S51–S82.

### Protein Expression and Purification

The expression and purification of cytochrome P450 and redox partner enzymes were performed as previously described.^19^ For cytochrome P450 expression, plasmids were first transformed into *E. coli* ArcticExpress (DE3) competent cells (NEB) using standard heat shock methodology. Cultures of 1 L of TB medium containing kanamycin (50 *μ*g/mL) and *δ*-aminolevulinic acid (0.1 mM) were inoculated with 10 mL of preculture. Cultures were incubated at 37 °C until the OD at 600 nm was between 0.6 −0.8 then cultures were cold-shocked for 30 min at 4 °C. IPTG (0.05 mM) was then added to each culture, and the cultures were incubated at 18 °C overnight. Cells were harvested by centrifugation and resuspended in buffer (50 mM Tris.HCl pH 8.0, 300 mM NaCl, 10 mM imidazole) with Benzonase (25 U/L cell culture) added prior to cell lysis via cell disruption (Avestin Emulsiflex C5). The cell lysate was clarified via centrifugation, and the supernatant was combined with equilibrated Ni-NTA agarose. The desired protein was eluted from Ni-NTA agarose using a buffer of 50 mM Tris.HCl pH 8.0, 300 mM NaCl, 500 mM imidazole prior to dilution of the protein a final concentration of 10 mM NaCl. Anion exchange chromatography (AEX) was conducted using a HiTrap Q HP AEX (Cytiva) column with elution over 20 column volumes from 10 mM – 1 M NaCl. Fractions containing the desired protein were combined and subjected to size exclusion chromatography using a Sepax SRT-10 SEC-300 column in buffer containing 50 mM Tris.HCl pH 7.4, 100 mM NaCl. Proteins were then stored at −80 °C.

For redox partner (PuR and PuxB A105 V) expression, plasmids were transformed into *E. coli* BL21 (DE3) competent cells (NEB) using standard heat shock methodology. Cultures of 1 L of LB medium containing kanamycin (50 *μ*g/mL) were inoculated with 10 mL of preculture, and incubated and induced as described above. Cells were harvested and lysed as described for cytochrome P450 expression. Due to the lack of histidine tag on the redox partner constructs, each protein was instead subjected to two AEX purifications as described above, using a HiTrap Q HP AEX (Cytiva) column and a Resource Q AEX (Cytiva) column. Size exclusion chromatography was performed as described previously, and proteins were stored at −80 °C.

### Cyclization Reactions

Cyclization reactions were performed as previously reported.^19^ Briefly, 100 *μ*L reactions containing HEPES (50 mM) pH 7.0, NaCl (50 mM), P450 (0.5 *μ*M), PuR (0.5 *μ*M), PuxB A105 V mutant (2.5 *μ*M),^51^ glucose (0.33%), glucose dehydrogenase (0.033 mg/mL, Sigma–Aldrich), peptide substrate (50 *μ*M) and NADH (200 *μ*M) were incubated overnight at 30 °C with 300 rpm shaking. Reactions were quenched with methanol containing 0.1% formic acid (FA, 350 *μ*L) and centrifuged at 15000 rpm for 15 min before the supernatant was removed and dried using a concentrator (Eppendorf). The dried sample was resuspended in 40 *μ*L of acetonitrile/water (1:4, 0.1% FA) before LC-MS analysis. Reactions were conducted in triplicate, and mean % conversion was calculated by determining the % of starting material that had converted to cross-linked product.

### LC-MS Analysis

Analysis was carried out on a Shimadzu high-performance liquid chromatography system coupled to a mass spectrometer LC-MS-2020 (ESI, operating in positive and negative mode) equipped with an SPD-20A Prominence photodiode array detector and an LC-20AD solvent delivery module. Liquid chromatography separation was performed using an Agilent ZORBAX 300SB-C_18_ 5 *μ*m column. All peptides were analyzed using a 5–95% gradient of acetonitrile (Fisher Scientific) +0.1% formic acid (Sigma-Aldrich) in water (Fisher Scientific) +0.1% formic acid in 20 min with a flow rate of 1 mL/min. Enzymatic reactions were analyzed on a gradient of 0–60% of acetonitrile +0.1% formic acid in 35 min. Mass spectra were acquired using full scan (200–1200 *m*/*z*) ESI operating in both positive and negative modes.

### HRMS Analysis

High-resolution mass spectrometry measurements were performed on an Orbitrap Eclipse Tribrid mass spectrometer (Thermo Scientific) coupled to a Vanquish Neo UHPLC (Thermo Scientific), an Acclaim PepMap RSLC analytical column (75 μm x 50 cm, nanoViper, C18, 2 μm, 100 Å; Thermo Scientific), and an Acclaim PepMap 100 trap column (100 μm x 2 cm, nanoViper, C18, 5 μm, 100 Å; Thermo Scientific). The peptides were separated by increasing concentrations of 80% acetonitrile/0.1% formic acid at a flow of 250 nL/min over 54 min. The instrument was operated in alternating data-dependent acquisition (DDA) and parallel reaction monitoring (PRM) cycles in such that 10 ms2 scans were triggered per survey ms1 scan followed by several targeted ms2 scans to ensure fragmentation of predefined, sample-dependent *m*/*z* precursors. Each survey ms1 scan (375–1575 *m*/*z*) was acquired with a resolution of 60,000 and a normalized AGC (automatic gain control) target of 200%. Dynamic exclusion was set to 310 s after one occurrence. The 5–10 most intense ions were selected for HCD fragmentation (fixed normalized collision energy mode, 30% HCD collision energy) with a resolution of 30,000, a normalized absolute AGC target of 5e4, and a fixed dynamic scan range based on precursor *m*/*z*. Subsequent targeted ms2 scans were acquired with essentially identical settings. The raw data files were analyzed with QualBrowser (XCalibur 3.0.63, Thermo Scientific) to view spectra and to generate extracted ion chromatograms.

### NMR Analysis

Nuclear magnetic resonance (NMR) spectra of **Nle-2** were collected using a Bruker Avance III HD equipped with a 16.4 T magnet and 5 mm TCI cryoprobe operating at 700 MHz (^1^H), 176 MHz (^13^C), 71 MHz (^15^N). ^1^H, ^13^C and ^15^N chemical shifts (δ) are reported in parts per million (ppm). ^1^H and ^13^C spectra are referenced to residual solvent signals. ^15^N spectra are referenced to liquid ammonia δ_N_ 0 ppm using the Bruker program Xiref. Coupling constants (*J*) are reported in Hz. ^15^N-HMBC spectra reported herein were collected using the Bruker pulse program hmbcf3gpndqf using various *J*_NH_ values as specified.

NMR spectra of other peptides were collected at 298 K on a Bruker Avance III NMR spectrometer equipped with a 14.1 T magnet and 5 mm TCI cryoprobe, operating at 600 MHz (^1^H), 150 MHz (^13^C) or on a Bruker Avance III NMR spectrometer equipped with a 9.4 T magnet and 5 mm BBFO probe, operating at 400 MHz (^1^H), 100 MHz (^13^C). ^1^H and ^13^C chemical shifts are reported in ppm and are referenced to the residual solvent signals.

### Cloning of Biarylitide BGCs

The biarylitide BGC from *S. variisporea* *sav* was amplified from isolated genomic DNA via PCR with specific primers SavAOHT_for: TGCGCGAGGCGGTGTCGTGACGGCGGATTGGGAGCG and SavAOHT_rev: GCAGGTCGACTCTAGAGAGGTCAGCCCAGGTAGTCCTCTGC. Then, linear amplicons were cloned into a linearized integrating plasmid pSET_152-ermE* via Gibson assembly. Post-PCR purification involved removing any remaining template DNA by adding 1 μL DpnI and incubating at 37 °C for 1 h. For the assembly, 5 μL of DNA (with insert and vector mixed at a 3:1 ratio) were combined with 15 μL of Gibson assembly master mix and incubated at 50 °C for 60 min. The resulting mixture was purified using the DNA Clean & Concentrator-5 Kit (Zymo Research), eluted in 5 μL H_2_O, and subsequently used for chemical transformation into *E. coli*.

### Conjugation Between *E. coli* and *Streptomyces*

pSET_152-ermE*_savAOHT was transformed into electrocompetent *E. coli* ET12567/pUZ8002 and plated on LB agar containing chloramphenicol (25 μg/mL), kanamycin (50 μg/mL), and apramycin (50 μg/mL). After overnight incubation, a single colony was inoculated into 5 mL of LB medium with the appropriate antibiotics and grown overnight to serve as a preculture. The following day, 10 mL of LB medium was inoculated with 100 μL of the preculture and incubated until an OD_600_ of 0.5 was reached. The cells were harvested by centrifugation (6000× *g*, 5 min, 4 °C) and washed twice with cooled LB containing 10 mM MgCl_2_ to remove antibiotics. The cells were then resuspended in 500 μL of LB and kept on ice until conjugation. For the recipient preparation, 100 μL of spores were thawed and washed twice with 1 mL of 2×YT medium. The spores were resuspended in 500 μL of fresh 2×YT and activated by heating to 50 °C for 10 min. Spores were allowed to cool down to room temperature before proceeding with conjugation. For conjugation, the donor and recipient were mixed in a 1.5 mL tube. The bacterial mixture was centrifuged (13,000× *g*, 1 min), and the pellet resuspended in 100 μL TSB. This mixture was then spread on an MS agar plate containing 10 mM MgCl_2_ and incubated at 30 °C. After 20 h, the plate was treated with nalidixic acid (0.5 mg) and apramycin (1 mg), and incubation was continued until ex-conjugants emerged. Single colonies were picked and transferred to new MS agar plates with apramycin (50 μg/mL) and nalidixic acid (25 μg/mL) to confirm the ex-conjugants. Following 5–7 d of incubation, colonies were validated through colony PCR, and positive clones were cultivated to test production.

### Heterologous Production of Biarylitides

*S. coelicolor* M1152 pSET- ermE*_savAOHT was utilized for the production of biarylitide YVH (**5**). The initial culture, inoculated from spores, was grown in 20 mL of production medium comprising a 1:1 mixture of YEME broth and TSB medium in a 100 mL Erlenmeyer flask. The culture conditions were maintained at 30 °C with shaking at 200 rpm for 3 d. This seed culture was then used to inoculate 80 mL of TSB/YEME (1:1) in a 300 mL Erlenmeyer flask containing 5 g of 3 mm glass beads and grown under the same conditions for another 3 d. This secondary culture was used to inoculate five 300 mL Erlenmeyer flasks, each containing 80 mL of TSB/YEME (1:1) and 5 g of glass beads. The cultures were incubated for 7 d at 30 °C, 200 rpm, with the addition of 50 μg/mL apramycin.

### Extraction and Isolation of Biarylitides

For the isolation of biarylitide YVH, the cultures of *S. coelicolor* pSET_ermE*-*savAOHT* were processed as follows: After 7 d of incubation, cultures were centrifuged at 10,000 rpm for 20 min. The supernatants containing the peptides were incubated with 5% (w/v) HP-20 resin (Supelco/Bellefonte, USA), which had been activated earlier by soaking in methanol for 20 min and subsequently washed twice with water. The total resin particles in water were transferred to the culture supernatants and incubated at room temperature for 1 h with continuous shaking. After incubation, the resin particles were added to appropriate columns, and the flow-through was discarded. The resin was washed with 5% acetonitrile and 0.1% formic acid and eluted with methanol. The eluate was concentrated under reduced pressure. The dried extract was fractionated with Flash chromatography using a Reveleris C18 column (220 g, 40 μm). The mobile phase consisted of: Phase 0.1% TFA in H_2_O (A), and 0.1% TFA in MeOH (B). The elution gradient applied was as follows: 5% B for 5 min, increasing to 25% B over 15 min, followed by 100% B from 25 to 30 min. Fractions containing biarylitide YVH were identified, dried, and further purified by HPLC using a semipreparative Nucleodur C18 column (250 × 8 mm, 5 μm, Macherey-Nagel) with a C18 precolumn. The isocratic elution was performed with 70/30 H_2_O/MeOH with 0.05% TFA at a flow rate of 2.5 mL/min. The purification was repeated twice under the same conditions to yield 8.7 mg of pure biarylitide YVH as a white powder from a total of 15 L of cultivation broth.

### Bioactivity Assays

Antibacterial activity of **5** was evaluated against *Escherichia coli* DH5α and *Bacillus subtilis* JH642 using a disc diffusion assay. Pure **5** was dissolved in MeOH and applied to sterile filter paper discs at concentrations of 32 μg/mL, 64 μg/mL, and 128 μg/mL. The discs were subsequently placed onto Mueller–Hinton agar plates that had been previously inoculated with the respective bacterial strains. MeOH was used as the negative control, while kanamycin and carbenicillin served as positive controls for *E. coli* DH5α and *B. subtilis* JH642, respectively. Antibacterial activity was evaluated following incubation at 37 °C for 24 h. All experiments were performed in duplicate. Antifungal activity of **5** was evaluated against *Botrytis cinerea* DSM 877 using the disc diffusion method. *B. cinerea* was subcultured on a Potato Dextrose agar plate for 5 d at 25 °C. Then, a 5 mm-plug of the fungus was placed in the center of a plate, 2 cm away from the sterile filter paper discs impregnated with the test compound at a concentration of 64 μg/mL. MeOH was used as a negative control. Antifungal activity was evaluated after 5 d of incubation at 25 °C.

Cytotoxic activity of **5** was evaluated using the human acute lymphoblastic leukemia cell line SEM. Cells (2 × 10^6) were cultured in a 6-well plate in 2 mL RPMI growth medium supplemented with 10% FBS, 1% l-glutamine +1% penicillin/streptomycin, and treated with 1 μL of a 40 mM stock solution of the test compound in DMSO, resulting in a final concentration of 20 μM. The histone deacetylase inhibitor entinostat was used as a positive control. Following incubation for 24 h under standard culture conditions, 40 μL of the cell suspension (approximately 4 × 10^4 cells) was transferred and mixed with 60 μL of culture medium and 100 μL of CellTiter-Glo 2.0 reagent (Promega). The mixture was shaken at 200 rpm for 2 min to induce cell lysis and then incubated for 10 min at room temperature to allow stabilization of the luminescent signal. Luminescence was measured using a Varioskan Flash microplate reader (Thermo Scientific). Cell viability was determined based on ATP-dependent luminescence and normalized to the DMSO-treated control. All measurements were performed in technical triplicates.

## Supplementary Material



## Notes

Experimental and analytical data; Figures S1–S132, Tables S1–S3 (PDF)
