# Supplementary material for: A Machine Learning-Based Genome Mining Approach Reveals Unprecedented Biarylitide Diversity
Source: JACS Au. 2026 Jul 13;6(7):3952–65. doi: 10.1021/jacsau.6c00500 (PMC13417212; doi:10.1021/jacsau.6c00500)
Supplement: Supplementary file 1 [file au6c00500_si_001.pdf]

Supplementary Information for: A machine learning-based genome mining approach reveals unprecedented biarylitide diversity

Leo Padva[1#], Jemma Gullick[2,3#], Friederike Biermann[4], Laura J. Coe[3,5], Yongwei Zhao[2,3], Sam Tucker[2,3], Lukas Zimmer[6], Julien Tailhades[2,3], Stefan Kehraus[1], Ralf B. Schittenhelm[2,7], James J. De Voss[3,5], Eric J.N. Helfrich[4,8], Max J. Cryle[2,3*], Max Crüsemann[1,6*]

Affiliations:

1: Institute of Pharmaceutical Biology, University of Bonn, 53115 Bonn, Germany

2: Department of Biochemistry and Molecular Biology, The Monash Biomedicine Discovery Institute, Monash University, Clayton, VIC 3800, Australia. *E-mail: max.cryle@monash.edu

3: ARC Centre of Excellence for Innovations in Peptide and Protein Science, Clayton, VIC 3800, Australia.

4: Institute of Molecular Biosciences, Goethe University Frankfurt, Max-von-Laue Strasse 9, 60438 Frankfurt am Main, Germany

5: School of Chemistry and Molecular Biosciences, The University of Queensland, Brisbane, QLD 4067, Australia

6: Institute of Pharmaceutical Biology, Goethe University Frankfurt, 60438 Frankfurt am Main, Germany. *E-mail: cruesemann@em.uni-frankfurt.de

7: The Monash Proteomics and Metabolomics Platform, Monash University, Clayton, VIC 3800, Australia.

8: Senckenberg Society for Nature Research, Senckenberganlage 25, 60325 Frankfurt am Main, Germany

#: These authors contributed equally

Table of Contents

1. ***byt* BGC from *S. nassauensis* and overview on detected BGCs** 5
2. **Structures of peptides** 20
3. **Structural characterisation of peptides** 24
4. **LCMS characterisation of peptides** 28
5. **NMR characterisation of peptides** 72
6. **Analysis of crosslinking reactions with AchB** 89
7. **Analysis of crosslinking reactions with AciB** 104
8. **Analysis of crosslinking reactions with SlyP** 113
9. **Characterisation of *in vivo* production of 5** 121
10. **Analysis of crosslinking reactions with SavB** 126
11. **AlphaFold3 predictions of novel P450 motifs** 132

Table of Items

Figure S1. The *Stackebrandtia nassauensis byt* BGC 5
Figure S2. Comparative analysis of all *byt* BGCs in the conserved MRYWY clade....5
Table S1. Biarylitide P450s identified through machine learning based genome mining..6
Figure S3. Structures of peptides **1**, **Nle-1** and **Nle-2** 20
Figure S4. Structures of peptides **Nle-3**....21
Figure S5. Structures of peptides **Nle-4**....22
Figure S6. Structures of peptides **5**, **Nle-6** and **Nle-7** 23
Figure S7. LCMS characterisation of **Nle-1** 28
Figure S8. Annotated MS^2 spectrum of **Nle-1**....29
Figure S9. LCMS characterisation of **Nle-1-(Ala4)** 30
Figure S10. Annotated MS^2 spectrum of **Nle-1-(Ala4)** 31
Figure S11. LCMS characterisation of **Nle-1-(Val4)**....32
Figure S12. Annotated MS^2 spectrum of **Nle-1-(Val4)**....33
Figure S13. LCMS characterisation of **Nle-1-(Leu4)**....34
Figure S14. Annotated MS^2 spectrum of **Nle-1-(Leu4)**....35
Figure S15. LCMS characterisation of **Nle-1-(Tyr4)** 36
Figure S16. Annotated MS^2 spectrum of **Nle-1-(Tyr4)**....37
Figure S17. LCMS characterisation of **Nle-1-(Glu4)**....38
Figure S18. Annotated MS^2 spectrum of **Nle-1-(Glu4)** 39
Figure S19. LCMS characterisation of **Nle-1-(Tyr5)**....40
Figure S20. Annotated MS^2 spectrum of **Nle-1-(Tyr5)**....41
Figure S21. LCMS characterisation of **Nle-1-(Trp5)** 42
Figure S22. Annotated MS^2 spectrum of **Nle-1-(Trp5)** 43
Figure S23. LCMS characterisation of **Nle-3-(Trp4)** 44
Figure S24. Annotated MS^2 spectrum of **Nle-3-(Trp4)** 45
Figure S25. LCMS characterisation of **Nle-3-(Ala4)** 46
Figure S26. Annotated MS^2 spectrum of **Nle-3-(Ala4)** 47
Figure S27. LCMS characterisation of **Nle-3-(Val4)**....48
Figure S28. Annotated MS^2 spectrum of **Nle-3-(Val4)**....49
Figure S29. LCMS characterisation of **Nle-3-(Leu4)**....50
Figure S30. Annotated MS^2 spectrum of **Nle-3-(Leu4)**....51
Figure S31. LCMS characterisation of **Nle-3-(3,5-d$_2$-Tyr3, Trp4)** 52
Figure S32. Annotated MS^2 spectrum of **Nle-3-(3,5-d$_2$-Tyr3, Trp4)** 53
Figure S33. LCMS characterisation of **Nle-3-(Trp4, 3,5-d$_2$-Tyr5)** 54
Figure S34. Annotated MS^2 spectrum of **Nle-3-(Trp4, 3,5-d$_2$-Tyr5)** 55
Figure S35. LCMS characterisation of **Nle-3-(Trp4, 3,5-d$_2$-Tyr3,5)** 56
Figure S36. Annotated MS^2 spectrum of **Nle-3-(Trp4, 3,5-d$_2$-Tyr3,5)** 57
Figure S37. LCMS characterisation of **Nle-3-(3,5-d$_2$-Tyr3, Leu4)**....58
Figure S38. Annotated MS^2 spectrum of **Nle-3-(3,5-d$_2$-Tyr3, Leu4)**....59
Figure S39. LCMS characterisation of **Nle-3-(Leu4, 3,5-d$_2$-Tyr5)**....60
Figure S40. Annotated MS^2 spectrum of **Nle-3-(Leu4, 3,5-d$_2$-Tyr5)**....61
Figure S41. LCMS characterisation of **Nle-3-(Leu4, His5)**....62
Figure S42. Annotated MS^2 spectrum of **Nle-3-(Leu4, His5)**....63
Figure S43. LCMS characterisation of **Nle-3-(Leu4, Trp5)**....64
Figure S44. Annotated MS^2 spectrum of **Nle-3-(Leu4, Trp5)**....65

Figure S45. LCMS characterisation of **Nle-6** 66
Figure S46. Annotated MS^2 spectrum of **Nle-6** 67
Figure S47. LCMS characterisation of **Nle-6-(3,5-d$_2$-Tyr3, Leu4)** 68
Figure S48. Annotated MS^2 spectrum of **Nle-6-(3,5-d$_2$-Tyr3, Leu4)** 69
Figure S49. LCMS characterisation of **Nle-6-(Leu4, 2-d-His5)** 70
Figure S50. Annotated MS^2 spectrum of **Nle-6-(Leu4, 2-d-His5)** 71
Figure S51. ^{1}H NMR spectrum of **Nle-1** 72
Figure S52. ^{13}C NMR spectrum of **Nle-1** 72
Figure S53. ^{1}H NMR spectrum of **Nle-1-(Ala4)** 73
Figure S54. ^{13}C NMR spectrum of **Nle-1-(Ala4)** 73
Figure S55. ^{1}H NMR spectrum of **Nle-1-(Val4)** 74
Figure S56. ^{13}C NMR spectrum of **Nle-1-(Val4)** 74
Figure S57. ^{1}H NMR spectrum of **Nle-1-(Leu4)** 75
Figure S58. ^{13}C NMR spectrum of **Nle-1-(Leu4)** 75
Figure S59. ^{1}H NMR spectrum of **Nle-1-(Tyr4)** 76
Figure S60. ^{13}C NMR spectrum of **Nle-1-(Tyr4)** 76
Figure S61. ^{1}H NMR spectrum of **Nle-1-(Glu4)** 77
Figure S62. ^{13}C NMR spectrum of **Nle-1-(Glu4)** 77
Figure S63. ^{1}H NMR spectrum of **Nle-1-(Tyr5)** 78
Figure S64. ^{13}C NMR spectrum of **Nle-1-(Tyr5)** 78
Figure S65. ^{1}H NMR spectrum of **Nle-1-(Trp5)** 79
Figure S66. ^{13}C NMR spectrum of **Nle-1-(Trp5)** 79
Figure S67. ^{1}H NMR spectrum of **Nle-3-(Trp4)** 80
Figure S68. ^{13}C NMR spectrum of **Nle-3-(Trp4)** 80
Figure S69. ^{1}H NMR spectrum of **Nle-3-(Ala4)** 81
Figure S70. ^{13}C NMR spectrum of **Nle-3-(Ala4)** 81
Figure S71. ^{1}H NMR spectrum of **Nle-3-(Val4)** 82
Figure S72. ^{13}C NMR spectrum of **Nle-3-(Val4)** 82
Figure S73. ^{1}H NMR spectrum of **Nle-3-(Leu4)** 83
Figure S74. ^{13}C NMR spectrum of **Nle-3-(Leu4)** 83
Figure S75. ^{1}H NMR spectrum of **Nle-3-(Leu4, His5)** 84
Figure S76. ^{13}C NMR spectrum of **Nle-3-(Leu4, His5)** 84
Figure S77. ^{1}H NMR spectrum of **Nle-3-(Leu4, Trp5)** 85
Figure S78. ^{13}C NMR spectrum of **Nle-3-(Leu4, Trp5)** 85
Figure S79. ^{1}H NMR spectrum of **Nle-6** 86
Figure S80. ^{13}C NMR spectrum of **Nle-6** 86
Figure S81. ^{1}H NMR spectrum of **Fmoc-Tyr(3,5-d$_2$)-OH** 87
Figure S82. ^{13}C NMR spectrum of **Fmoc-Tyr(3,5-d$_2$)-OH** 88
Figure S83. Crosslinking assay data for **Nle-1** with AchB 89
Figure S84. HRMS of **Nle-2** formed from turnover of **Nle-1** with AchB 90
Figure S85. Crosslinking assay data for **Nle-1-(Ala4)** with AchB 91
Figure S86. Crosslinking assay data for **Nle-1-(Val4)** with AchB 92
Figure S87. Crosslinking assay data for **Nle-1-(Leu4)** with AchB 93
Figure S88. Crosslinking assay data for **Nle-1-(Tyr4)** with AchB 94
Figure S89. Crosslinking assay data for **Nle-1-(Glu4)** with AchB 95
Figure S90. Crosslinking assay data for **Nle-1-(Tyr5)** with AchB 96
Figure S91. Crosslinking assay data for **Nle-1-(Trp5)** with AchB 97

Figure S92. ^{1}H spectrum of **Nle-2** in DMF-d_7 ... 98
Figure S93. 1D ROESY spectrum of **Nle-2** in DMF-d7 stacked with ^{1}H spectrum ... 98
Figure S94. ^{1}H-^{1}H-COSY spectrum of **Nle-2** in DMF-d_7 ... 99
Figure S95. 2D TOCSY spectrum of **Nle-2** in DMF-d7. ... 99
Figure S96. ^{13}C-HSQC Edit spectrum of **Nle-2** in DMF-d_7 optimised for $^1J_{CH}$ = 145 Hz. ... 100
Figure S97. ^{13}C-HMBC spectrum of **Nle-2** in DMF-d_7 ... 100
Figure S98. ^{13}C spectrum of **Nle-2** in DMF-d_7 ... 101
Figure S99. ^{15}N-HMBC spectrum of **Nle-2** in DMF-d_7 optimised for $^3J_{NH}$ = 4 Hz ... 101
Figure S100. Key NMR correlations used in the characterisation of **Nle-2** ... 102
Table S2. NMR data for crosslinked **Nle-2** ... 102
Figure S101. Crosslinking assay data for **Nle-3-(Trp4)** with AciB ... 104
Figure S102. Crosslinking assay data for **Nle-3-(3,5-d_2-Tyr3, Trp4)** with AciB ... 105
Figure S103. Crosslinking assay data for **Nle-3-(Trp4, 3,5-d_2-Tyr5)** with AciB ... 106
Figure S104. Crosslinking assay data for **Nle-3-(Trp4, 3,5-d_2-Tyr3,5)** with AciB ... 107
Figure S105. Crosslinking assay data for **Nle-3-(Ala4)** with AciB ... 108
Figure S106. Crosslinking assay data for **Nle-3-(Val4)** with AciB ... 109
Figure S107. Crosslinking assay data for **Nle-3-(Leu4)** with AciB ... 110
Figure S108. Crosslinking assay data for **Nle-3-(Leu4, His5)** with AciB ... 111
Figure S109. Crosslinking assay data for **Nle-3-(Leu4, Trp5)** with AciB ... 112
Figure S110. Crosslinking assay data for **Nle-3-(Ala4)** with SlyP ... 113
Figure S111. Crosslinking assay data for **Nle-3-(Val4)** with SlyP ... 114
Figure S112. Crosslinking assay data for **Nle-3-(Leu4)** with SlyP ... 115
Figure S113. Crosslinking assay data for **Nle-3-(3,5-d_2-Tyr3, Leu4)** with SlyP ... 116
Figure S114. Crosslinking assay data for **Nle-3-(Leu4, 3,5-d_2-Tyr5)** with SlyP ... 117
Figure S115. Crosslinking assay data for **Nle-3-(Trp4)** with SlyP ... 118
Figure S116. Crosslinking assay data for **Nle-3-(Leu4, His5)** with SlyP ... 119
Figure S117. Crosslinking assay data for **Nle-3-(Leu4, Trp5)** with SlyP ... 120
Figure S118. ^{1}H NMR spectrum of **5** in MeOH-d_4 (600 MHz) ... 121
Figure S119. ^{13}C NMR spectrum of **5** in MeOH-d_4 (150 MHz) ... 122
Figure S120. ^{1}H-^{1}H COSY spectrum of **5** in MeOH-d_4 (600 MHz) ... 122
Figure S121. ^{1}H-^{13}C HSQC spectrum of **5** in MeOH-d_4 (600 MHz) ... 123
Figure S122. ^{1}H-^{13}C HMBC spectrum of **5** in MeOH-d_4 (600 MHz) ... 123
Figure S123. ^{1}H-^{1}H ROESY spectrum of **5** in MeOH-d_4 (600 MHz) ... 124
Table S3. NMR assignments of biarylitide YVH (**5**) ... 125
Figure S124. Crosslinking assay data for **Nle-6** with SavB ... 126
Figure S125. Crosslinking assay data for **Nle-6-(3,5-d_2-Tyr3, Leu4)** with SavB ... 127
Figure S126. Crosslinking assay data for **Nle-6-(Leu4, 2-d-His5)** with SavB ... 128
Figure S127. LCMS analysis of products from enzymatic reaction of SavB with **A) Nle-6-(Leu4, 3,5-d2-Tyr5)** and **B) Nle-6-(Leu4, 2-d-His5)** ... 129
Figure S128. Crosslinking assay data for **Nle-3-(Leu4)** with SavB ... 130
Figure S129. Comparison of products formed from turnover of **Nle-3-(Leu4)** with AciB and SavB ... 130
Figure S130. Crosslinking assay data for **Nle-3-(Leu4, Trp5)** with SavB ... 131
Figure S131. Comparison of products formed from turnover of **Nle-3-(Leu4, Trp5)** with $P450_{Blt}$, SavB, SlyP and AciB ... 131
Figure S132. Predicted structures of novel biarylitide P450 motifs with AlphaFold3 ... 132

1. *byt* BGC from *S. nassauensis* and overview on detected BGCs

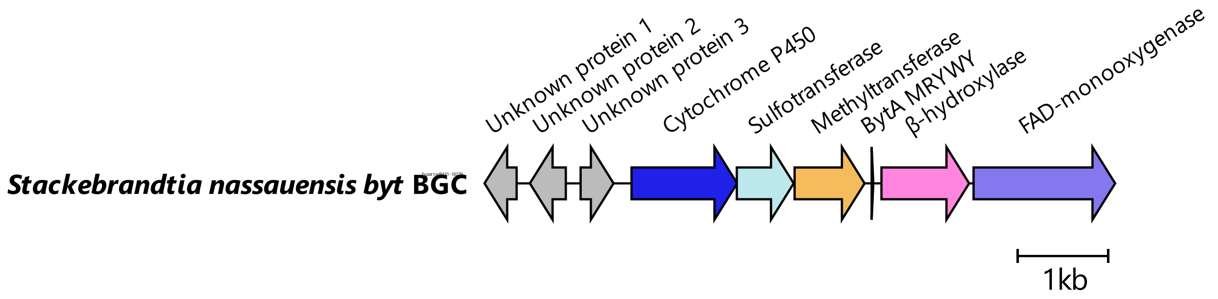

Figure S1. The *Stackebrandtia nassauensis byt* BGC showing the atypical positioning and sequence of *bytA*. The gene cluster includes genes encoding a sulfotransferase, methyltransferase, β-hydroxylase, and FAD monooxygenase.

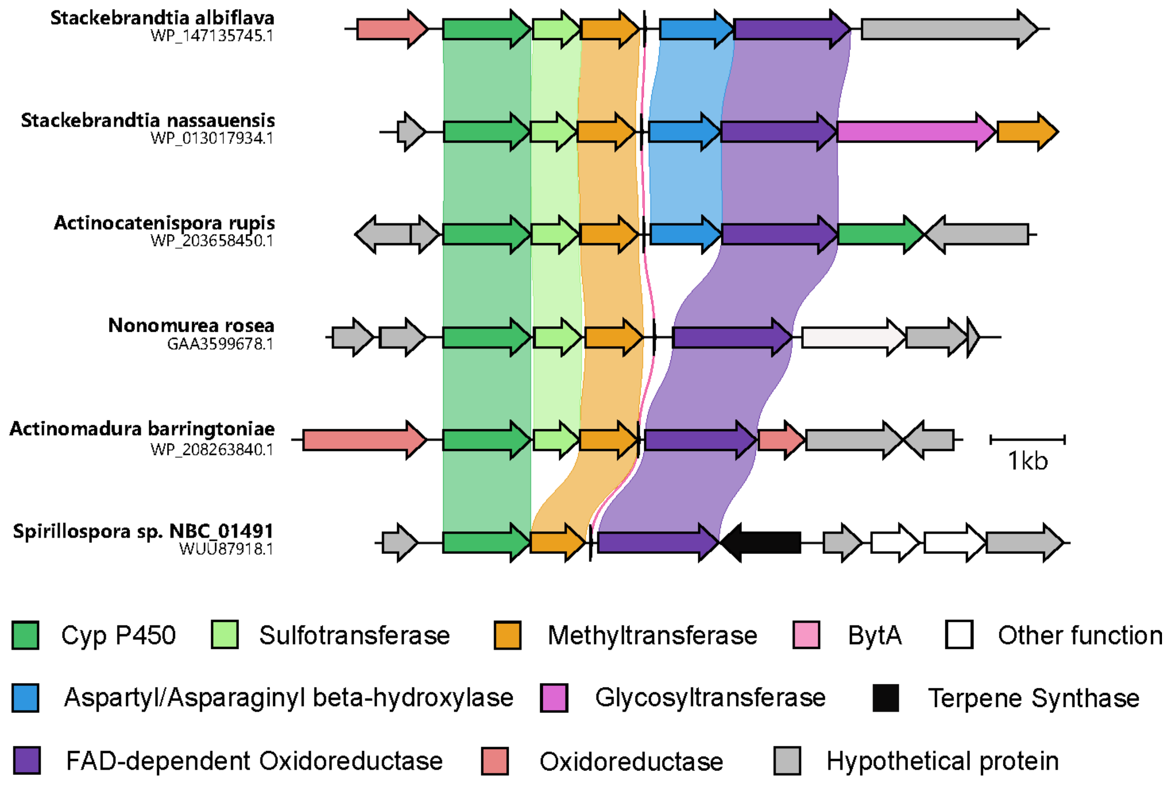

Figure S2. Comparative analysis of all *byt* BGCs in the conserved MRYWY clade.

Table S1. Biarylitide P450s identified through machine learning based genome mining. The table presents protein reference IDs, bacterial species, precursor sequences, number of precursors, and co-occurring proteins for each P450 enzyme. The associated phylogenetic tree is presented in Figure 2.

Protein reference	Species	Precursor sequence	Number of precursors	Co-occuring proteins
AIK38645.1	*Bacillus pseudomycoides*	MNYNH*	1	
CUB11028.1	*Bacillus cereus*	MIYDH*	1	
GAA1025571.1	*Amycolatopsis albidoflavus*	MLYRH*	1	
GAA2260327.1	*Actinomadura glauciflava*	MRYLH*	1	
GAA4057093.1	*Actinomadura miaoliensis*	MKYIW*	1	
GAA4139197.1	*Actinomadura keratinilytica*	MKYLW*	1	
GAA4372804.1	*Actinomadura verrucosospora*	MRYLH*	1	
GAA4493708.1	*Actinoallomurus oryzae*	MRYVH*	1	
GGV42769.1	*Actinomadura cremea*	MRYLH*	1	
PEP54637.1	*Bacillus pseudomycoides*	MNYLH*	1	
PGX89698.1	*Bacillus cereus*	MNYIH*	1	
TNP01825.1	*Bacillus pacificus*	MNYNH*	1	
WP_015623560.1	*Actinoplanes sp.* N902-109	MQYDH*	1	Methyltransferase
WP_026422380.1	*Actinokineospora inagensis* DSM 44258	MSYDH*	1	PGM_ligase
WP_034520669.1	*Actinomadura rifamycini* DSM 43936	MRYLH*	1	Hydroxylase, Oxidoreductase, Oxidoreductase, Methyltransferase, Acetyltransferase, Protease/Peptidase, Sulfotransferase
WP_067473018.1	*Actinomadura hibisca* NBRC 15177	MKYWH*	1	additional_P450, Hydroxylase, Methyltransferase
WP_067804628.1	*Actinomadura formosensis* NBRC 14204	MRYLH*	1	Hydroxylase, Methyltransferase, Protease/Peptidase
WP_075974605.1	*Actinokineospora bangkokensis*	MSYDHIS*	1	additional_P450

WP_080039781.1	*Actinomadura parvosata subsp. kistnae*	MRYRH*	1	Hydroxylase
WP_098623608.1	*Bacillus pseudomycoides*	MNYNH*	1	
WP_113692517.1	*Amycolatopsis albispora*	MDYPH*	1	Methyltransferase, Protease/Peptidase
WP_117356064.1	*Actinomadura logoneensis*	MKYWH*	1	
WP_122195090.1	*Actinomadura harenae*	MKYWH*	1	Hydroxylase, Methyltransferase
WP_125616065.1	*Actinomadura sp.* WAC 06369	MRYLH*	1	Hydroxylase, Methyltransferase, Acetyltransferase, Protease/Peptidase, Sulfotransferase
WP_127361668.1	*Actinacidiphila soli*	MSYDH*	1	Hydroxylase, Methyltransferase, PGM_ligase
WP_138637966.1	*Actinomadura geliboluensis*	MRYLH*	1	Hydroxylase, Methyltransferase, Protease/Peptidase, Sulfotransferase
WP_149261402.1	*Actinomadura sp.* K4S16	MRYLH*	1	Hydroxylase, Methyltransferase, Protease/Peptidase, Sulfotransferase
WP_157429744.1	*Actinomadura oligospora* ATCC 43269	MKYWH*	1	Hydroxylase, Methyltransferase
WP_160826862.1	*Actinomadura sp.* J1-007	MRYRH*	1	Hydroxylase, Acetyltransferase, Protease/Peptidase
WP_169813928.1	*Actinomadura kijaniata* NBRC 14229	MRHVH*	1	Methyltransferase, Sulfotransferase
WP_179846876.1	*Actinomadura luteofluorescens*	MRYLH*	1	Hydroxylase, Methyltransferase, Protease/Peptidase, Sulfotransferase
WP_182847545.1	*Actinomadura namibiensis*	MRHVH*	1	Methyltransferase, Sulfotransferase
WP_185026858.1	*Actinomadura coerulea*	MRYLH*	1	Hydroxylase, Methyltransferase, Protease/Peptidase, Sulfotransferase
WP_187242675.1	*Actinomadura alba*	MRYIH*	1	Hydroxylase, Aminotransferase, Methyltransferase,

				Protease/Peptidase, Sulfotransferase
WP_203405941.1	*Archangium violaceum*	MNYLH*	1	Methyltransferase
WP_203658450.1	*Actinocatenispora rupis*	MRYWY*	1	Hydroxylase, Methyltransferase, Protease/Peptidase, Sulfotransferase
WP_208263840.1	*Actinomadura barringtoniae*	MRYWY*	1	Oxidoreductase, Methyltransferase, Sulfotransferase
WP_220506603.1	*Amycolatopsis dendrobii*	MLYVH*	1	Acetyltransferase
WP_221320770.1	*Actinoplanes sp.* L3-i22	MRYFH*	1	Methyltransferase
WP_329238118.1	*Actinoallomurus sp.* NBC_01490	MRYVH*	1	Protease/Peptidase
WP_097787522.1	*Bacillus sp.* FSL K6-6038	MNYNH*	1	
WP_088119720.1	*Bacillus thuringiensis*	MNYNH*	1	Oxidoreductase
WP_212211904.1	*Bradyrhizobium sp.*	MLWPH*	1	Protease/Peptidase
WP_131770571.1	*Candidatus Protofrankia californiensis*	MIYDH*	1	Hydroxylase, Methyltransferase, PGM_ligase
WP_122147575.1	*Cellulomonas triticagri*	MTYNHIV*	1	PGM_ligase, Protease/Peptidase
GAA1714241.1	*Fodinicola feengrottensis*	MRYLH*	1	
WP_073388255.1	*Jatrophihabitans endophyticus*	MTYVH*	1	Oxidoreductase, Protease/Peptidase
WP_054292478.1	*Kibdelosporangium phytohabitans*	MNYRH*	1	
WP_052478416.1	*Kibdelosporangium sp.* MJ126-NF4	MHYKH*	1	Aminotransferase
WP_123563405.1	*Kitasatospora cineracea*	MSYDH*	1	Aminotransferase, PGM_ligase
WP_184914010.1	*Kitasatospora gansuensis*	MRYDY*	1	Methyltransferase
WP_184933437.1	*Kitasatospora kifunensis*	MSYDH*	1	Hydroxylase, Oxidoreductase, PGM_ligase
GAA1228896.1	*Kitasatospora nipponensis*	MGYIH*	1	
WP_329486261.1	*Kitasatospora sp.* NBC_01246	MSYNH*	2	Protease/Peptidase

WP_329561375.1	*Kitasatospora sp.* NBC_01266	MSYDH*	1	Methyltransferase, PGM_ligase
WP_063857100.1	*Kitasatospora sp.* Root107	MRYDY*	1	Methyltransferase, Protease/Peptidase
WP_051817038.1	*Kitasatospora sp.* YST-16	MSYDH*	1	PGM_ligase
WP_221339774.1	*Kutzneria kofuensis*	MSYDH*	1	Oxidoreductase
WP_330854646.1	*Lentzea sp.*	MSYDH*	1	Protease/Peptidase
WP_089955640.1	*Lentzea xinjiangensis*	MRYLH*	1	Hydroxylase, Methyltransferase
SKB57425.1	*Lysinibacillus sp.* AC-3	MQWNY*	3	
WP_197142288.1	*Lysinibacillus sphaericus*	MQWNY*	3	
TQR30875.1	*Lysinibacillus sphaericus*	MQWNY*	4	
WP_323657460.1	*Lysinibacillus xylanilyticus*	MQWNY*	6	
GDX38889.1	*Methylocystaceae bacterium*	MKYLY*	1	
WP_166795220.1	*Micrococcus yunnanensis*	MTYDHIL*	1	PGM_ligase, additional_P450
RQX01605.1	*Micromonospora arida*	MSYDH*	1	
WP_208601958.1	*Micromonospora eburnea*	MRYIH*	1	Protease/Peptidase
WP_208812380.1	*Micromonospora echinofusca*	MRYLH*	1	Oxidoreductase, Acetyltransferase
WP_145776703.1	*Micromonospora olivasterospora*	VRYLH*	1	Methyltransferase, Protease/Peptidase
WP_184536939.1	*Micromonospora polyrhachis*	MRHYY*	2	Aminotransferase, Methyltransferase, Hydroxylase, Protease/Peptidase
WP_128137096.1	*Micromonospora provocatoris*	MRYAY*	1	Hydroxylase, Protease/Peptidase
WP_201752802.1	*Micromonospora rubida*	MRYLH*	1	Protease/Peptidase
WP_216932370.1	*Micromonospora sp.* b486	MRYAY*	1	Hydroxylase, Acetyltransferase
WP_165878048.1	*Micromonospora sp.* MW-13	MRYWH*	1	Hydroxylase, Methyltransferase
WP_207892810.1	*Micromonospora sp.* MW-13	MRYLH*	1	Protease/Peptidase
WTY81129.1	*Micromonospora sp.* NBC_01405	MRYLH*	1	Protease/Peptidase

WTY49331.1	*Micromonospora sp.* NBC_01412	MRYLH*	1	Hydroxylase, Protease/Peptidase, Sulfotransferase
WP_128137097.1	*Micromonospora tulbaghiae*	MRYAY*	1	Hydroxylase, Methyltransferase, Protease/Peptidase
WP_325687722.1	*Mycobacterium sp.*	MFYWH*	1	
WP_101951728.1	*Mycobacterium sp.* 3519A	MFYWH*	1	
WP_085304054.1	*Mycolicibacillus koreensis*	MFYWH*	1	
WP_163895064.1	*Mycolicibacterium hippocampi*	MFYWH*	1	
WP_195096709.1	*Nocardia blacklockiae*	MSYDHVAV *	1	Oxidoreductase
WP_203236935.1	*Nocardia panacis*	MDYPH*	1	Aminotransferase, Acetyltransferase, Protease/Peptidase
WP_225731383.1	*Nocardia sp.* JCM 34519	MRHEY*	1	Methyltransferase
WTL55286.1	*Nocardia sp.* NBC_01499	MMYWH*	1	Methyltransferase
WP_017622485.1	*Nocardiopsis chromatogenes* YIM 90109	MRYIH*	1	Hydroxylase
WP_192775534.1	*Nonomuraea africana*	VRYLH*	1	Oxidoreductase
WP_138720392.1	*Nonomuraea basaltis*	MRYLH*	1	NO_synthase, Methyltransferase, Protease/Peptidase
WP_195896979.1	*Nonomuraea cypriaca*	MRYLH*	1	NO_synthase, Methyltransferase, Protease/Peptidase
GAA3599678.1	*Nonomuraea rosea*	MRYWY*	1	Methyltransferase, Sulfotransferase, Oxidoreductase
WP_103962916.1	*Nonomuraea solani*	MRYLH*	1	NO_synthase, Methyltransferase, Protease/Peptidase, Oxidoreductase
WP_103964457.1	*Nonomuraea solani*	MRYRH*	1	Aminotransferase, Hydroxylase
QLG40373.1	*Paenibacillus sp.* E222	MIYTH*	3	PGM_ligase
WP_203790048.1	*Paractinoplanes rishiriensis*	MSYDH*	1	

WP_197938166.1	*Phytohabitans flavus*	MRYIH*	1	Hydroxylase, Methyltransferase, Acetyltransferase, Protease/Peptidase
BCB75145.1	*Phytohabitans flavus*	MRYIH*	1	Methyltransferase, Acetyltransferase, Protease/Peptidase
WP_196450865.1	*Planomonospora sp.* ID82291	MRYYH*	1	Oxidoreductase, Protease/Peptidase
WP_136921689.1	*Polyangium aurulentum*	MSYDH*	2	
WP_212826894.1	*Polymorphospora rubra*	MRYLH*	1	Oxidoreductase
WP_163988023.1	*Pyxidicoccus caerfyrddinensis*	MNYLH*	1	Methyltransferase
WP_169344400.1	*Pyxidicoccus fallax*	MNYLH*	1	Methyltransferase, Protease/Peptidase
WP_095343844.1	*Rothia dentocariosa*	MQYAH*	1	Oxidoreductase, additional_P450
WP_070535711.1	*Rothia sp. HMSC058E10*	MQYAH*	1	Oxidoreductase, additional_P450
WP_203920145.1	*Rugosimonospora africana*	MKYWH*	1	Hydroxylase, Methyltransferase
WP_211898415.1	*Saccharopolyspora erythraea*	MDYPH*	1	Protease/Peptidase
EQD85547.1	*Saccharopolyspora erythraea* D	MDYPH*	1	
WP_168587632.1	*Saccharopolyspora sp.* ASAGF58	MVYDH*	1	Hydroxylase, PGM_ligase, Acetyltransferase
GGI73495.1	*Saccharopolyspora subtropica*	MSYIH*	1	
WP_121006811.1	*Saccharothrix australiensis*	MRYLH*	1	
WP_015100824.1	*Saccharothrix espanaensis* DSM 44229	MDYPH*	1	Methyltransferase, Protease/Peptidase
GAA3463537.1	*Saccharothrix longispora*	MDYPH*	1	
WP_219441052.1	*Saccharothrix obliqua*	MDYPH*	1	Methyltransferase, Protease/Peptidase
WP_201435482.1	*Saccharothrix sp.* 6-C	MYYVH*	1	Oxidoreductase, Protease/Peptidase
WP_143531888.1	*Saccharothrix sp.* ALI-22-I	MFYDH*	1	Acetyltransferase, PGM_ligase
WP_073897089.1	*Saccharothrix sp.* CB00851	MRHLH*	1	Protease/Peptidase

WP_053716375.1	*Saccharothrix sp.* NRRL B-16348	MYYLH*	1	Oxidoreductase, Acetyltransferase, Protease/Peptidase
WP_033431254.1	*Saccharothrix syringae*	MDYPH*	1	Protease/Peptidase
WP_211348027.1	*Saccharothrix texasensis*	MYYVH*	1	Oxidoreductase, Protease/Peptidase
RKT69193.1	*Saccharothrix variisporea*	MRYVH*	1	Hydroxylase, Methyltransferase
WP_061608438.1	*Sorangium cellulosum*	MKYCW*	1	Hydroxylase, Oxidoreductase
WP_204011461.1	*Sphaerimonospora thailandensis*	MRYLH*	1	Hydroxylase, Methyltransferase
WP_176497114.1	*Sphingomonas sp.* HMP6	MVHPH*	1	Oxidoreductase
WUU87918.1	*Spirillospora sp.* NBC_01491	MRYWY*	1	Methyltransferase
WP_147135745.1	*Stackebrandtia albiflava*	MRYWY*	1	Hydroxylase, Methyltransferase, Acetyltransferase, Sulfotransferase
WP_013017934.1	*Stackebrandtia nassauensis* DSM 44728	MRYWY*	1	Hydroxylase, Methyltransferase, Glycosyltransferase, Sulfotransferase
WP_196194857.1	*Streptacidiphilus fuscans*	MRYVH*	1	Methyltransferase, Protease/Peptidase
WP_052809996.1	*Streptomonospora alba*	MRYDH*	1	Hydroxylase, Methyltransferase, PGM_ligase, additional_P450
WTQ78865.1	*Streptomyces achromogenes*	MSYDH*	1	Hydroxylase, PGM_ligase
WWM30798.1	*Streptomyces acidiscabies*	MSYDH*	1	Hydroxylase, Methyltransferase, PGM_ligase
GHC29544.1	*Streptomyces albogriseolus*	MRHIH*	1	
TGG76237.1	*Streptomyces albus*	MRYVH*	1	
WP_055499314.1	*Streptomyces albus*	MRYVH*	1	Protease/Peptidase
WP_164439288.1	*Streptomyces albus*	MRYVH*	1	Protease/Peptidase
WP_143648821.1	*Streptomyces alkaliterrae*	MSYDH*	1	Hydroxylase, PGM_ligase
BBA20962.1	*Streptomyces atratus*	MRYLH*	1	Aminotransferase, NO_synthase, additional_P450

AXE76435.1	*Streptomyces atratus*	MRYLH*	1	Aminotransferase, NO_synthase, additional_P450
WP_159041763.1	*Streptomyces aureus*	MKYWH*	1	Methyltransferase, Protease/Peptidase
WP_107082903.1	*Streptomyces avermitilis*	MRYVH*	1	Hydroxylase, Methyltransferase, Acetyltransferase
BAU77552.1	*Streptomyces avermitilis* MA-4680	MRYVH*	1	Hydroxylase, Methyltransferase
WP_061920834.1	*Streptomyces bungoensis*	MRYAY*	1	Hydroxylase, Protease/Peptidase
WP_030819383.1	*Streptomyces caelestis*	MSYDH*	1	Hydroxylase, PGM_ligase
WP_191207733.1	*Streptomyces chumphonensis*	MRYLH*	2	Hydroxylase, Methyltransferase
AXU14123.1	*Streptomyces clavuligerus*	MRHEY*	1	Methyltransferase
GAA4934895.1	*Streptomyces coeruleoprunus*	MKYWH*	1	
WP_114025389.1	*Streptomyces diacarni*	MRYVH*	1	Protease/Peptidase
WP_191867971.1	*Streptomyces diastaticus*	MRYLH*	2	Hydroxylase, Methyltransferase
WP_190056927.1	*Streptomyces echinoruber*	MSYDH*	1	Hydroxylase, PGM_ligase
WP_024761848.1	*Streptomyces exfoliatus* DSM 41693	MKYWH*	1	Protease/Peptidase
WP_190042383.1	*Streptomyces filamentosus*	MSYDH*	1	Hydroxylase, PGM_ligase
WP_051819776.1	*Streptomyces flavochromogenes*	MKYWH*	1	Methyltransferase, Protease/Peptidase
QPK49168.1	*Streptomyces gardneri*	MKYWH*	1	Protease/Peptidase
WP_167534015.1	*Streptomyces gardneri*	MKYWH*	1	Protease/Peptidase
ARF53562.1	*Streptomyces gilvosporeus*	MRYIH*	1	Hydroxylase, Methyltransferase, Protease/Peptidase
WP_190144208.1	*Streptomyces glebosus*	MRYAY*	1	
WP_158718955.1	*Streptomyces globisporus*	MKYWH*	1	Methyltransferase, additional_P450
GGU84509.1	*Streptomyces gougerotii*	MRYLH*	2	
WP_086758420.1	*Streptomyces griseiscabiei*	MRYLH*	2	Hydroxylase, Methyltransferase

WP_051865888.1	*Streptomyces griseus*	MRHAH*	2	Aminotransferase, Oxidoreductase, Methyltransferase
TQE28776.1	*Streptomyces ipomoeae*	MRYLH*	2	
TQE17623.1	*Streptomyces ipomoeae*	MRYLH*	2	
WP_153526325.1	*Streptomyces jumonjinensis*	MSYDH*	1	Hydroxylase, Methyltransferase, PGM_ligase
WP_189599827.1	*Streptomyces lateritius*	MKYWH*	1	Methyltransferase, Acetyltransferase
WP_158071316.1	*Streptomyces lateritius*	MKYWH*	1	Methyltransferase, Protease/Peptidase
WP_190158429.1	*Streptomyces litmocidini*	MKYWH*	1	Methyltransferase, additional_P450
GAA2712158.1	*Streptomyces luteosporeus*	MRYLH*	1	
WP_042158038.1	*Streptomyces lydicamycinicus*	MRYAY*	1	
WP_069570164.1	*Streptomyces lydicus*	MRHRW*	1	
WP_159674842.1	*Streptomyces mexicanus*	MGYDH*	1	Hydroxylase, PGM_ligase
WUS51385.1	*Streptomyces mirabilis*	MSYDH*	1	
WP_189512238.1	*Streptomyces narbonensis*	MKYWH*	1	Protease/Peptidase
WP_181924436.1	*Streptomyces nymphaeiformis*	MKYWH*	1	Methyltransferase, Protease/Peptidase
WP_209238816.1	*Streptomyces oryzae*	MRYFY*	1	
WP_135337917.1	*Streptomyces palmae*	MRYLH*	3	Protease/Peptidase
OSY48349.1	*Streptomyces platensis*	MRYAY*	1	
WUK00188.1	*Streptomyces platensis*	MRYAY*	1	
QEV56021.1	*Streptomyces platensis*	MRYAY*	1	
WP_074999637.1	*Streptomyces qinglanensis*	MRYIH*	1	Oxidoreductase, Protease/Peptidase
WP_114013457.1	*Streptomyces reniochalinae*	MRYIH*	1	Methyltransferase, Protease/Peptidase
WP_048478122.1	*Streptomyces roseus*	MSYDH*	1	Hydroxylase, PGM_ligase
WP_193453809.1	*Streptomyces rutgersensis*	MRYLH*	2	Hydroxylase, Methyltransferase

WP_198550307.1	*Streptomyces silvensis*	MSYDH*	1	Hydroxylase, PGM_ligase
WP_129248903.1	*Streptomyces sioyaensis*	MRYAY*	1	
WP_203525348.1	*Streptomyces sioyaensis*	MRYAY*	1	
WP_143627967.1	*Streptomyces sp.* 1-11	MRYAY*	1	Hydroxylase, Protease/Peptidase
WP_167744107.1	*Streptomyces sp.* 2BBP-J2	MRHIH*	1	Protease/Peptidase
RKT08294.1	*Streptomyces sp.* 3211.6	MSYDH*	1	
WP_216724382.1	*Streptomyces sp.* A108	MRYLH*	1	Aminotransferase, NO_synthase, additional_P450
WP_136214634.1	*Streptomyces sp.* A1136	MSYDH*	1	Hydroxylase, PGM_ligase
WP_019357243.1	*Streptomyces sp.* AA1529	MRYIH*	1	Protease/Peptidase
WP_111332071.1	*Streptomyces sp.* AC1-42T	MSYDH*	1	Hydroxylase, Glycosyltransferase, PGM_ligase
WP_217245349.1	*Streptomyces sp.* AC602_WCS936	MRHAH*	1	Protease/Peptidase
WP_148006904.1	*Streptomyces sp.* adm13	MKYWH*	1	Protease/Peptidase, additional_P450
RPF29694.1	*Streptomyces sp.* Ag109_G2-6	MSYDH*	1	
WP_221907285.1	*Streptomyces sp.* BHT-5-2	MRHVH*	1	
WP_143204839.1	*Streptomyces sp.* CB02009	MKYWH*	1	
WP_187621367.1	*Streptomyces sp.* CB02980	MKYWH*	1	Methyltransferase, Protease/Peptidase, additional_P450
WP_188273567.1	*Streptomyces sp.* CBMA152	MRHVH*	2	
WP_058928901.1	*Streptomyces sp.* CdTB01	MTYDH*	1	Hydroxylase, Methyltransferase, PGM_ligase
WP_047017281.1	*Streptomyces sp.* CNQ-509	MSYDH*	1	Hydroxylase, Methyltransferase, PGM_ligase
SCD88565.1	*Streptomyces sp.* di50b	MRHAH*	1	
PLW72023.1	*Streptomyces sp.* DJ	MSYDH*	1	

QIS75529.1	*Streptomyces sp.* DSM 40868	MSYDH*	1	Methyltransferase, PGM_ligase
WP_121715848.1	*Streptomyces sp.* E5N91	MRHAH*	1	
WP_227728090.1	*Streptomyces sp.* ET3-23	MTYYY*	1	
WP_093764026.1	*Streptomyces sp.* F-7	MRHIH*	1	Protease/Peptidase
WP_026246867.1	*Streptomyces sp.* HmicA12	MTYDH*	1	PGM_ligase
TXC99749.1	*Streptomyces sp.* ISID311	MRHAH*	1	
WP_215071590.1	*Streptomyces sp.* ISL-36	MKYWH*	1	additional_P450
WP_215017310.1	*Streptomyces sp.* ISL-94	MRYDHCL*	1	Methyltransferase, PGM_ligase
WP_093682451.1	*Streptomyces sp.* LamerLS-31b	MSYDH*	1	Hydroxylase, Methyltransferase, PGM_ligase
WP_158685075.1	*Streptomyces sp.* LaPpAH-108	MRYAY*	1	Oxidoreductase
WP_081222223.1	*Streptomyces sp.* M41(2017)	MTYDH*	1	Hydroxylase, Methyltransferase, PGM_ligase
WP_200723493.1	*Streptomyces sp.* MBT49	MSYDH*	1	Hydroxylase, Methyltransferase, PGM_ligase, Acetyltransferase
WP_172388015.1	*Streptomyces sp.* MNP-20	MSYDH*	1	Hydroxylase, Methyltransferase, PGM_ligase
WTR14400.1	*Streptomyces sp.* NBC_00138	MSYDH*	1	Hydroxylase, PGM_ligase
WTR07800.1	*Streptomyces sp.* NBC_00144	MRHAH*	1	Methyltransferase
WP_328941248.1	*Streptomyces sp.* NBC_00250	MKYWH*	1	Methyltransferase, Protease/Peptidase
WP_328330918.1	*Streptomyces sp.* NBC_00455	MRHAH*	1	Methyltransferase
WUG07218.1	*Streptomyces sp.* NBC_00466	MRHAH*	1	Methyltransferase
WUD46218.1	*Streptomyces sp.* NBC_00513	MSYDH*	1	Hydroxylase, PGM_ligase
WTX68379.1	*Streptomyces sp.* NBC_00647	MTYDH*	1	Hydroxylase, Methyltransferase, PGM_ligase

WP_329285285.1	*Streptomyces sp.* NBC_00691	MKYWH*	1	Methyltransferase, Protease/Peptidase
WTJ09092.1	*Streptomyces sp.* NBC_00715	MTYDH*	1	Hydroxylase, Methyltransferase, PGM_ligase
WSX67477.1	*Streptomyces sp.* NBC_00932	MRHAH*	1	Methyltransferase
WSV42528.1	*Streptomyces sp.* NBC_01077	MKYWH*	1	Methyltransferase, Protease/Peptidase
WSU39749.1	*Streptomyces sp.* NBC_01089	MSYDH*	1	
WSS51411.1	*Streptomyces sp.* NBC_01180	MRHAH*	1	Methyltransferase
WP_327393381.1	*Streptomyces sp.* NBC_01186	MRYIH*	1	Hydroxylase, Oxidoreductase, Protease/Peptidase
WSS45373.1	*Streptomyces sp.* NBC_01187	MRYIH*	1	Hydroxylase, Oxidoreductase, Protease/Peptidase
WP_331720860.1	*Streptomyces sp.* NBC_01241	MSYDH*	1	Hydroxylase, PGM_ligase
WP_329619874.1	*Streptomyces sp.* NBC_01255	MKYWH*	1	Methyltransferase, Protease/Peptidase
WTY58779.1	*Streptomyces sp.* NBC_01411	MRHAH*	1	Methyltransferase
WSD37542.1	*Streptomyces sp.* NBC_01750	MRYVH*	1	Hydroxylase, Methyltransferase
WP_326806865.1	*Streptomyces sp.* NBC_01775	MRYIH*	1	Hydroxylase, Oxidoreductase, Protease/Peptidase
WSB05146.1	*Streptomyces sp.* NBC_01794	MRYVH*	1	Hydroxylase, Methyltransferase
WP_326686421.1	*Streptomyces sp.* NBC_01795	MRYIH*	1	Hydroxylase, Oxidoreductase, Protease/Peptidase
WP_225799993.1	*Streptomyces sp.* NK15101	MKYWH*	1	Methyltransferase, Protease/Peptidase, additional_P450
WP_209497897.1	*Streptomyces sp.* NPDC090301	MKYWH*	1	Methyltransferase, Protease/Peptidase
WP_143664724.1	*Streptomyces sp.* NRRL B-24572	MKYWH*	1	Methyltransferase
WP_030800649.1	*Streptomyces sp.* NRRL S-337	MSYDH*	1	Oxidoreductase

WP_185094699.1	*Streptomyces sp.* PanSC19	MKYWH*	1	Methyltransferase, Protease/Peptidase, additional_P450
WP_158992425.1	*Streptomyces sp.* QHH-9511	MKYWH*	1	Methyltransferase, Acetyltransferase
WP_056562044.1	*Streptomyces sp.* Root66D1	MKYWH*	1	Methyltransferase, Protease/Peptidase, additional_P450
WP_168482770.1	*Streptomyces sp.* RPA4-5	MKYWH*	1	
WP_164657610.1	*Streptomyces sp.* S1A1-7	MKYWH*	1	Hydroxylase, Methyltransferase, PGM_ligase
WP_164405930.1	*Streptomyces sp.* S1D4-23	MKYWH*	1	Hydroxylase, Methyltransferase, PGM_ligase
WP_329853522.1	*Streptomyces sp.* SP18ES09	MKYWH*	1	Methyltransferase, Protease/Peptidase
WP_162689260.1	*Streptomyces sp.* ST1020	MSYDH*	1	Hydroxylase, Methyltransferase, PGM_ligase
WP_055718270.1	*Streptomyces sp.* ST1020	MSYDH*	1	Hydroxylase, Methyltransferase, PGM_ligase
PZT70810.1	*Streptomyces sp.* SW4	MRHAH*	1	
WP_143060225.1	*Streptomyces sp.* TLI_105	MKYWH*	1	Methyltransferase, Protease/Peptidase, additional_P450
WP_129279950.1	*Streptomyces sp.* TM32	MRYAY*	1	
WP_187821553.1	*Streptomyces sp.* TRM68367	MTYDH*	1	Hydroxylase, Oxidoreductase, PGM_ligase
WP_125523660.1	*Streptomyces sp.* WAC 05379	MSYDH*	1	
WP_125741628.1	*Streptomyces sp.* WAC01280	MKYWH*	1	Protease/Peptidase
WP_143098833.1	*Streptomyces sp.* yr375	MSYDH*	1	Hydroxylase, Methyltransferase, PGM_ligase
OEJ36126.1	*Streptomyces subrutilus*	MSYDH*	1	
WP_079154530.1	*Streptomyces subrutilus*	MSYDH*	1	Hydroxylase, PGM_ligase
WP_189804110.1	*Streptomyces tanashiensis*	MKYWH*	1	

WP_190106205.1	*Streptomyces tanashiensis*	MKYWH*	1	Methyltransferase, Protease/Peptidase, additional_P450
QES18500.1	*Streptomyces venezuelae*	MKYWH*	1	Protease/Peptidase
WP_150162815.1	*Streptomyces venezuelae*	MKYWH*	1	Methyltransferase, Protease/Peptidase, additional_P450
QES09683.1	*Streptomyces venezuelae*	MKYWH*	1	Methyltransferase, Protease/Peptidase
WP_145953730.1	*Streptomyces venezuelae* ATCC 10712	MKYWH*	1	Methyltransferase, Protease/Peptidase, additional_P450
WP_015037861.1	*Streptomyces venezuelae* ATCC 10712	MKYWH*	1	Methyltransferase, Protease/Peptidase, additional_P450
WP_175439194.1	*Streptomyces vilmorinianum*	MKYWH*	1	Methyltransferase
WP_188342106.1	*Streptomyces xanthii*	MKYWH*	1	Hydroxylase, PGM_ligase
WP_328489878.1	*Streptomyces zaomyceticus*	MKYWH*	1	Methyltransferase, Protease/Peptidase
WP_327165416.1	*Streptomyces zaomyceticus*	MKYWH*	1	Methyltransferase, Protease/Peptidase
WTR68839.1	*Streptomyces zaomyceticus*	MKYWH*	1	Methyltransferase, Protease/Peptidase
WTT42013.1	*Streptomyces zaomyceticus*	MKYWH*	1	Methyltransferase, Protease/Peptidase
WP_157995908.1	*Thermomonospora amylolytica*	MRYIH*	1	Hydroxylase, Glycosyltransferase, Protease/Peptidase, Sulfotransferase
WP_182708337.1	*Thermomonospora cellulosilytica*	MRYIH*	1	Hydroxylase, Glycosyltransferase, Protease/Peptidase, Sulfotransferase

2. Structures of peptides

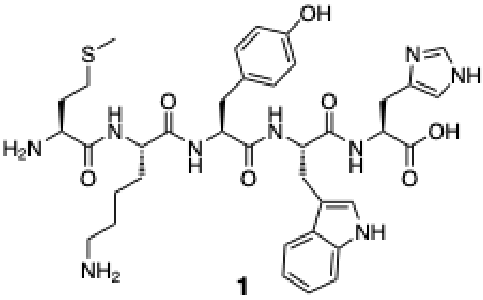

1

Exact Mass: 763.34757

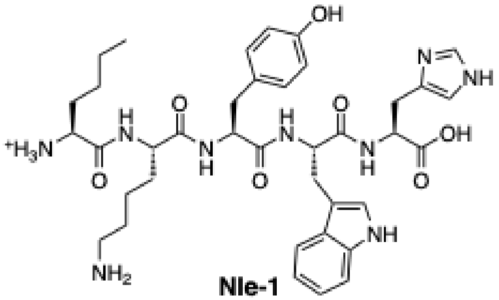

Nle-1

Exact Mass: 746.39842

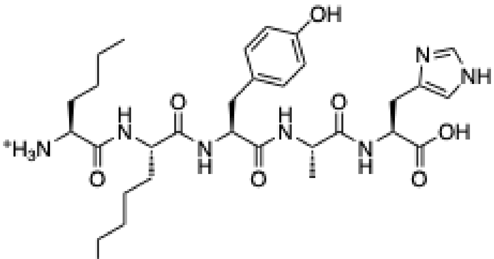

Nle-1-(Ala4)

Exact Mass: 631.35622

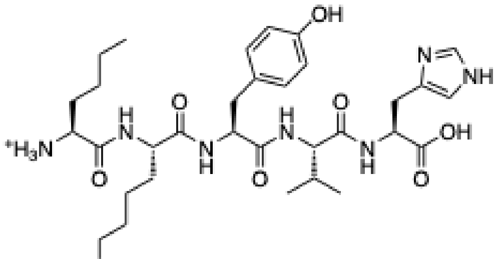

Nle-1-(Val4)

Exact Mass: 659.38752

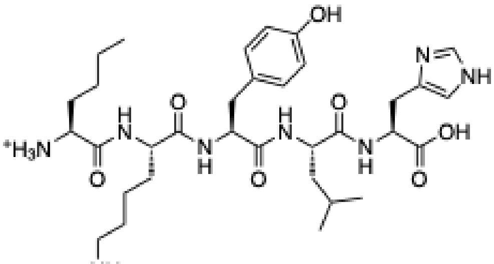

Nle-1-(Leu4)

Exact Mass: 673.40317

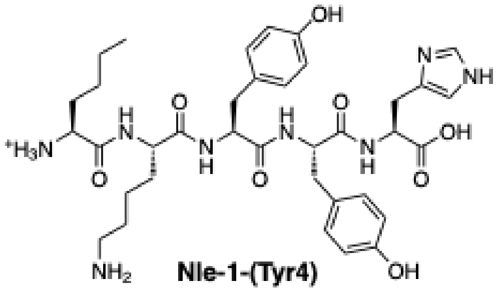

Nle-1-(Tyr4)

Exact Mass: 723.38244

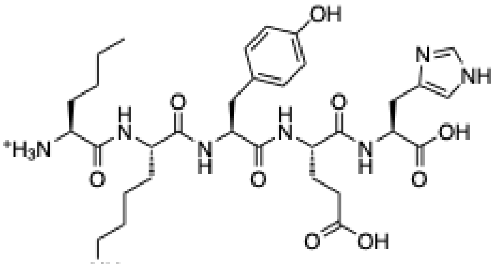

Nle-1-(Glu4)

Exact Mass: 689.36170

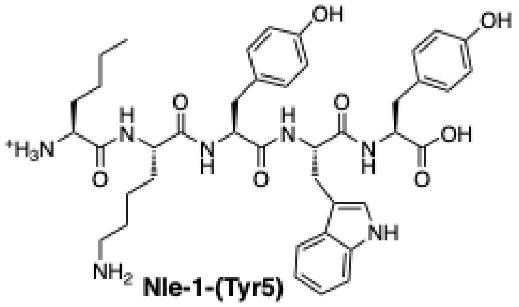

Nle-1-(Tyr5)

Exact Mass: 772.40284

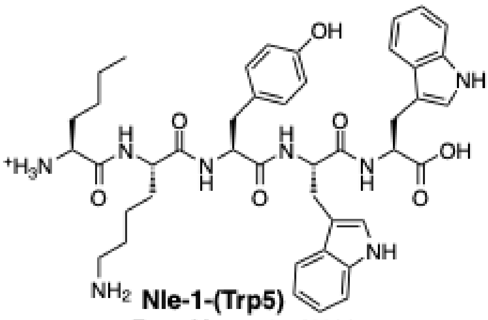

Nle-1-(Trp5)

Exact Mass: 795.41882

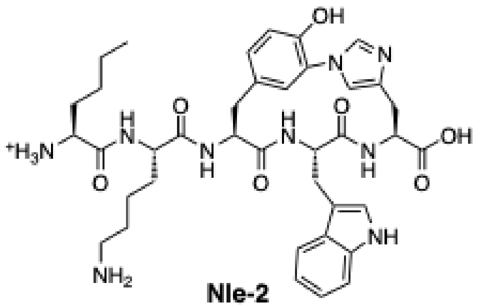

Nle-2

Exact Mass: 744.38277

Figure S3. Structures of peptides **1**, **Nle-1** and **Nle-2**.

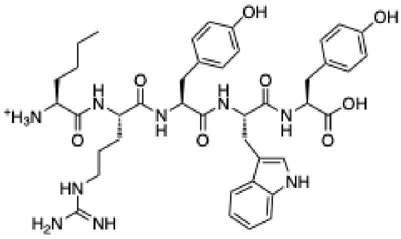

Nle-3-(Trp4)
Exact Mass: 800.40899

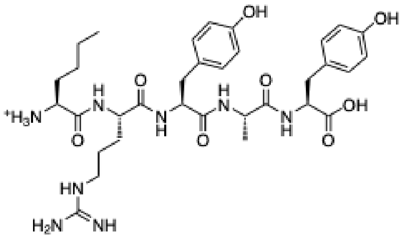

Nle-3-(Ala4)
Exact Mass: 685.36679

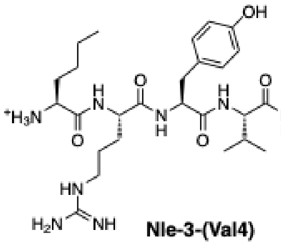

Nle-3-(Val4)
Exact Mass: 713.39809

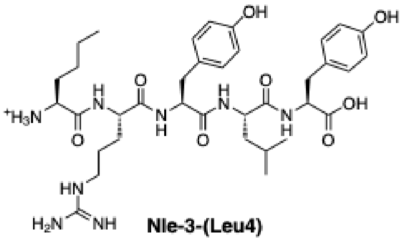

Nle-3-(Leu4)
Exact Mass: 727.41374

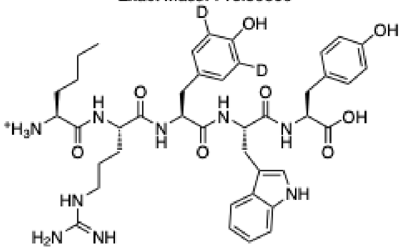

Nle-3-(3,5-d$_2$-Tyr3, Trp4)
Exact Mass: 802.42154

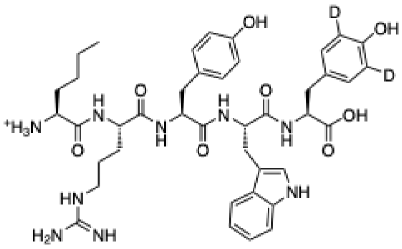

Nle-3-(Trp4, 3,5-d$_2$-Tyr5)
Exact Mass: 802.42154

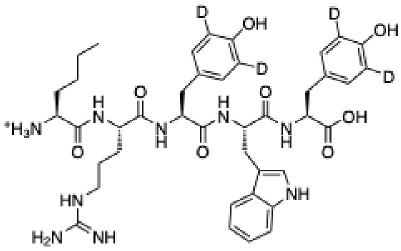

Nle-3-(Trp4, 3,5-d$_2$-Tyr3,5)
Exact Mass: 804.43409

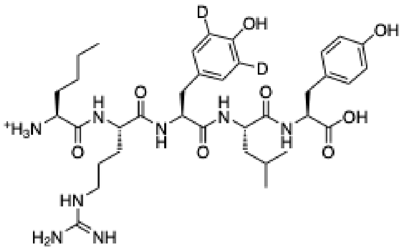

Nle-3-(3,5-d$_2$-Tyr3, Leu4)
Exact Mass: 729.42629

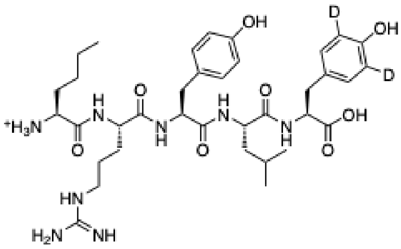

Nle-3-(Leu4, 3,5-d$_2$-Tyr5)
Exact Mass: 729.42629

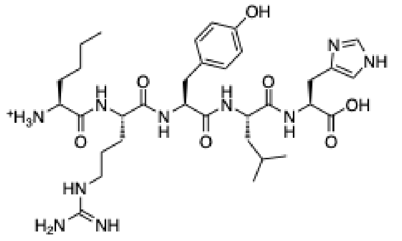

Nle-3-(Leu4, His5)
Exact Mass: 701.40932

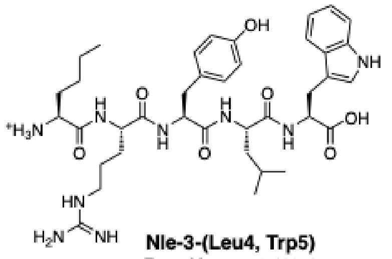

Nle-3-(Leu4, Trp5)
Exact Mass: 750.42972

Figure S4. Structures of peptides **Nle-3**.

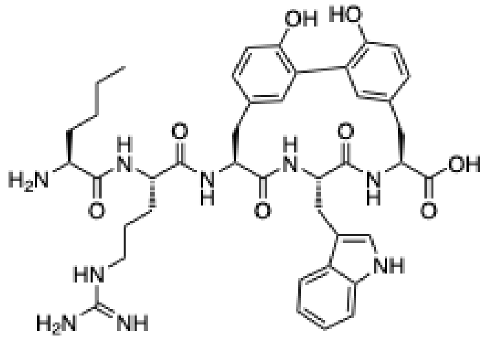

Nle-4-(Trp4)

Exact Mass: 797.3861

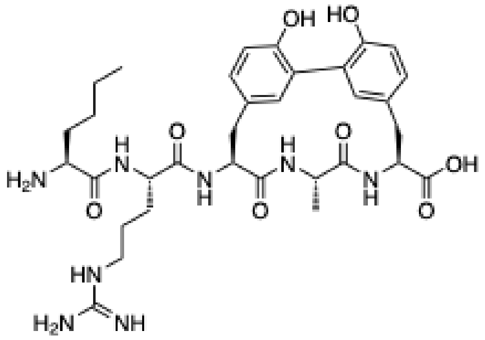

Nle-4-(Ala4)

Exact Mass: 682.3439

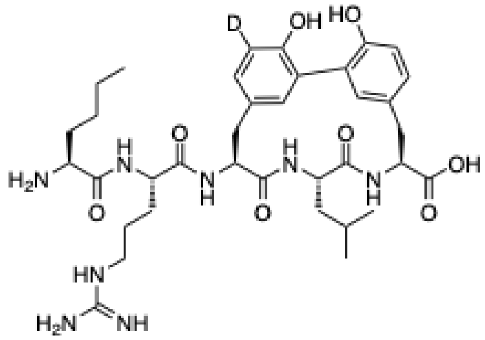

Nle-4-(3,5-d$_2$-Tyr3, Leu4)

Exact Mass: 725.3971

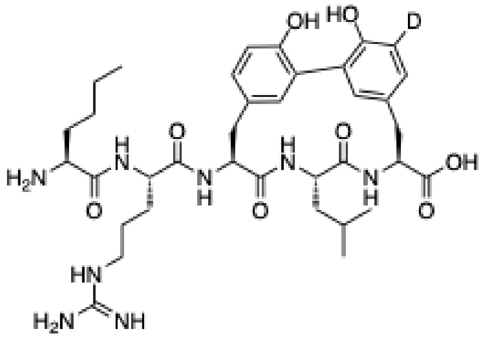

Nle-4-(Leu4, 3,5-d$_2$-Tyr5)

Exact Mass: 725.3971

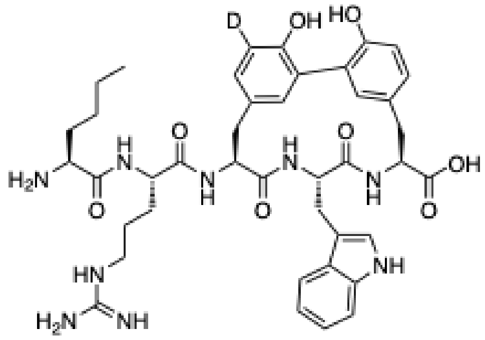

Nle-4-(3,5-d$_2$-Tyr3, Trp4)

Exact Mass: 798.3923

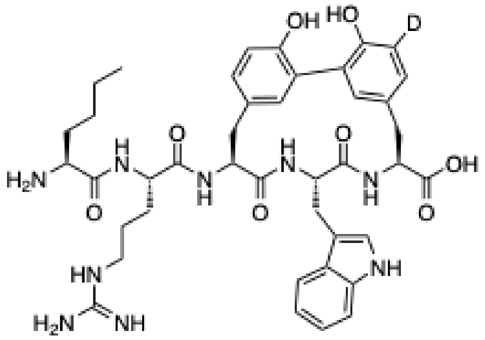

Nle-4-(Trp4, 3,5-d$_2$-Tyr5)

Exact Mass: 798.3923

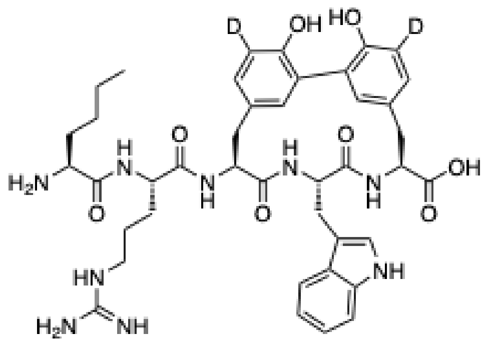

Nle-4-(Trp4, 3,5-d$_2$-Tyr3,5)

Exact Mass: 799.3986

Figure S5. Structures of peptides **Nle-4**.

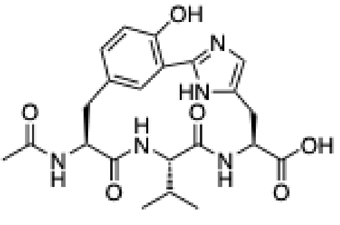

5

Exact Mass: 457.1961

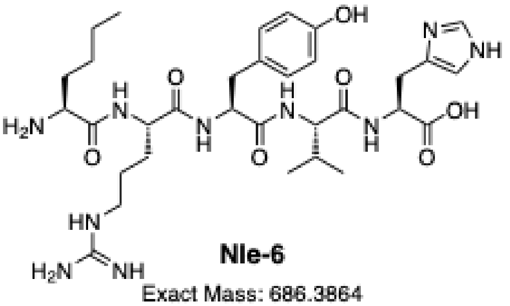

Nle-6

Exact Mass: 686.3864

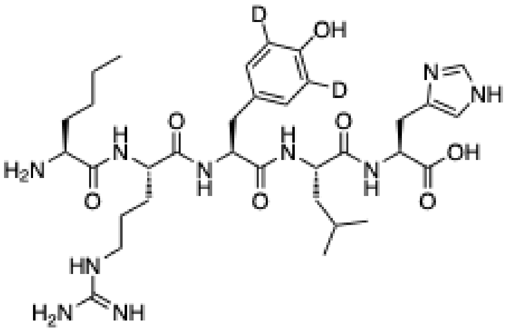

Nle-6-(3,5-d$_2$-Tyr3, Leu4)

Exact Mass: 702.4146

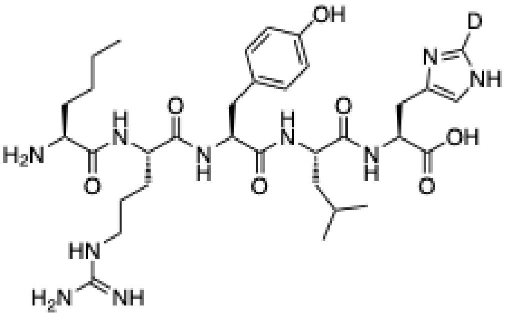

Nle-6-(Leu4, 2-d-His5)

Exact Mass: 701.4083

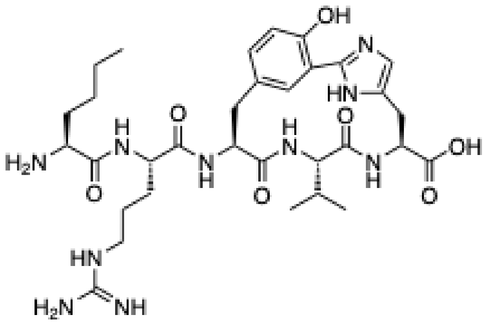

Nle-7/Nle-7-(Leu4, 2-d-His5)

Exact Mass: 684.3707

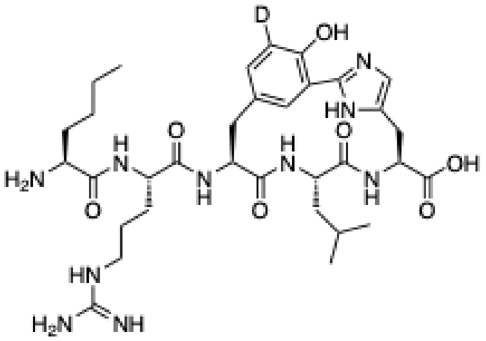

Nle-7-(3,5-d$_2$-Tyr3, Leu4)

Exact Mass: 699.3927

Figure S6. Structures of peptides **5**, **Nle-6** and **Nle-7**.

3. Structural characterisation of peptides

Nle-1. ^{1}H-NMR (600 MHz, DMSO-d_6): δ 10.86 (s, 1 H), 8.77 (s, 1 H), 8.51 (d, *J* = 8.2 Hz, 1 H), 8.46 (d, *J* = 7.9 Hz, 1 H), 8.16 (d, *J* = 7.7 Hz, 1 H), 7.99 (d, *J* = 8.1 Hz, 1 H), 7.89 (bs, 2 H), 7.57 (d, *J* = 8.0 Hz, 1 H), 7.32 (d, *J* = 8.1 Hz, 1 H), 7.27 (s, 1 H), 7.14 (d, *J* = 2.6 Hz, 1 H), 7.05 (td, *J* = 6.9 Hz, 1.3 Hz, 1 H), 6.97 (t, *J* = 7.8 Hz, 1 H), 6.93 (d, *J* = 8.6, 2 H), 6.56 (d, *J* = 8.6 Hz, 2 H), 4.51-4.59 (m, 2 H), 4.42-4.45 (m, 1 H), 4.26-4.29 (m, 1 H), 3.76 (t, *J* = 6.6 Hz, 1 H), 3.08-3.16 (m, 2 H), 2.94-3.03 (m, 2 H), 2.84-2.88 (m, 1 H), 2.71-2.75 (m, 2 H), 2.60 (q, *J* = 5.0 Hz, 1 H), 1.56-1.64 (m, 3 H), 1.47-1.51 (m, 2 H), 1.40-1.43 (m ,1 H), 1.21-1.22 (m, 5 H), 0.79 (t, *J* = 6.9 Hz, 3 H). ^{13}C{^{1}H}-NMR (150 MHz, DMSO-d_6): δ 172.0, 171.5, 171.2, 170.8, 168.5, 155.8, 136.1, 133.9, 130.1, 130.0, 127.6, 127.4, 123.7, 120.9, 120.2, 118.4, 118.3, 118.2, 117.0, 116.2, 114.8, 111.4, 109.8, 54.0, 53.6, 52.4, 52.2, 51.4, 38.7, 36.7, 35.9, 31.7, 31.0, 27.7, 26.7, 26.3, 22.2, 21.8, 13.7. HRMS (ESI) *m/z*: [M+H]$^+$ Calcd. for $C_{38}H_{52}N_9O_7^+$ 746.3984; Found 746.3986; error 0.3 ppm.

Nle-1-(Ala4). ^{1}H-NMR (600 MHz, DMSO-d_6): δ 8.69 (s, 1 H), 8.52 (d, *J* = 8.3 Hz, 1 H), 8.27 (d, *J* = 8.0 Hz, 1 H), 8.15 (d, *J* = 7.3 Hz, 1 H), 8.04 (d, *J* = 8.0 Hz, 1 H), 7.26 (s, 1 H), 6.99 (d, *J* = 8.6 Hz, 2 H), 6.60 (d, *J* = 8.5 Hz, 2 H), 4.50-4.53 (m, 1 H), 4.43-4.47 (m, 1 H), 4.23-4.31 (m, 2 H), 3.76 (t, *J* = 6.6 Hz, 1 H), 3.12 (dd, *J* = 15.5 Hz, 5.1 Hz, 1 H), 2.97-3.01 (m, 1 H), 2.90 (dd, *J* = 14.3 Hz, 4.1 Hz, 1 H), 2.74 (t, *J* = 7.8 Hz, 2 H), 2.64 (q, *J* = 4.7 Hz, 1 H), 1.58-1.65 (m, 3 H), 1.43-1.54 (m, 3 H), 1.21-1.32 (m, 6 H), 1.18-1.20 (m, 3 H), 0.81 (t, *J* = 6.8 Hz, 3 H). ^{13}C{^{1}H}-NMR (150 MHz, DMSO-d_6): δ 172.2, 172.0, 170.9, 168.8, 134.0, 130.0, 127.7, 120.2, 118.2, 117.0, 116.2, 114.8, 53.9, 52.4, 52.2, 51.4, 48.2, 38.7, 36.5, 31.7, 30.9, 26.8, 26.7, 26.2, 22.2, 21.8, 18.1, 13.7. HRMS (ESI) *m/z*: [M+H]$^+$ Calcd. for $C_{30}H_{47}N_8O_7^+$ 631.3562; Found 631.3563; error 0.2 ppm.

Nle-1-(Val4). ^{1}H-NMR (600 MHz, DMSO-d_6): δ 8.78 (s, 1 H), 8.53 (d, *J* = 8.3 Hz, 1 H), 8.40 (d, *J* = 7.8 Hz, 1 H), 8.09 (d, *J* = 8.1 Hz, 1 H), 7.90 (d, *J* = 8.7 Hz, 3 H), 7.30 (s, 1 H), 6.99 (d, *J* = 8.6 Hz, 2 H), 6.60 (d, *J* = 8.4 Hz, 2 H), 4.50-4.56 (m, 2 H), 4.29-4.33 (m, 1 H), 4.13-4.15 (m, 1 H), 3.76 (t, *J* = 6.5 Hz, 1 H), 3.13 (dd, *J* = 15.5 Hz, 5.5 Hz, 1 H), 2.97-3.01 (m, 1 H), 2.86 (dd, *J* = 14.2 Hz, 4.2 Hz, 1 H), 2.74 (t, *J* = 8.2 Hz, 2 H), 2.65 (q, *J* = 4.7 Hz, 1 H), 1.94 (s, *J* = 6.8 Hz, 1 H), 1.57-1.64 (m, 3 H), 1.46-1.53 (m, 3 H), 1.27-1.32 (m, 1 H), 1.22-1.25 (m, 5 H), 1.18-1.20 (m, 3 H), 0.81 (m, 9 H). ^{13}C{^{1}H}-NMR (150 MHz, DMSO-d_6): δ 172.0, 171.1, 170.9, 170.9, 168.5, 155.8, 133.9, 130.0, 127.7, 120.1, 118.2, 117.0, 116.2, 114.8, 57.6, 53.9, 52.3, 52.2, 51.2, 43.7, 38.7, 36.4, 31.9, 30.9, 30.7, 26.8, 26.3, 22.1, 21.8, 19.1, 18.0, 13.7. HRMS (ESI) *m/z*: [M+H]$^+$ Calcd. for $C_{32}H_{51}N_8O_7^+$ 659.3875; Found 659.3870; error 0.8 ppm.

Nle-1-(Leu4). ^{1}H-NMR (600 MHz, DMSO-d_6): δ 8.83 (s, 1 H), 8.52 (d, *J* = 8.3 Hz, 1 H), 8.30 (d, *J* = 8.0 Hz, 1 H), 8.11 (d, *J* = 8.2 Hz, 1 H), 8.06 (d, *J* = 8.2 Hz, 1 H), 7.31 (s, 1 H), 6.99 (d, *J* = 8.6 Hz, 2 H), 6.60 (d, *J* = 8.5 Hz, 2 H), 4.51-4.55 (m, 1 H), 4.45-4.48 (m, 1 H), 4.25-4.33 (m, 2 H), 4.13-4.15 (m, 1 H), 3.76 (t, *J* = 6.6 Hz, 1 H), 3.13 (dd, *J* = 15.3 Hz, 5.3 Hz, 1 H), 2.98-3.02 (m, 1 H), 2.87 (m, 1 H), 2.73 (m, 2 H), 2.65 (q, *J* = 4.7 Hz, 1 H), 1.94 (s, *J* = 6.8 Hz, 1 H), 1.38-1.65 (m, 9 H), 1.21-1.32 (m, 6 H), 0.83 (m, 9 H). ^{13}C{^{1}H}-NMR (150 MHz, DMSO-d_6): δ 172.0, 171.9, 171.1, 170.8, 168.4, 155.8, 133.9, 130.0, 127.8, 120.1, 118.2, 117.0, 116.2, 114.8, 53.8, 52.3, 52.1, 51.1, 51.1, 43.7, 40.8, 38.7, 36.4, 31.9, 30.9, 26.8, 26.2, 24.0, 23.0, 22.1, 21.7, 21.6 ,13.7. HRMS (ESI) *m/z*: [M+H]$^+$ Calcd. for $C_{33}H_{52}N_8O_7^+$ 673.4032; Found 673.4028; error 0.6 ppm.

Nle-1-(Tyr4). ^{1}H-NMR (600 MHz, DMSO-d_6): δ 8.79 (s, 1 H), 8.50 (d, *J* = 8.3 Hz, 1 H), 8.44 (d, *J* = 8.0 Hz, 1 H), 8.07 (d, *J* = 7.9 Hz, 1 H), 8.00 (d, *J* = 8.3 Hz, 1 H), 7.30 (s, 1 H), 7.01 (d, *J* = 8.6 Hz, 2 H), 6.94 (d, *J* = 8.8 Hz, 2 H), 6.63 (d, *J* = 8.5 Hz, 2 H), 6.58 (d, *J* = 8.5 Hz, 2 H), 4.53-4.57 (m, 1 H), 4.39-4.43 (m, 2 H), 4.26-4.30 (m, 1 H), 3.75 (t, *J* = 6.6 Hz, 1 H), 3.13 (dd, *J* = 15.4 Hz, 5.3 Hz, 1 H), 2.98-3.02 (m, 1 H), 2.83-2.88 (m, 2 H), 2.67-2.74 (m, 4 H), 2.57-2.61 (m, 1 H), 1.59-1.65 (m, 2 H), 1.55-1.58 (m, 1 H), 1.47-1.53 (m, 2 H), 1.40-1.46 (m, 1 H), 1.18-1.25 (m, 6 H), 0.80 (t, *J* = 6.9 Hz, 3 H). ^{13}C{^{1}H}-NMR (150 MHz, DMSO-d_6): δ 172.0, 171.2, 171.1, 170.8, 168.5, 155.9, 133.9, 130.1. 130.0, 129.8, 127.7, 127.5, 120.2, 118.2,

117.0, 116.2, 114.9, 114.8, 114.2, 54.2, 53.9, 52.4, 52.2, 51.3, 43.7, 38.7, 36.7, 31.8, 30.9, 26.7, 26.2, 22.2, 21.7, 13.7.HRMS (ESI) *m/z*: $[M+H]^+$ Calcd. for $C_{36}H_{51}N_8O_8^+$ 723.3824; Found 723.3820; error 0.6 ppm.

Nle-1-(Glu4). ^{1}H-NMR (600 MHz, DMSO-d_6): δ 8.51 (d, *J* = 8.4 Hz, 1 H), 8.32 (d, *J* = 7.8 Hz, 1 H), 8.13 (d, *J* = 7.8 Hz, 1 H), 8.06 (d, *J* = 8.0 Hz, 1 H), 7.20 (s, 1 H), 6.99 (d, *J* = 8.6 Hz, 2 H), 6.60 (d, *J* = 8.6 Hz, 2 H), 4.45-4.53 (m, 2 H), 4.24-4.33 (m, 2 H), 3.75 (t, *J* = 6.5 Hz, 1 H), 3.09 (dd, *J* = 15.2 Hz, 5.3 Hz, 1 H), 2.96-3.00 (m, 1 H), 2.87-2.90 (m, 1 H), 2.73-2.76 (m, 2 H), 2.64-2.68 (m, 1 H), 2.23 (t, *J* = 8.8 Hz, 2 H), 1.86-1.90 (m, 1 H), 1.72-1.76 (m, 1 H), 1.60-1.65 (m, 3 H), 1.44-1.54 (m, 3 H), 1.20-1.33 (m, 6 H), 0.81 (t, *J* = 6.9 Hz, 3 H). ^{13}C{^{1}H}-NMR (150 MHz, DMSO-d_6): δ 173.9, 172.1, 171.1, 171.0, 170.9, 168.4, 155.8, 134.1, 130.0, 127.6, 118.2, 116.2, 114.8, 53.9, 52.3, 52.1, 51.8, 51.5, 38.7, 36.5, 31.8, 30.9, 29.9, 27.5, 27.0, 26.7, 26.2, 22.1, 21.7, 13.7. HRMS (ESI) *m/z*: $[M+H]^+$ Calcd. for $C_{32}H_{49}N_8O_9^+$ 689.3617; Found 689.3621; error 0.5 ppm.

Nle-1-(Tyr5). ^{1}H-NMR (400 MHz, DMSO-d_6): δ 10.80 (s, 1 H), 9.22 (s, 1 H), 9.14 (s, 1 H), 8.45 (d, *J* = 8.2 Hz, 1 H), 8.06-8.15 (m, 5 H), 7.95 (d, *J* = 8.2 Hz, 1 H), 7.57 (d, *J* = 7.8 Hz, 1 H), 7.30 (d, *J* = 8.0 Hz, 1 H), 7.11 (d, *J* = 2.5 Hz, 1 H), 7.05 (t, *J* = 6.9 Hz, 1 H), 6.95-6.99 (m, 3 H), 6.93 (d, *J* = 8.5 Hz, 2 H), 6.64 (d, *J* = 8.5 Hz, 2 H), 6.57 (d, *J* = 8.5 Hz, 2 H), 4.56 (q, *J* = 5.3 Hz, 1 H), 4.36-4.45 (m, 2 H), 4.28 (q, *J* = 5.1 Hz, 1 H), 3.74 (bs, 1 H), 3.13 (dd, *J* = 14.8 Hz, 5.4 Hz, 1 H), 2.87-2.96 (m, 3 H), 2.79-2.85 (m, 2 H), 2.60-2.70 (m, 3 H), 1.56-1.65 (m, 3 H), 1.42-1.51 (m, 3 H), 1.22-1.25 (m, 6 H), 0.80 (t, *J* = 6.9 Hz, 3 H). ^{13}C{^{1}H}-NMR (100 MHz, DMSO-d_6): δ 172.7, 171.2, 170.9, 170.6, 168.4, 158.2, 157.9, 156.9, 155.7, 136.0, 130.1, 130.0, 127.6, 127.4, 127.2, 123.6, 120.8, 118.8, 118.2, 115.8, 115.0, 114.7, 111.3, 109.8, 53.8, 53.3, 52.3, 52.1, 36.7, 36.1, 31.7, 30.9, 27.9, 26.7, 26.2, 22.1, 21.7, 13.7. HRMS (ESI) *m/z*: $[M+H]^+$ Calcd. for $C_{41}H_{54}N_7O_8^+$ 772.4028; Found 772.4027; error 0.2 ppm.

Nle-1-(Trp5). ^{1}H-NMR (400 MHz, DMSO-d_6): δ 10.85 (d, *J* = 2.5 Hz, 1 H), 10.80 (d, *J* = 2.5 Hz, 1 H), 9.13 (bs, 1 H), 8.46 (d, *J* = 8.4 Hz, 1 H), 8.22 (d, *J* = 7.5 Hz, 1 H), 8.04-8.09 (m, 4 H), 7.97 (d, *J* = 8.1 Hz, 1 H), 7.72 (bs, 3 H), 7.58 (d, *J* = 7.8 Hz, 1 H), 7.52 (d, *J* = 7.8 Hz, 1 H), 7.32 (dd, *J* = 8.0 Hz, 2.8 Hz, 2 H), 7.12 (d, *J* = 2.6 Hz, 2 H), 7.05 (t, *J* = 6.9 Hz, 2 H), 6.93-6.99 (m, 4 H), 6.56 (d, *J* = 8.5 Hz, 2 H), 4.60 (q, 5.2 Hz, 1 H), 4.52 (q, *J* = 7.4 Hz, 1 H), 4.40-4.45 (m, 1 H), 4.28 (q, *J* = 5.2 Hz, 1 H), 3.74 (q, *J* = 5.6 Hz, 1 H), 3.05-3.19 (m, 3 H), 2.93-2.99 (m, 1 H), 2.83-2.88 (m, 1 H), 2.60-2.73 (m, 3 H), 1.57-1.65 (m, 3 H), 1.42-1.52 (m, 3 H), 1.21-1.23 (m, 6 H), 0.80 (t, *J* = 6.8 Hz, 3 H). ^{13}C{^{1}H}-NMR (100 MHz, DMSO-d_6): δ 173.0, 171.2, 170.8, 170.6, 168.4, 155.7, 136.1, 136.0, 130.0, 127.6, 127.4, 127.3, 123.6, 120.9, 118.4, 118.2, 114.7, 111.4, 109.5, 53.9, 53.3, 53.1, 52.1, 31.7, 30.9, 27.2, 26.7, 26.2, 22.2, 21.7, 13.7. HRMS (ESI) *m/z*: $[M+H]^+$ Calcd. for $C_{43}H_{55}N_8O_7^+$ 795.4188; Found 795.4184; error 0.5 ppm.

Nle-3-(Trp4). ^{1}H-NMR (600 MHz, DMSO-d_6): δ 10.87 (d, *J* = 2.5 Hz, 1 H), 9.15 (bs, 1 H), 9.07 (bs, 1 H), 8.64 (d, *J* = 8.3 Hz, 1 H), 8.07-8.09 (m, 2 H), 7.57 (d, *J* = 7.9 Hz, 1 H), 7.35 (d, *J* = 8.0 Hz, 1 H), 7.15 (d, *J* = 2.6 Hz, 1 H), 7.09 (t, *J* = 7.1 Hz, 1 H), 7.01 (t, *J* = 7.5 Hz, 1 H), 6.95 (d, *J* = 8.5 Hz, 2 H), 6.63 (d, *J* = 8.6 Hz, 2 H), 6.55 (bs, 2 H), 6.39 (d, *J* = 8.0 Hz, 2 H), 4.37 (bs, 1 H), 4.26-4.30 (m, 2 H), 4.14 (bs, 1 H), 3.71 (t, *J* = 6.6 Hz, 1 H), 3.17 (dd, *J* = 14.7 Hz, 4.3 Hz, 1 H), 3.00-3.07 (m, 3 H), 2.88-2.94 (m, 2 H), 1.74 (bs, 1 H), 1.59-1.63 (m, 2 H), 1.47-1.52 (m, 2 H), 1.40 (bs, 1 H), 1.19-1.25 (m, 4 H), 0.80 (t, *J* = 6.9 Hz, 3 H). ^{13}C{^{1}H}-NMR (150 MHz, DMSO-d_6): δ 170.7, 168.4, 157.0, 155.8, 155.7, 136.2, 130.4, 130.4, 129.7, 127.2, 123.7, 121.0, 118.3, 114.8, 111.4, 110.1, 52.1, 40.3, 31.0, 28.8, 28.1, 26.3, 21.7, 13.7. HRMS (ESI) *m/z*: $[M+H]^+$ Calcd. for $C_{41}H_{54}N_9O_8^+$ 800.4090; Found 800.4080; error 1.3 ppm.

Nle-3-(Ala4). ^{1}H-NMR (600 MHz, DMSO-d_6): δ 9.20 (s, 1 H), 9.15 (s, 1 H), 8.51 (d, *J* = 8.0 Hz, 1 H), 8.04-8.08 (m, 3 H), 7.93 (bs, 1 H), 6.98 (td, *J* = 8.4 Hz, 2.8 Hz, 4 H), 6.63 (d, *J* = 8.5 Hz, 2 H), 6.59 (d, *J* = 8.5 Hz, 2 H), 4.44-4.47 (m, 1 H), 4.32-4.36 (m, 1 H), 4.25-4.29 (m, 2 H), 3.74 (t, *J* = 5.5 Hz, 1 H), 3.06 (q, *J* = 6.6 Hz, 2 H), 2.92-2.95 (m, 1 H), 2.80-2.88 (m, 2 H), 2.65-2.69 (m, 1 H), 1.60-1.66 (m, 3 H), 1.43-1.49 (m, 3 H), 1.19-1.25 (m, 4 H), 1.18 (d, *J* = 7.1 Hz, 3 H), 0.82 (t, *J* = 6.9 Hz, 3 H). ^{13}C{^{1}H}-NMR (150 MHz,

DMSO-d_6): δ 170.5, 168.4, 155.9, 155.8, 130.1, 130.0, 114.9, 114.8, 53.8, 52.1, 40.5, 35.9, 31.0, 26.2, 24.8, 21.7, 18.4, 13.7. HRMS (ESI) *m/z*: $[M+H]^+$ Calcd. for $C_{33}H_{49}N_8O_8^+$ 685.3668; Found 685.3671; error 0.4 ppm.

Nle-3-(Val4). ^{1}H-NMR (600 MHz, DMSO-d_6): δ 8.70 (d, *J* = 8.3 Hz, 1 H), 8.13 (d, *J* = 8.2 Hz, 1 H), 8.04 (d, *J* = 7.6 Hz, 1 H), 7.96 (d, *J* = 8.9 Hz, 1 H), 6.99-7.02 (m, 4 H), 6.64 (d, *J* = 8.5 Hz, 2 H), 6.60 (d, *J* = 8.4 Hz, 2 H), 4.47 (q, *J* = 5.0 Hz, 1 H), 4.29-4.33 (m, 2 H), 4.15 (t, *J* = 6.9 Hz, 1 H), 3.80 (t, *J* = 6.4 Hz, 1 H), 3.44-3.54 (m, 1 H), 3.03 (m, 2 H), 2.79-2.93 (m, 4 H), 2.64-2.72 (m, 2 H), 1.54-1.66 (m, 3 H), 1.42-1.49 (m, 3 H), 1.20-1.23 (m, 4 H), 0.79-0.82 (m, 9 H). ^{13}C{^{1}H}-NMR (150 MHz, DMSO-d_6): δ 173.2, 171.0, 170.9, 170.7, 168.7, 157.1, 156.0, 155.9, 130.1, 130.1, 127.7, 127.6, 115.0, 114.9, 57.8, 54.3, 54.2, 53.5, 52.4, 52.2, 47.9, 37.7, 36.6, 31.7, 28.3, 26.3, 26.0, 23.4, 21.9, 19.2, 18.1, 13.8. HRMS (ESI) *m/z*: $[M+H]^+$ Calcd. for $C_{35}H_{53}N_8O_8^+$ 713.3981; Found 713.3990; error 1.3 ppm.

Nle-3-(Leu4). ^{1}H-NMR (600 MHz, DMSO-d_6): δ 9.20 (bs, 2 H), 8.70 (d, *J* = 8.4 Hz, 1 H), 8.07 (d, *J* = 8.6 Hz, 1 H), 8.03 (d, *J* = 8.3 Hz, 1 H), 7.67 (bs, 1 H), 6.93-6.98 (m, 4 H), 6.60-6.64 (m, 4 H), 4.43 (q, *J* = 4.9 Hz, 1 H), 4.30 (q, *J* = 5.2 Hz, 1 H), 4.16-4.21 (m, 2 H), 3.77 (t, *J* = 6.4 Hz, 1 H), 2.94-3.05 (m, 4 H), 2.78-2.85 (m, 2 H), 2.68-2.72 (m, 1 H), 1.63-1.70 (m, 3 H), 1.371.55 (m, 3 H), 1.19-1.25 (m, 4 H), 0.79-0.87 (m, 9 H). ^{13}C{^{1}H}-NMR (150 MHz, DMSO-d_6): δ 173.5, 171.4, 170.7, 170.5, 168.7, 157.0, 155.9, 155.8, 130.4, 130.2, 130.0, 127.7, 127.3, 114.9, 114.8, 54.4, 54.2, 52.3, 52.2, 51.6, 41.0, 40.5, 36.9, 35.8, 30.9, 29.1, 26.3, 24.3, 24.0, 23.0, 21.8, 21.5, 13.7. HRMS (ESI) *m/z*: $[M+H]^+$ Calcd. for $C_{36}H_{55}N_8O_8^+$ 727.4137; Found 727.4146; error 1.2 ppm.

Nle-3-(3,5-d$_2$-Tyr3, Trp4). HRMS (ESI) *m/z*: $[M+H]^+$ Calcd. for $C_{41}H_{52}D_2N_9O_8^+$ 802.4215; Found 802.4205; error 1.3 ppm.

Nle-3-(Trp4, 3,5-d$_2$-Tyr5). HRMS (ESI) *m/z*: $[M+H]^+$ Calcd. for $C_{41}H_{52}D_2N_9O_8^+$ 802.4215; Found 802.4210; error 0.7 ppm.

Nle-3-(Trp4, 3,5-d$_2$-Tyr3,5). HRMS (ESI) *m/z*: $[M+H]^+$ Calcd. for $C_{41}H_{50}D_4N_9O_8^+$ 804.4341; Found 804.4333; error 1.0 ppm.

Nle-3-(3,5-d$_2$-Tyr3, Leu4). HRMS (ESI) *m/z*: $[M+H]^+$ Calcd. for $C_{36}H_{53}D_2N_8O_8^+$ 729.4263; Found 729.4263; error 0.1 ppm.

Nle-3-(Leu4, 3,5-d$_2$-Tyr5). HRMS (ESI) *m/z*: $[M+H]^+$ Calcd. for $C_{36}H_{53}D_2N_8O_8^+$ 729.4263; Found 729.4263; error 0.1 ppm.

Nle-3-(Leu4, His5). ^{1}H-NMR (400 MHz, DMSO-d_6): δ 8.61 (d, *J* = 8.0 Hz, 1 H), 8.38 (s, 1 H), 8.20 (d, *J* = 7.8 Hz, 1 H), 8.16 (d, *J* = 8.0 Hz, 1 H), 8.09 (d, *J* = 8.2 Hz, 1 H), 7.94 (bs, 1 H), 7.14 (s, 1 H), 7.00 (d, *J* = 8.5 Hz, 2 H), 6.60 (d, *J* = 8.5 Hz, 2 H), 4.42-4.49 (m, 2 H), 4.30-4.40 (m, 1 H), 4.21-4.26 (m, 1 H), 3.77 (t, *J* = 6.2 Hz, 1 H), 3.05-3.10 (m, 3 H), 2.86-2.99 (m, 2 H), 2.64-2.70 (m, 1 H), 1.60-1.66 (m, 3 H), 1.38-1.57 (m, 6 H), 1.21-1.25 (m, 4 H), 0.80-0.87 (m, 9 H). ^{13}C{^{1}H}-NMR (100 MHz, DMSO-d_6): δ 172.3, 171.9, 171.0, 170.6, 168.5, 156.9, 155.8, 134.0, 130.0, 127.6, 118.7, 115.7, 114.8, 54.0, 52.2, 51.7, 51.2, 40.7, 40.5, 36.6, 30.9, 29.4, 26.2, 24.7, 24.0, 23.0, 21.7, 21.6 ,13.7. HRMS (ESI) *m/z*: $[M+H]^+$ Calcd. for $C_{33}H_{53}N_{10}O_7^+$ 701.4093; Found 701.4086; error 1.0 ppm.

Nle-3-(Leu4, Trp5). ^{1}H-NMR (400 MHz, DMSO-d_6): δ 10.85 (s, 1 H), 9.15 (s, 1 H), 8.53 (d, *J* = 8.2 Hz, 1 H), 8.04-8.09 (m, 5 H), 7.60 (bs, 1 H), 7.52 (d, *J* = 7.9 Hz, 1 H), 7.32 (d, *J* = 8.0 Hz, 1 H), 7.13 (d, *J* = 2.5 Hz, 1 H), 7.05 (t, *J* = 8.2 Hz, 1 H), 695-6.99 (m, 3 H), 6.59 (d, *J* = 8.5 Hz, 2 H), 4.43-4.50 (m, 2 H), 4.31-4.38 (m, 2 H), 3.75 (bs, 1 H), 3.14-3.20 (m, 1 H), 3.05-3.10 (m, 3 H), 2.86 (dd, *J* = 14.7 Hz, 3.6 Hz, 1 H), 2.63-2.69 (m, 1 H), 1.56-1.64 (m, 4 H), 1.38-1.50 (m, 5 H), 0.87 (d, *J* = 6.6 Hz, 3 H), 0.80-0.83 (m, 6 H).

$^{13}C\{^1H\}$-NMR (100 MHz, DMSO-d_6): δ 173.1, 171.9, 170.7, 170.6, 168.4, 158.4, 158.1, 156.8, 155.8, 136.1, 130.0, 127.6, 127.3, 123.5, 120.9, 118.7, 118.4, 118.2, 114.8, 111.3, 109.6, 92.4, 53.8, 52.9, 52.1, 51.0, 41.0, 40.5, 36.5, 30.9, 29.6, 27.1, 26.2, 24.8, 24.1, 23.1, 21.7, 21.6 ,13.7. HRMS (ESI) *m/z*: $[M+H]^+$ Calcd. for $C_{38}H_{56}N_9O_7^+$ 750.4297; 750.4294; error 0.5 ppm.

Nle-6. ^1H-NMR (400 MHz, DMSO-d_6): δ 8.63 (bs, 1 H), 8.25 (d, *J* = 7.5 Hz, 1 H), 8.13 (d, *J* = 8.6 Hz, 1 H), 8.00 (bs, 1 H), 7.93 (d, *J* = 8.9 Hz, 1 H), 7.00 (d, *J* = 8.3 Hz, 3 H), 6.59 (d, *J* = 8.4 Hz, 2 H), 4.42-4.54 (m, 2 H), 4.34 (bs, 1 H), 4.12 (t, *J* = 6.6 Hz, 1 H), 3.76 (bs, 1 H), 3.00-3.07 (m, 2 H), 2.86-2.95 (m, 2 H), 2.66-2.73 (m, 1 H), 1.91-1.99 (m, 1 H), 1.63 (bs, 3 H), 1.49 (bs, 3 H), 1.21-1.25 (m, 4 H), 0.82 (t, *J* = 7.0 Hz, 9 H). $^{13}C\{^1H\}$-NMR (100 MHz, DMSO-d_6): δ 172.6, 171.0, 170.7, 168.4, 159.5, 156.8, 155.8, 150.2, 137.2, 134.3, 130.0, 127.6, 119.2, 114.8, 111.5, 54.1, 52.2, 36.6, 30.9, 30.6, 26.2, 24.7, 21.7, 19.1, 18.1, 13.7. HRMS (ESI) *m/z*: $[M+H]^+$ Calcd. for $C_{32}H_{51}N_{10}O_7^+$ 687.3937; Found 687.3942; error 0.8 ppm.

Nle-6-(3,5-d$_2$-Tyr3, Leu4). HRMS (ESI) *m/z*: $[M+H]^+$ Calcd. for $C_{33}H_{51}D_2N_{10}O_7^+$ 703.4219; Found 703.4222; error 0.5 ppm.

Nle-6-(Leu4, 2-d-His5). HRMS (ESI) *m/z*: $[M+H]^+$ Calcd. for $C_{33}H_{52}DN_{10}O_7^+$ 702.4156; Found 702.4152; error 0.5 ppm.

Fmoc-L-tyrosine-(3,5-phenyl-d$_2$). ^1H-NMR (400 MHz, DMSO-d_6): δ 9.20 (bs, 1 H), 7.86-7.92 (m, 2 H), 7.63-7.66 (m, 3 H), 7.26-7.43 (m, 5 H), 7.06 (s, 1 H), 4.10-4.22 (m, 4 H), 2.91–2.99 (m, 1 H), 2.75-2.79 (m, 1 H). $^{13}C\{^1H\}$-NMR (100 MHz, DMSO-d_6): 173.5, 155.9, 155.8, 143.8, 140.7, 129.9, 127.6, 127.0, 125.3, 125.3, 120.3, 120.1, 65.6, 55.9, 46.6, 35.8.

4. LCMS characterisation of peptides

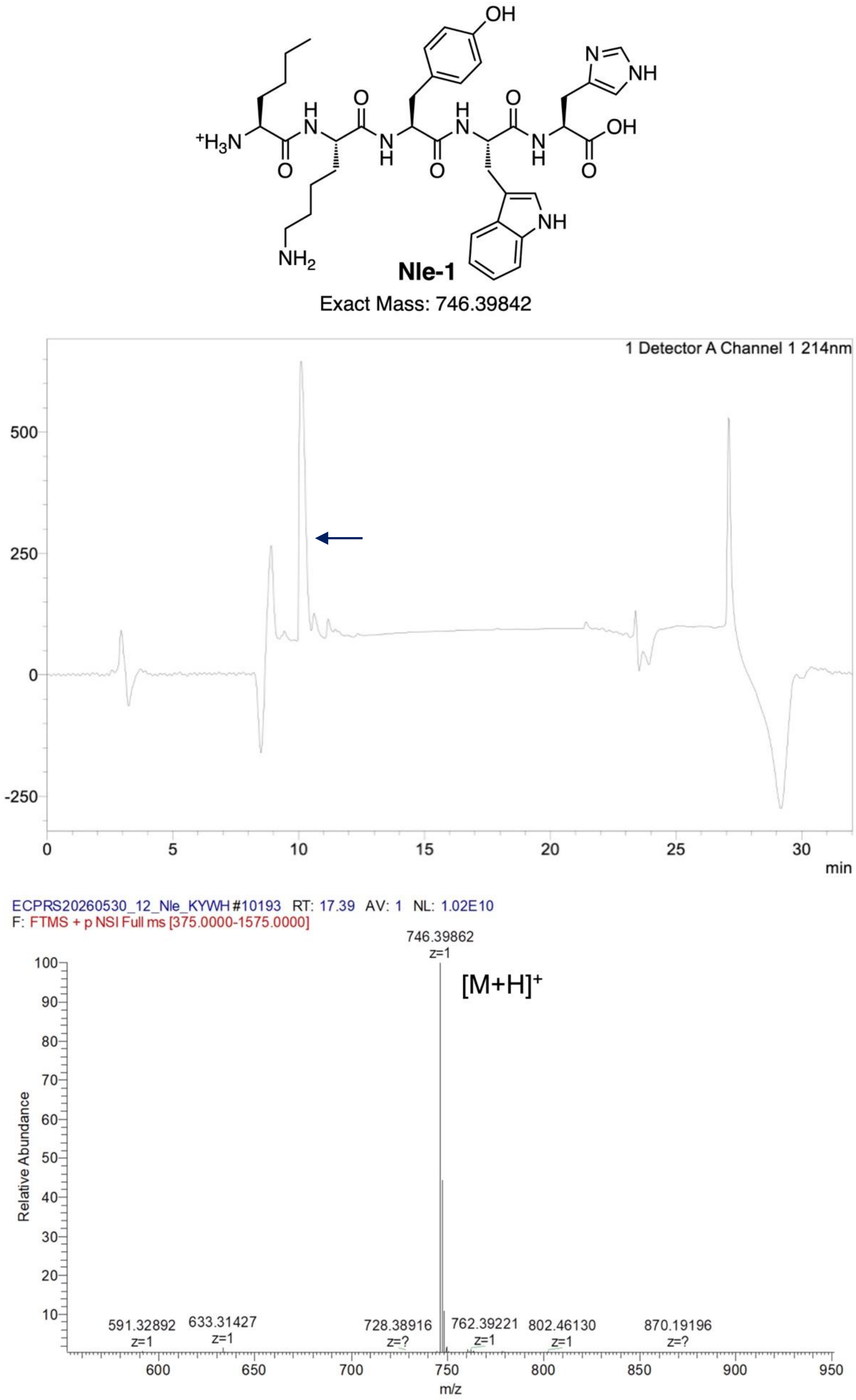

Figure S7. LCMS characterisation of **Nle-1**. Peptides were analysed via LCMS (Shimadzu LCMS-2020) on a gradient of 5-95% acetonitrile + 0.1% FA/water + 0.1% FA in 20 minutes. The arrow indicates the desired product peak.

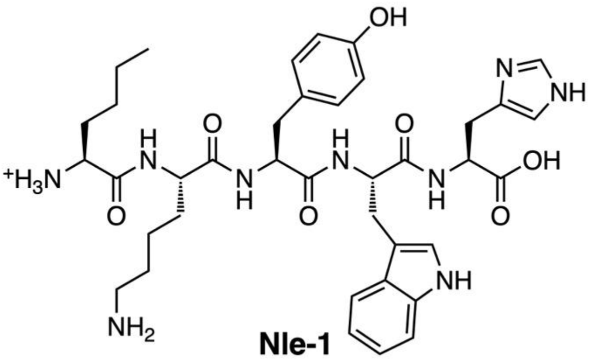

Nle-1

Exact Mass: 746.39842

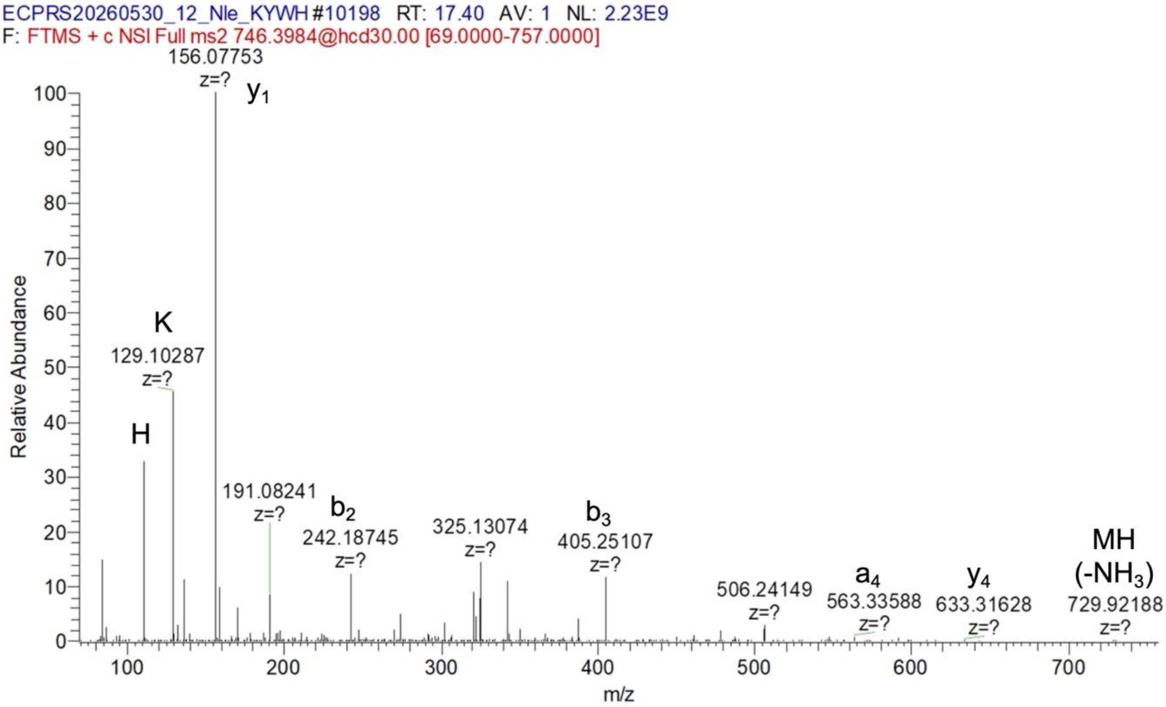

Figure S8. Annotated MS^2 spectrum of **Nle-1**.

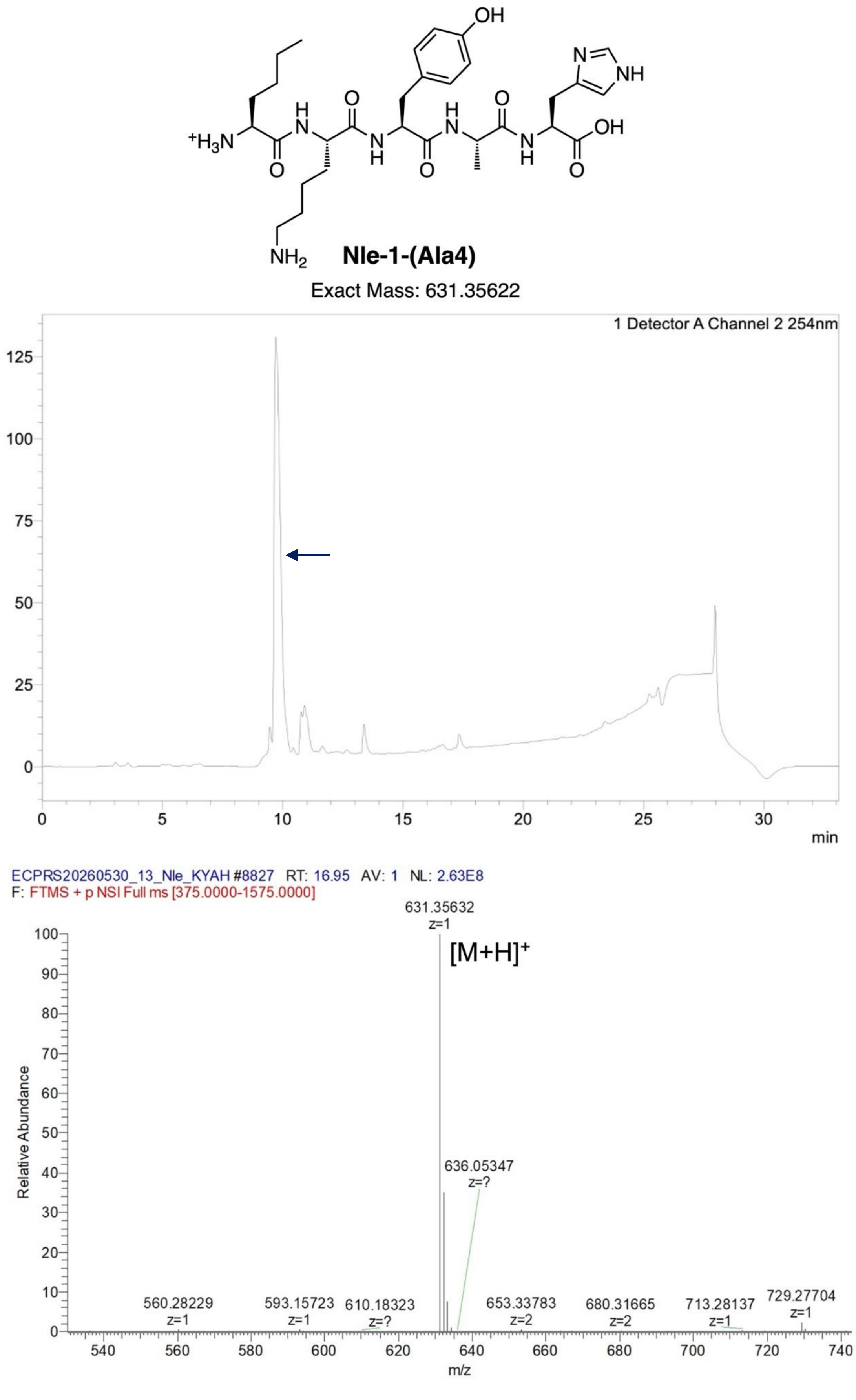

Figure S9. LCMS characterisation of **Nle-1-(Ala4)**. The arrow indicates the desired product peak.

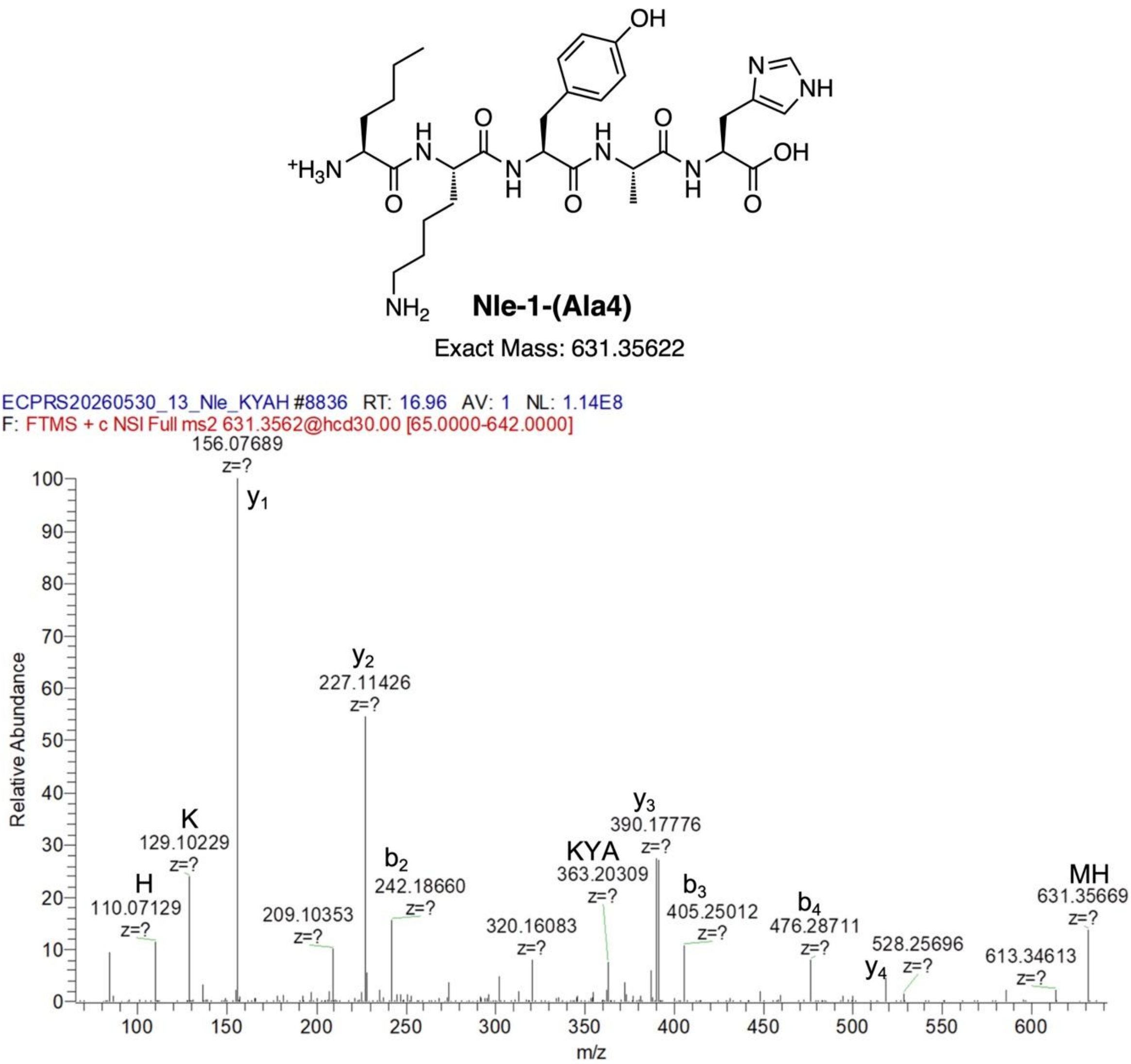

Figure S10. Annotated MS^2 spectrum of **Nle-1-(Ala4)**.

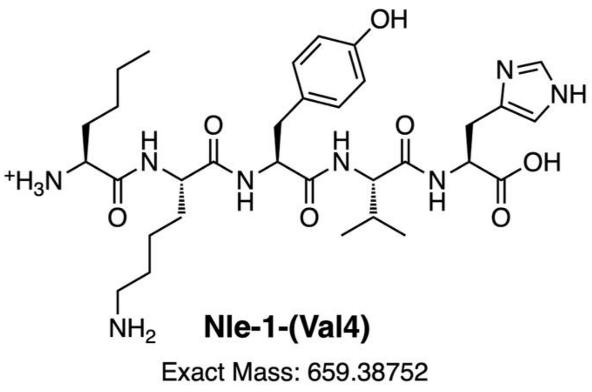

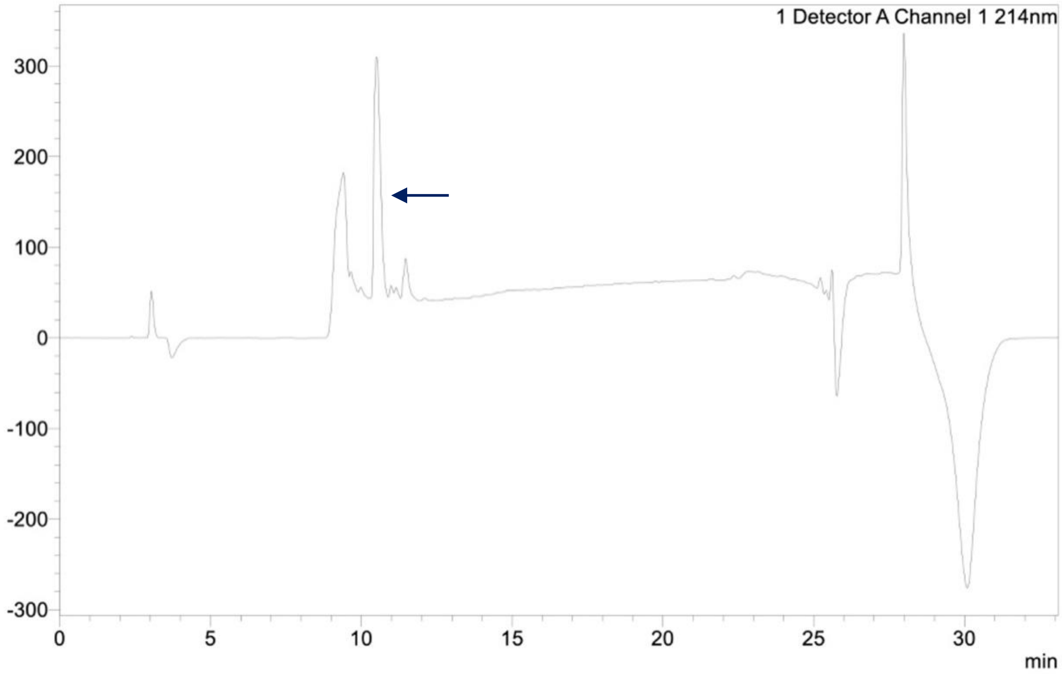

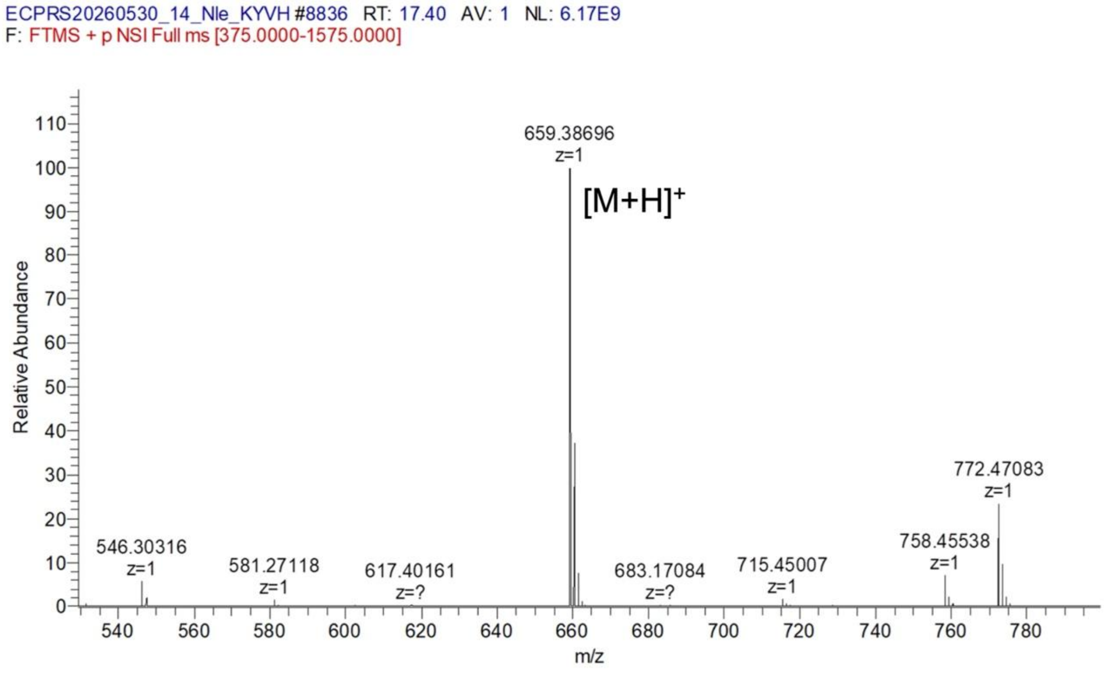

Figure S11. LCMS characterisation of **Nle-1-(Val4)**. The arrow indicates the desired product peak.

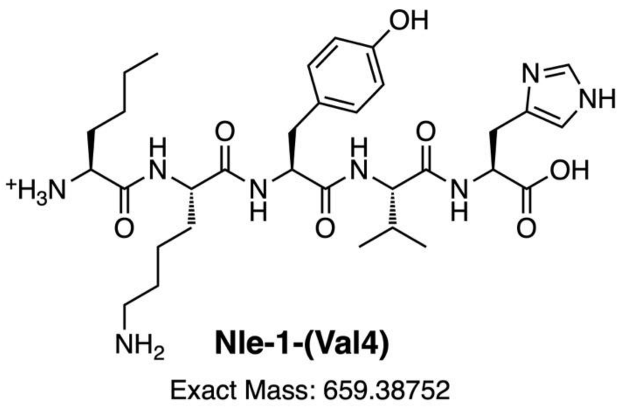

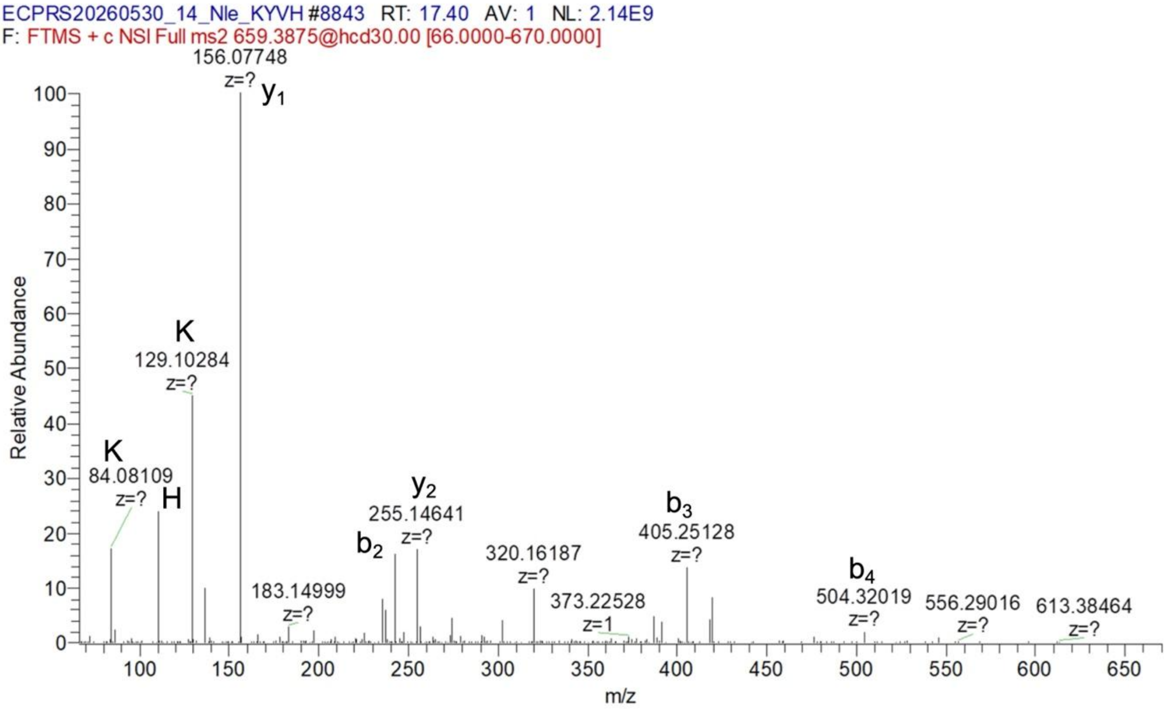

Figure S12. Annotated MS^2 spectrum of **Nle-1-(Val4)**.

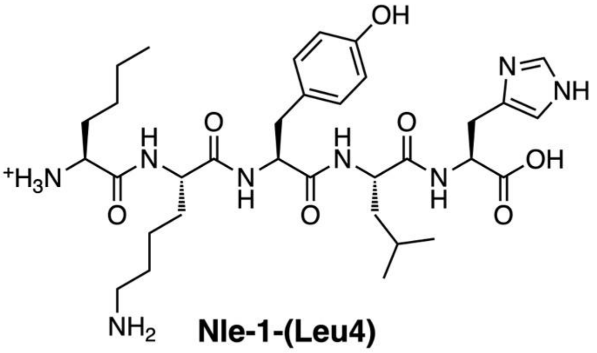

Nle-1-(Leu4)

Exact Mass: 673.40317

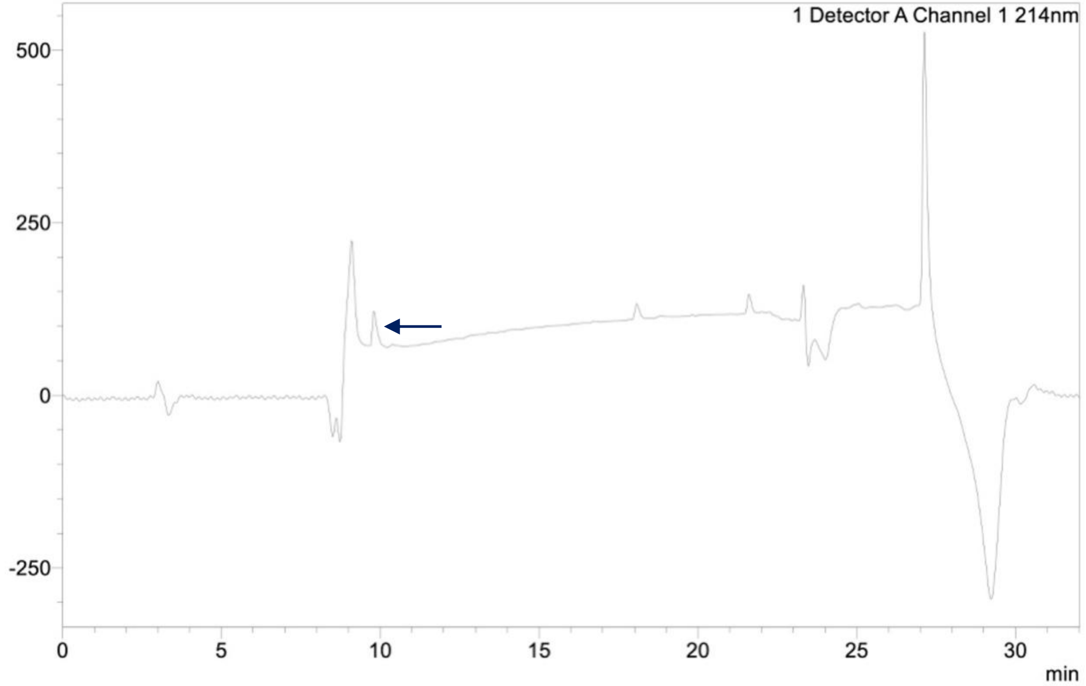

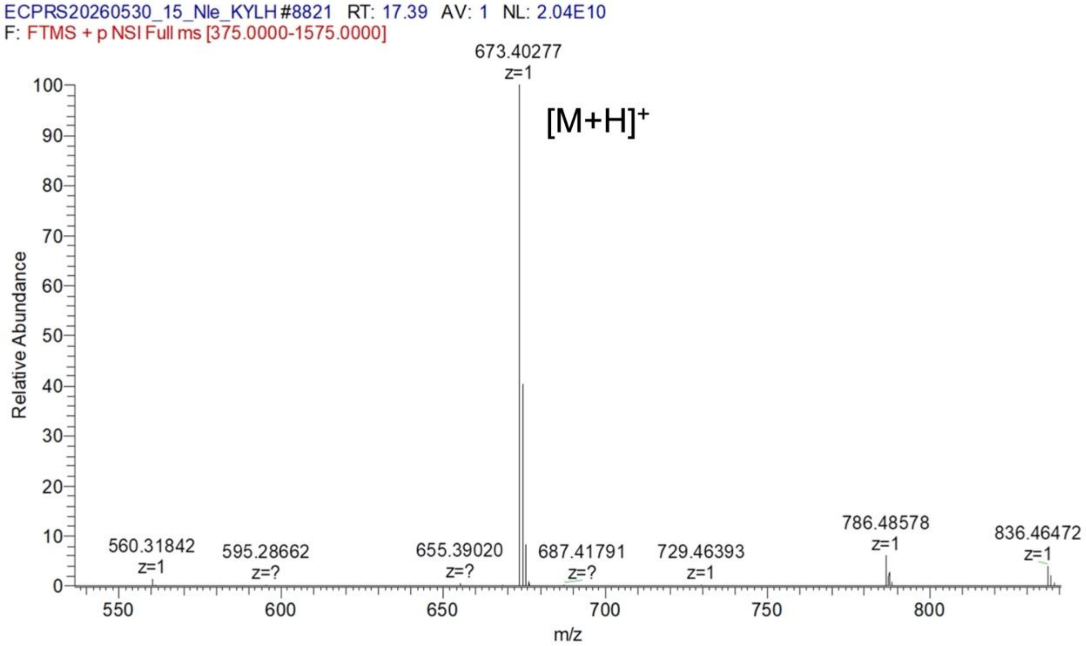

Figure S13. LCMS characterisation of **Nle-1-(Leu4)**. The arrow indicates the desired product peak.

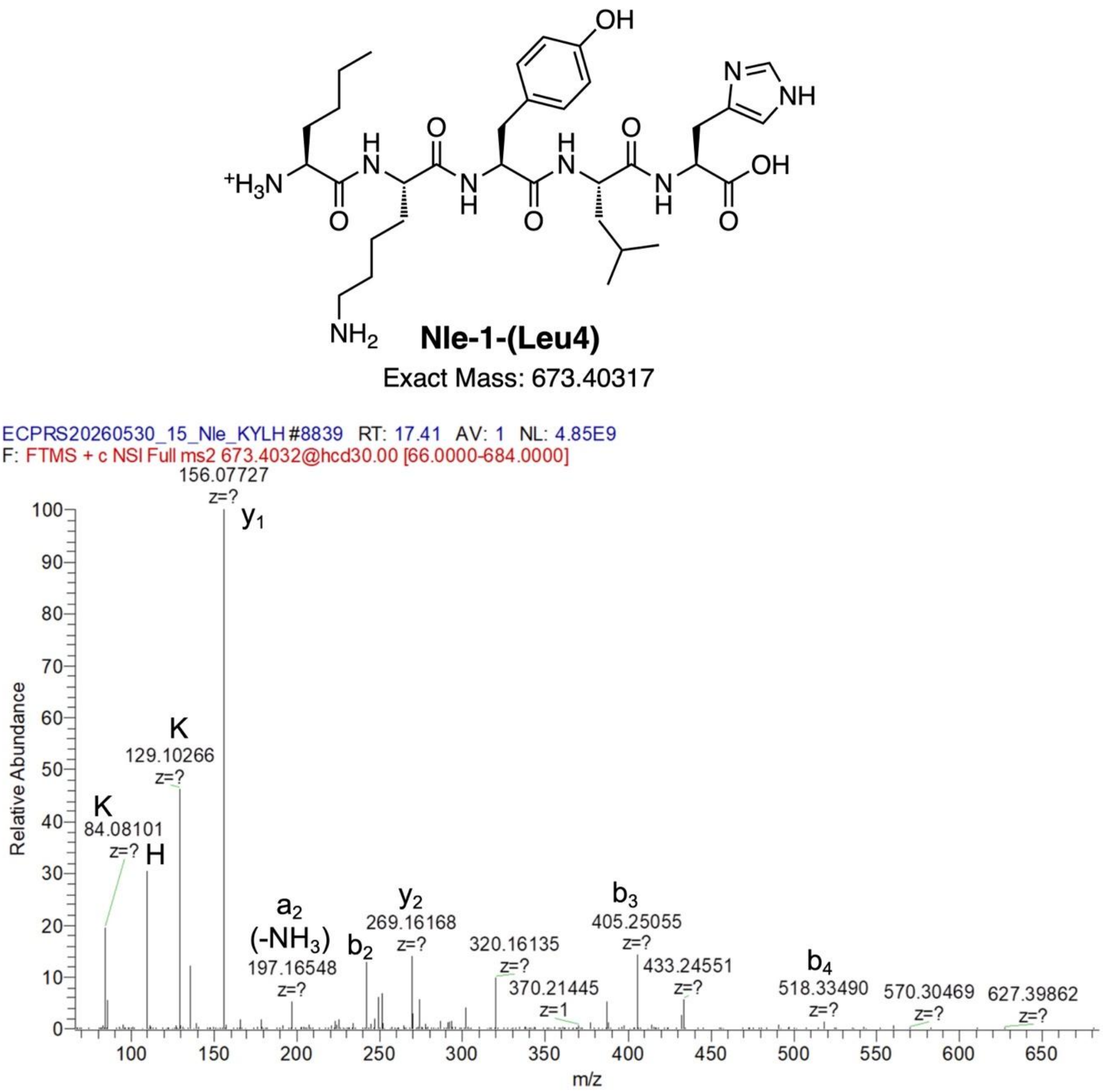

Figure S14. Annotated MS^2 spectrum of **Nle-1-(Leu4)**.

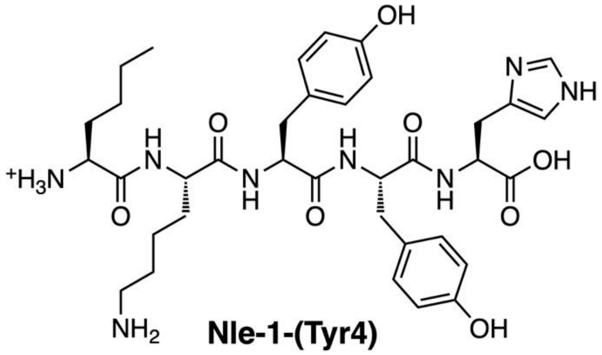

Nle-1-(Tyr4)

Exact Mass: 723.38244

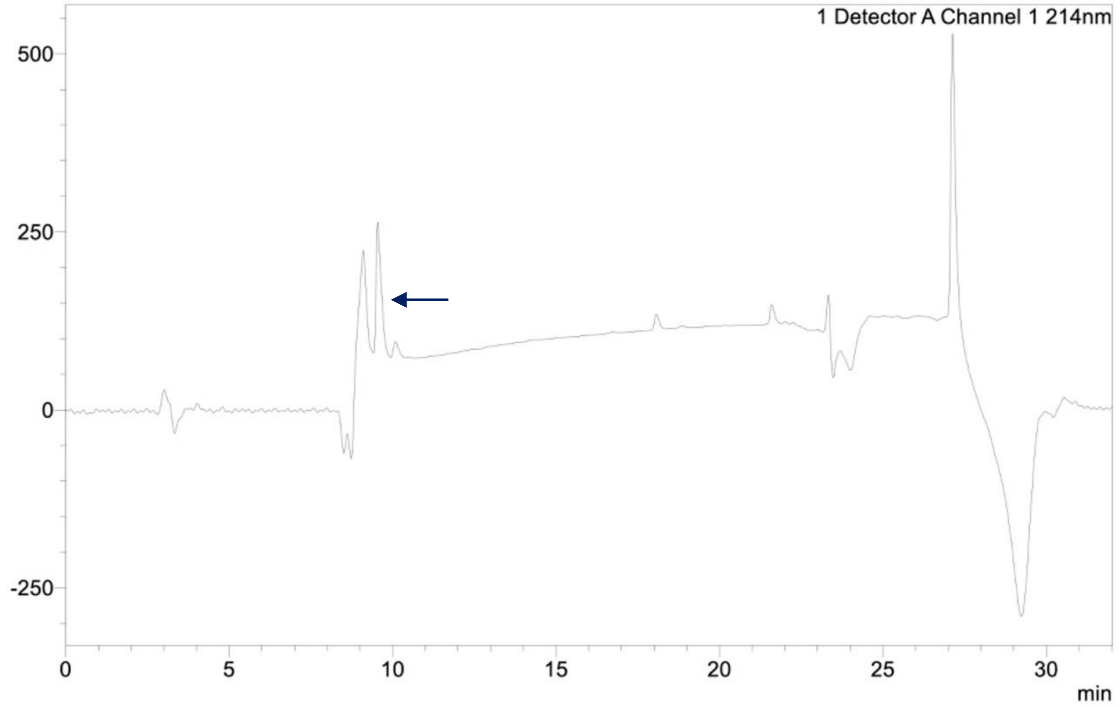

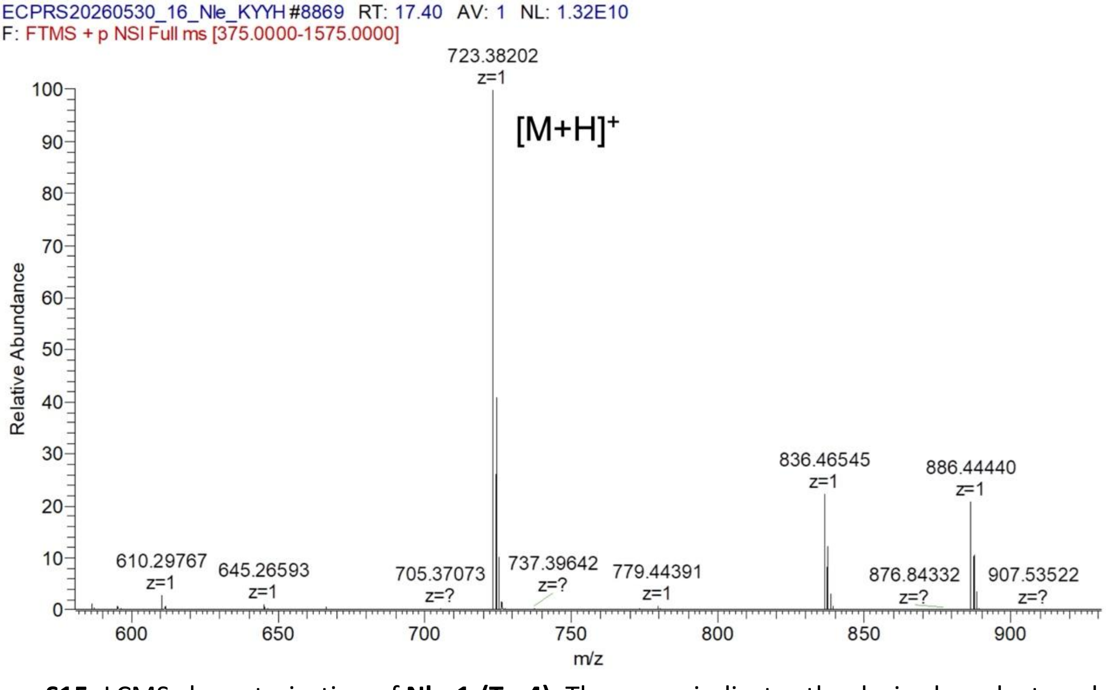

Figure S15. LCMS characterisation of **Nle-1-(Tyr4)**. The arrow indicates the desired product peak.

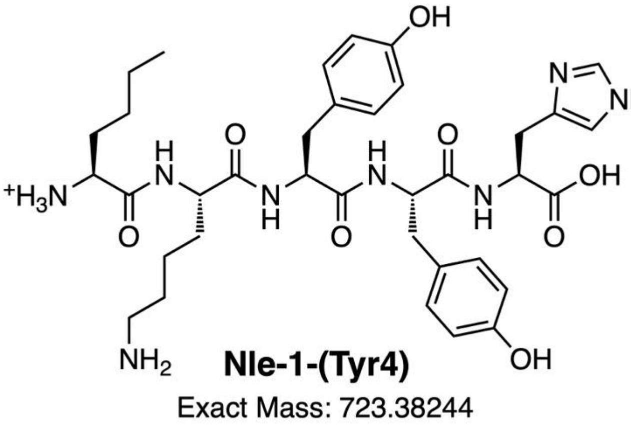

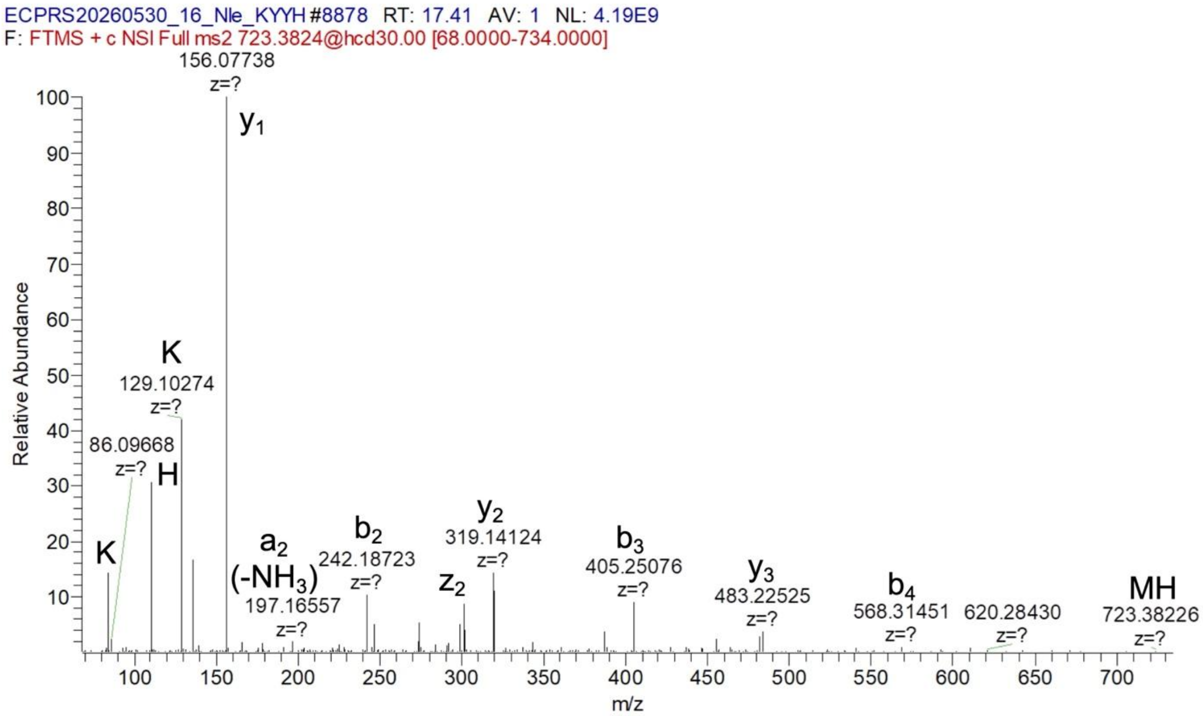

Figure S16. Annotated MS^2 spectrum of **Nle-1-(Tyr4)**.

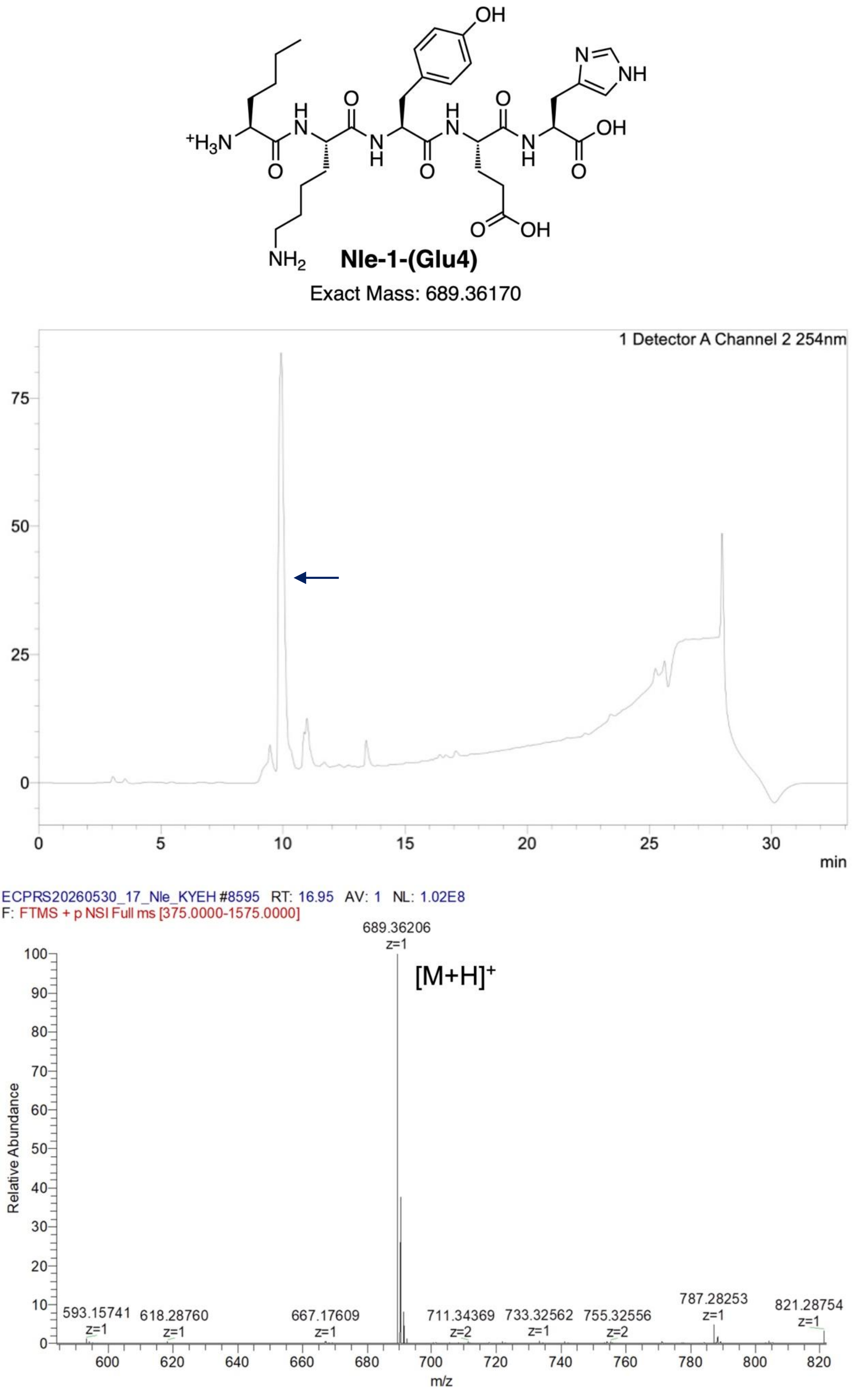

Figure S17. LCMS characterisation of **Nle-1-(Glu4)**. The arrow indicates the desired product peak.

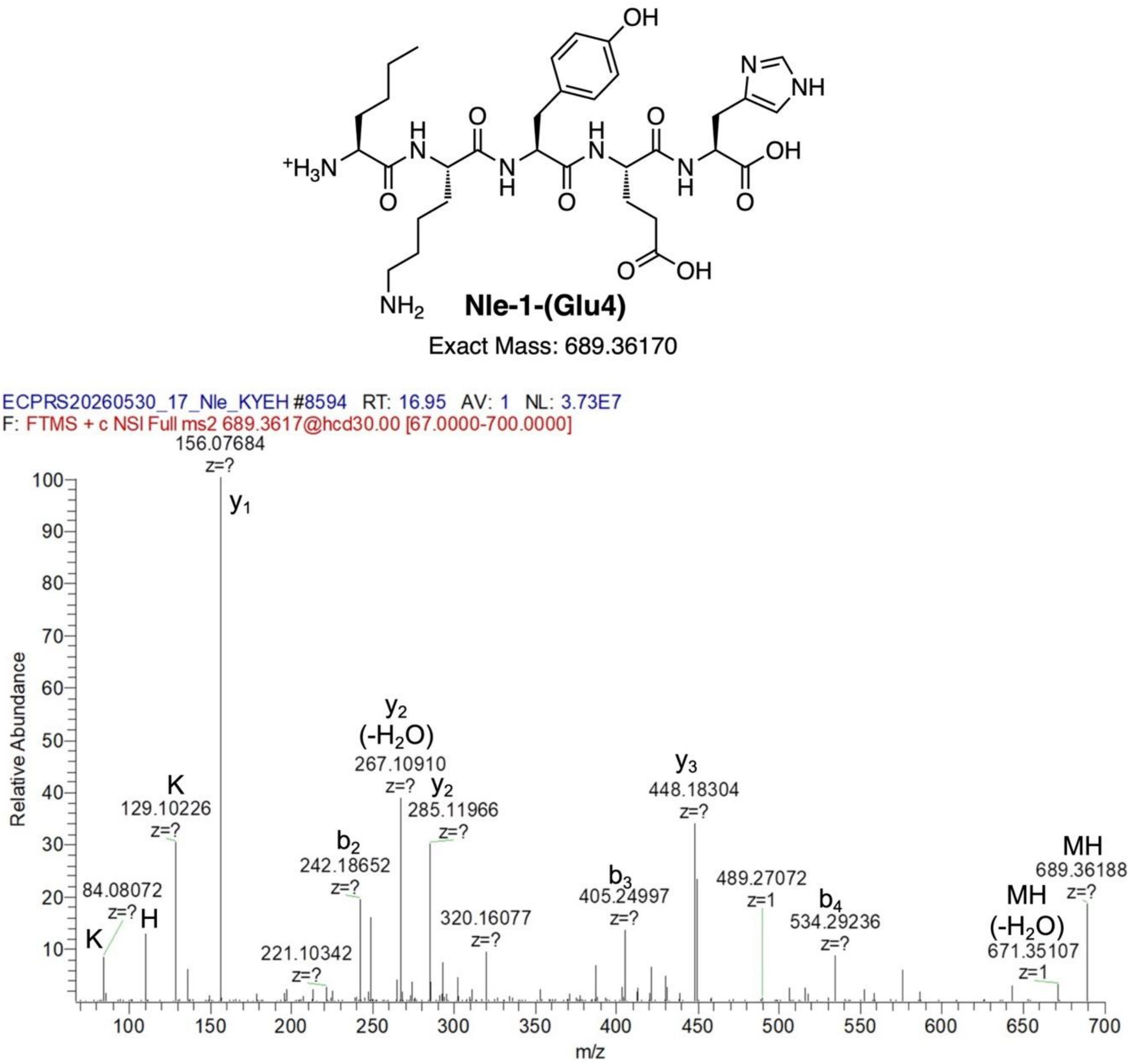

Figure S18. Annotated MS^2 spectrum of **Nle-1-(Glu4)**.

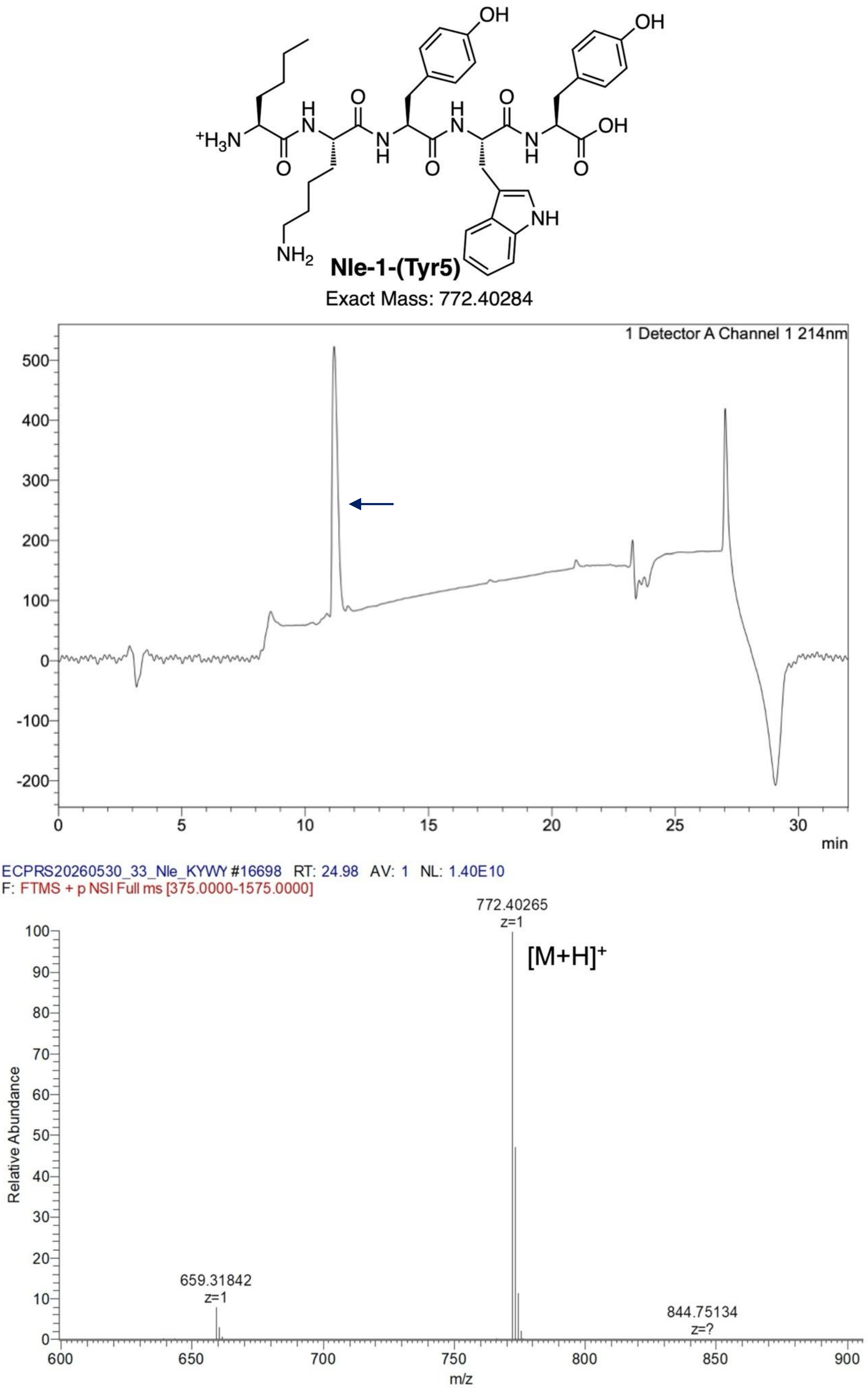

Figure S19. LCMS characterisation of **Nle-1-(Tyr5)**. The arrow indicates the desired product peak.

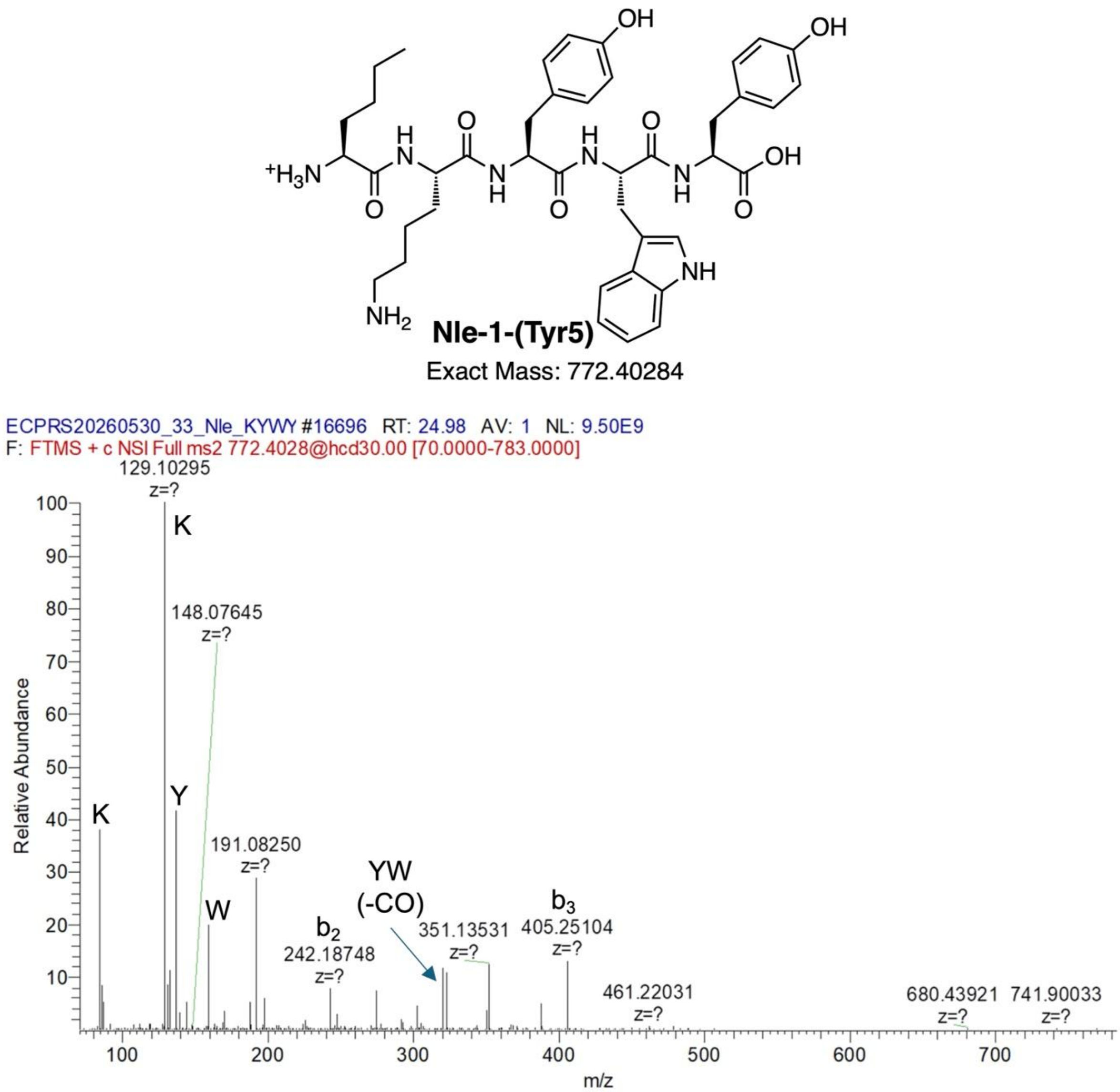

Figure S20. Annotated MS^2 spectrum of **Nle-1-(Tyr5)**.

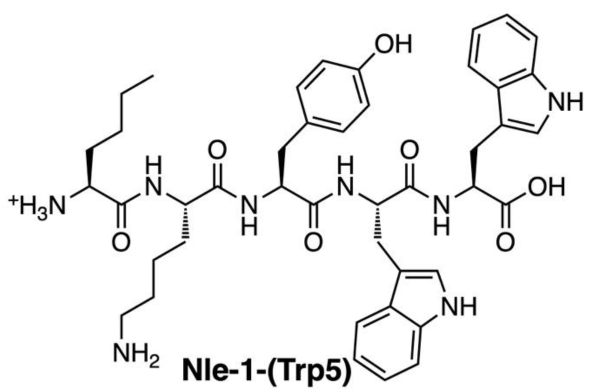

Nle-1-(Trp5)

Exact Mass: 795.41882

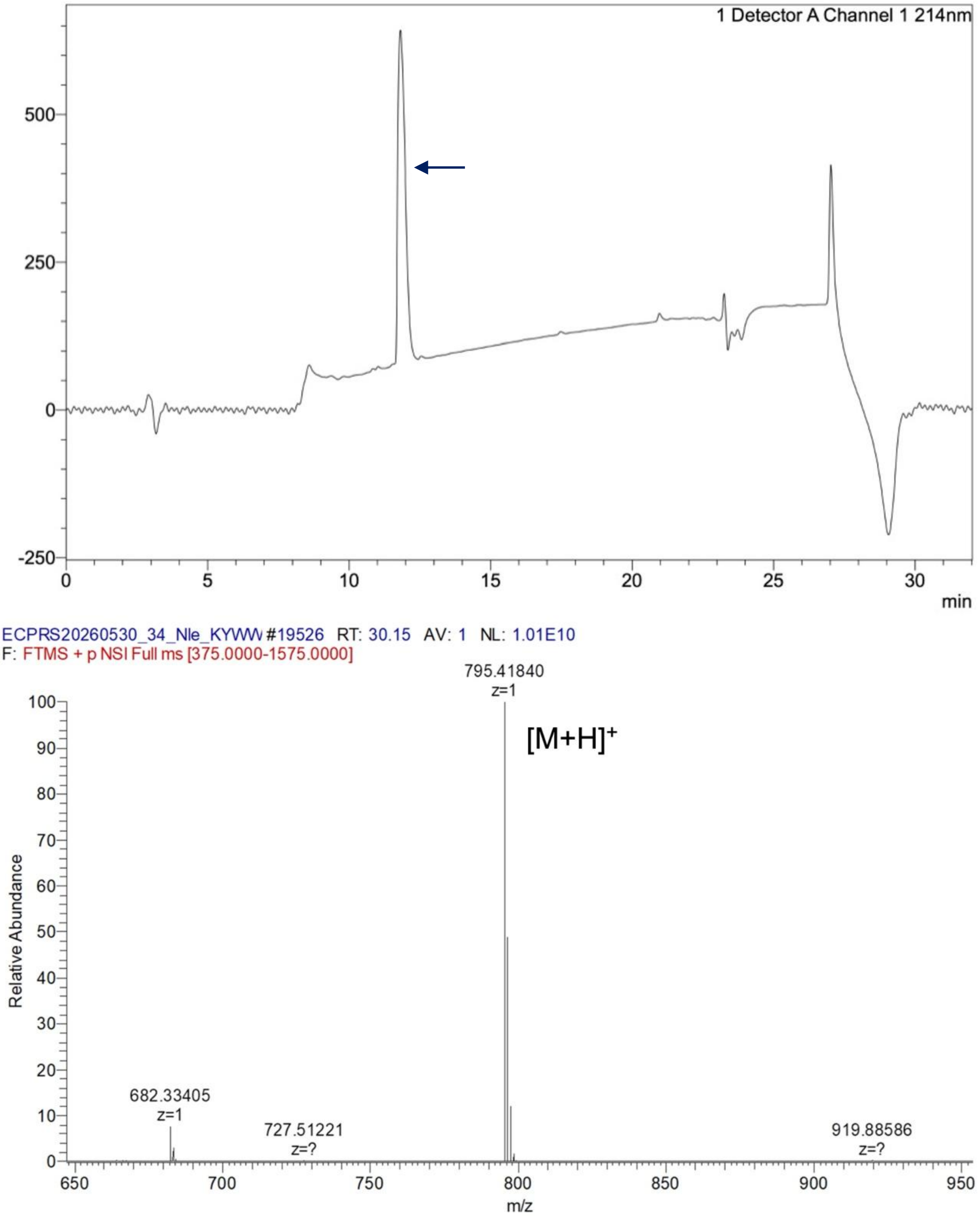

Figure S21. LCMS characterisation of **Nle-1-(Trp5)**. The arrow indicates the desired product peak.

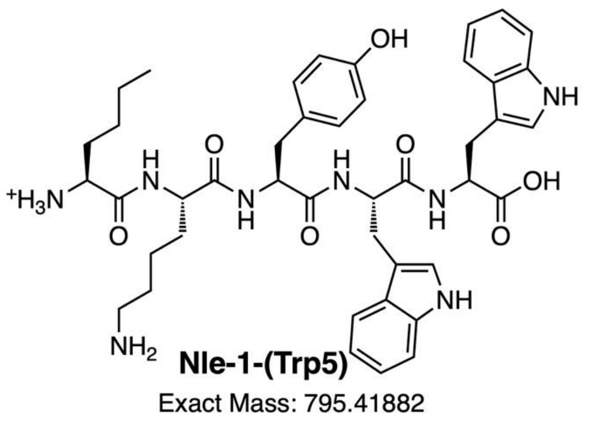

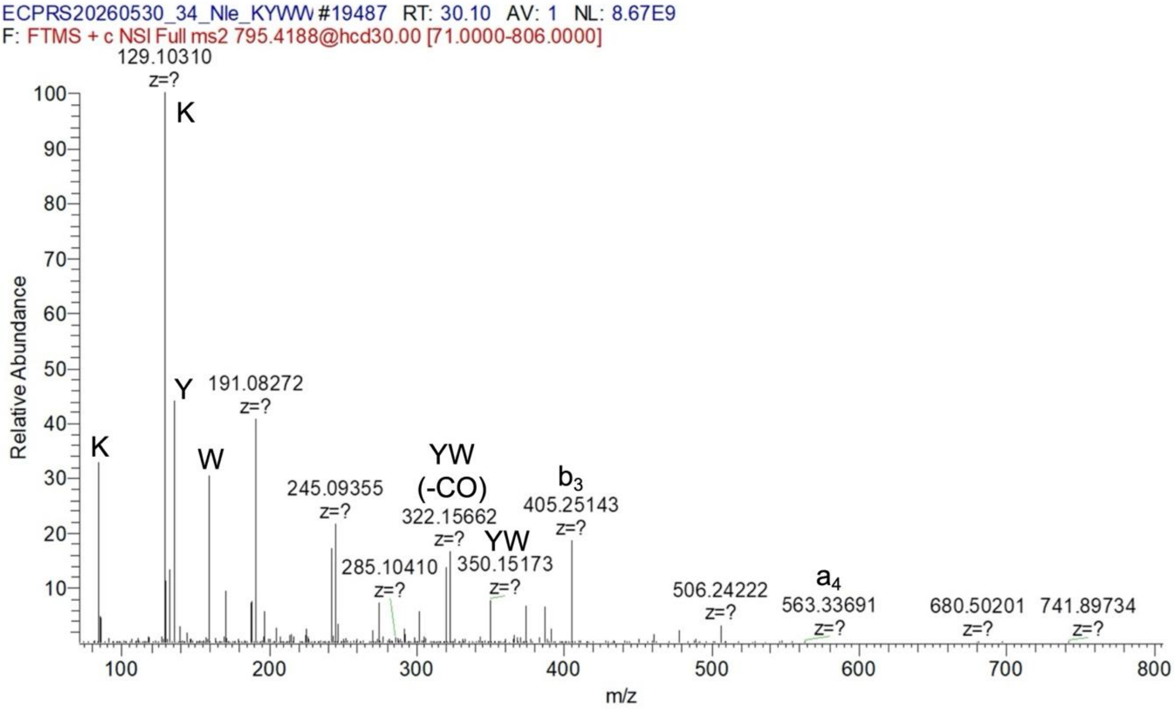

Figure S22. Annotated MS^2 spectrum of **Nle-1-(Trp5)**.

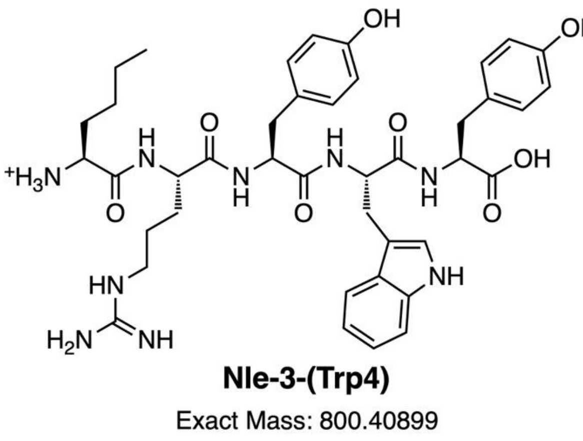

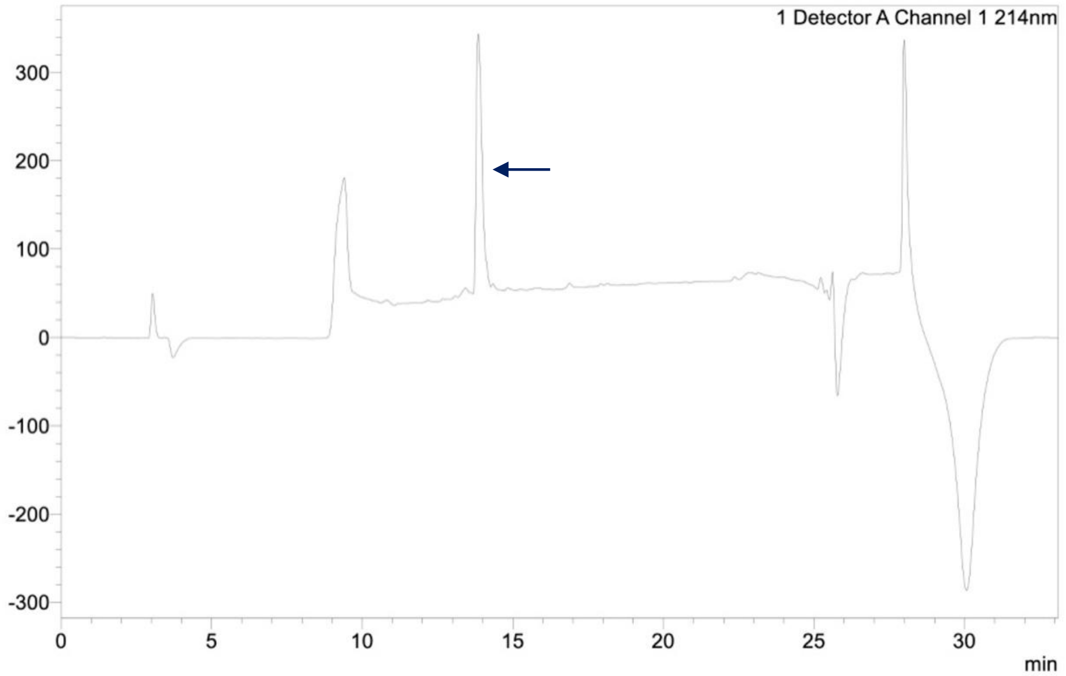

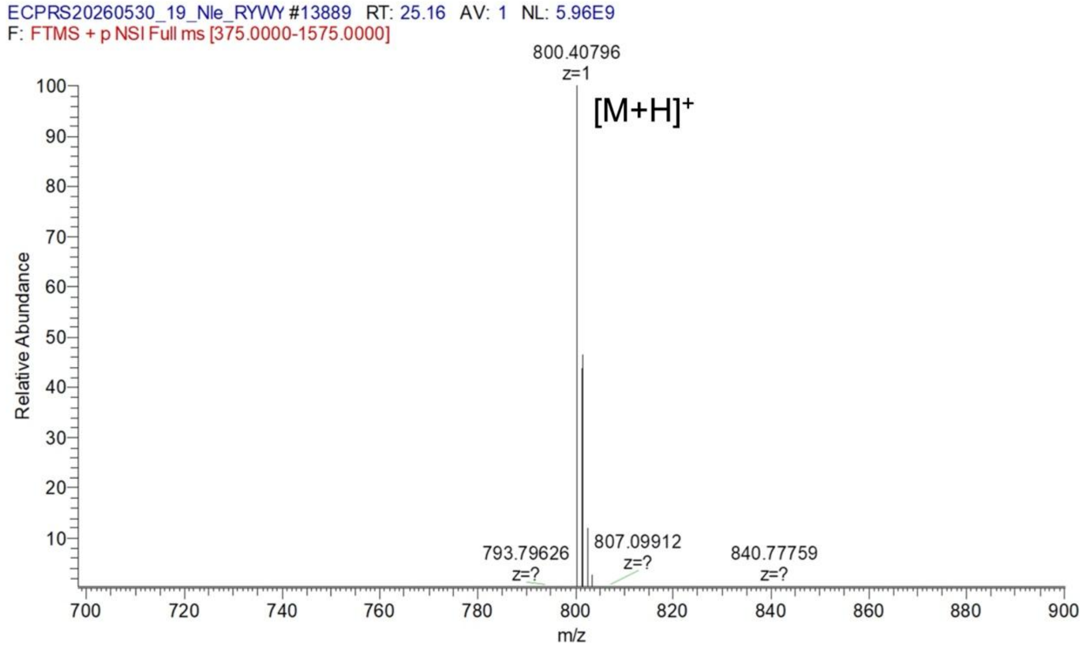

Figure S23. LCMS characterisation of **Nle-3-(Trp4)**. The arrow indicates the desired product peak.

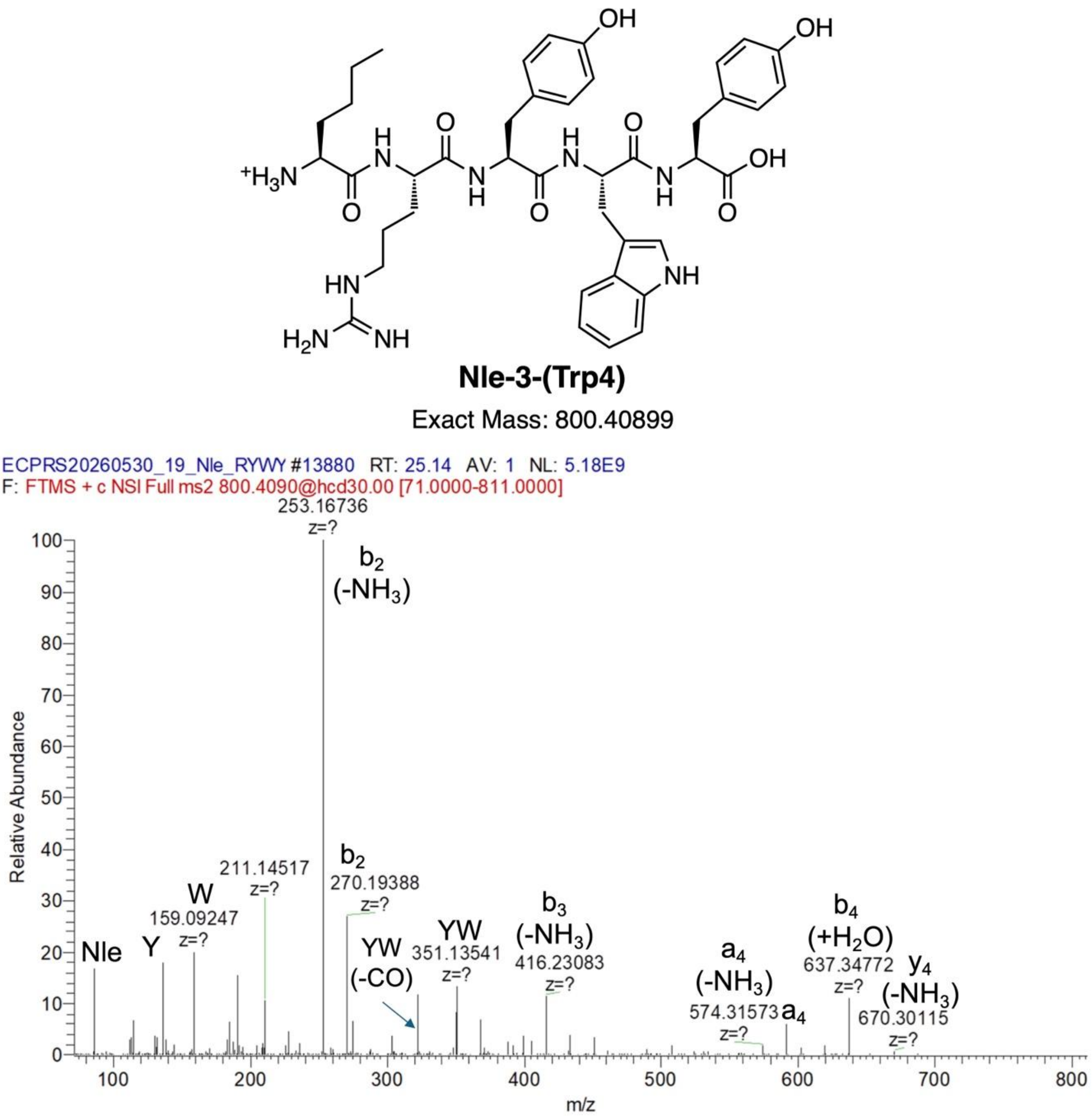

Figure S24. Annotated MS^2 spectrum of **Nle-3-(Trp4)**.

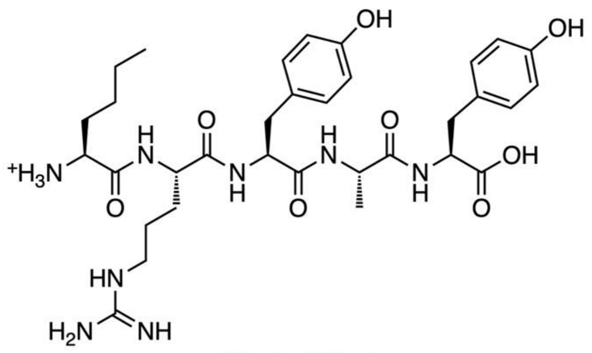

Nle-3-(Ala4)

Exact Mass: 685.36679

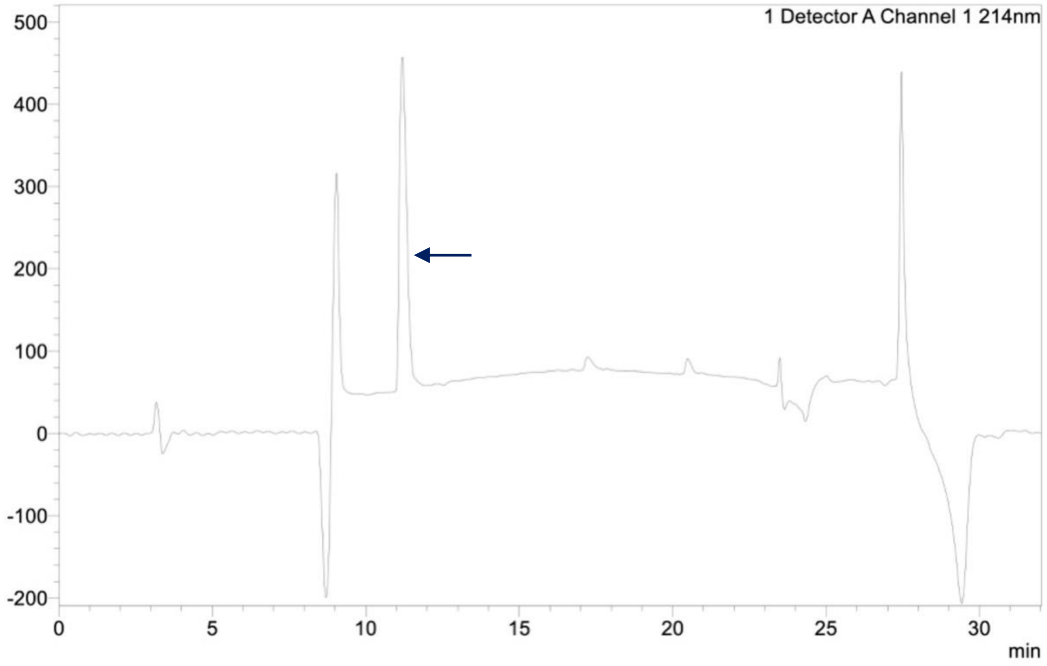

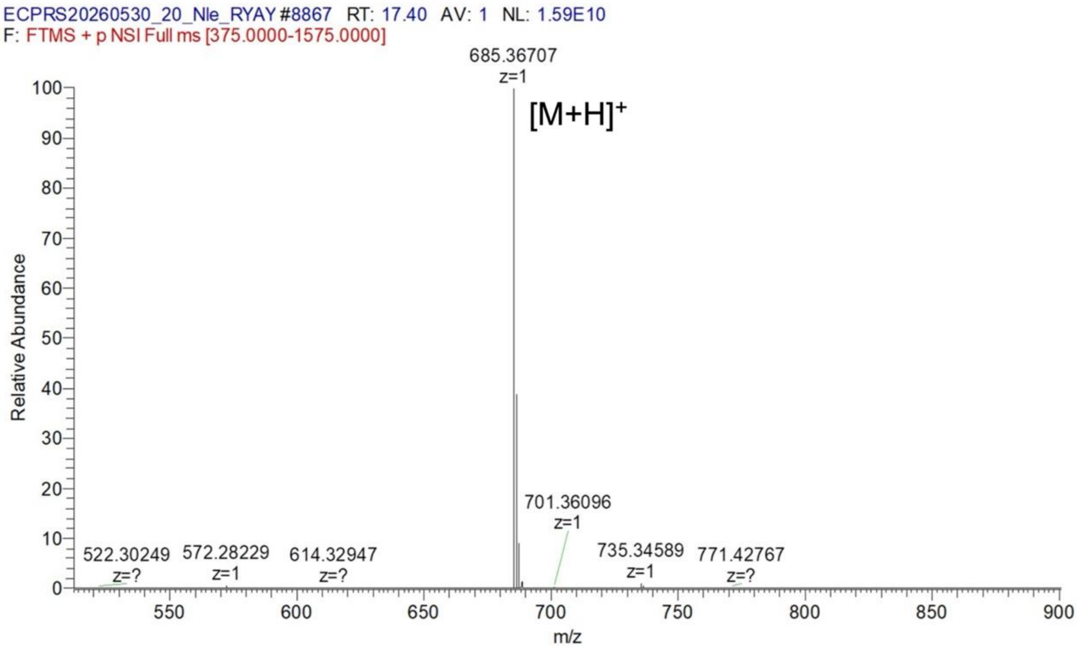

Figure S25. LCMS characterisation of **Nle-3-(Ala4)**. The arrow indicates the desired product peak.

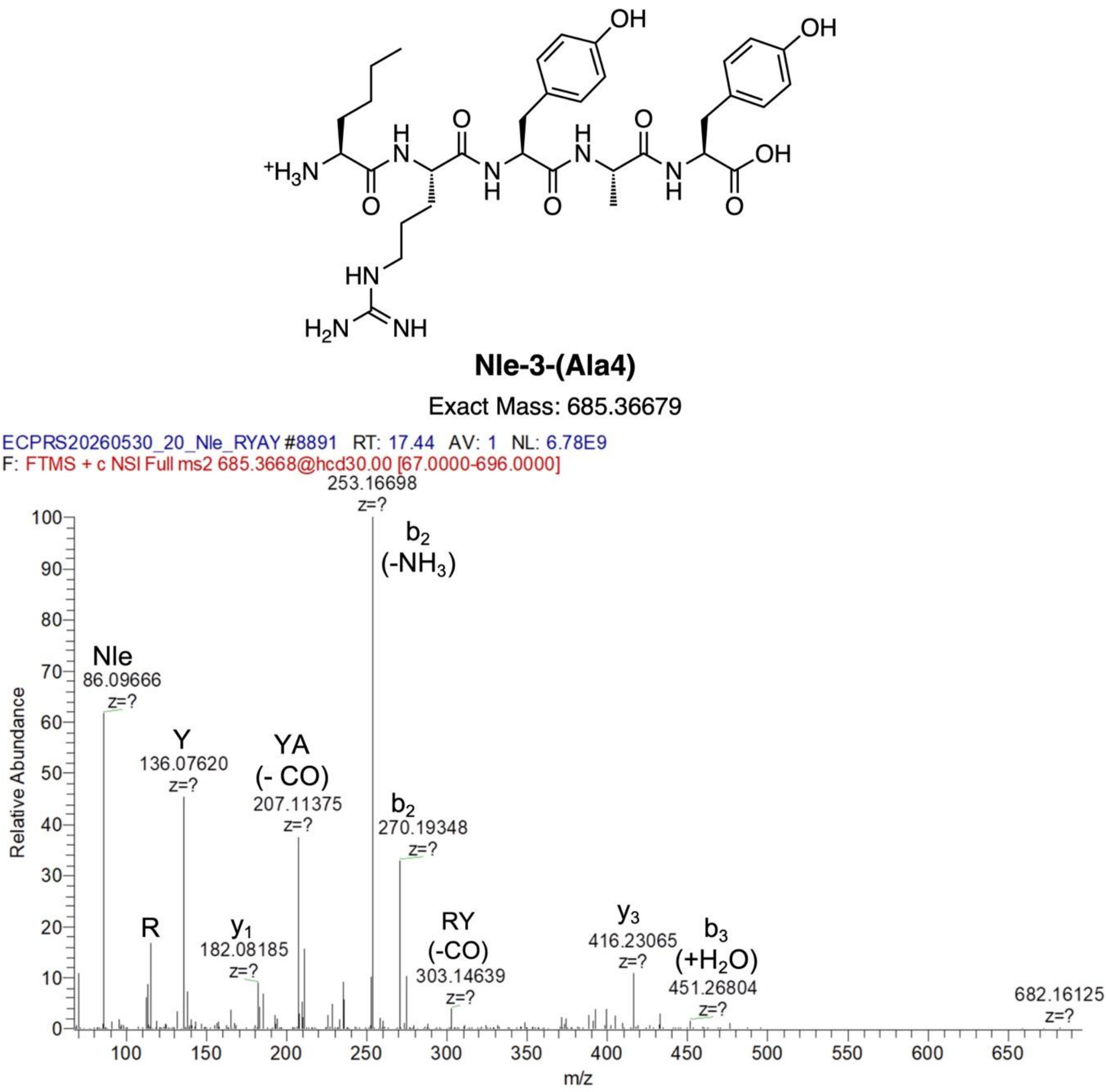

Figure S26. Annotated MS^2 spectrum of **Nle-3-(Ala4)**.

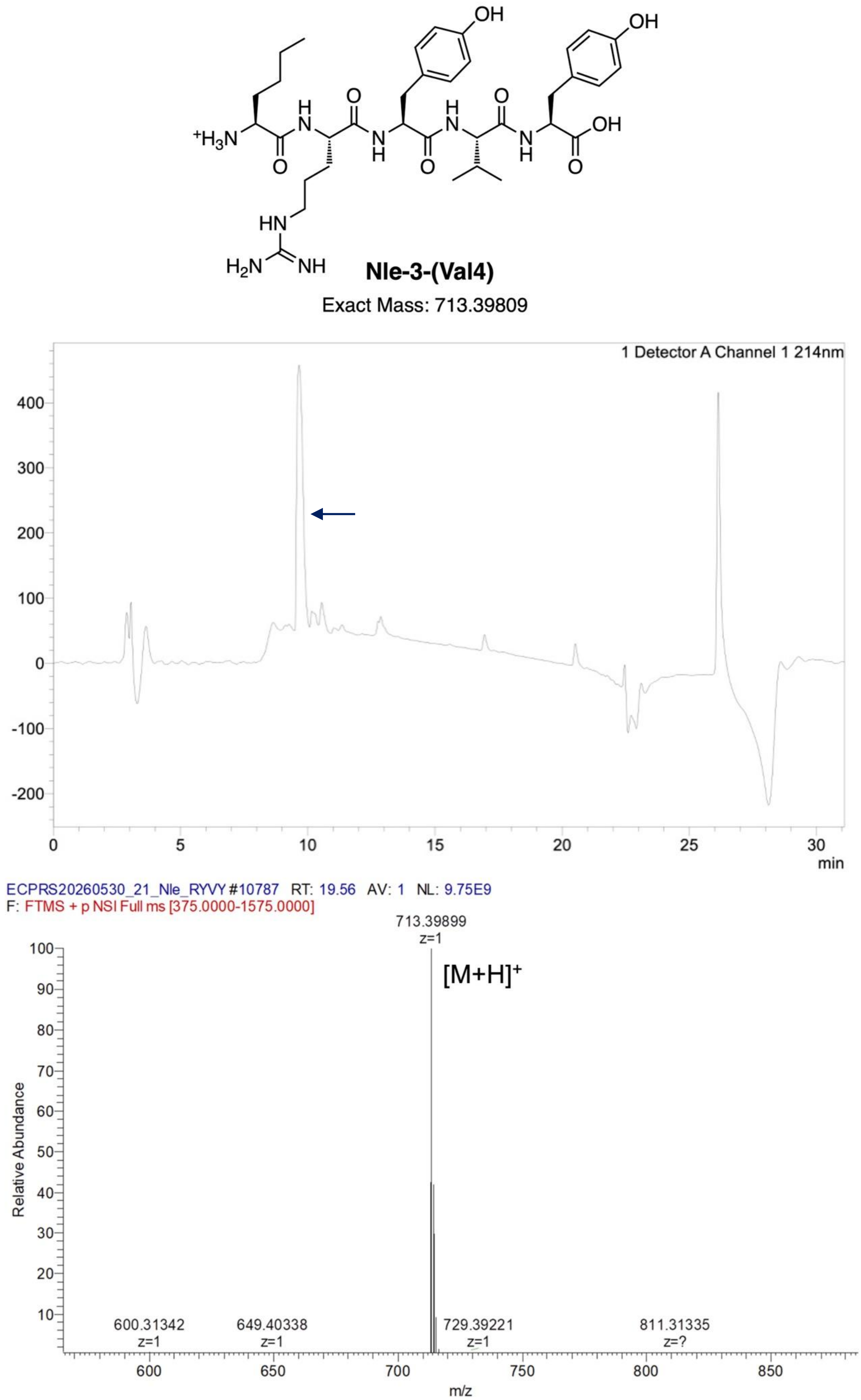

Figure S27. LCMS characterisation of **Nle-3-(Val4)**. The arrow indicates the desired product peak.

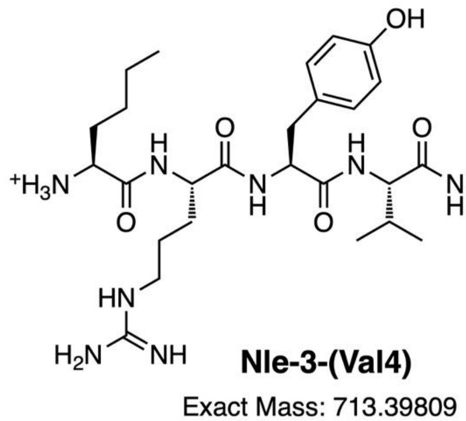

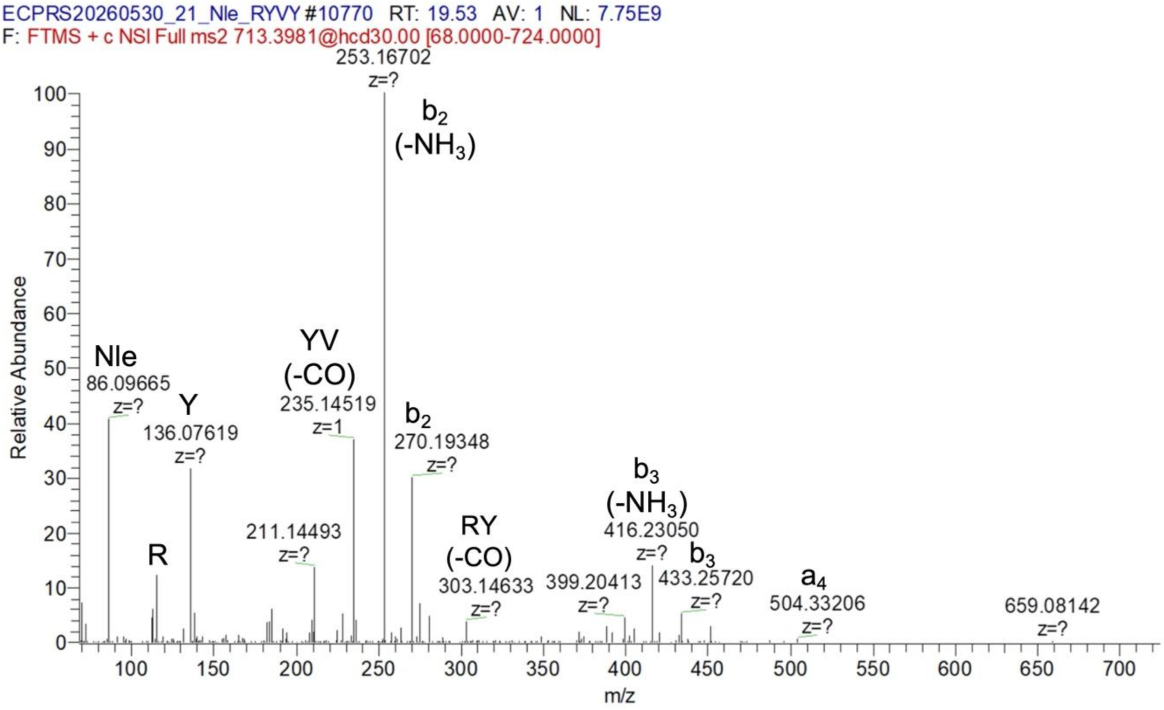

Figure S28. Annotated MS^2 spectrum of **Nle-3-(Val4)**.

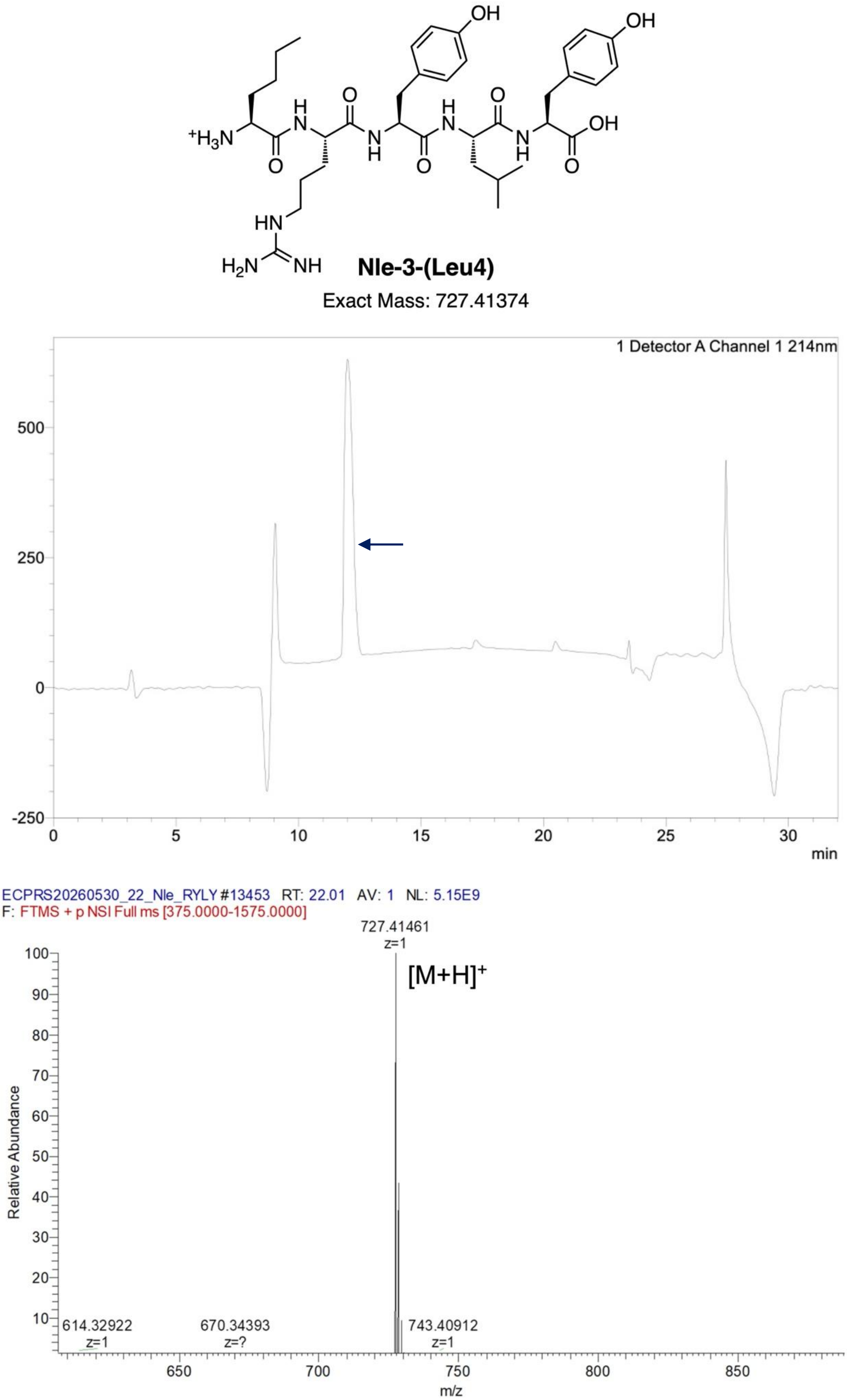

Figure S29. LCMS characterisation of **Nle-1-(Leu4)**. The arrow indicates the desired product peak.

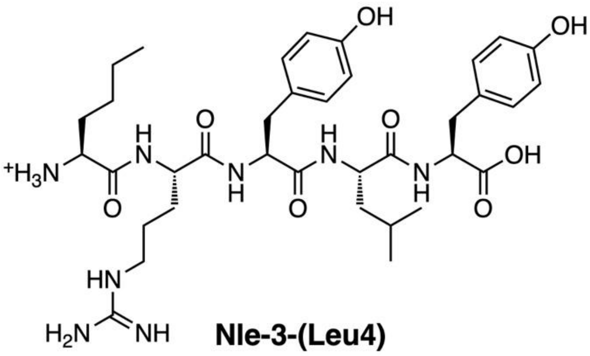

Nle-3-(Leu4)

Exact Mass: 727.41374

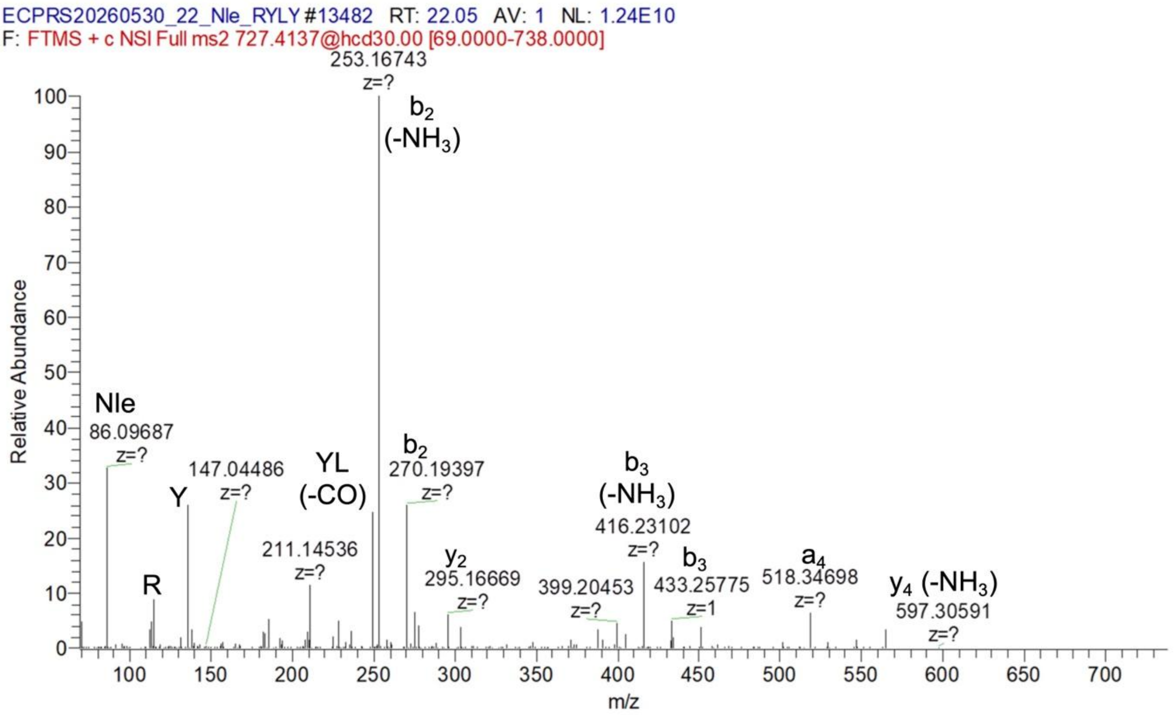

Figure S30. Annotated MS^2 spectrum of **Nle-3-(Leu4)**.

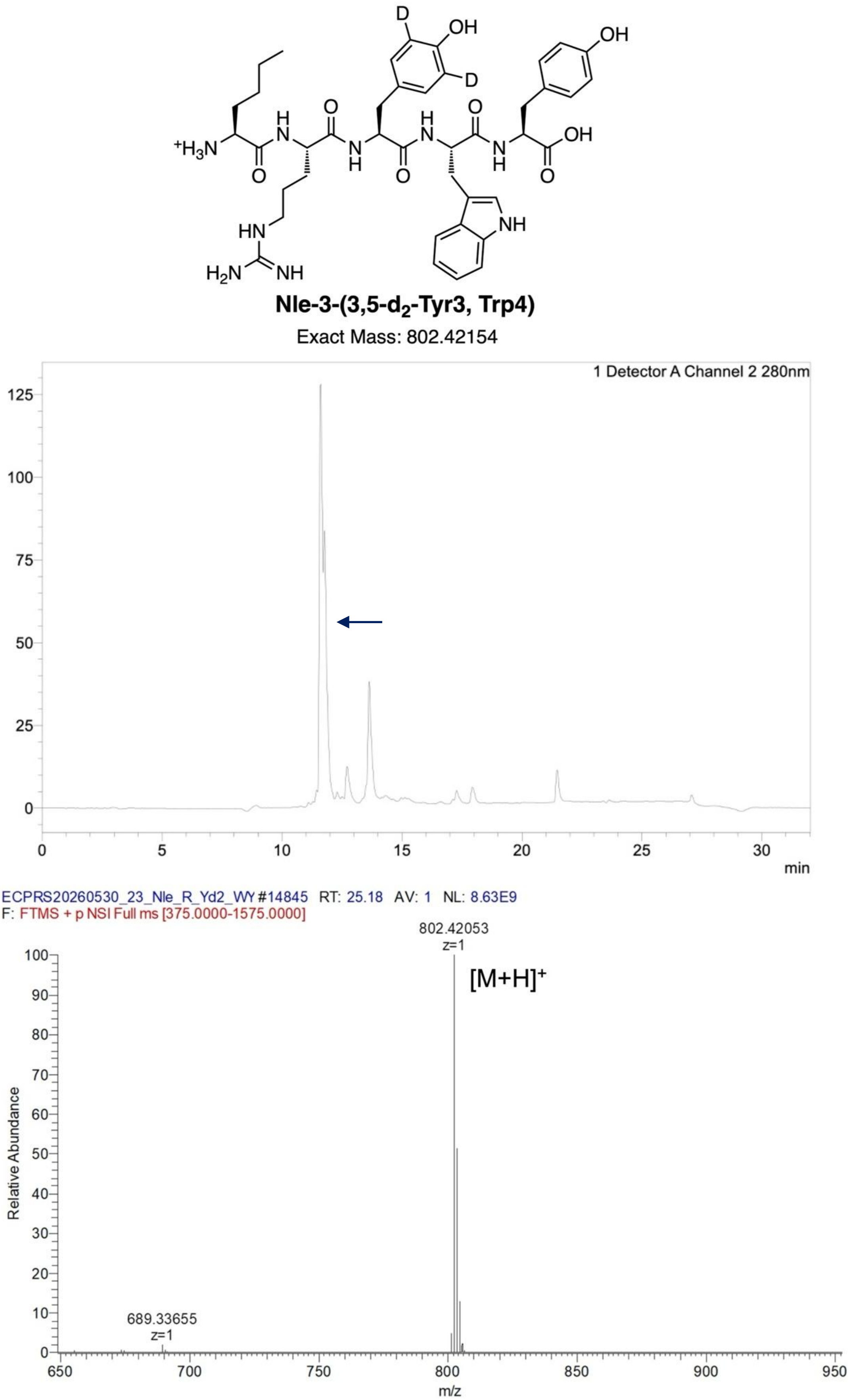

Figure S31. LCMS characterisation of **Nle-3-(3,5-d$_2$-Tyr3, Trp4)**. The arrow indicates the desired product peak.

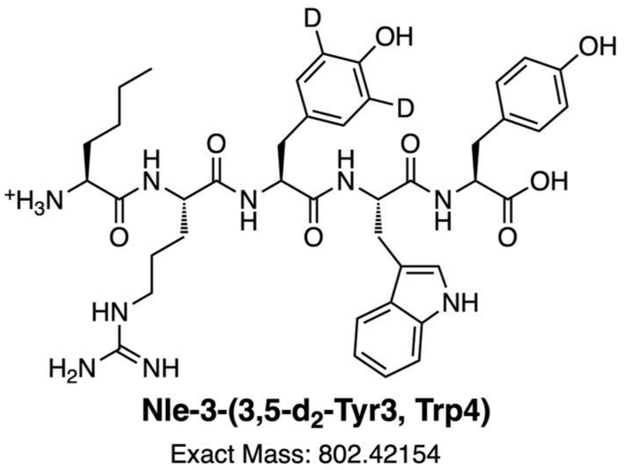

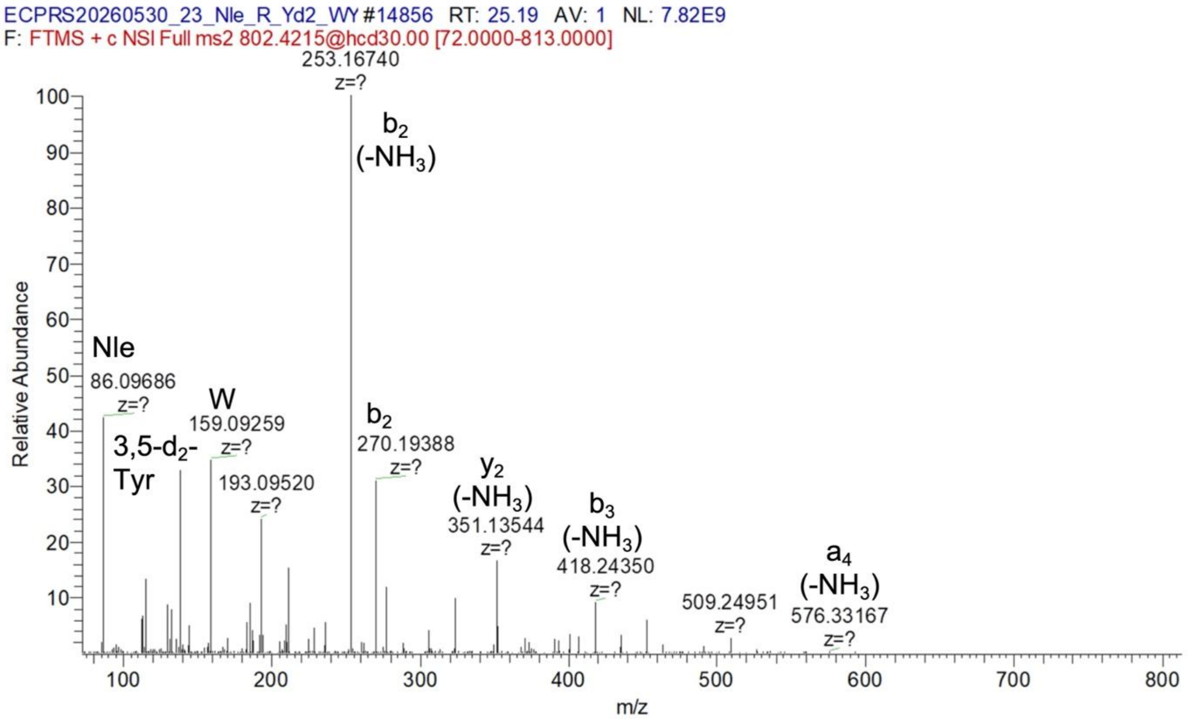

Figure S32. Annotated MS^2 spectrum of **Nle-3-(3,5-d$_2$-Tyr3, Trp4)**.

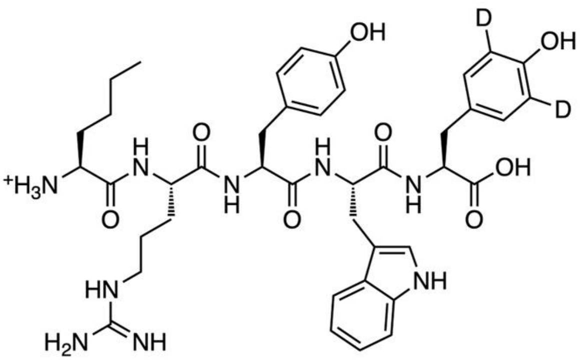

Nle-3-(Trp4, 3,5-d_2-Tyr5)

Exact Mass: 802.42154

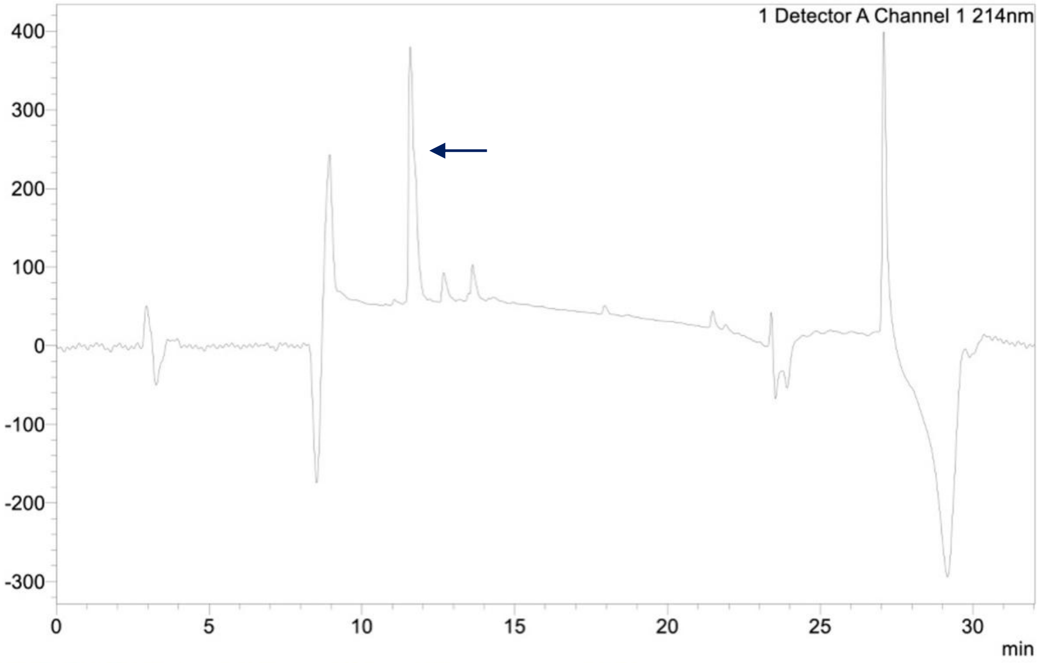

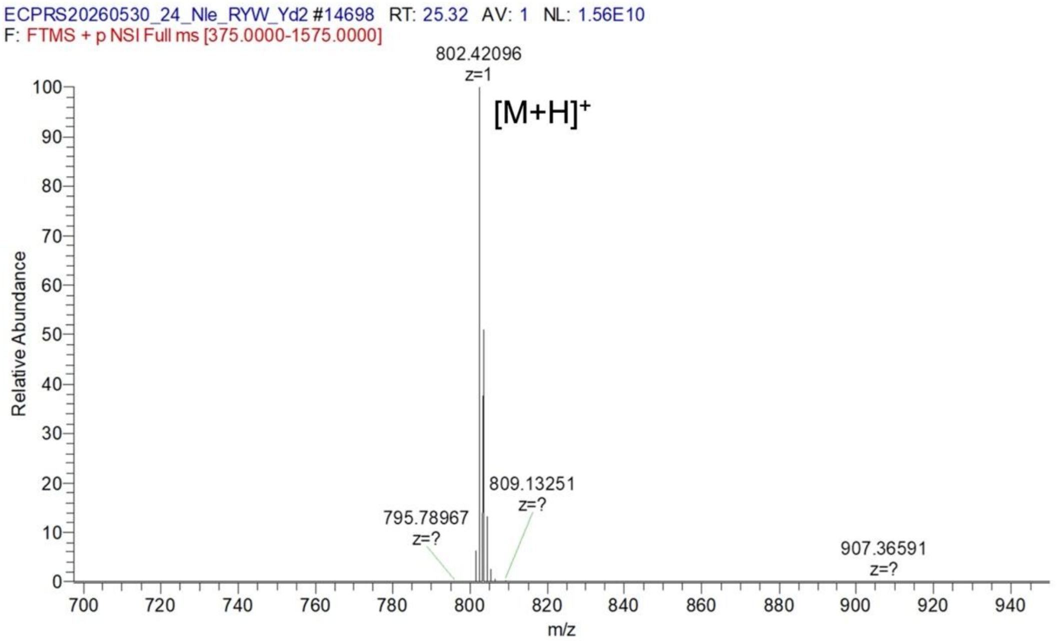

Figure S33. LCMS characterisation of **Nle-3-(Trp4, 3,5-d_2-Tyr5)**. The arrow indicates the desired product peak.

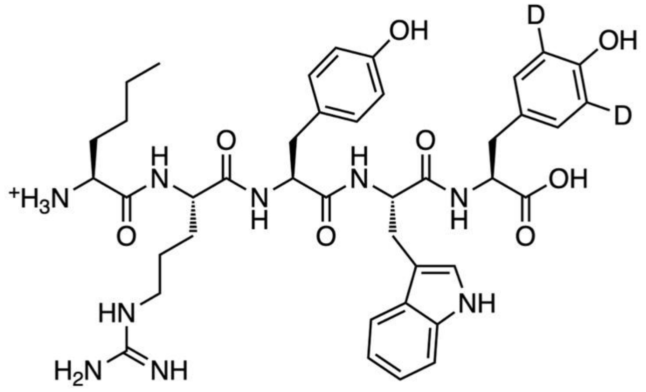

Nle-3-(Trp4, 3,5-d$_2$-Tyr5)

Exact Mass: 802.42154

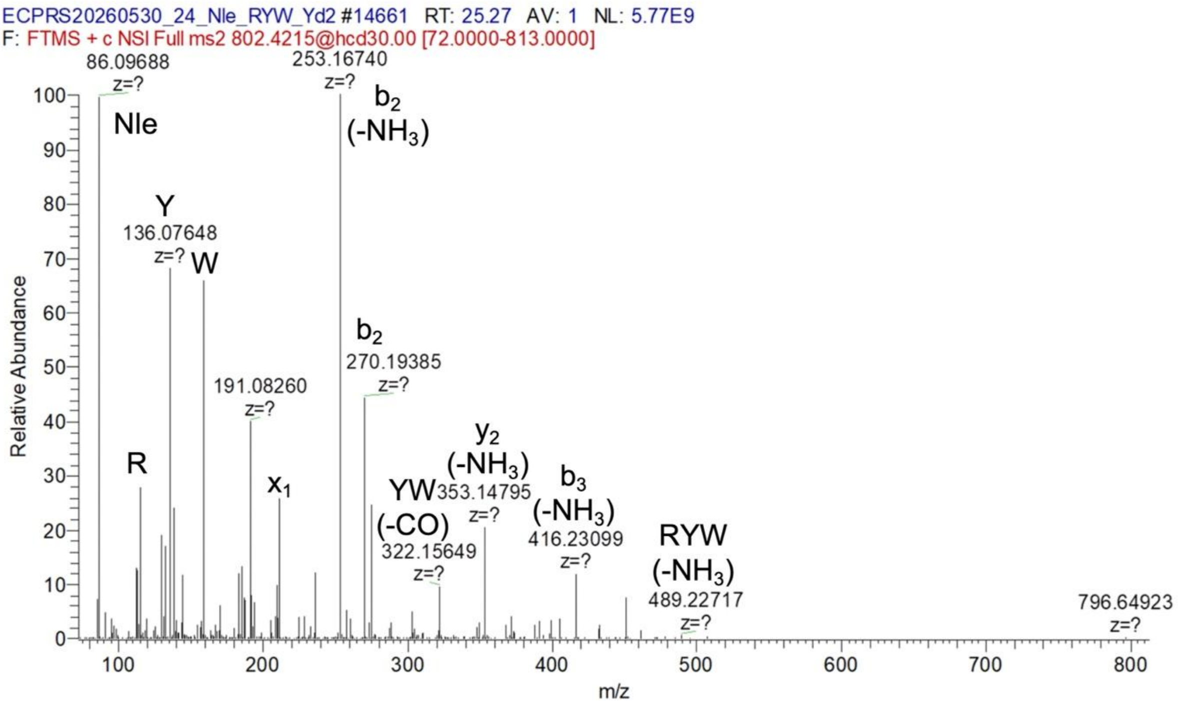

Figure S34. Annotated MS^2 spectrum of **Nle-3-(Trp4, 3,5-d$_2$-Tyr5)**.

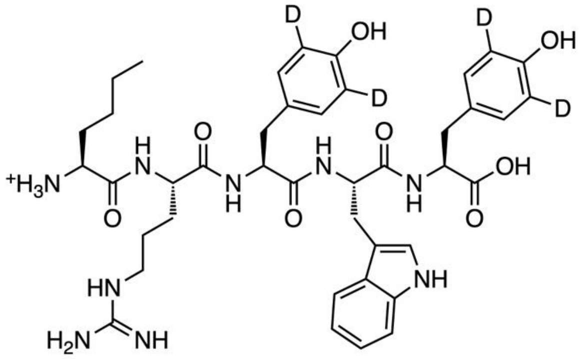

Nle-3-(Trp4, 3,5-d$_2$-Tyr3,5)

Exact Mass: 804.43409

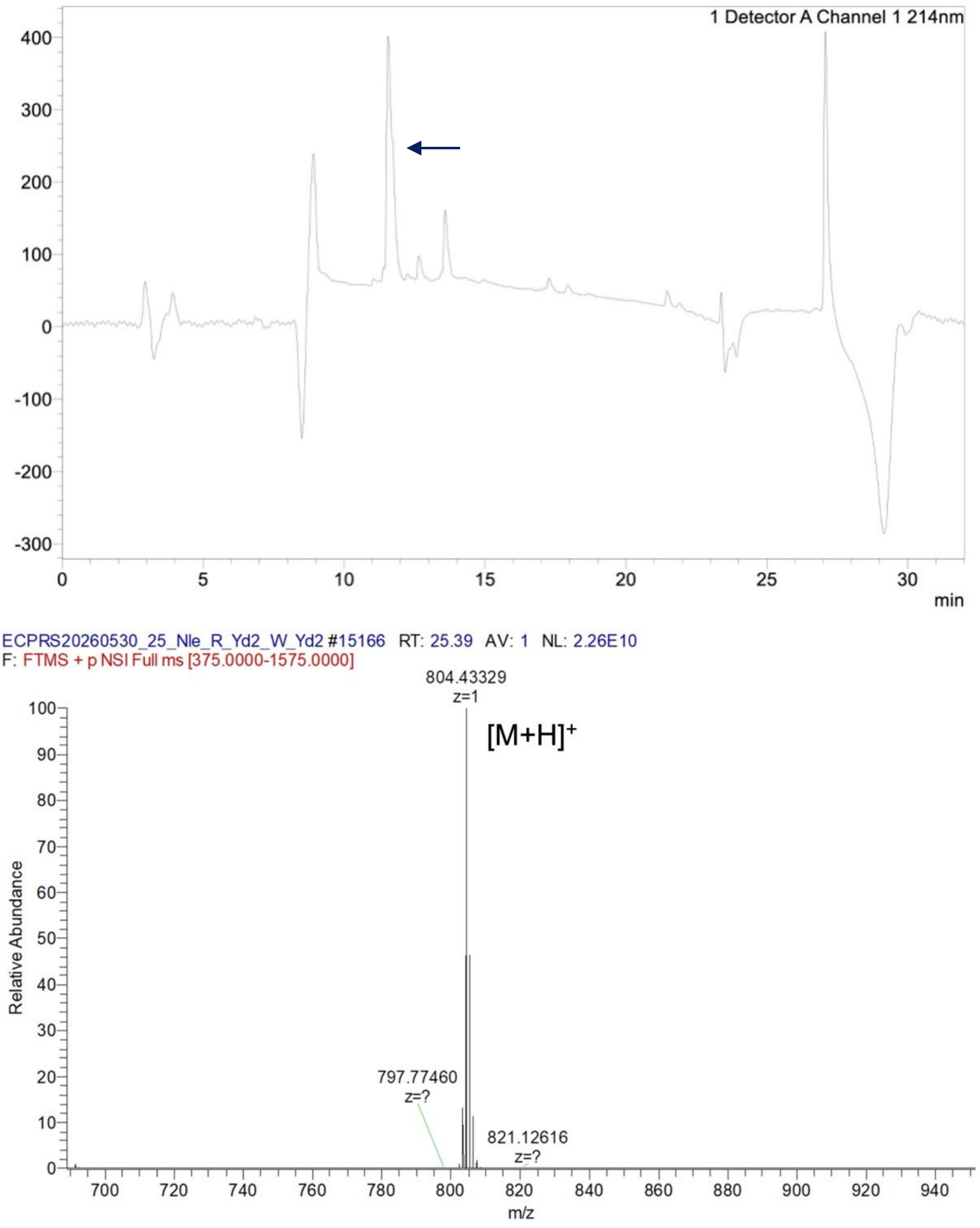

Figure S35. LCMS characterisation of **Nle-3-(Trp4, 3,5-d$_2$-Tyr3,5)**. The arrow indicates the desired product peak.

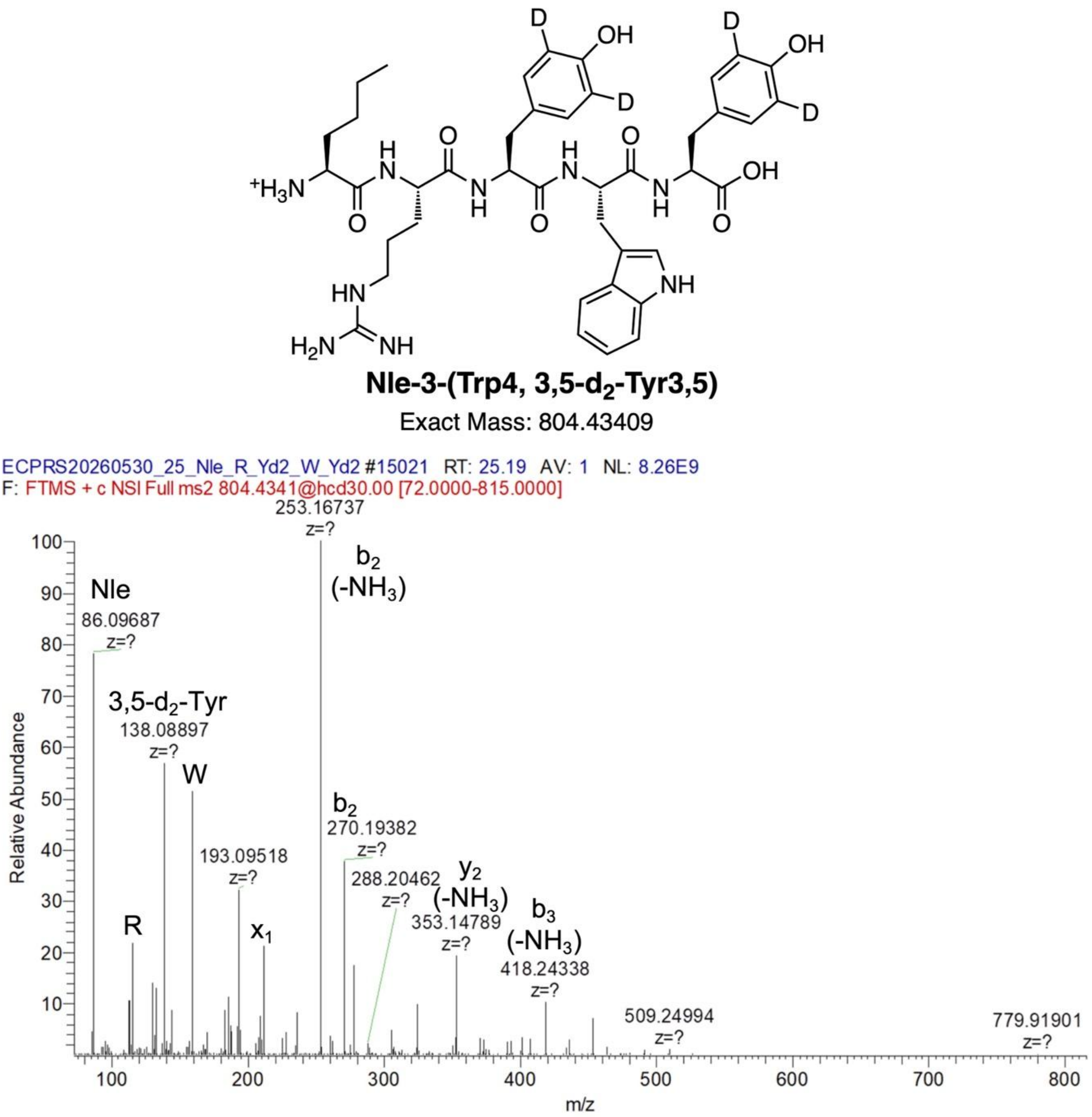

Figure S36. Annotated MS^2 spectrum of **Nle-3-(Trp4, 3,5-d$_2$-Tyr3,5)**.

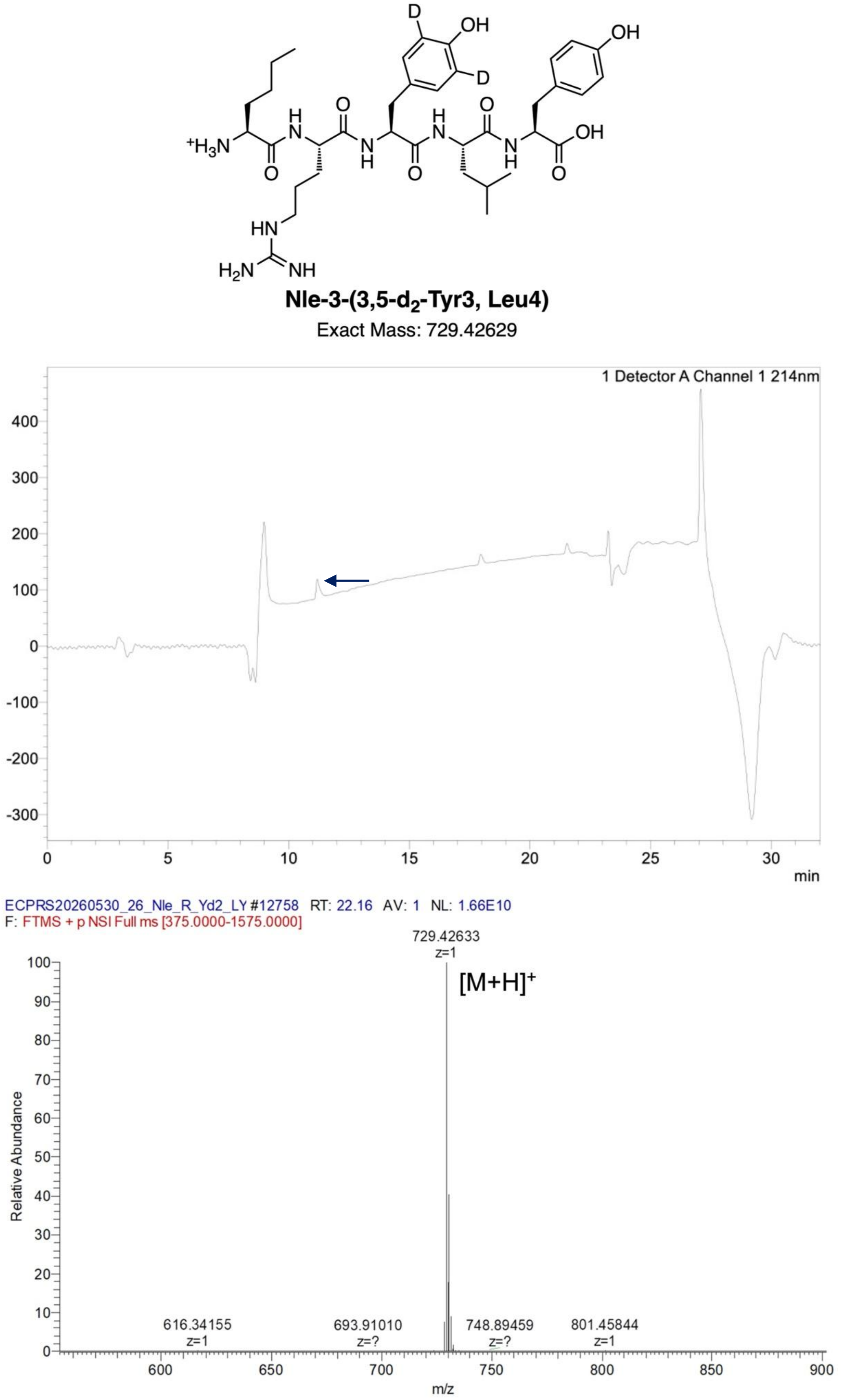

Figure S37. LCMS characterisation of **Nle-3-(3,5-d$_2$-Tyr3, Leu4)**. The arrow indicates the desired product peak.

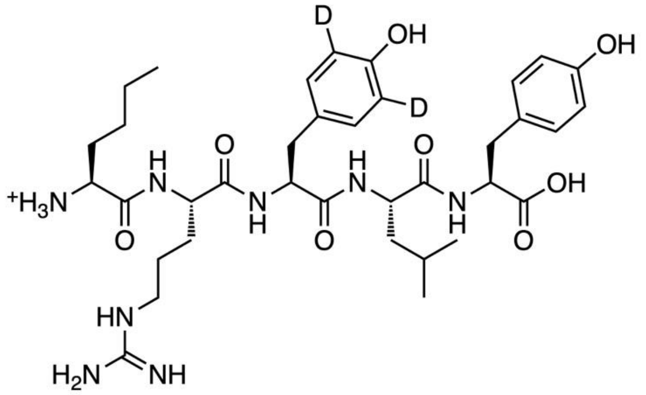

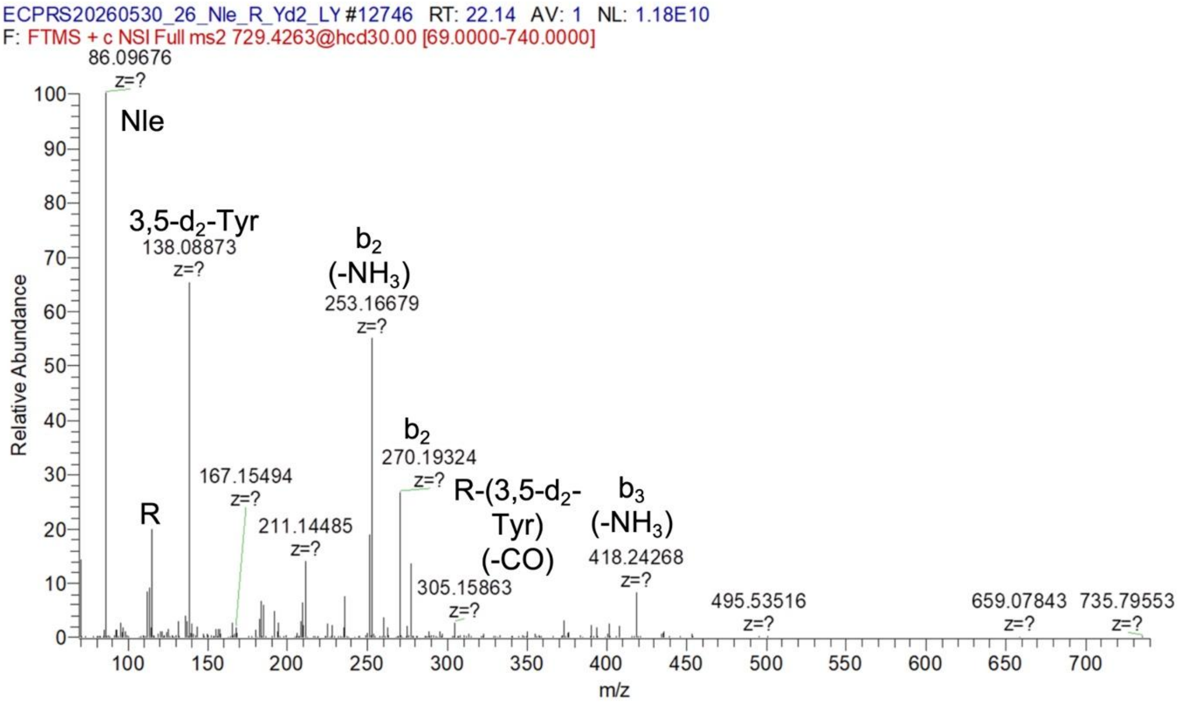

Figure S38. Annotated MS^2 spectrum of **Nle-3-(3,5-d$_2$-Tyr3, Leu4)**.

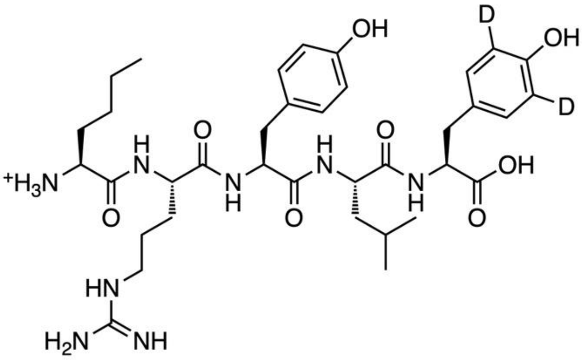

Nle-3-(Leu4, 3,5-d$_2$-Tyr5)

Exact Mass: 729.42629

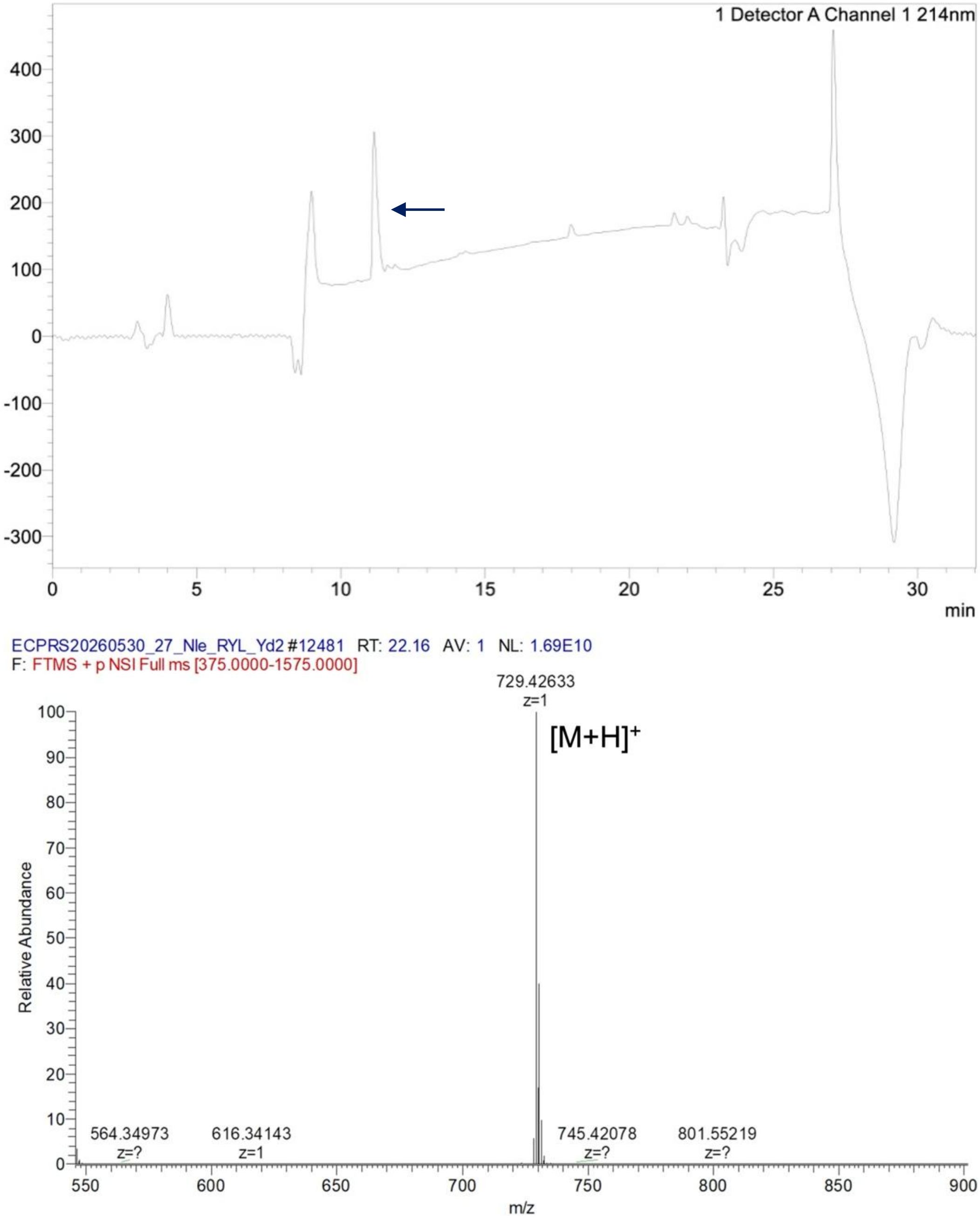

Figure S39. LCMS characterisation of **Nle-3-(Leu4, 3,5-d$_2$-Tyr5)**. The arrow indicates the desired product peak.

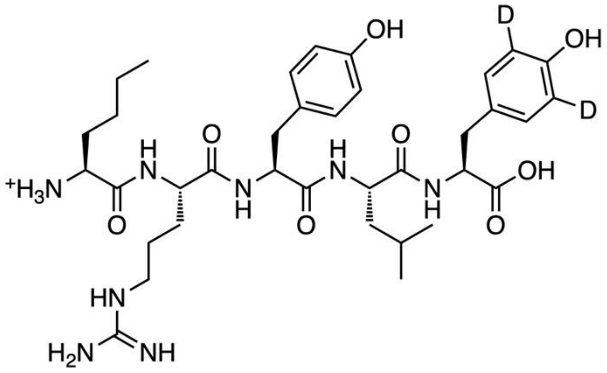

Nle-3-(Leu4, 3,5-d$_2$-Tyr5)

Exact Mass: 729.42629

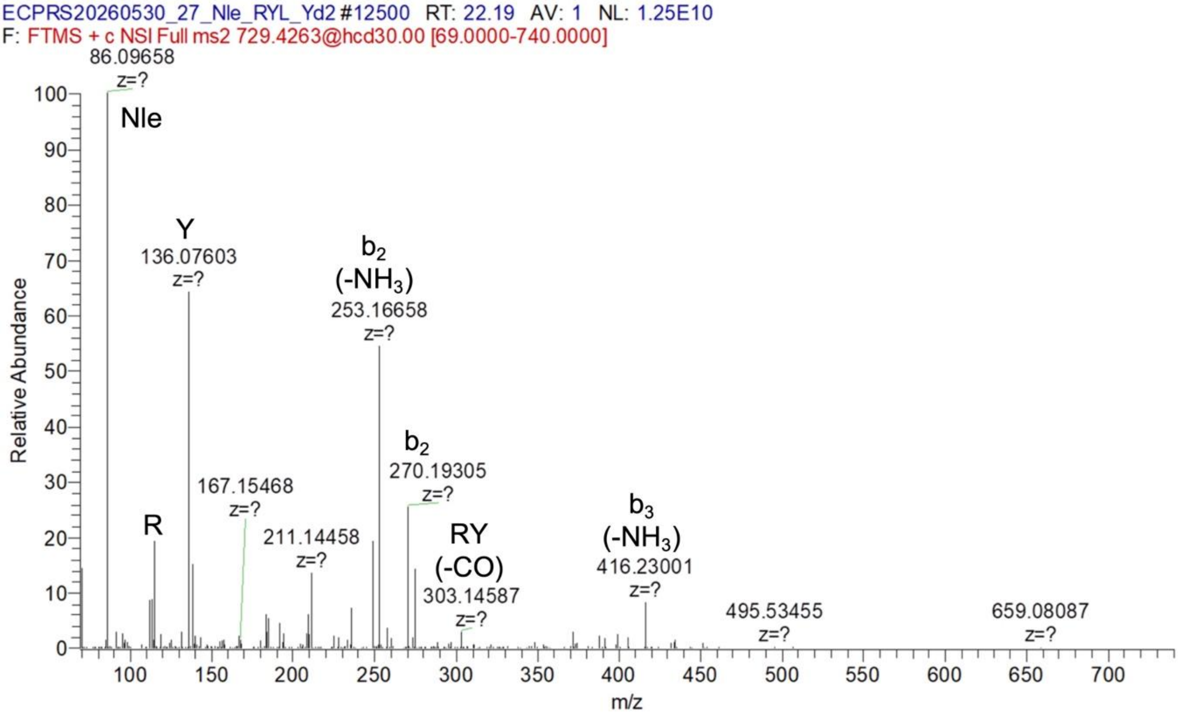

Figure S40. Annotated MS^2 spectrum of **Nle-3-(Leu4, 3,5-d$_2$-Tyr5)**.

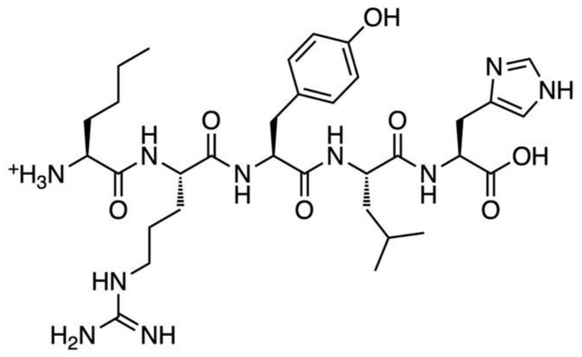

Nle-3-(Leu4, His5)

Exact Mass: 701.40932

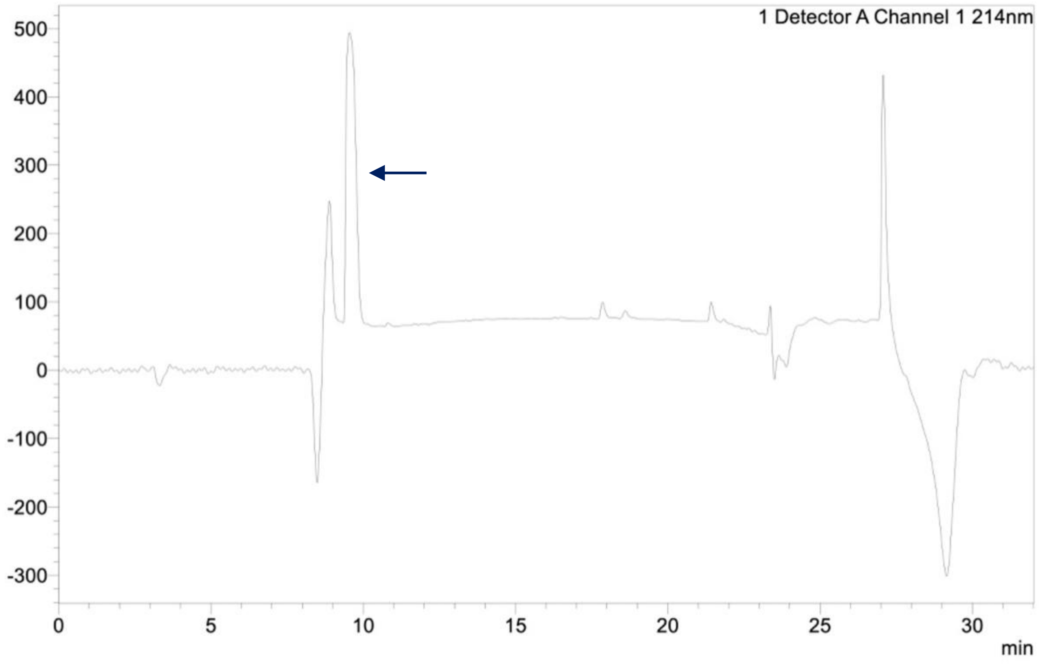

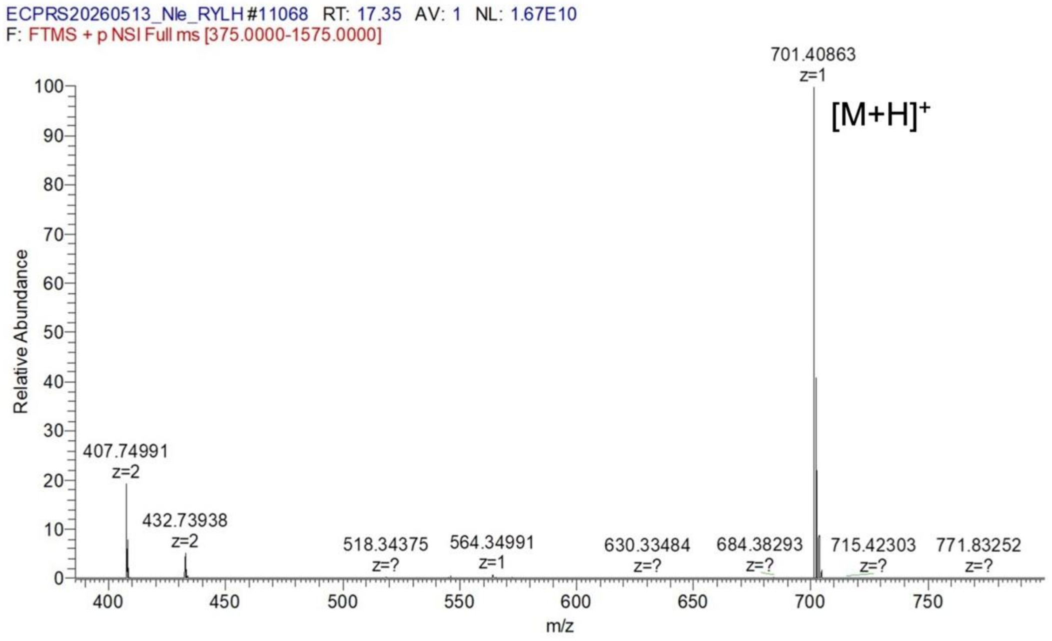

Figure S41. LCMS characterisation of **Nle-3-(Leu4, His5)**. The arrow indicates the desired product peak.

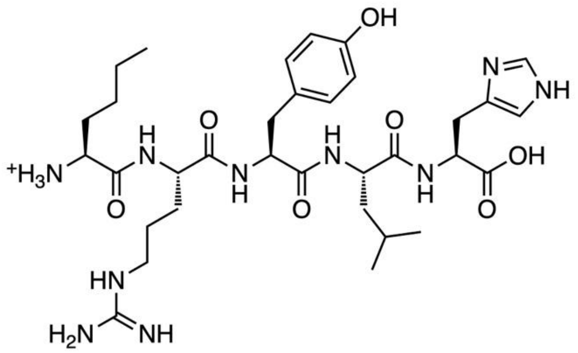

Nle-3-(Leu4, His5)

Exact Mass: 701.40932

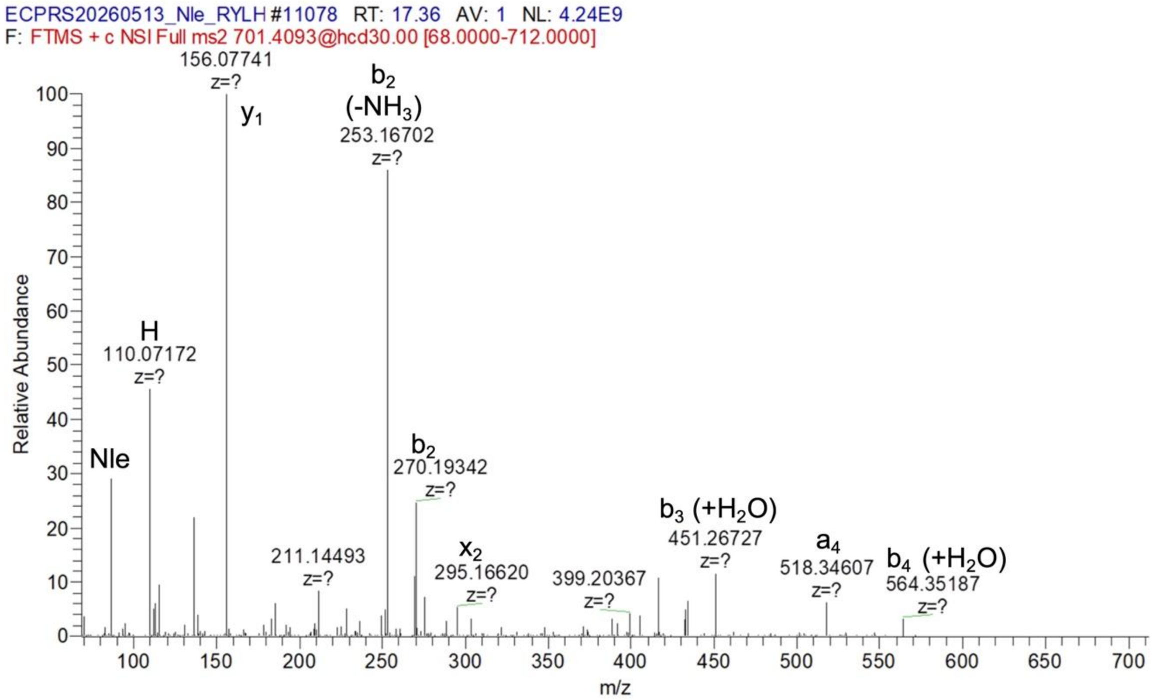

Figure S42. Annotated MS^2 spectrum of **Nle-3-(Leu4, His5)**.

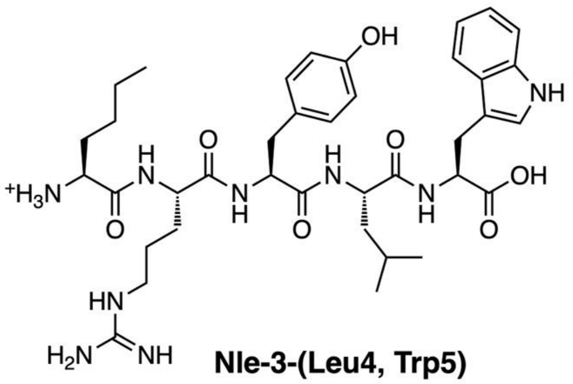

Nle-3-(Leu4, Trp5)
Exact Mass: 750.42972

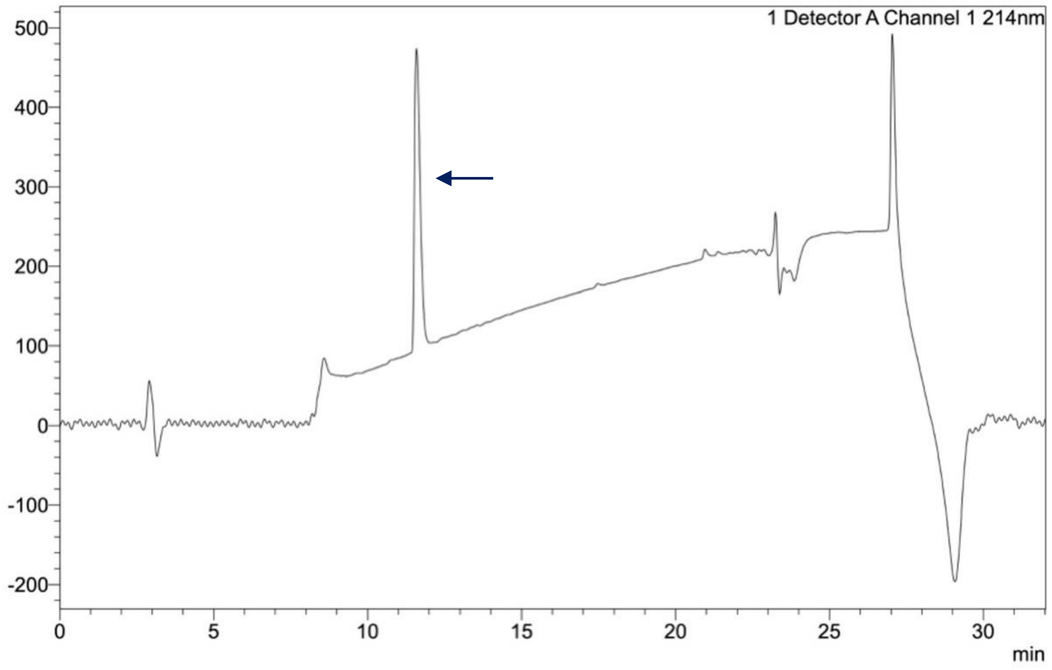

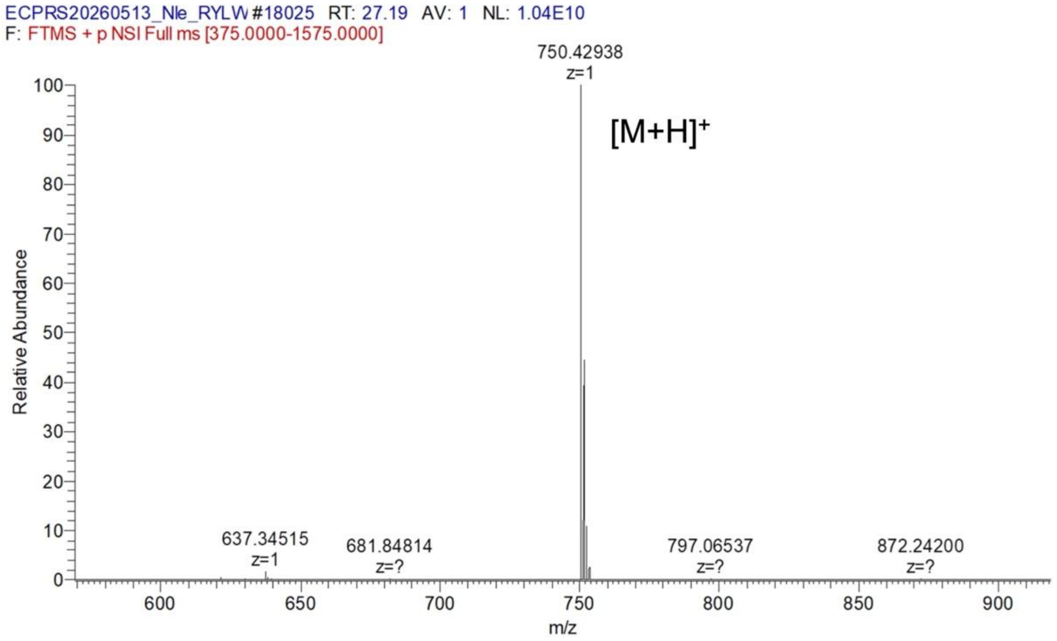

Figure S43. LCMS characterisation of **Nle-3-(Leu4, Trp5)**. The arrow indicates the desired product peak.

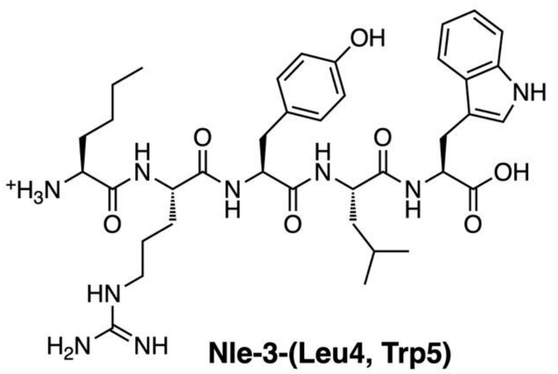

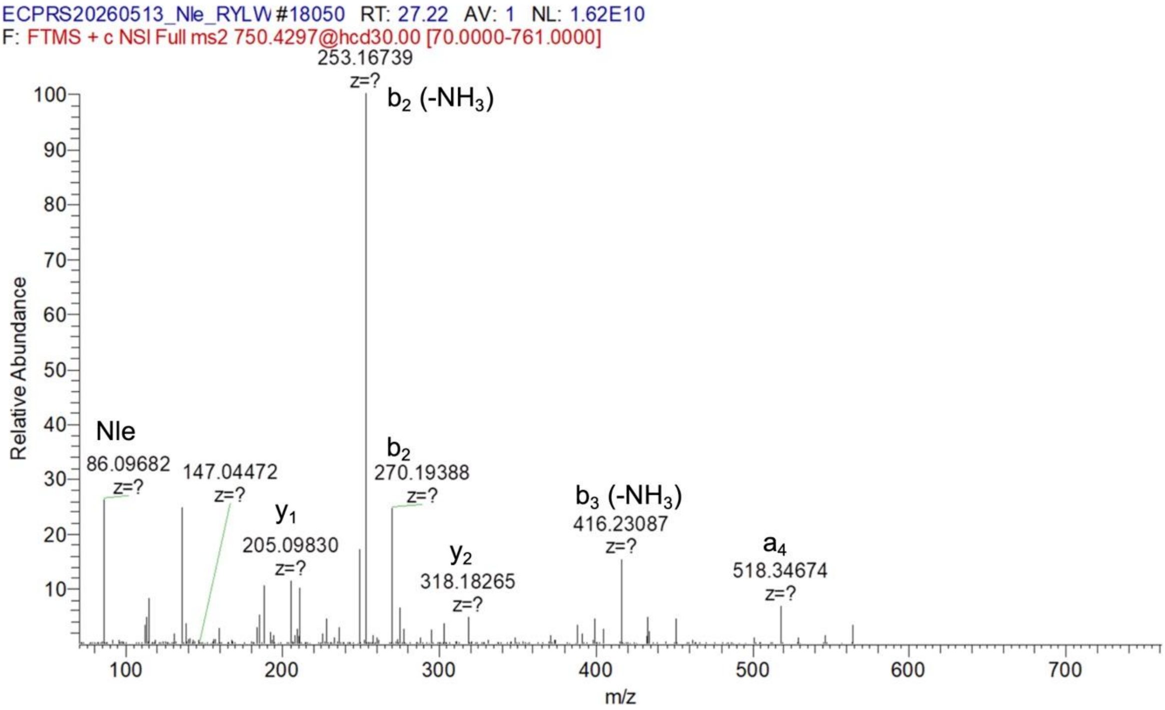

Figure S44. Annotated MS^2 spectrum of **Nle-3-(Leu4, Trp5)**.

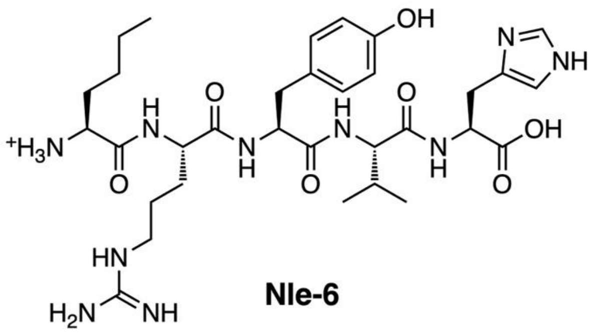

Nle-6

Exact Mass: 687.39367

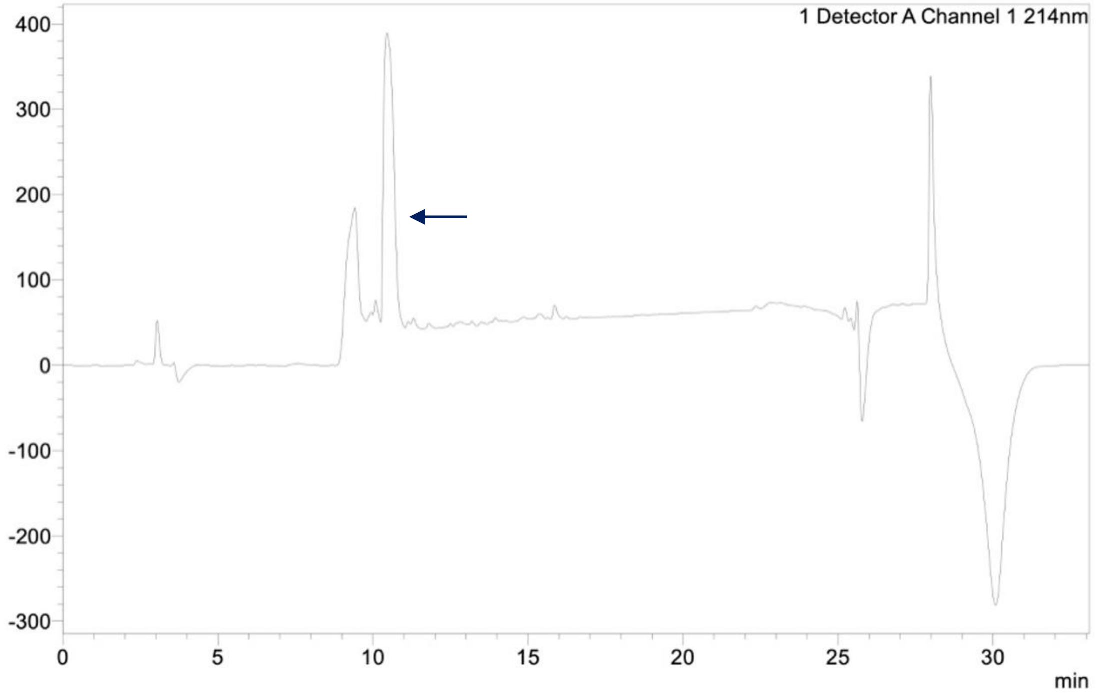

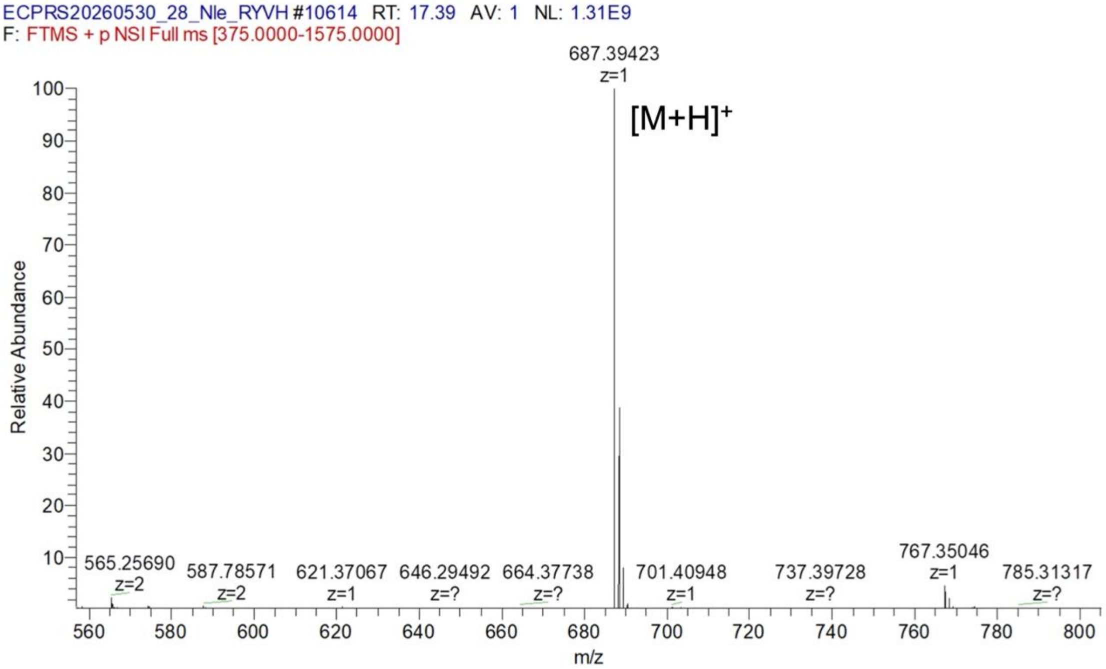

Figure S45. LCMS characterisation of **Nle-6**. The arrow indicates the desired product peak.

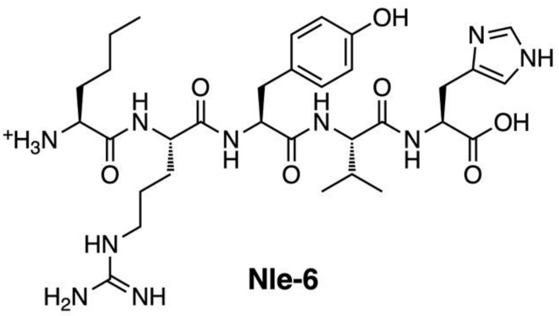

Nle-6

Exact Mass: 687.39367

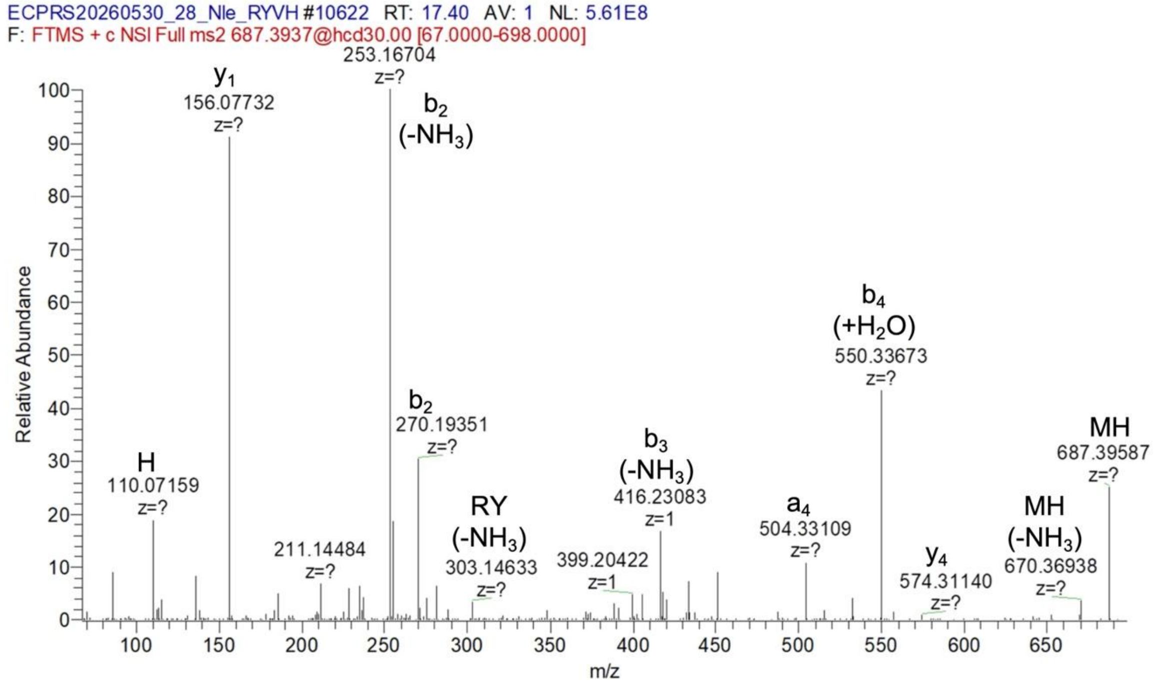

Figure S46. Annotated MS^2 spectrum of **Nle-6**.

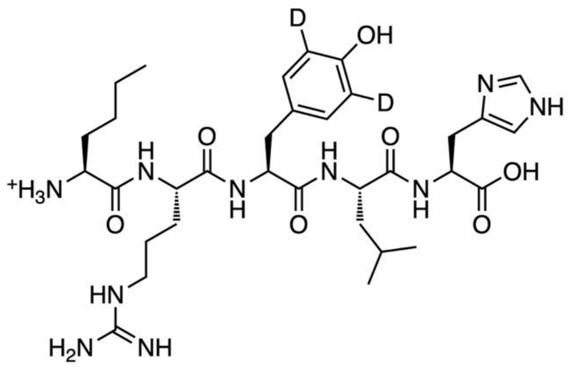

Nle-6-(3,5-d$_2$-Tyr3, Leu4)

Exact Mass: 703.42187

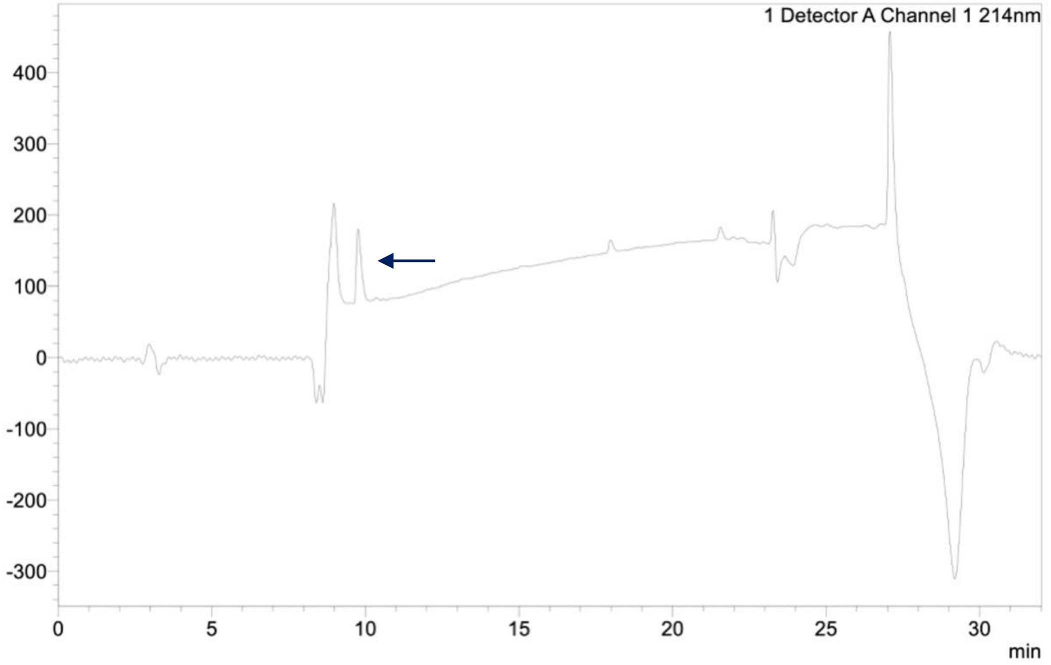

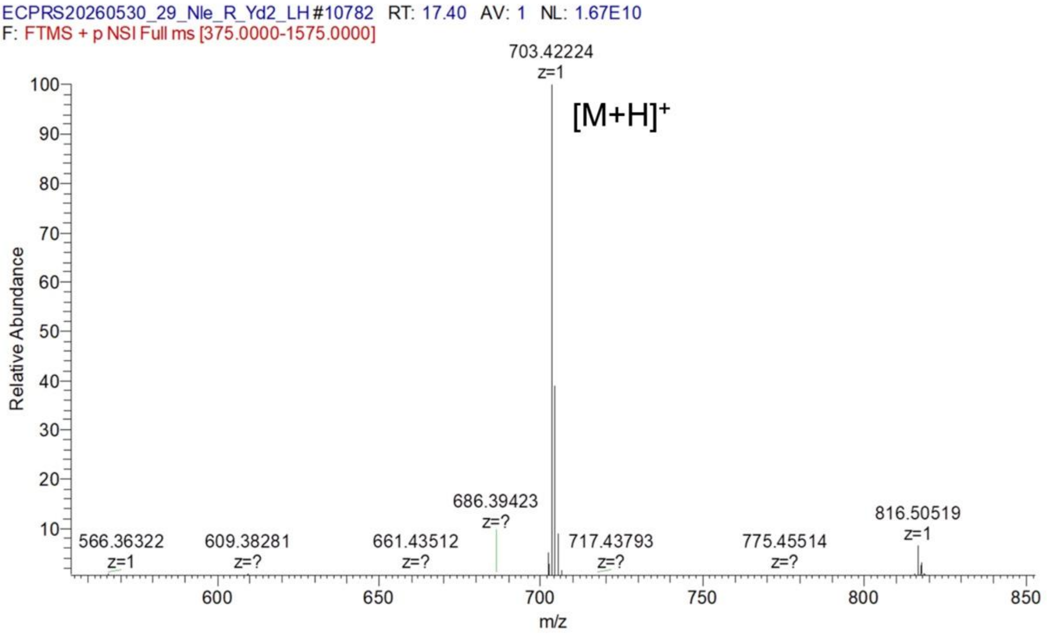

Figure S47. LCMS characterisation of **Nle-6-(3,5-d$_2$-Tyr3, Leu4)**. The arrow indicates the desired product peak.

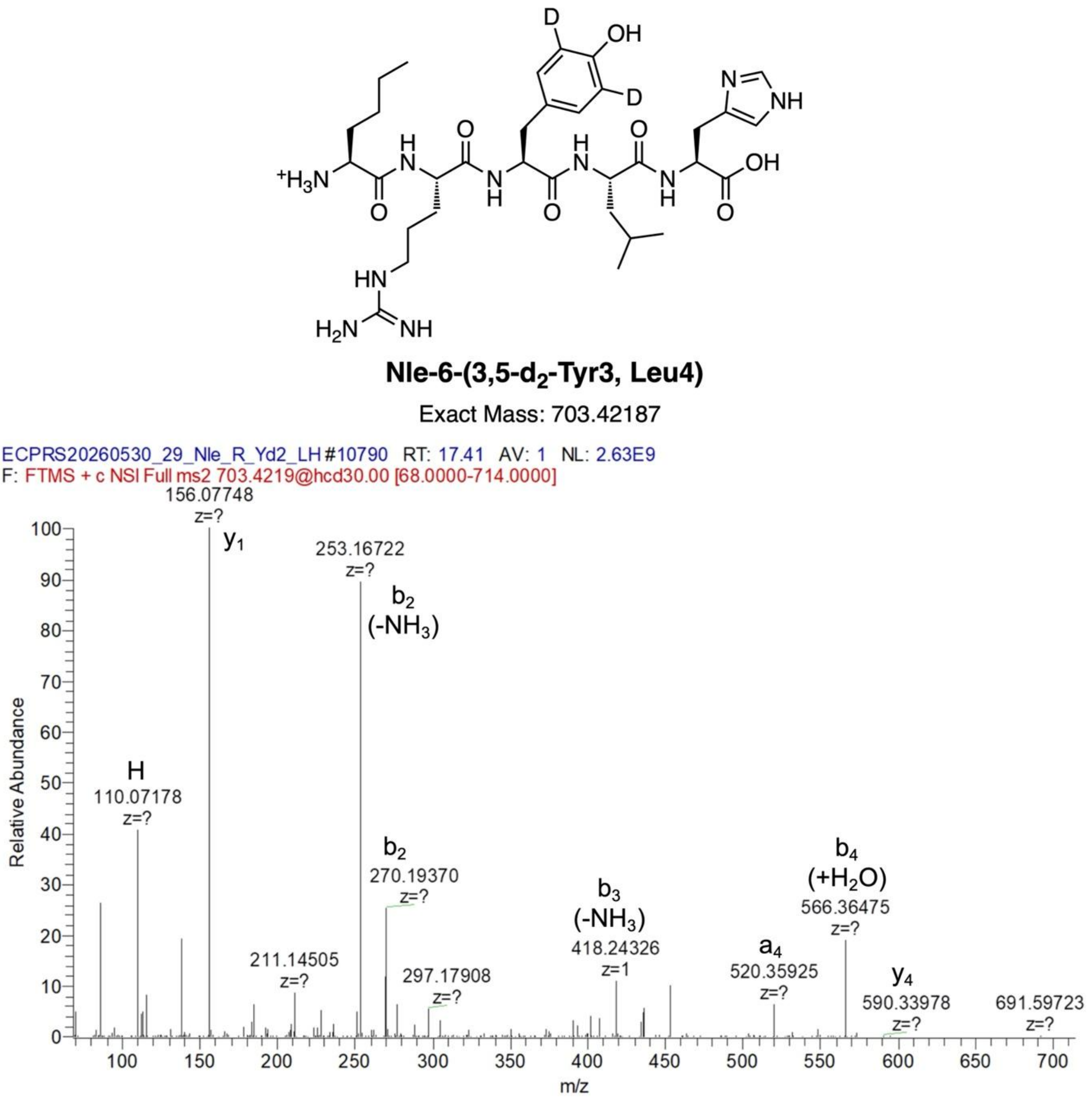

Figure S48. Annotated MS^2 spectrum of **Nle-6-(3,5-d$_2$-Tyr3, Leu4)**.

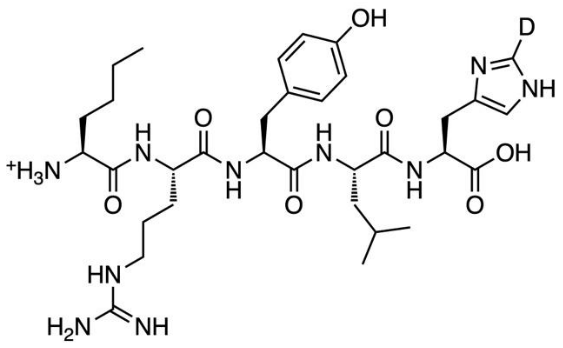

Nle-6-(Leu4, 2-d-His5)

Exact Mass: 702.41560

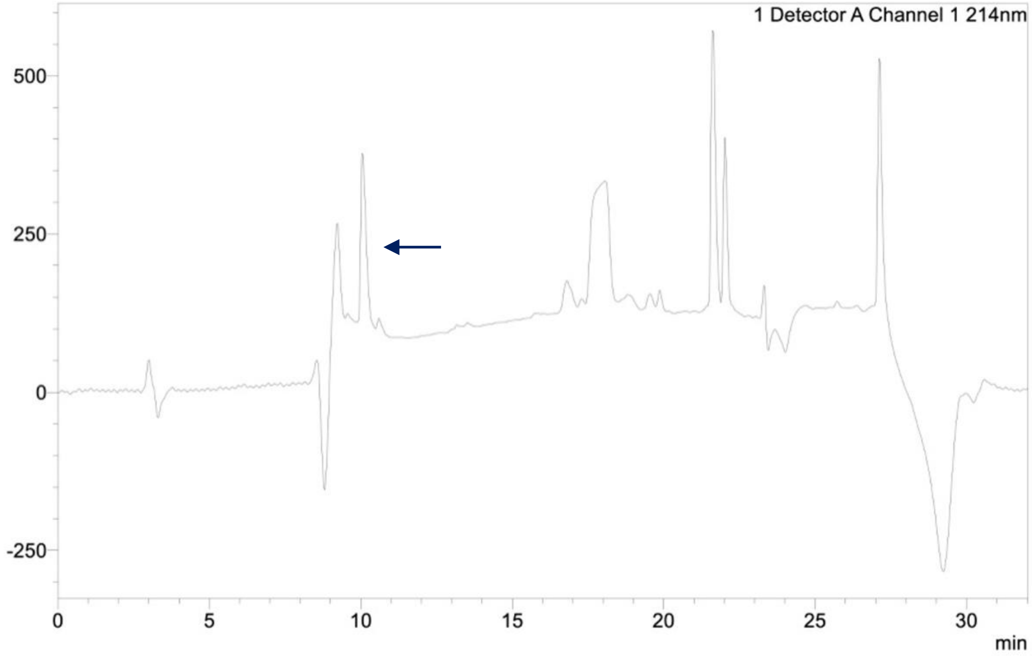

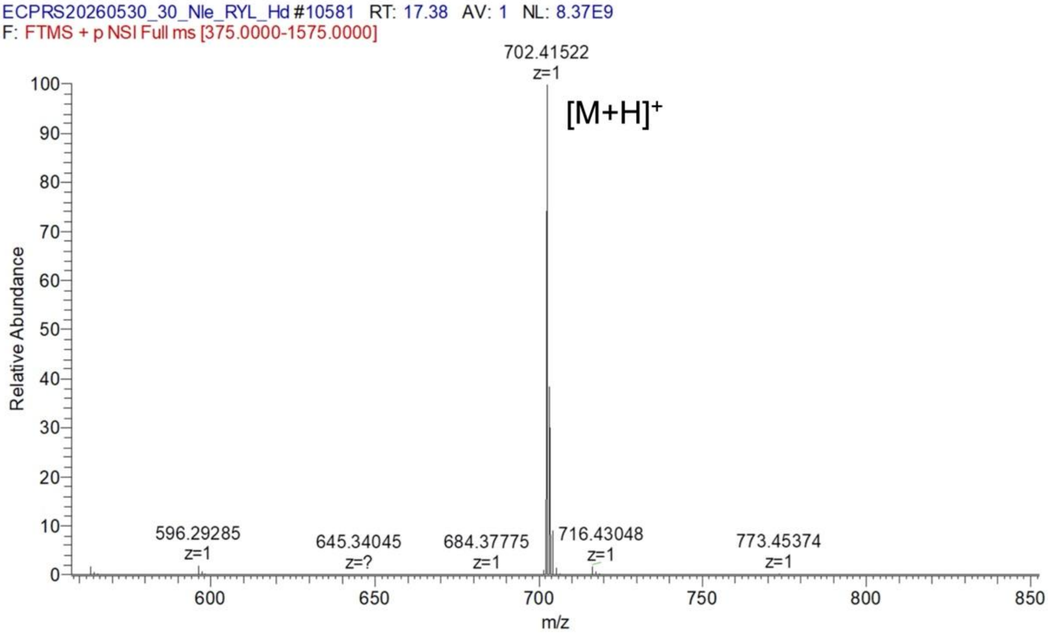

Figure S49. LCMS characterisation of **Nle-6-(Leu4, 2-d-His5)**. The arrow indicates the desired product peak.

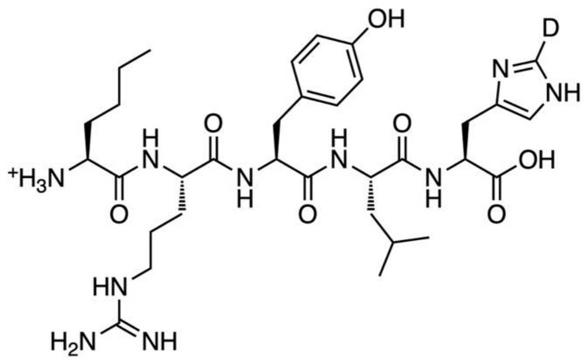

Nle-6-(Leu4, 2-d-His5)

Exact Mass: 702.41560

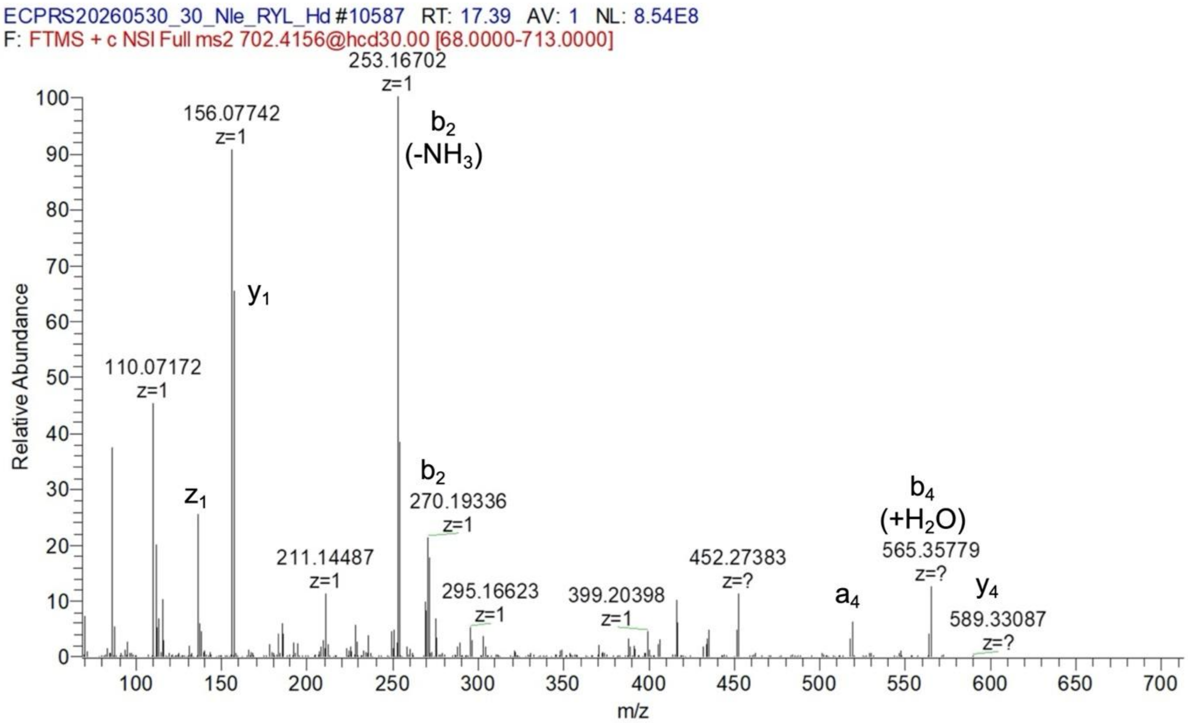

Figure S50. Annotated MS^2 spectrum of **Nle-6-(Leu4, 2-d-His5)**.

5. NMR characterisation of peptides

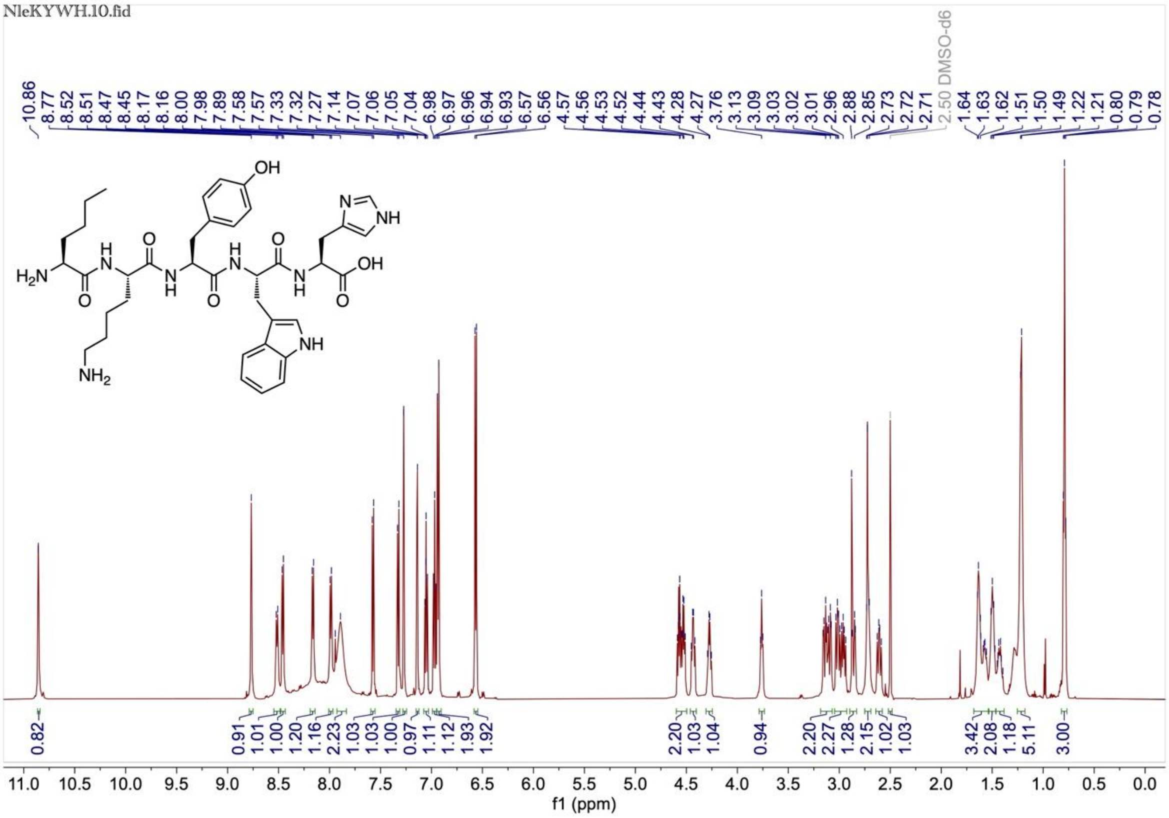

Figure S51. ^{1}H NMR spectrum of **Nle-1**.

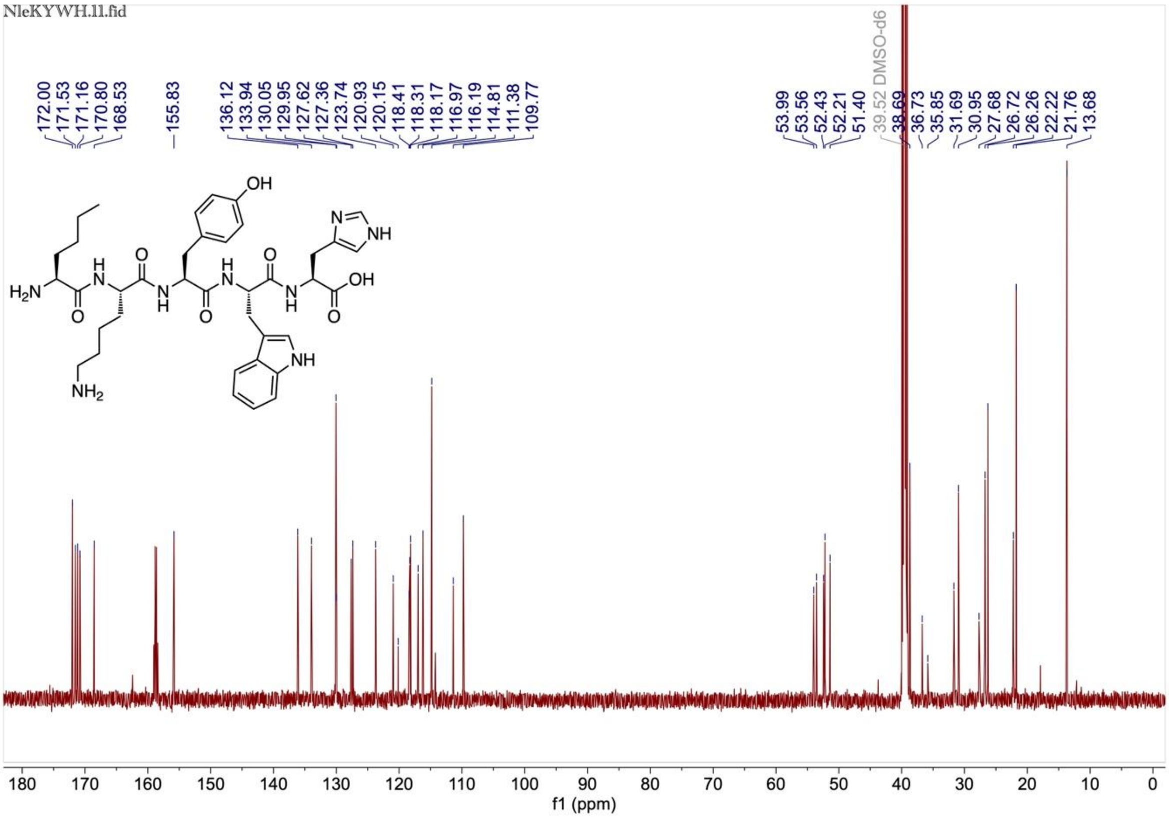

Figure S52. ^{13}C NMR spectrum of **Nle-1**.

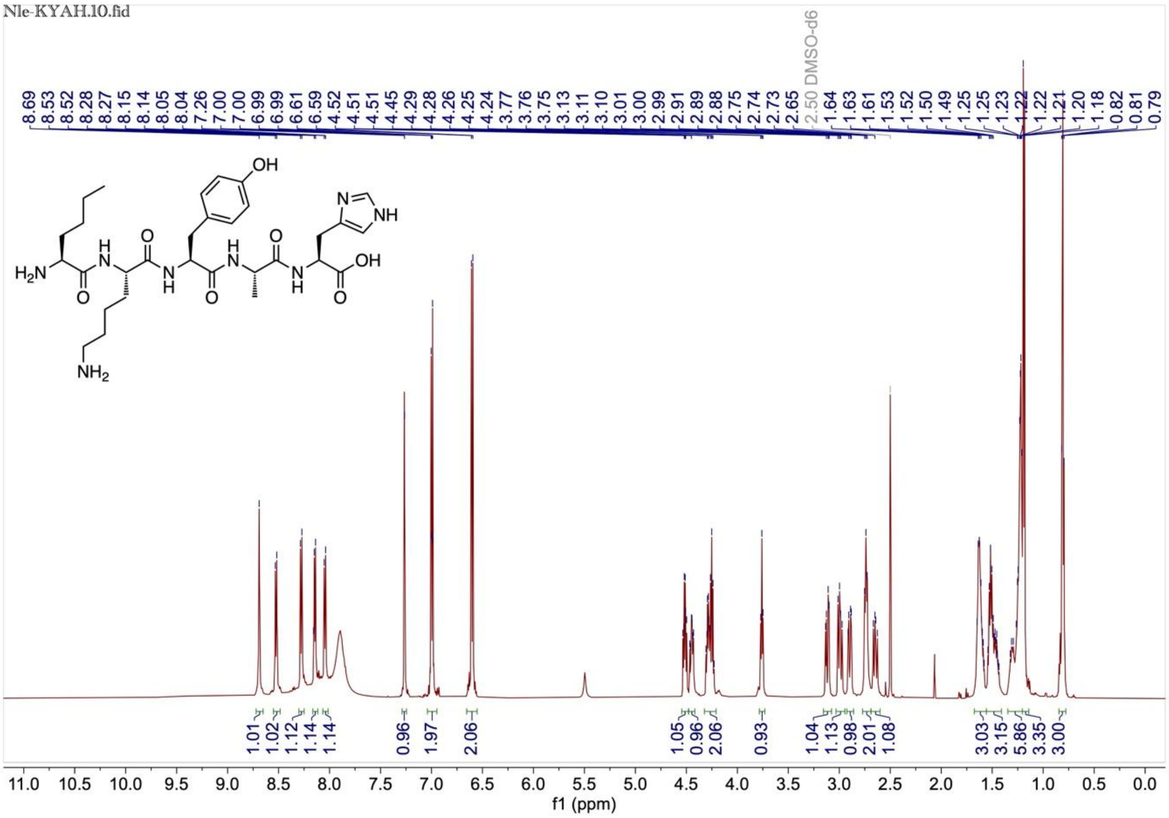

Figure S53. ^{1}H NMR spectrum of **Nle-1-(Ala4)**.

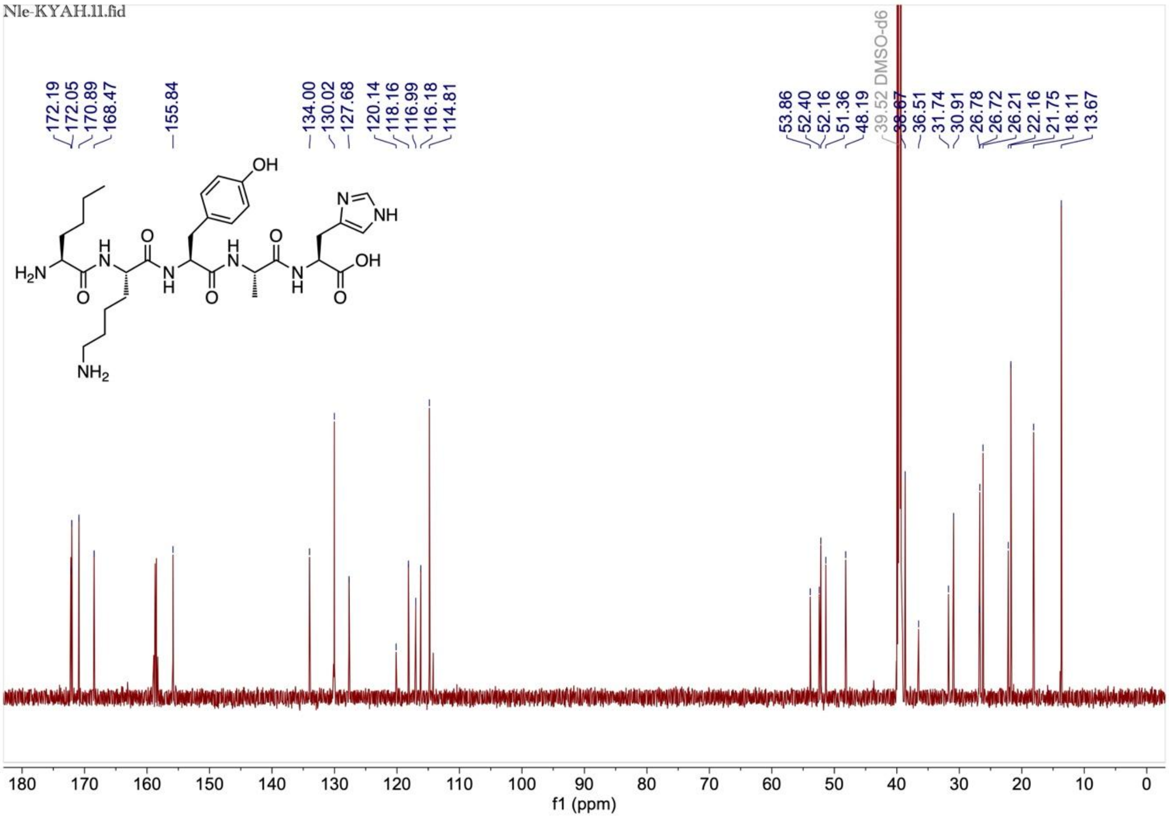

Figure S54. ^{13}C NMR spectrum of **Nle-1-(Ala4)**.

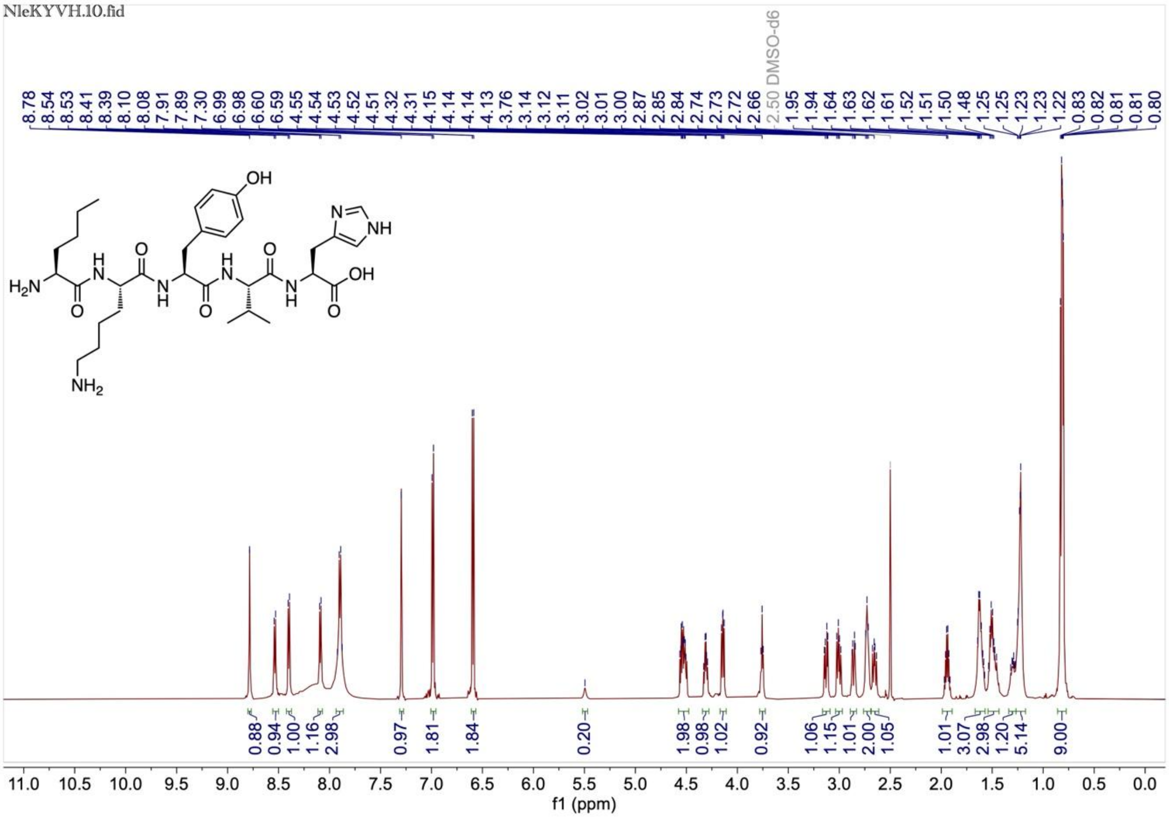

Figure S55. ^{1}H NMR spectrum of **Nle-1-(Val4)**.

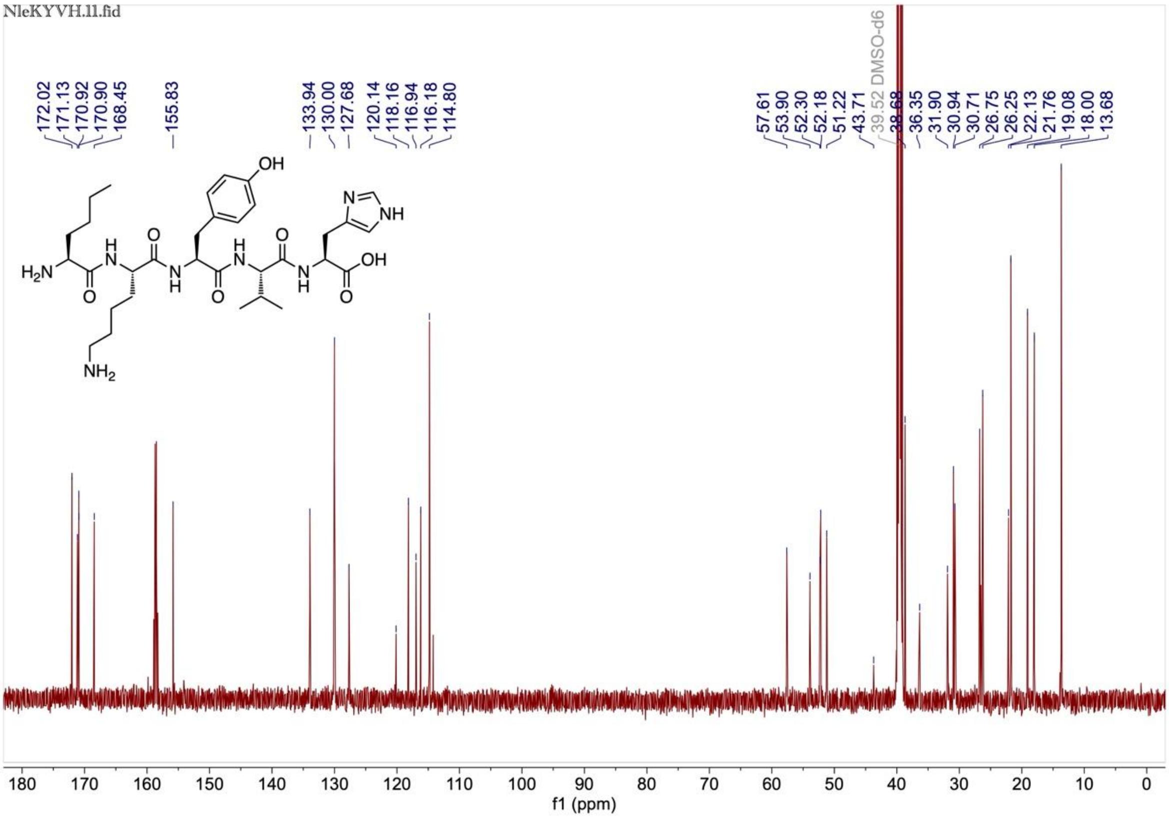

Figure S56. ^{13}C NMR spectrum of **Nle-1-(Val4)**.

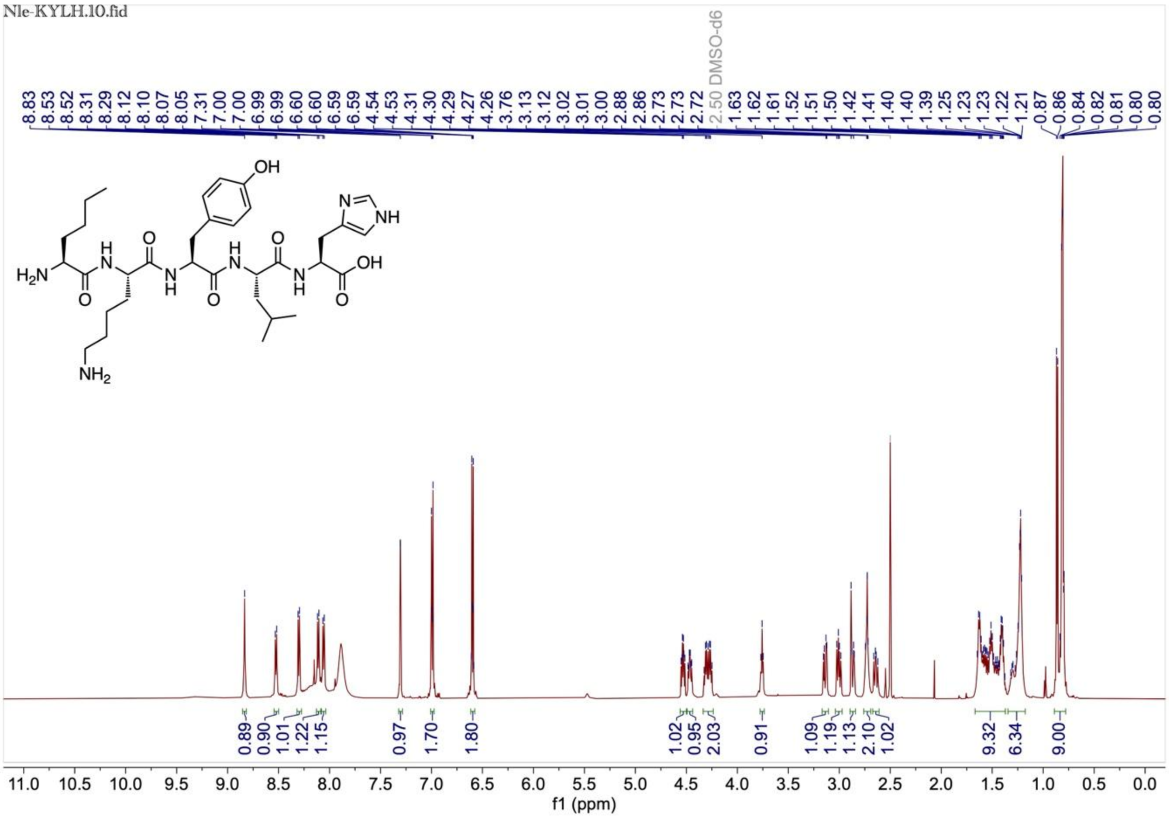

Figure S57. ^{1}H NMR spectrum of **Nle-1-(Leu4)**.

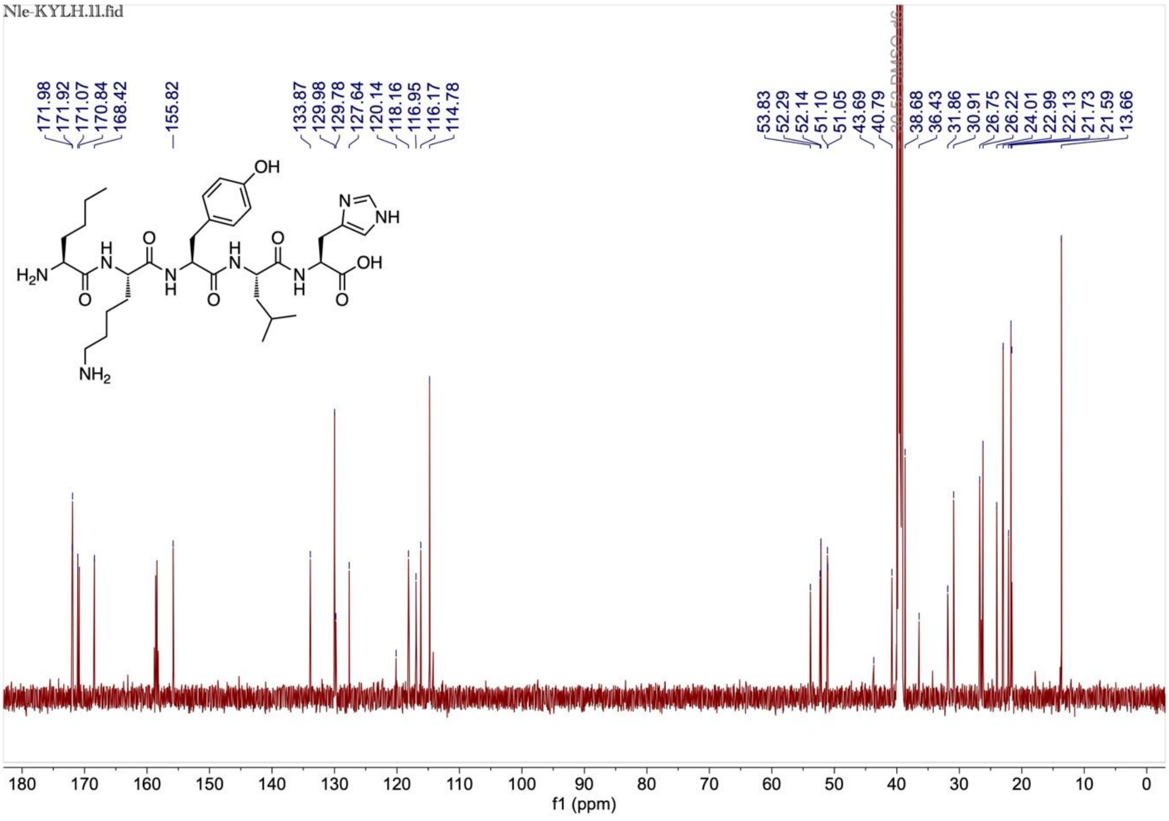

Figure S58. ^{13}C NMR spectrum of **Nle-1-(Leu4)**.

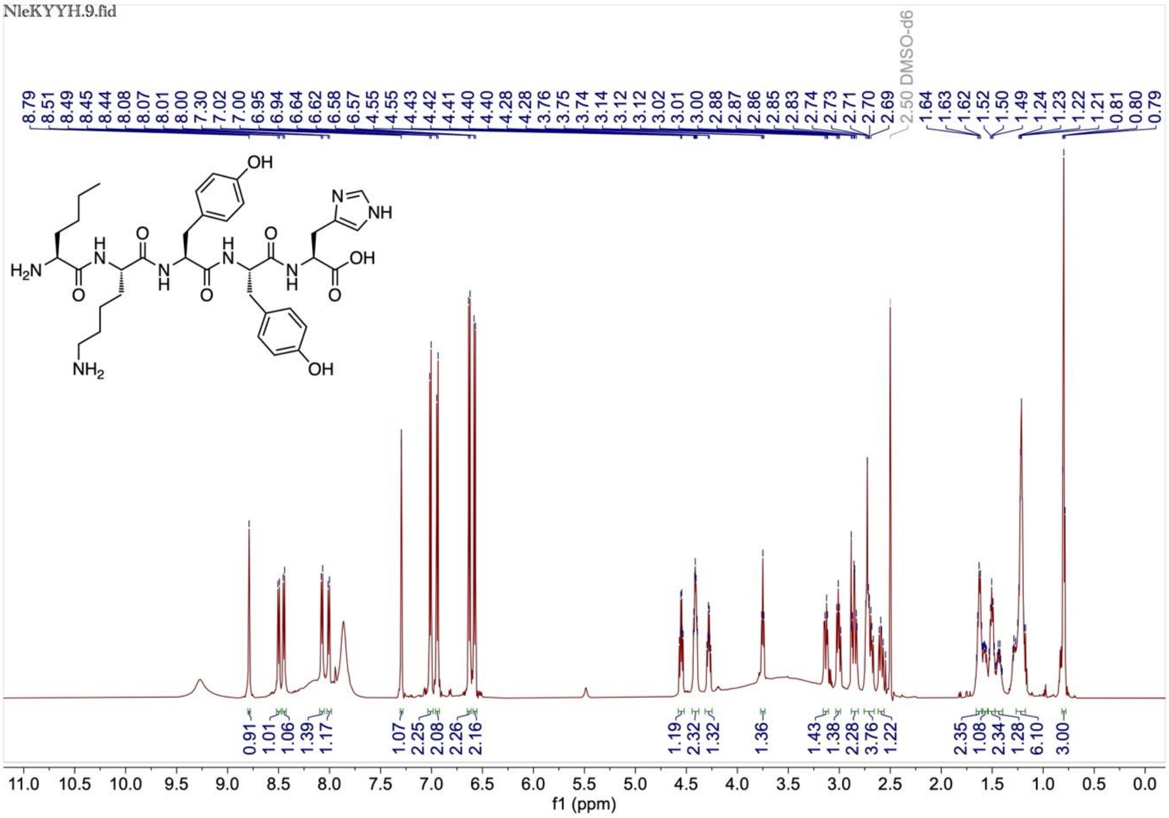

Figure S59. ^{1}H NMR spectrum of **Nle-1-(Tyr4)**.

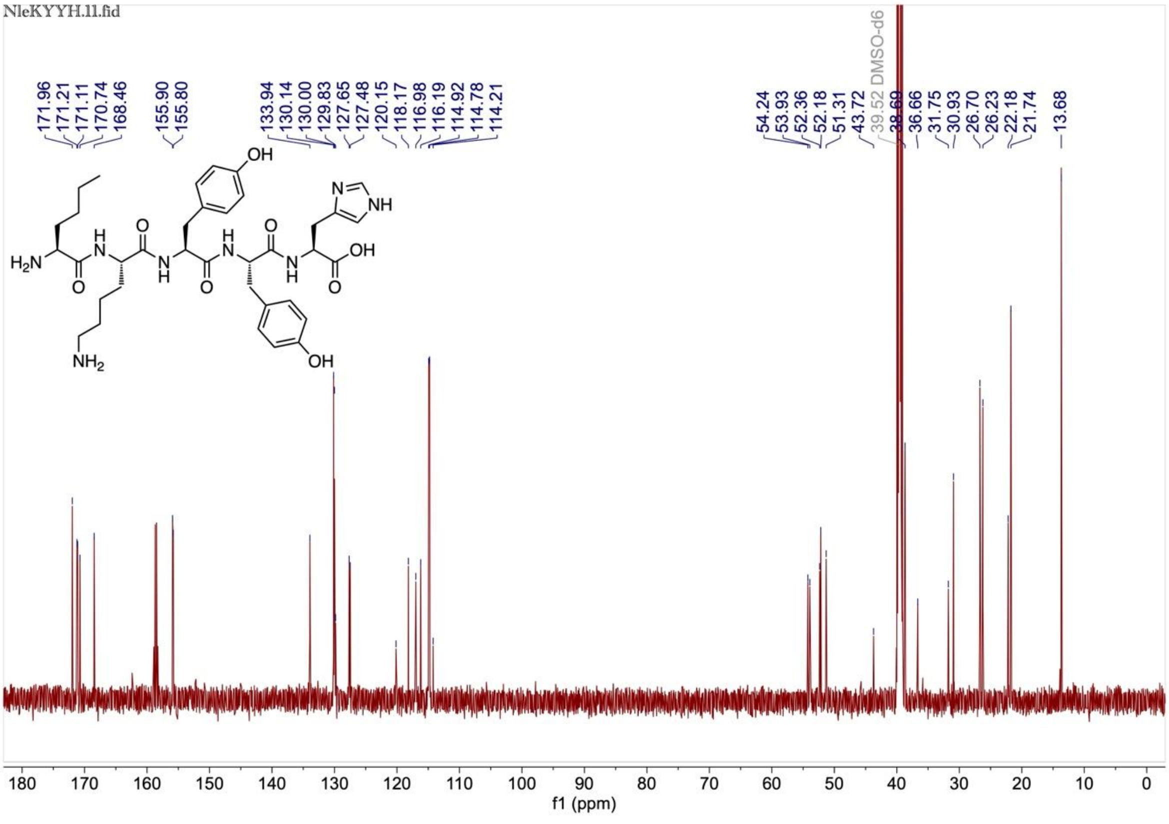

Figure S60. ^{13}C NMR spectrum of **Nle-1-(Tyr4)**.

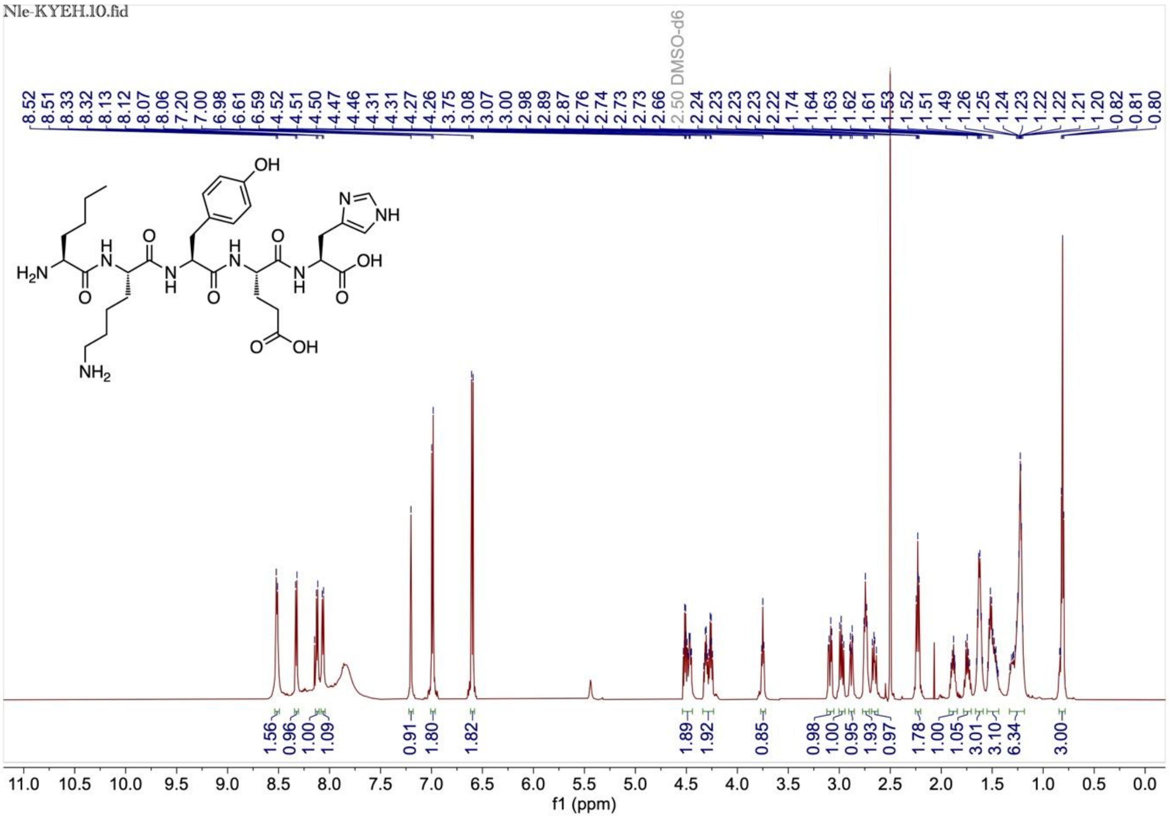

Figure S61. ^{1}H NMR spectrum of **Nle-1-(Glu4)**.

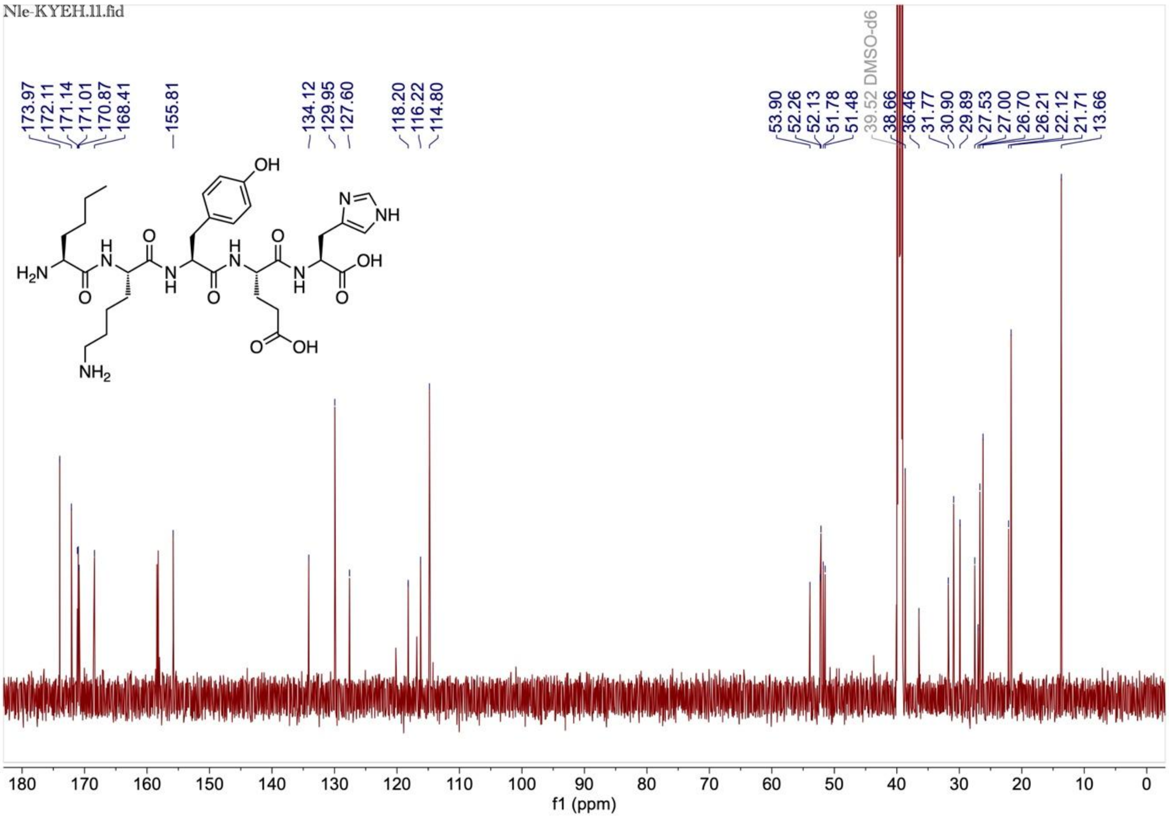

Figure S62. ^{13}C NMR spectrum of **Nle-1-(Glu4)**.

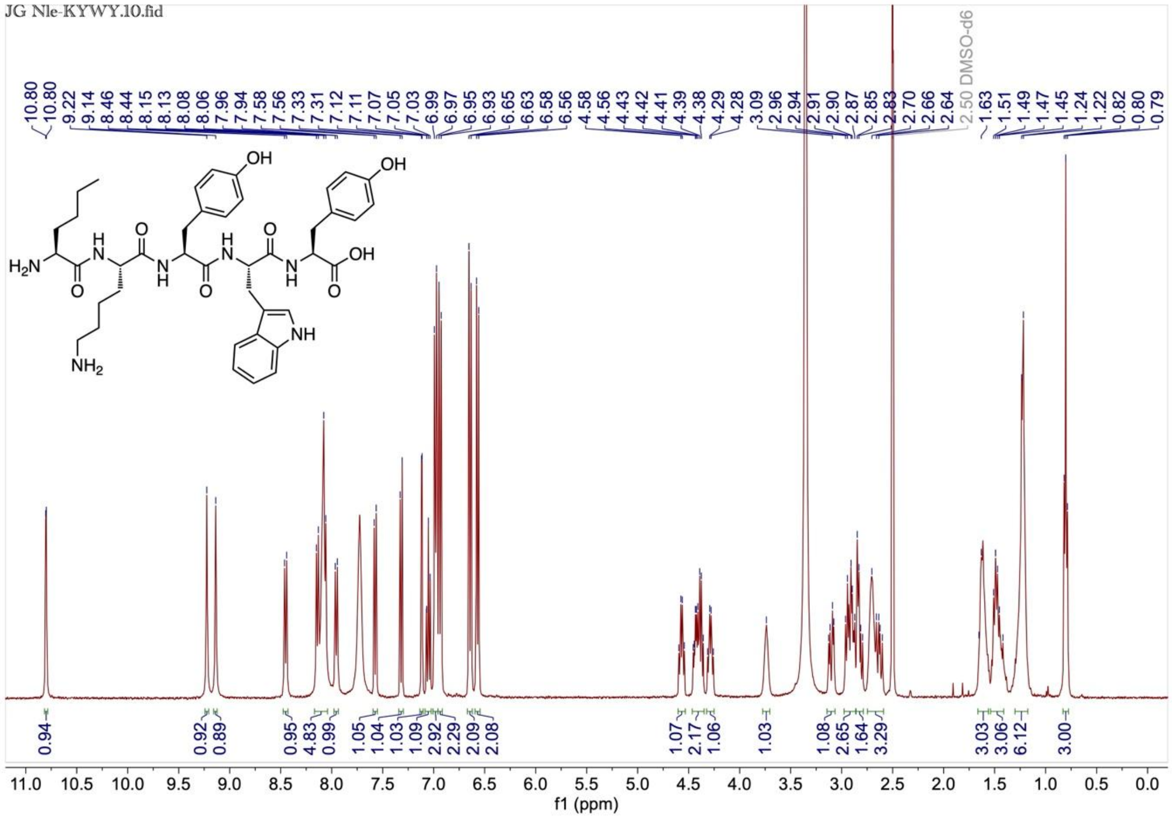

Figure S63. ^{1}H NMR spectrum of **Nle-1-(Tyr5)**.

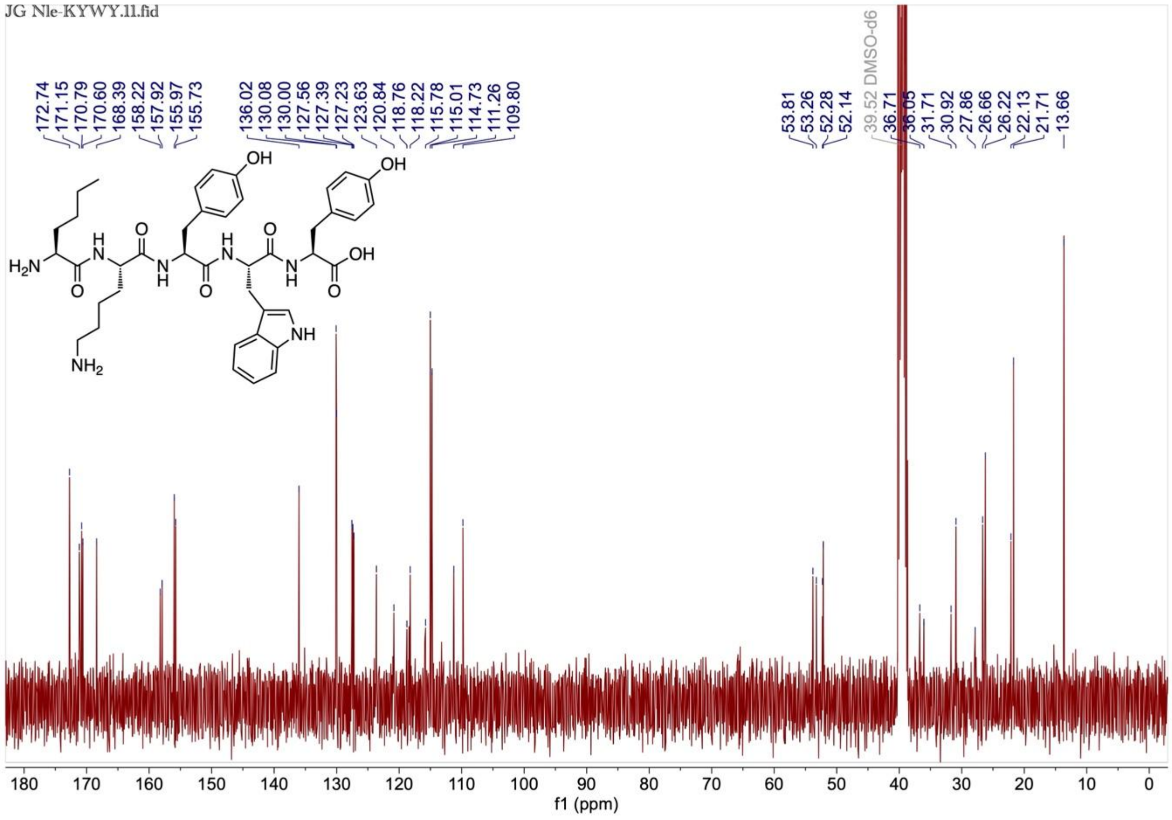

Figure S64. ^{13}C NMR spectrum of **Nle-1-(Tyr5)**.

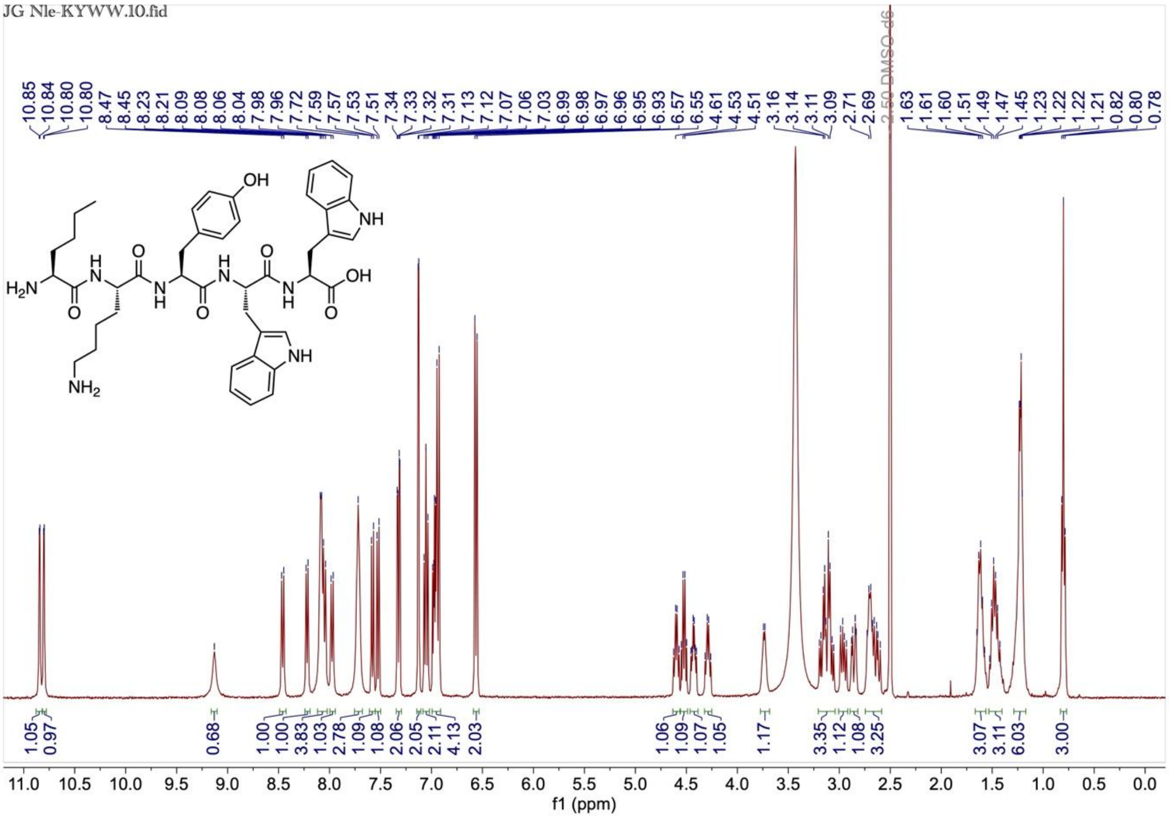

Figure S65. ^{1}H NMR spectrum of **Nle-1-(Trp5)**.

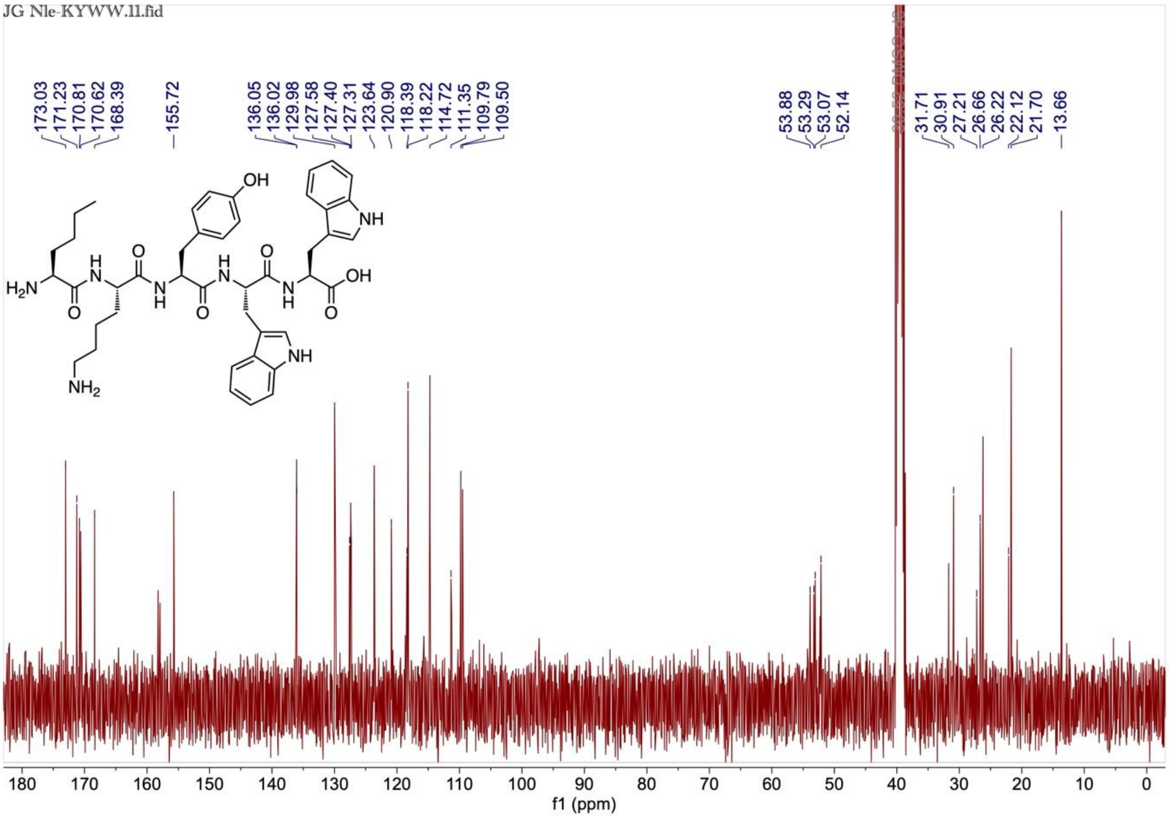

Figure S66. ^{13}C NMR spectrum of **Nle-1-(Trp5)**.

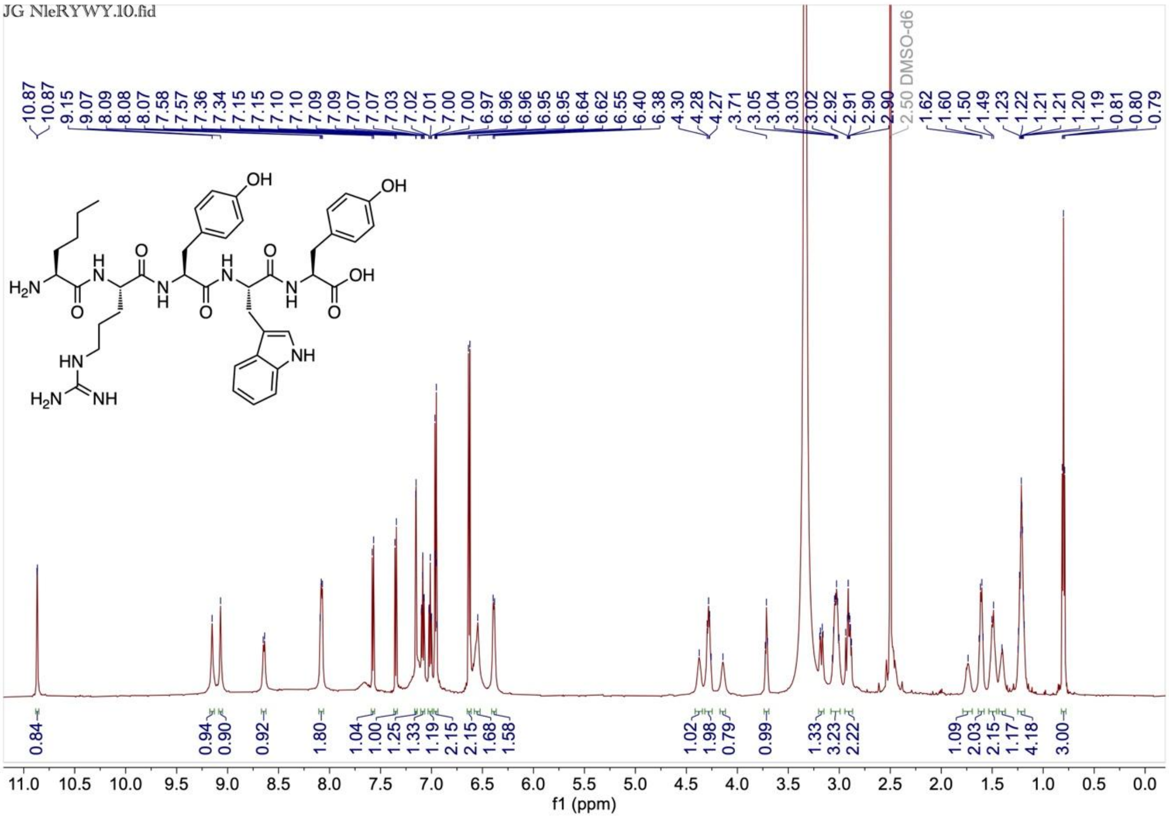

Figure S67. ^{1}H NMR spectrum of **Nle-3-(Trp4)**.

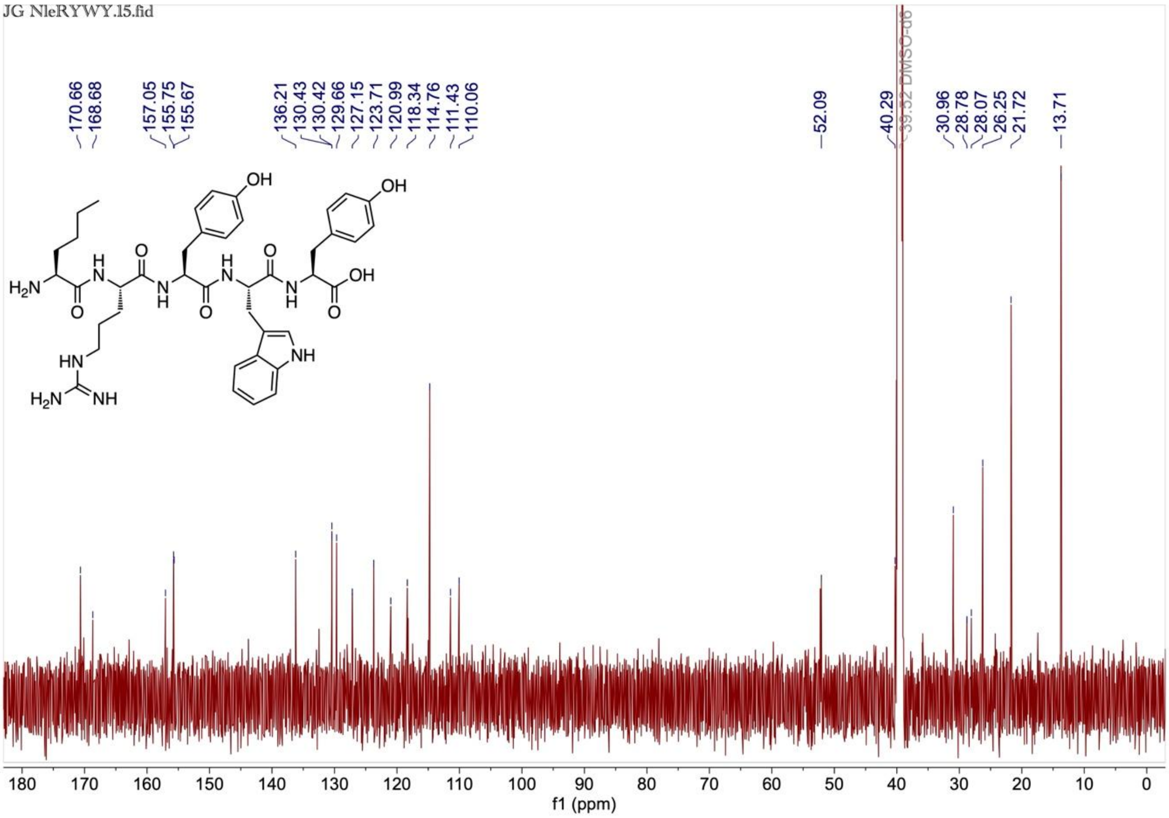

Figure S68. ^{13}C NMR spectrum of **Nle-3-(Trp4)**.

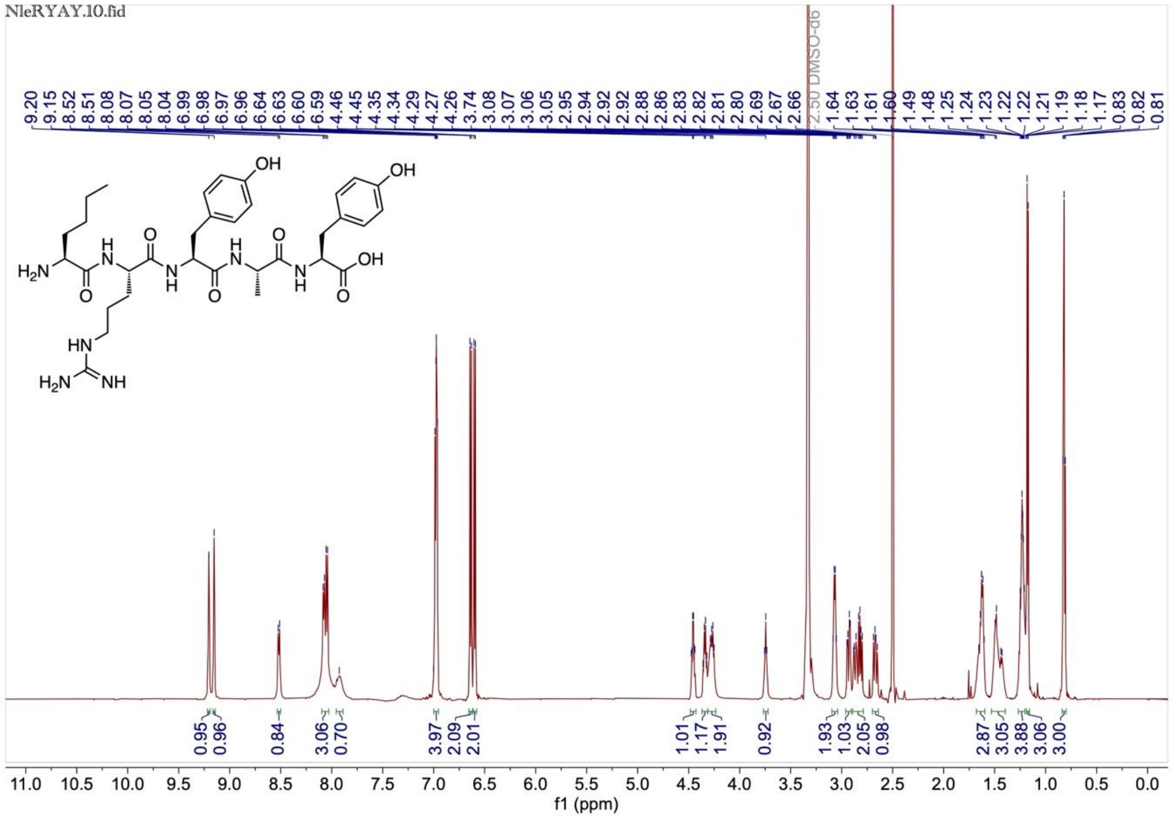

Figure S69. ^{1}H NMR spectrum of **Nle-3-(Ala4)**.

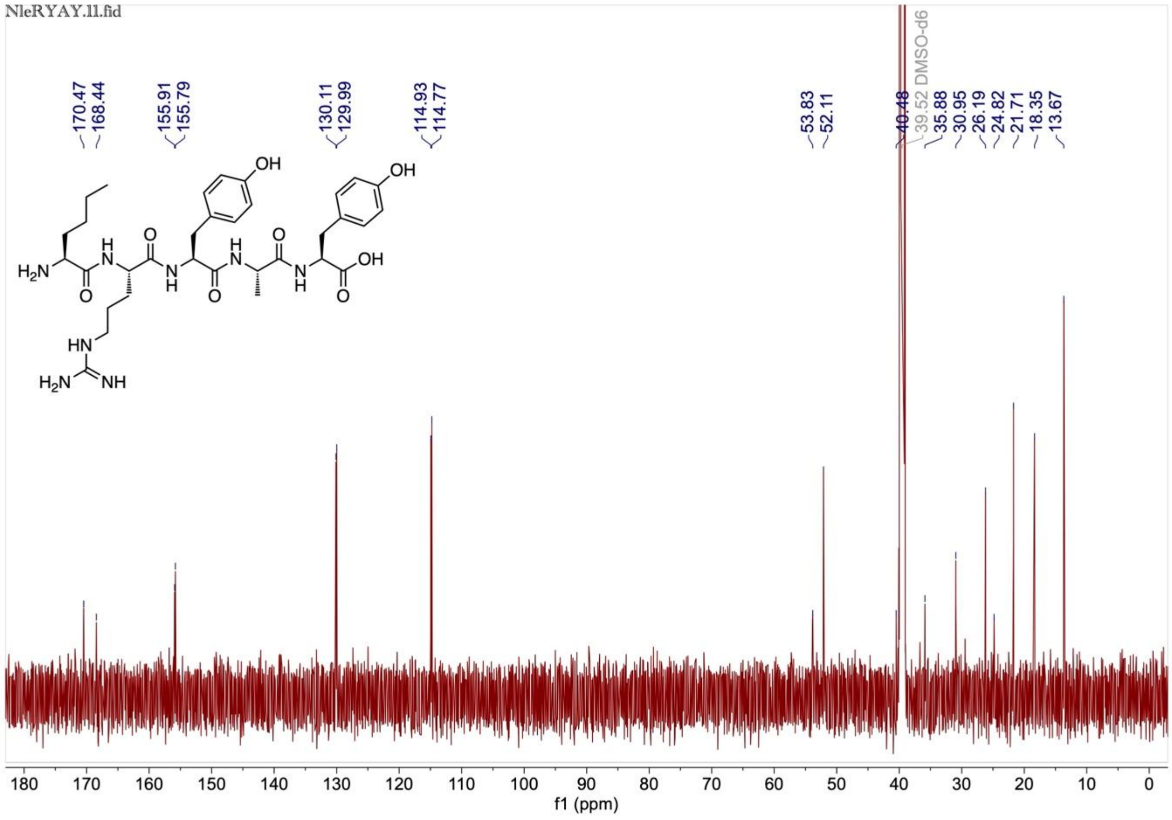

Figure S70. ^{13}C NMR spectrum of **Nle-3-(Ala4)**.

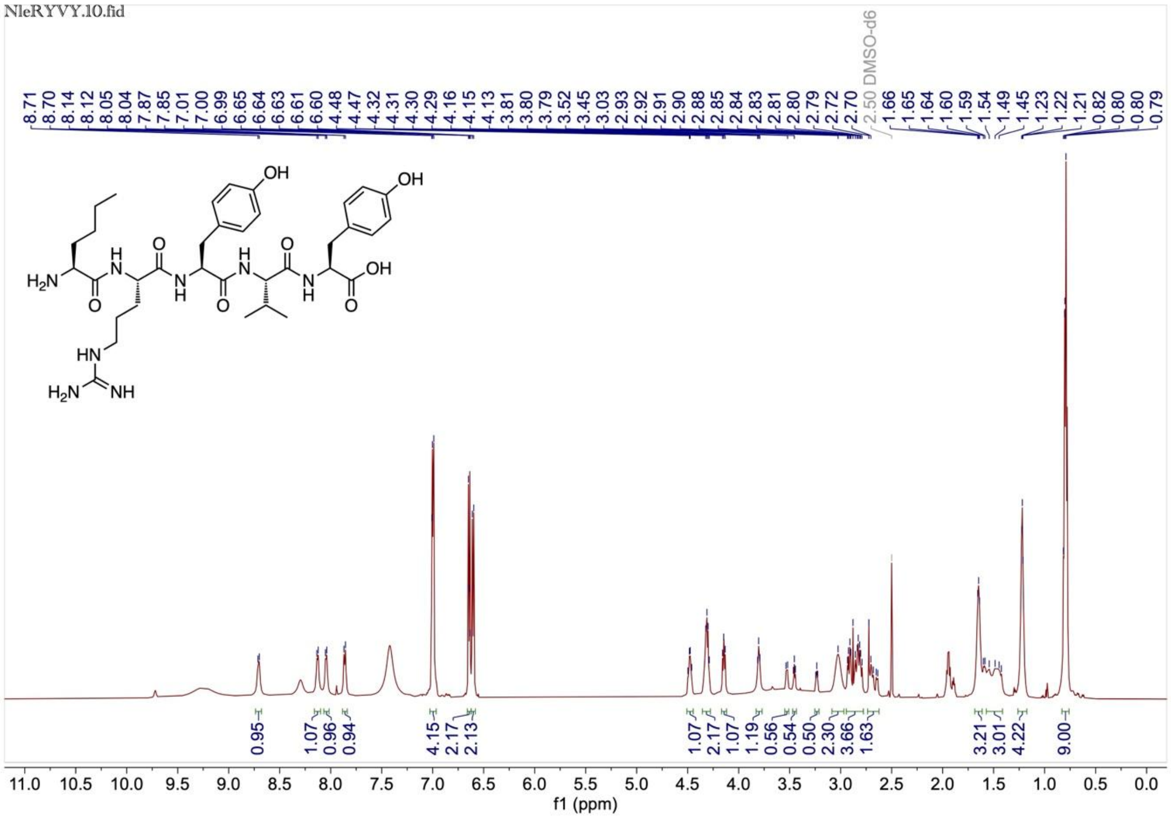

Figure S71. ^{1}H NMR spectrum of **Nle-3-(Val4)**.

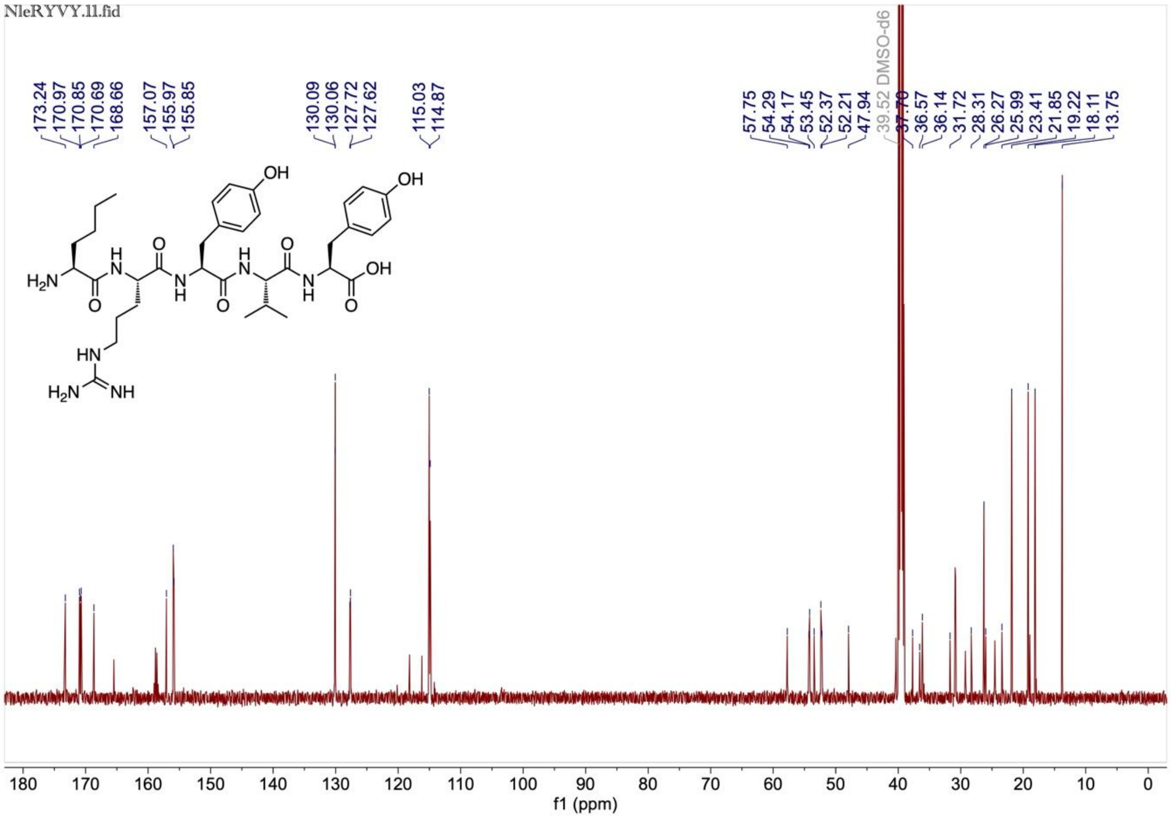

Figure S72. ^{13}C NMR spectrum of **Nle-3-(Val4)**.

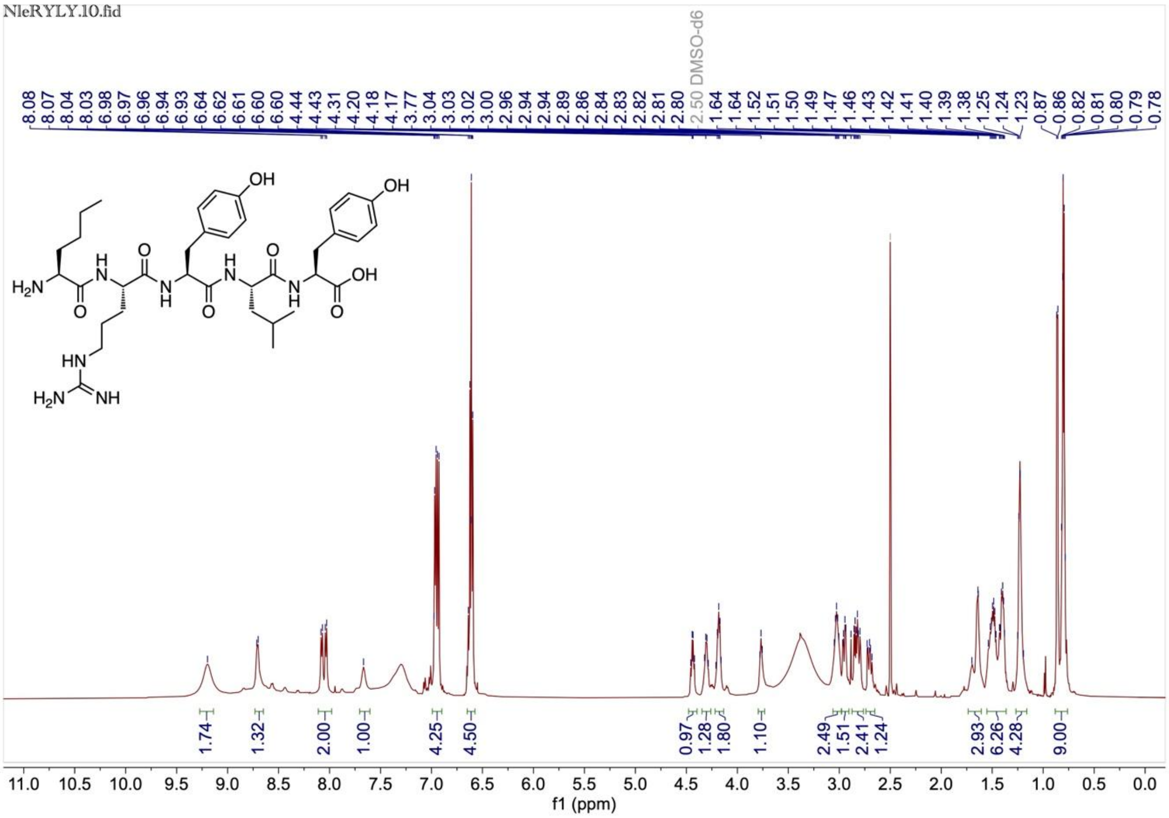

Figure S73. ^{1}H NMR spectrum of **Nle-3-(Leu4)**.

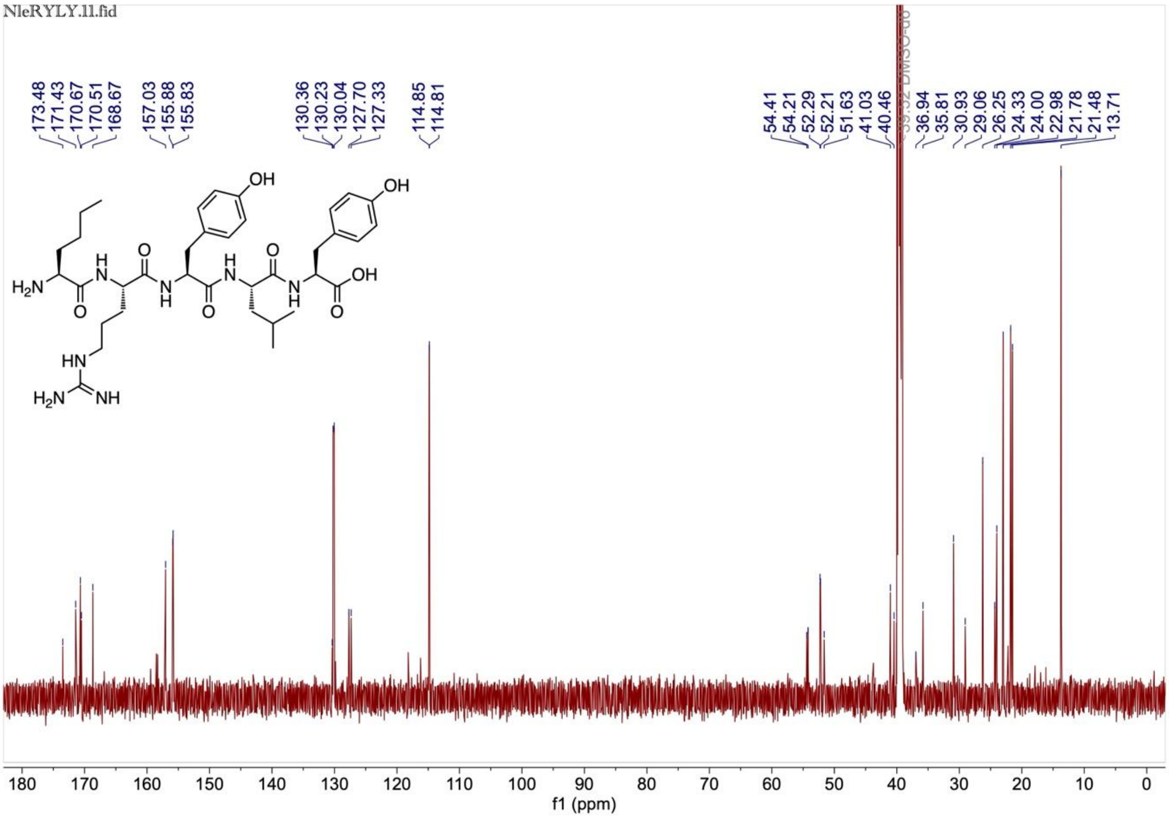

Figure S74. ^{13}C NMR spectrum of **Nle-3-(Leu4)**.

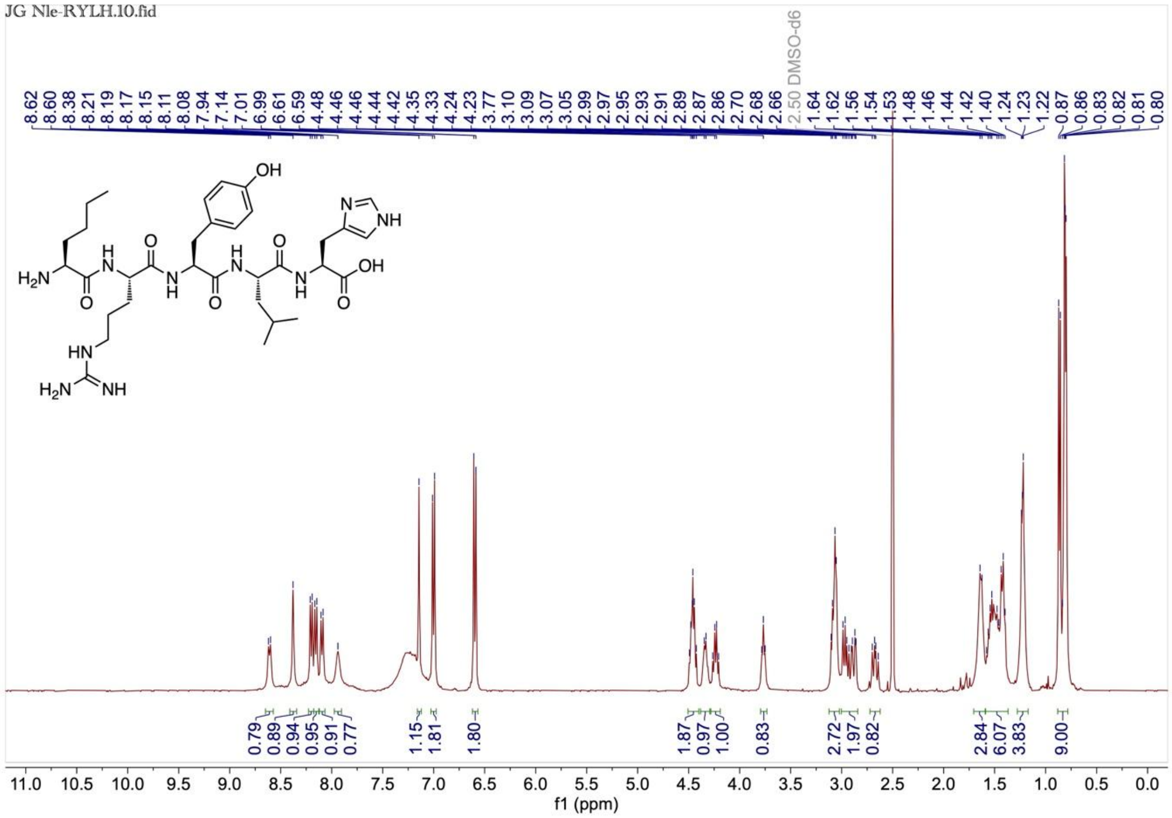

Figure S75. ^{1}H NMR spectrum of **Nle-3-(Leu4, His5)**.

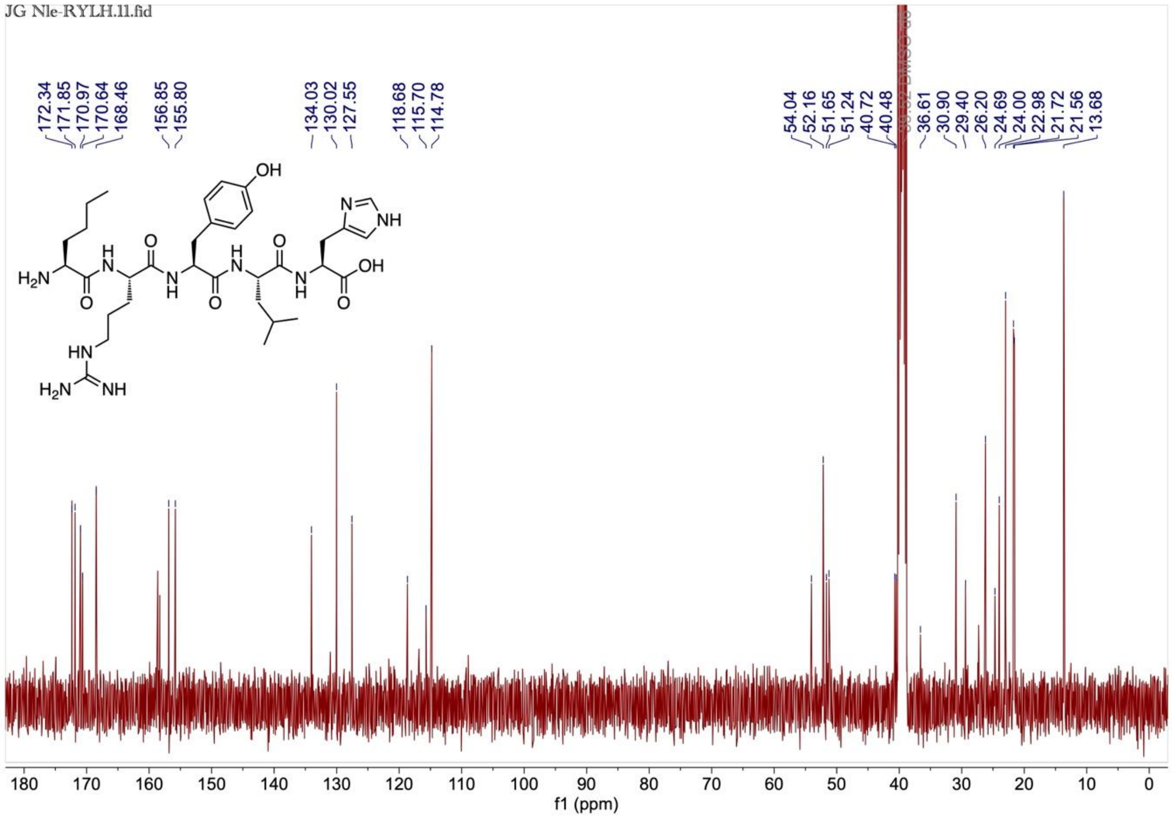

Figure S76. ^{13}C NMR spectrum of **Nle-3-(Leu4, His5)**.

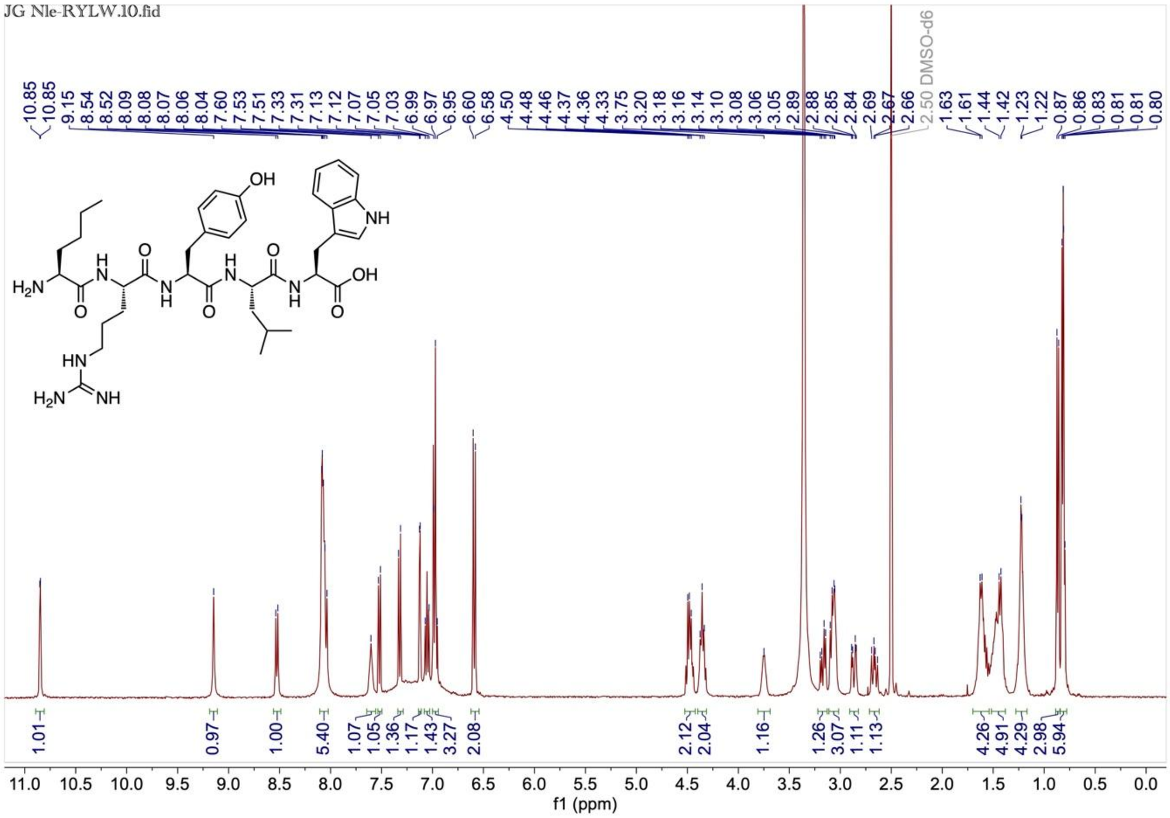

Figure S77. ^{1}H NMR spectrum of **Nle-3-(Leu4, Trp5)**.

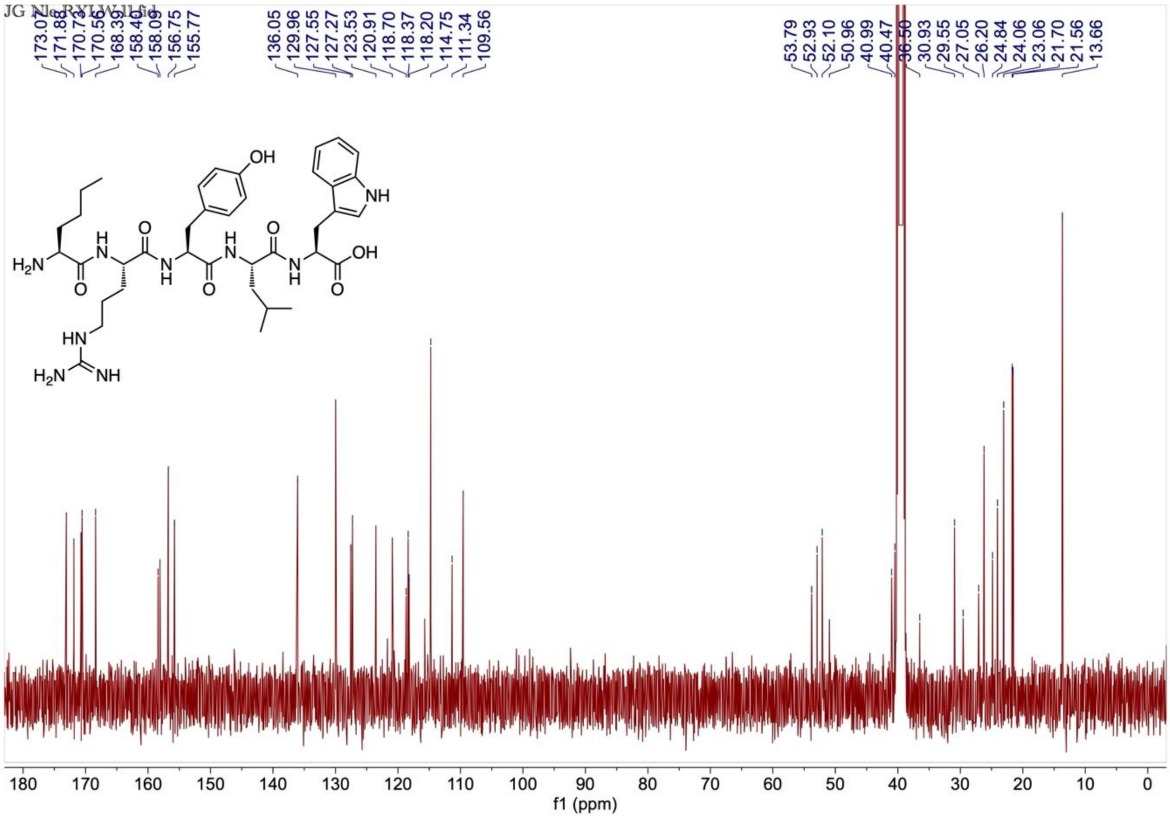

Figure S78. ^{13}C NMR spectrum of **Nle-3-(Leu4, Trp5)**.

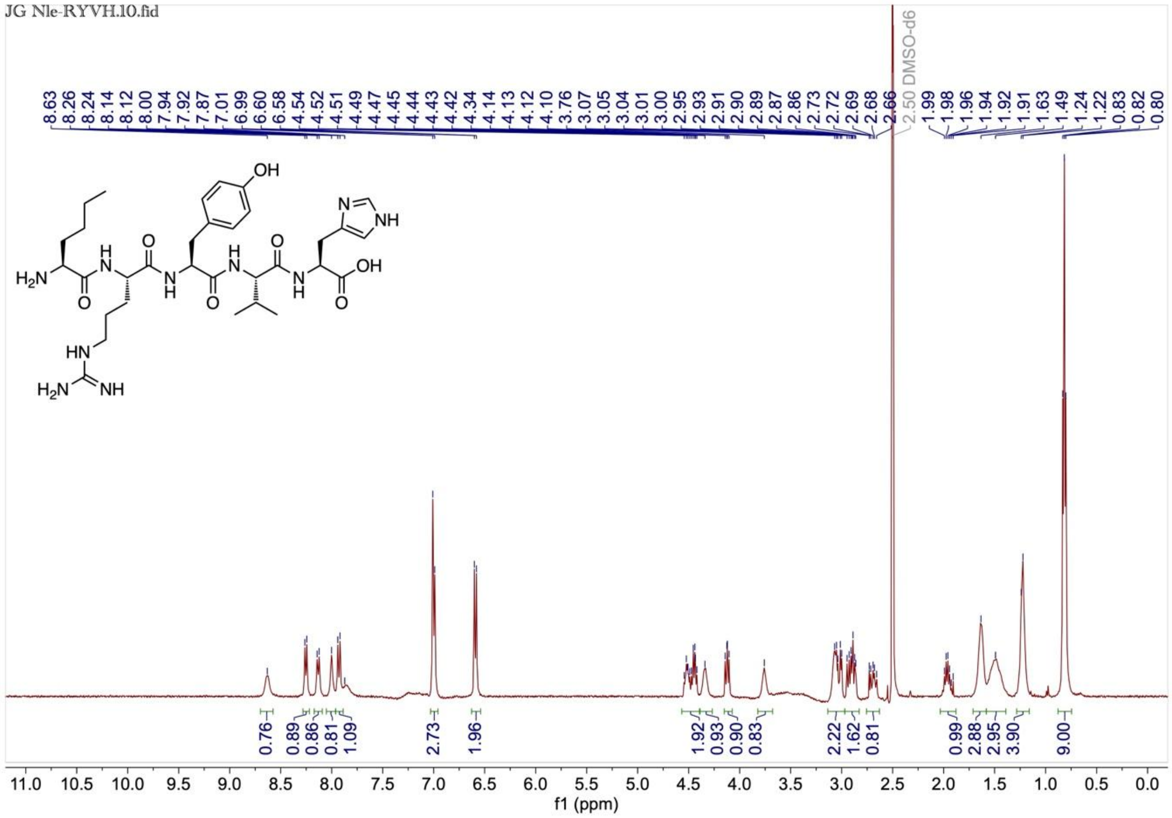

Figure S79. ^{1}H NMR spectrum of **Nle-6**.

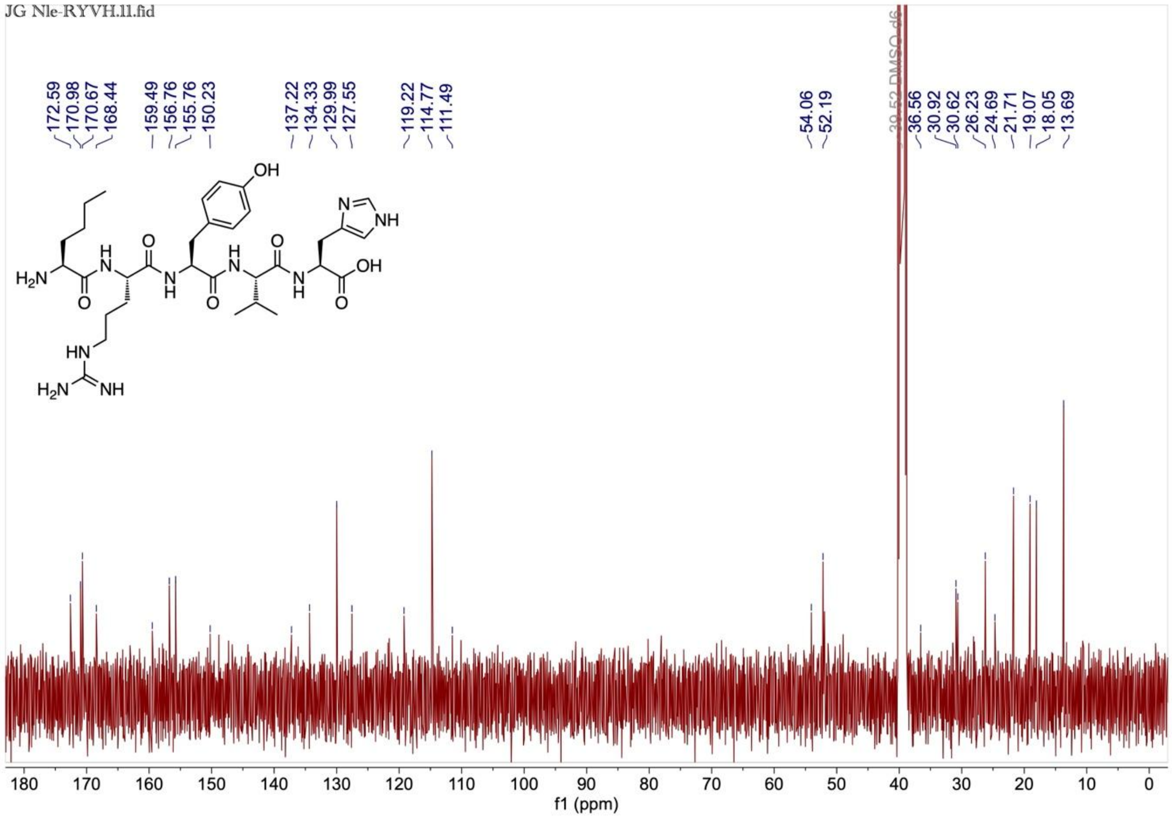

Figure S80. ^{13}C NMR spectrum of **Nle-6**.

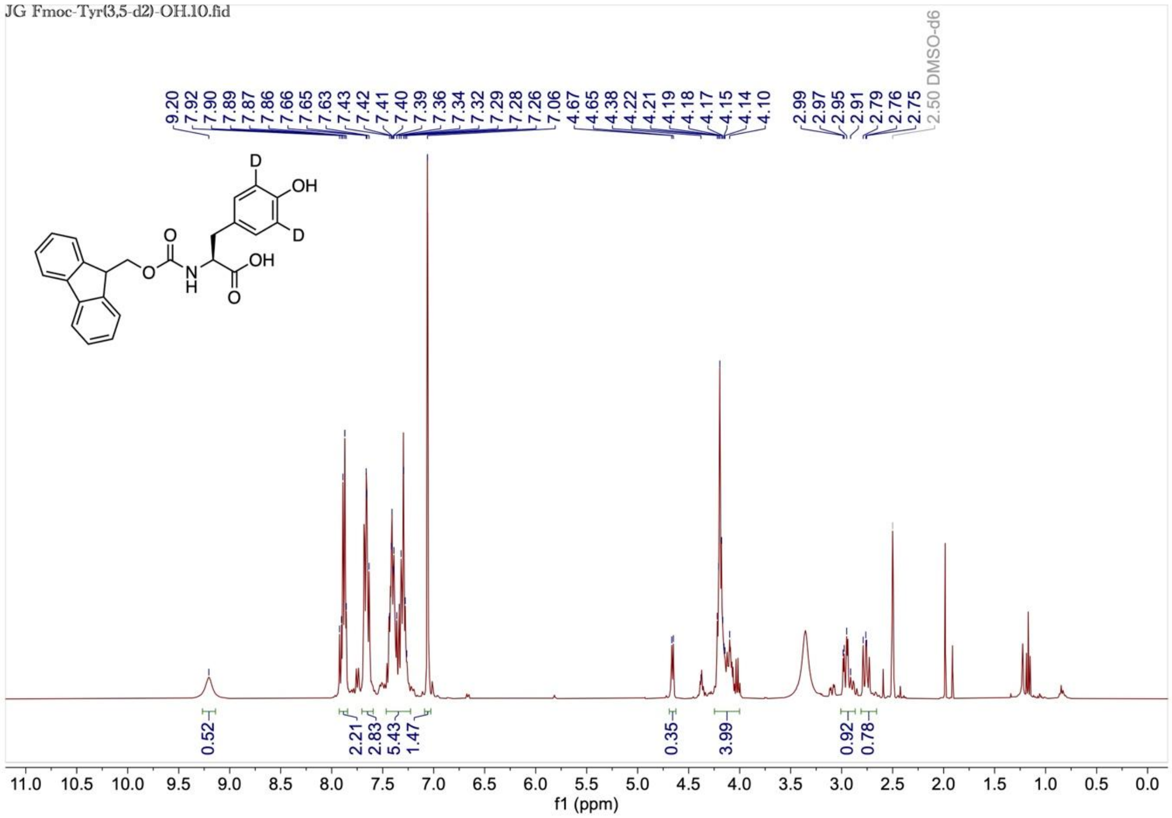

Figure S81. ^{1}H NMR spectrum of **Fmoc-Tyr(3,5-d$_2$)-OH**. Unmarked peaks are impurities and solvent; the amino acid was used as crude due to subsequent incorporation into peptides followed by their purification.

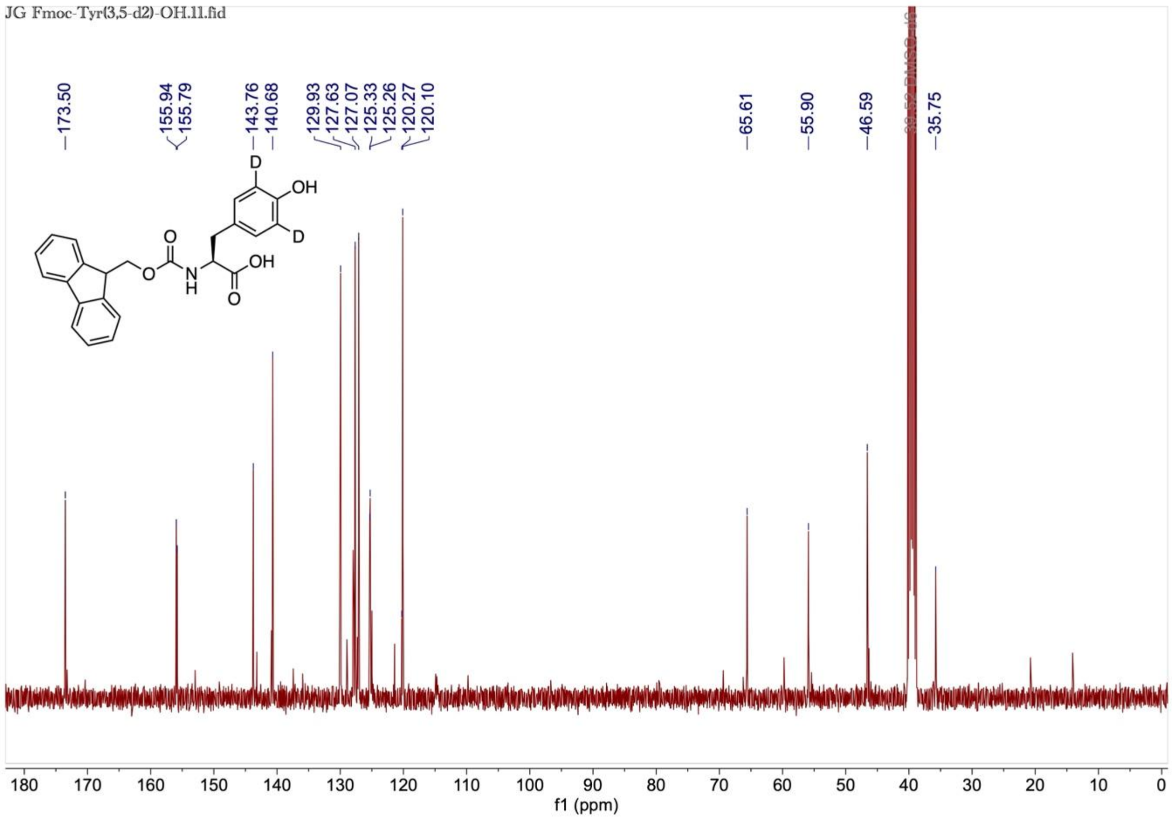

Figure S82. ^{13}C NMR spectrum of **Fmoc-Tyr(3,5-d$_2$)-OH**. Unmarked peaks are impurities and solvent; the amino acid was used as crude due to subsequent incorporation into peptides followed by their purification.

6. Analysis of crosslinking reactions with AchB

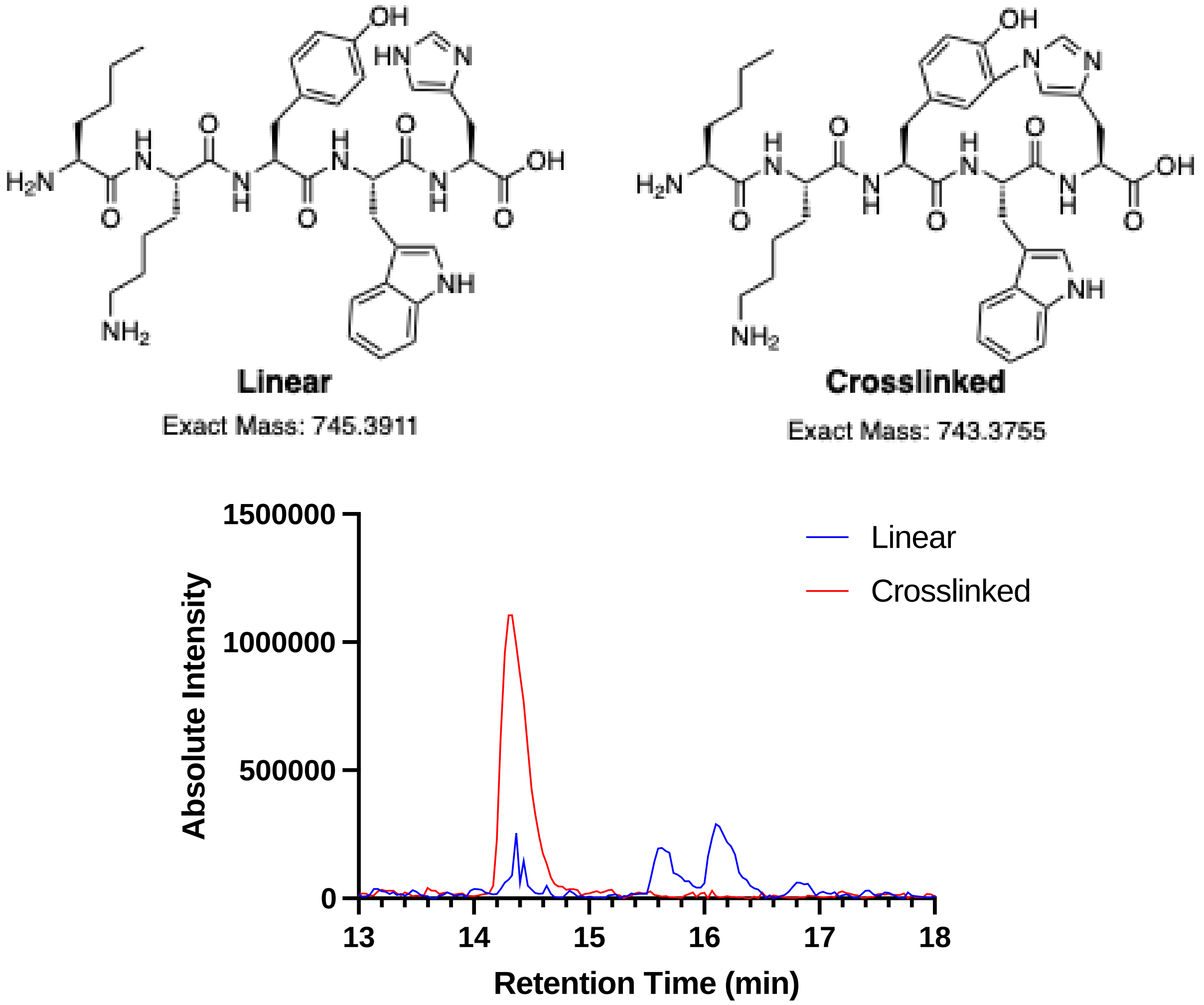

Figure S83. Crosslinking assay data for **Nle-1** with AchB. Structures of linear and crosslinked peptides shown.

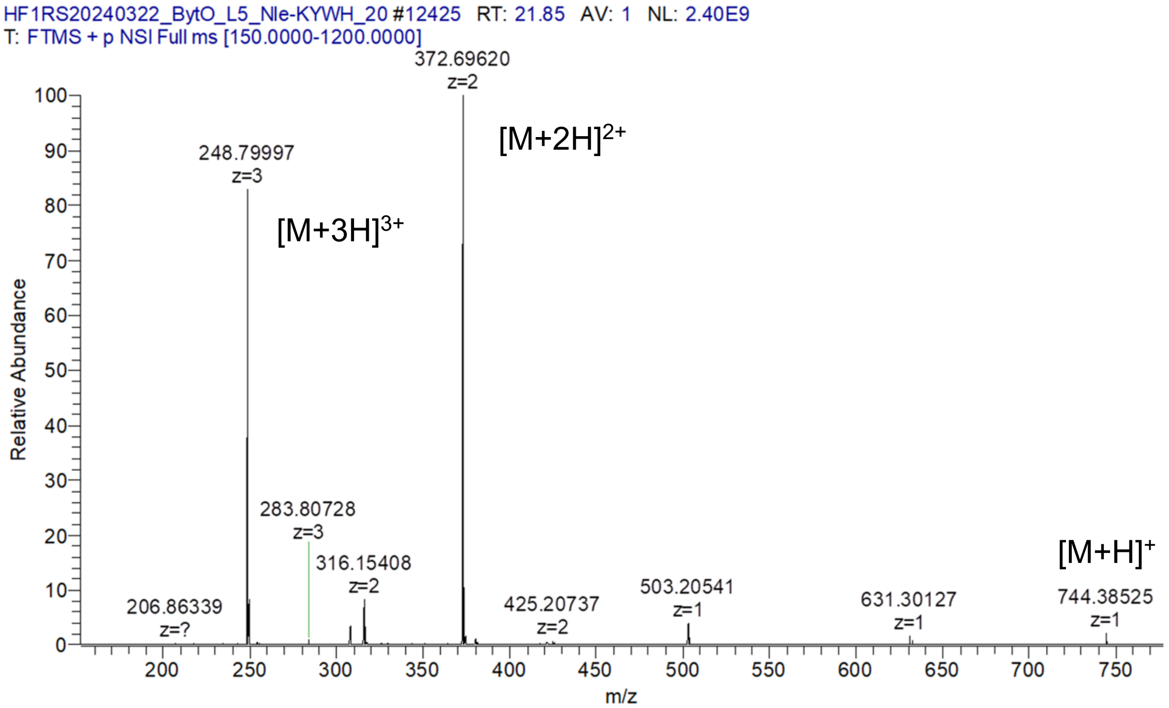

Figure S84. HRMS of crosslinked product (**Nle-2**) formed from the turnover of **Nle-1** with AchB.

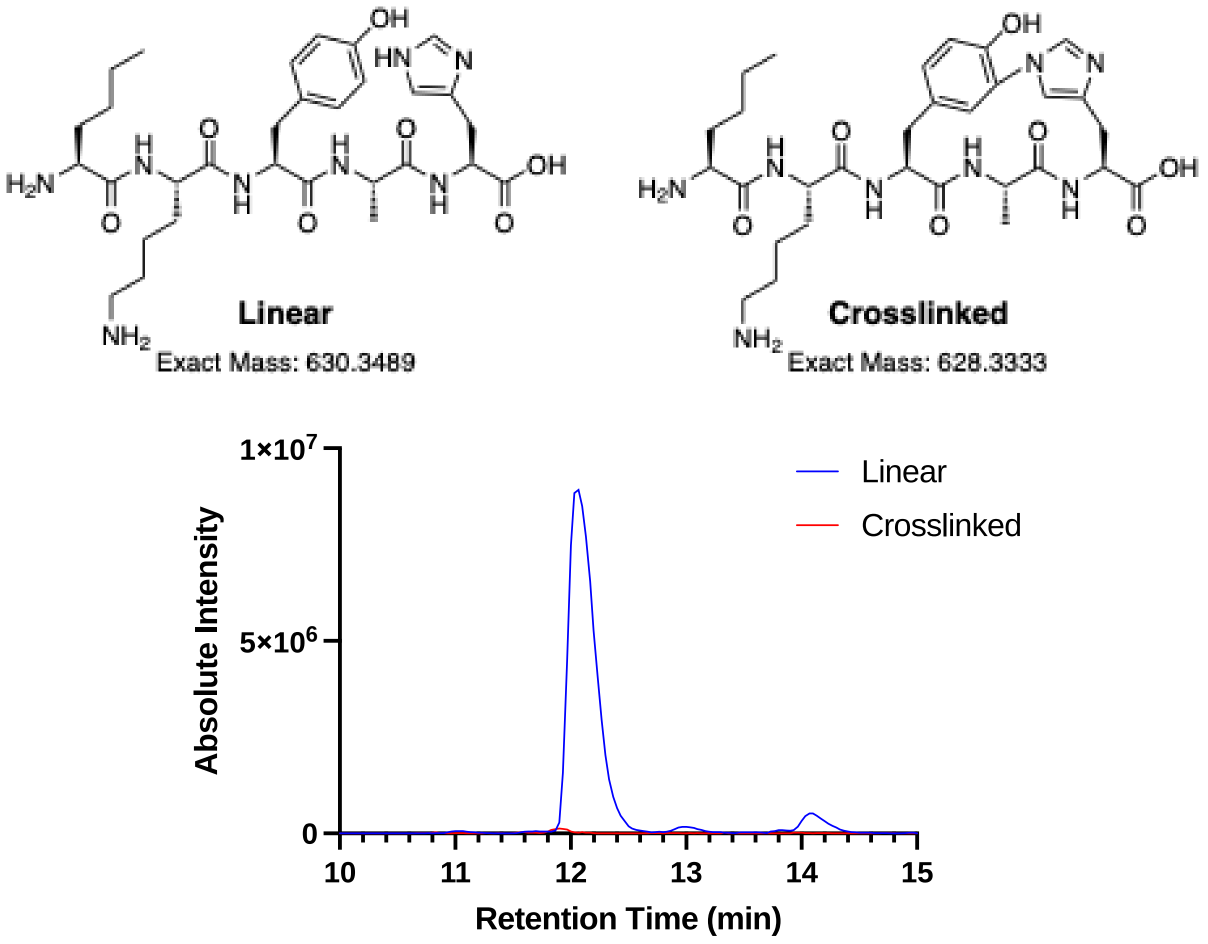

Figure S85. Crosslinking assay data for **Nle-1-(Ala4)** with AchB. Structures of linear and crosslinked peptides shown.

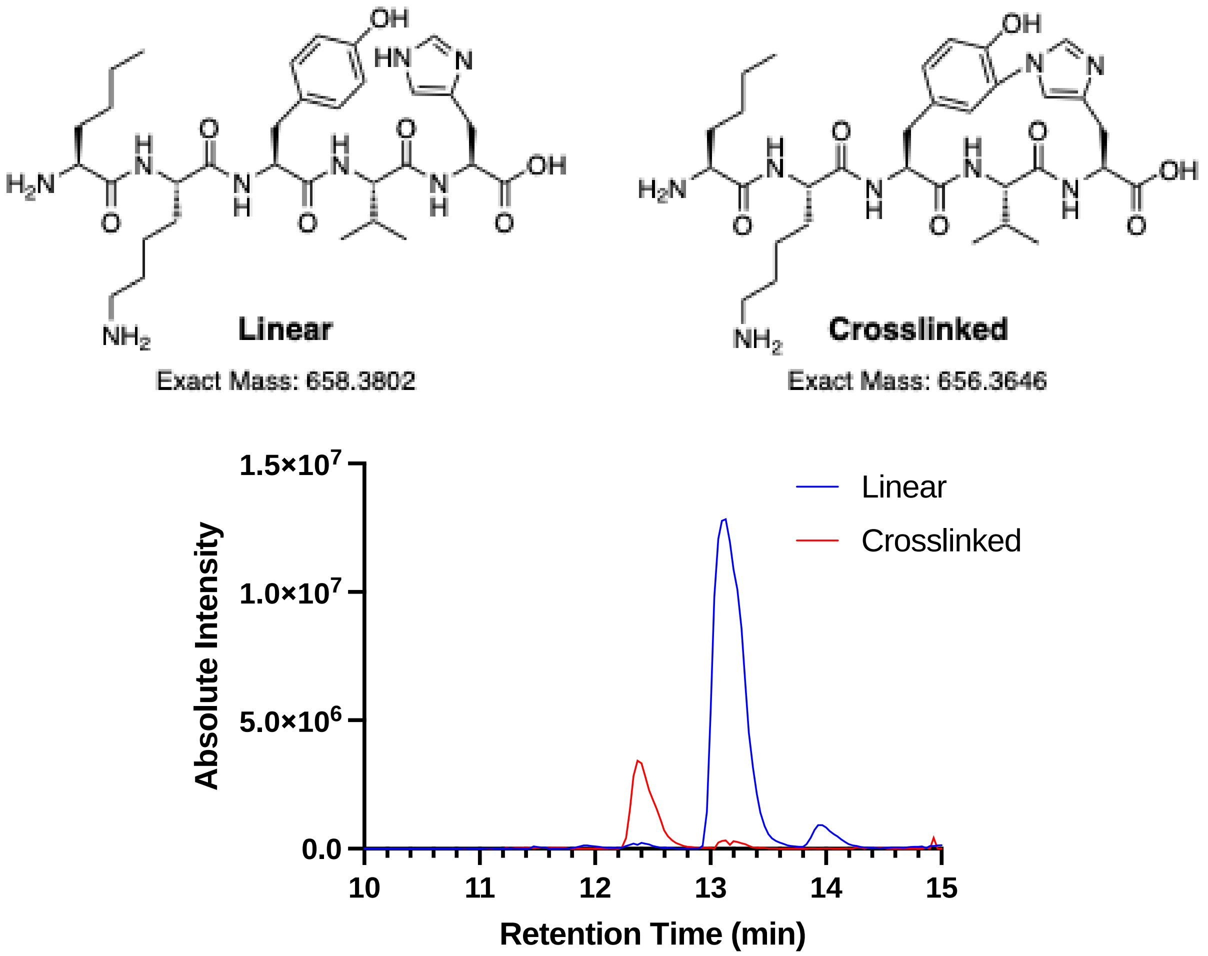

Figure S86. Crosslinking assay data for **Nle-1-(Val4)** with AchB. Structures of linear and crosslinked peptides shown.

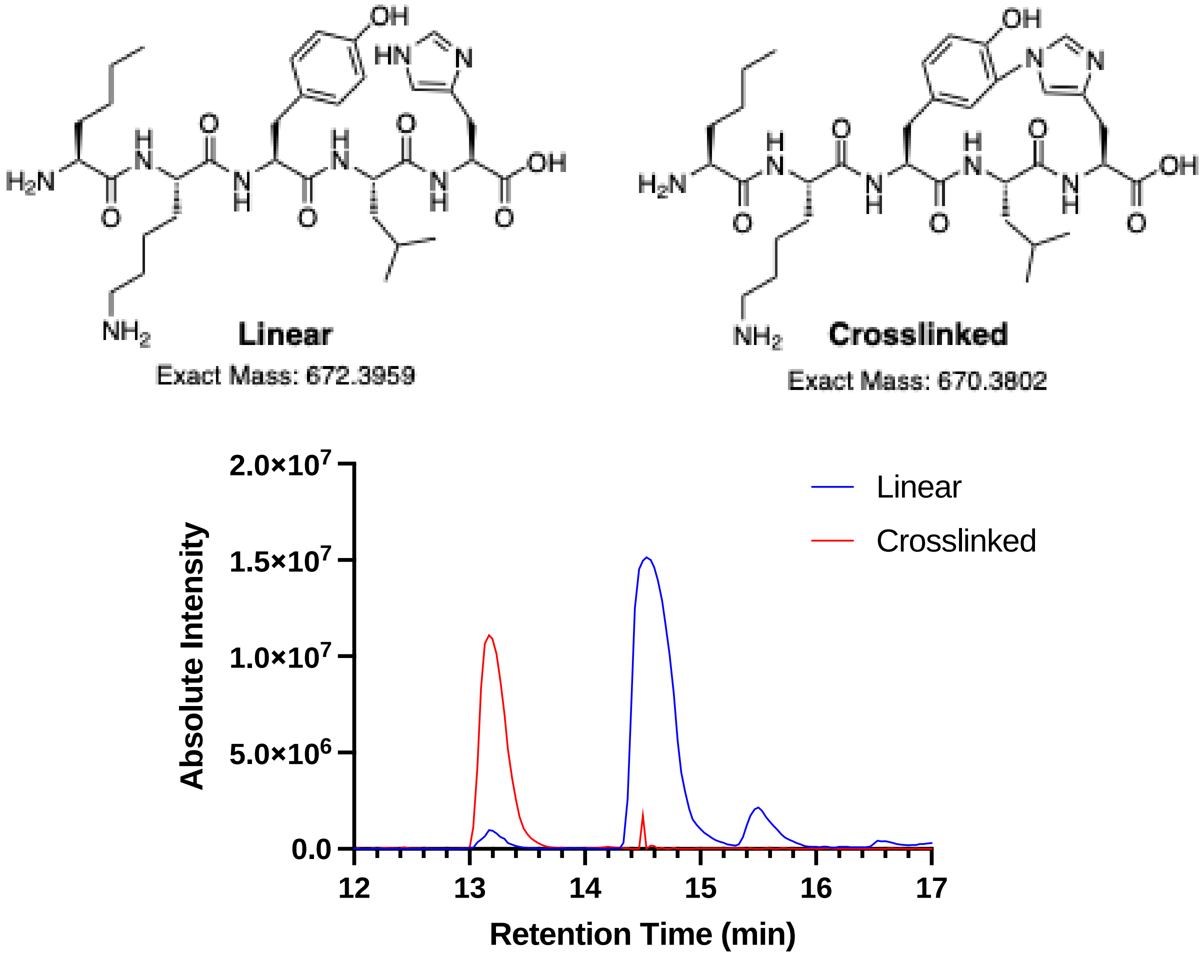

Figure S87. Crosslinking assay data for **Nle-1-(Leu4)** with AchB. Structures of linear and crosslinked peptides shown.

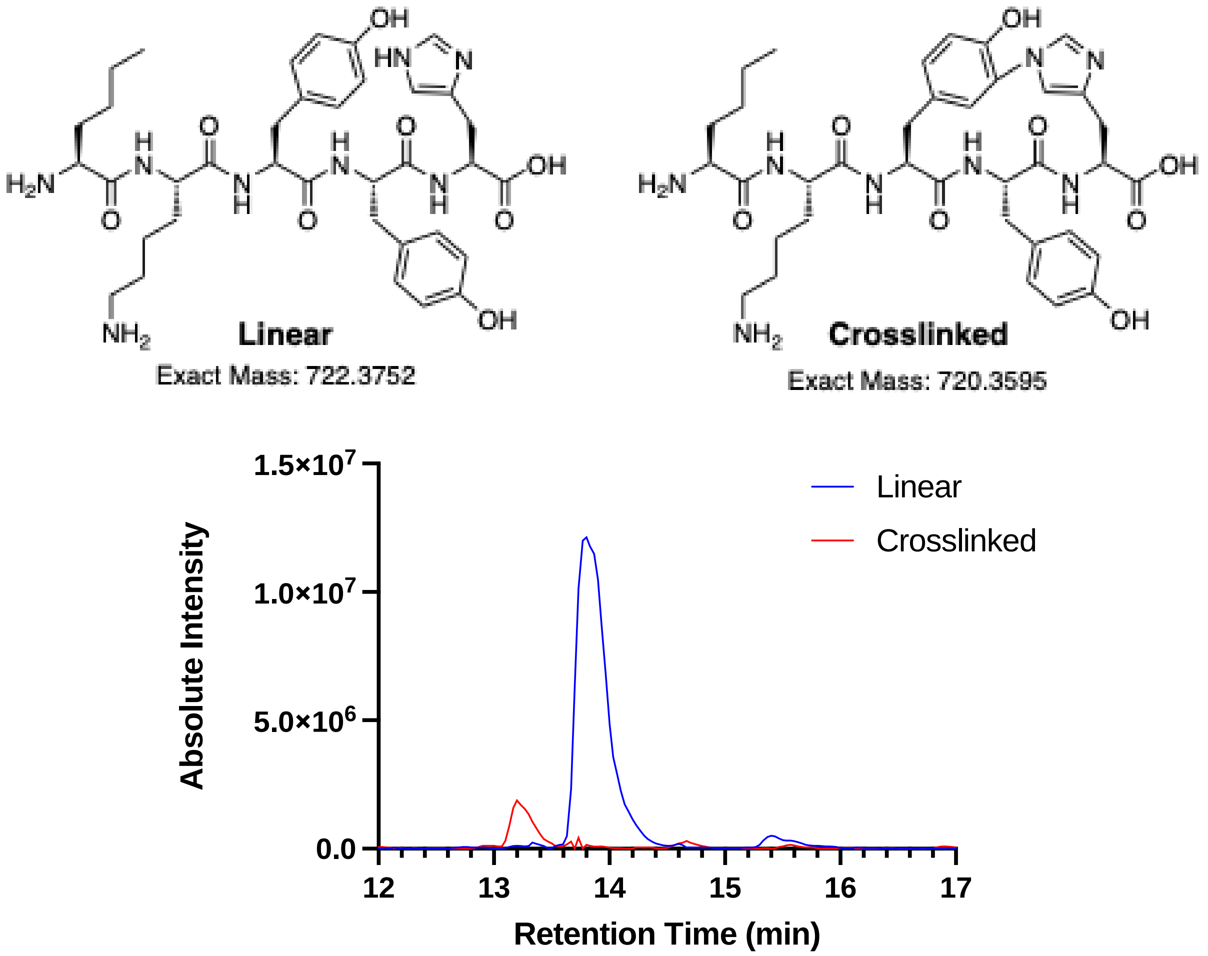

Figure S88. Crosslinking assay data for **Nle-1-(Tyr4)** with AchB. Structures of linear and crosslinked peptides shown.

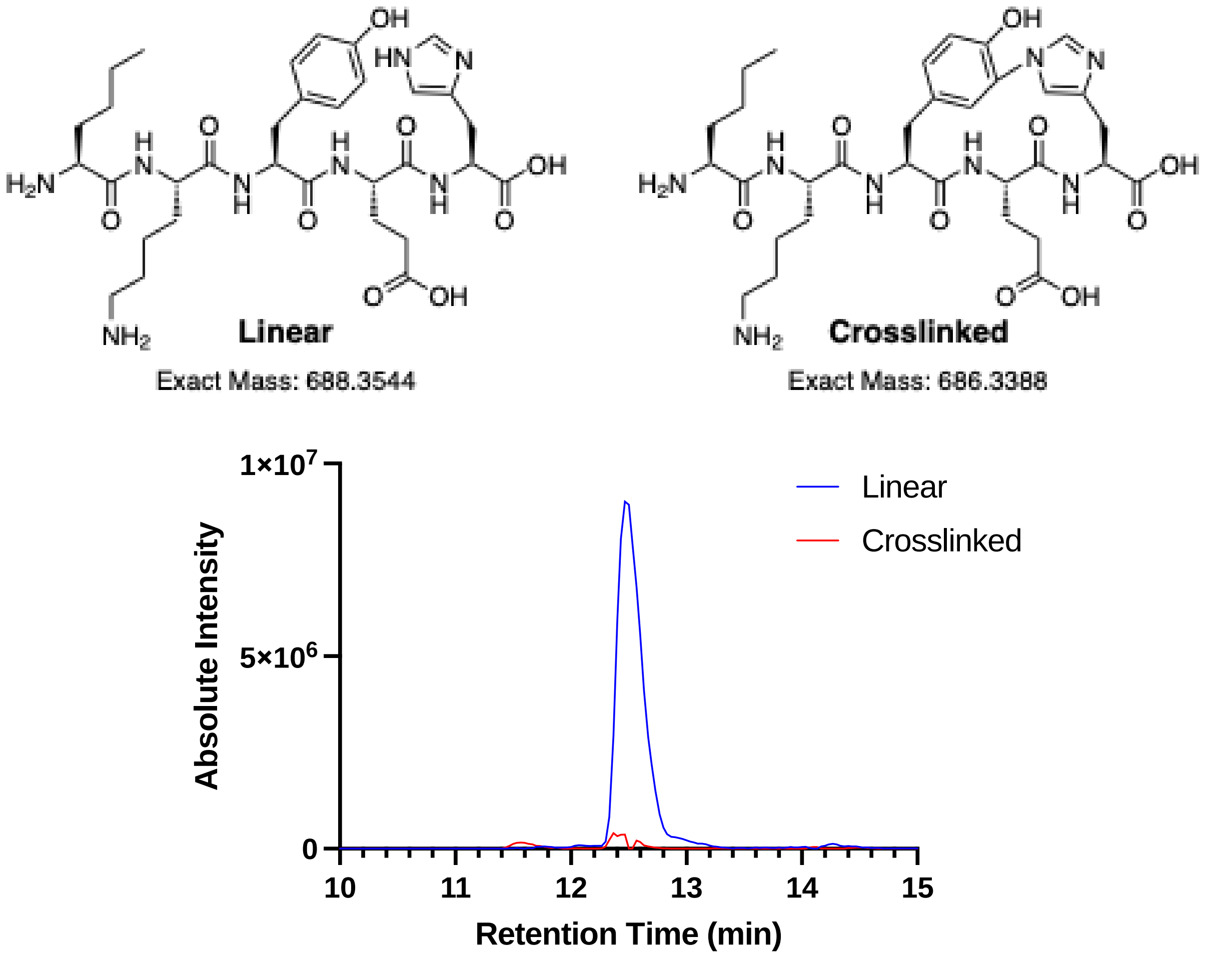

Figure S89. Crosslinking assay data for **Nle-1-(Glu4)** with AchB. Structures of linear and crosslinked peptides shown.

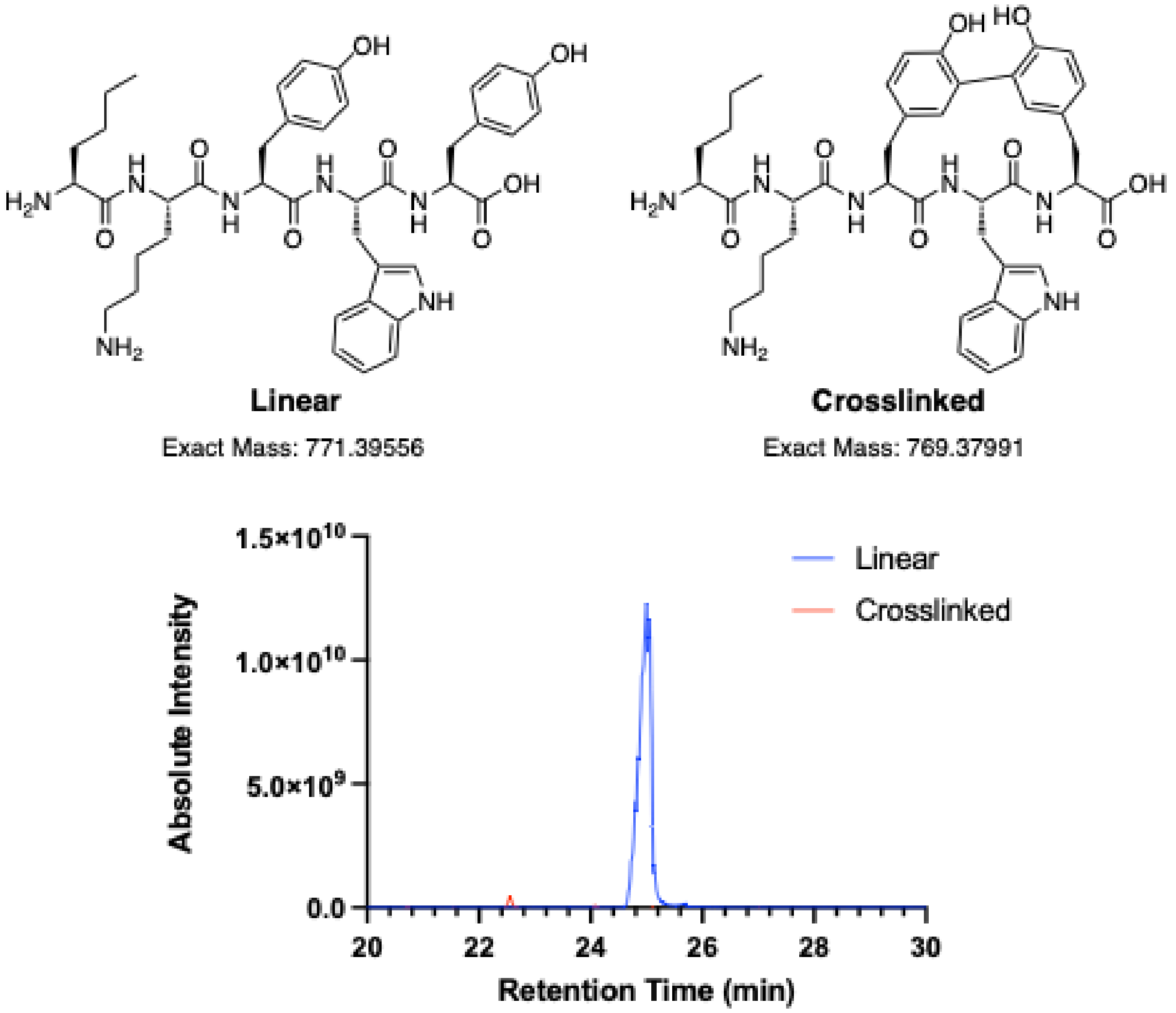

Figure S90. Crosslinking assay data for **Nle-1-(Tyr5)** with AchB. Structures of linear and crosslinked peptides shown.

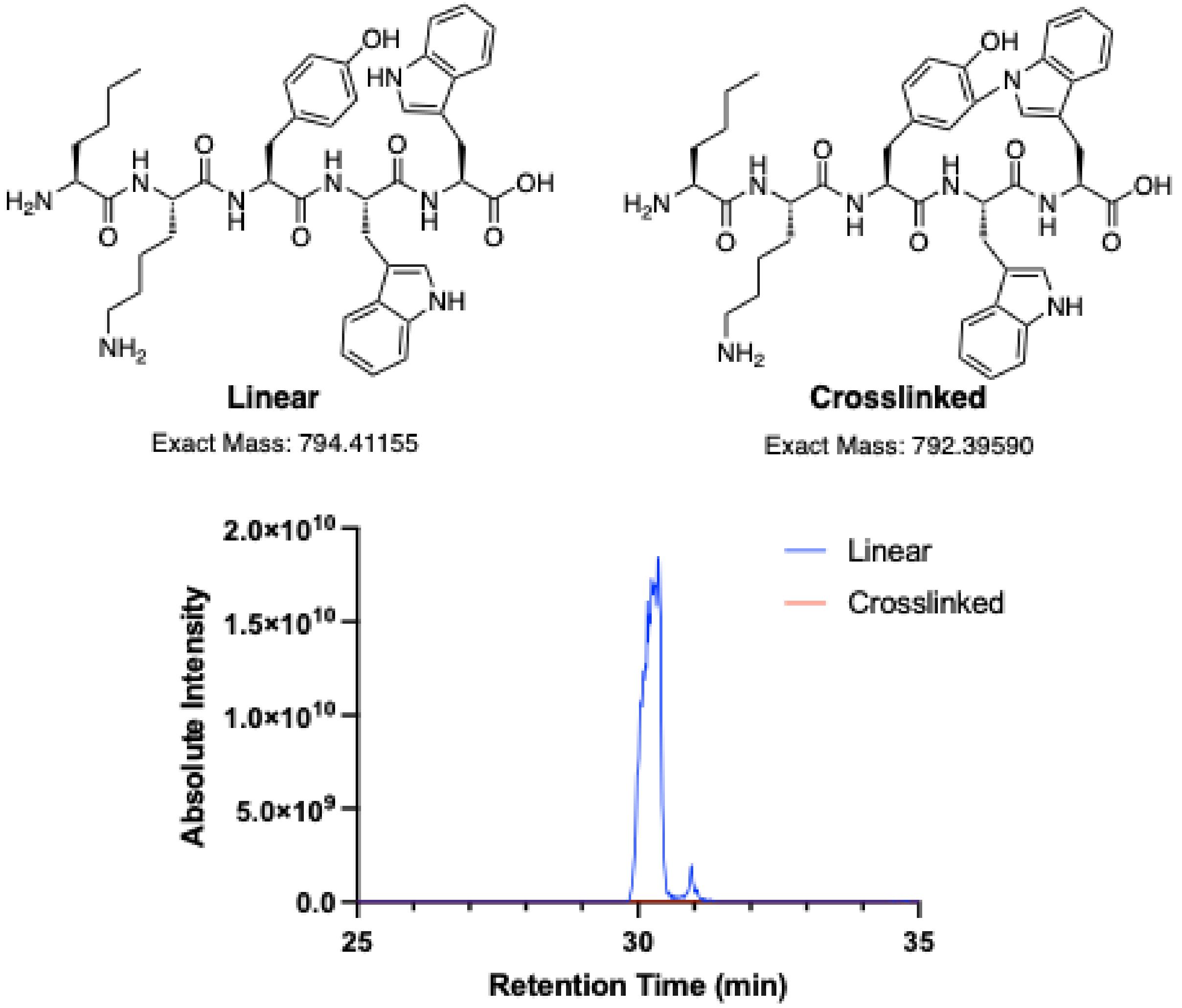

Figure S91. Crosslinking assay data for **Nle-1-(Trp5)** with AchB. Structures of linear and crosslinked peptides shown.

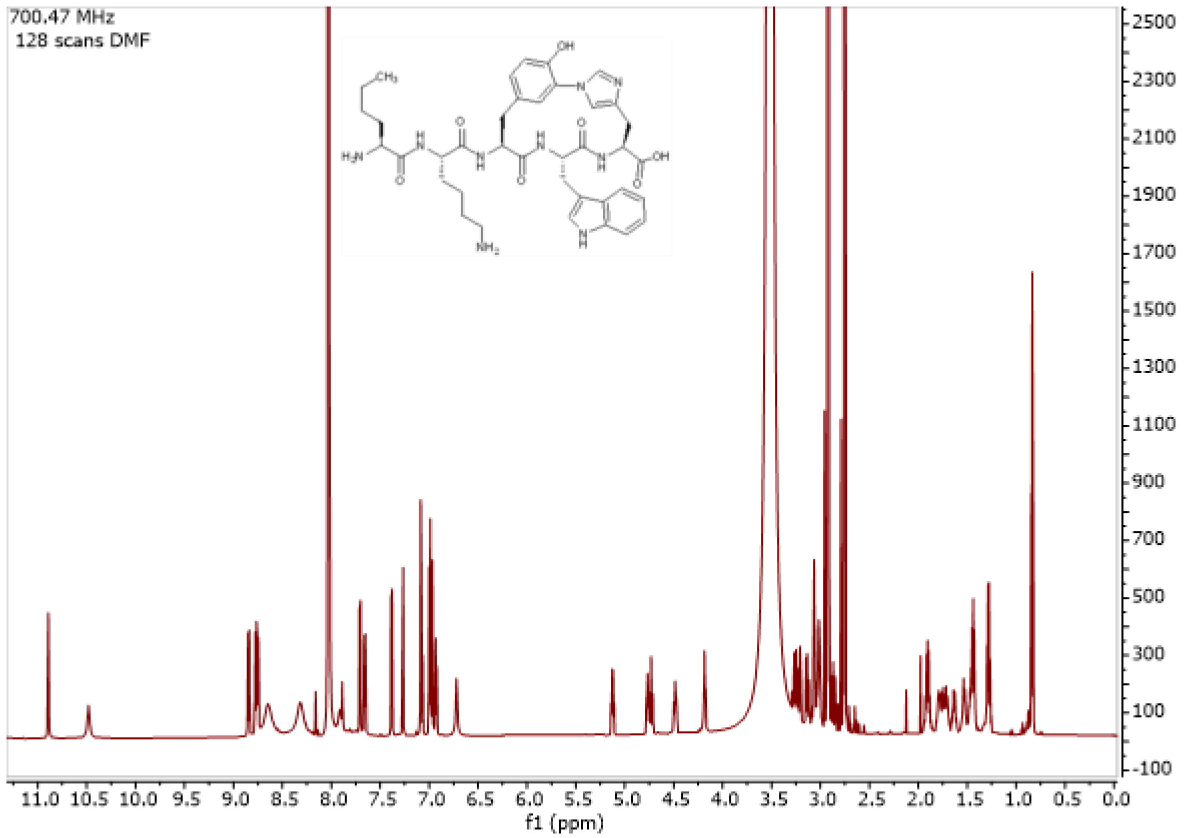

Figure S92. ^{1}H spectrum of **Nle-2** in DMF-d_7.

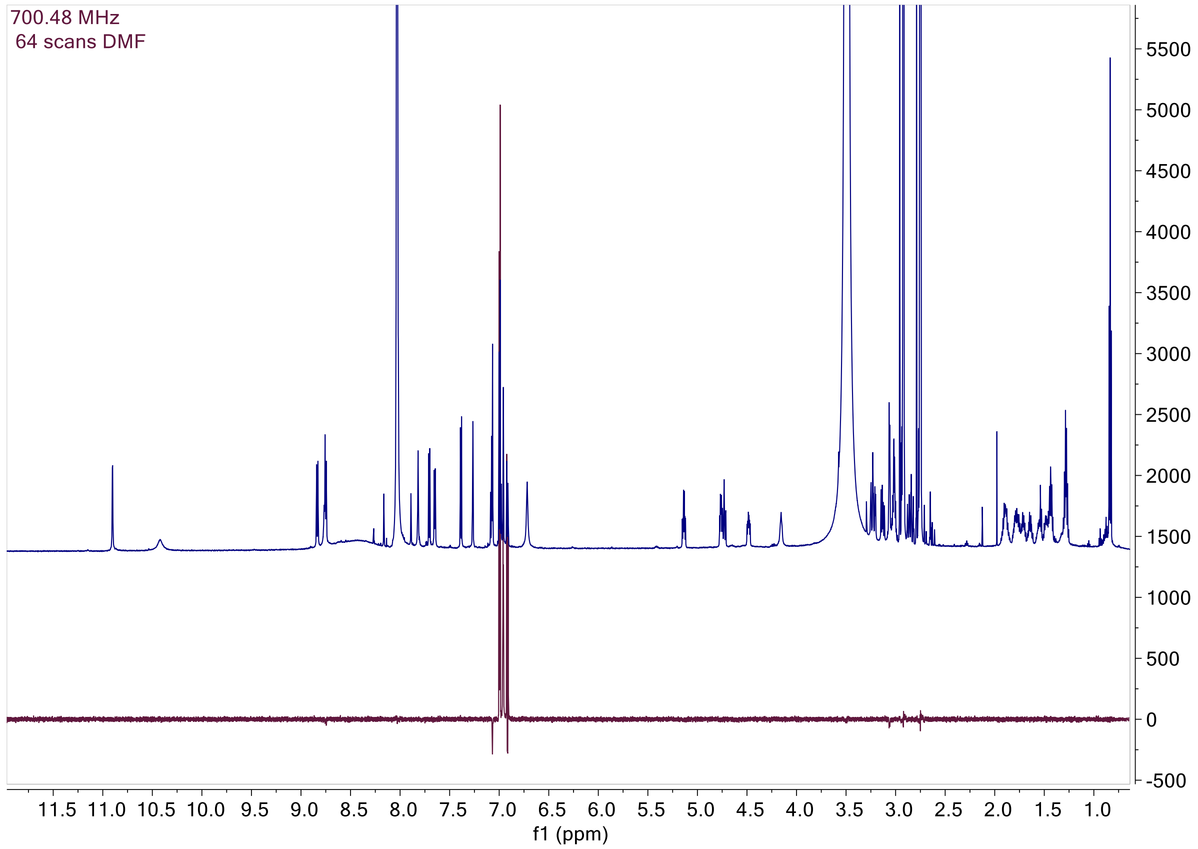

Figure S93. 1D ROESY spectrum of **Nle-2** in DMF-d_7 stacked with ^{1}H spectrum.

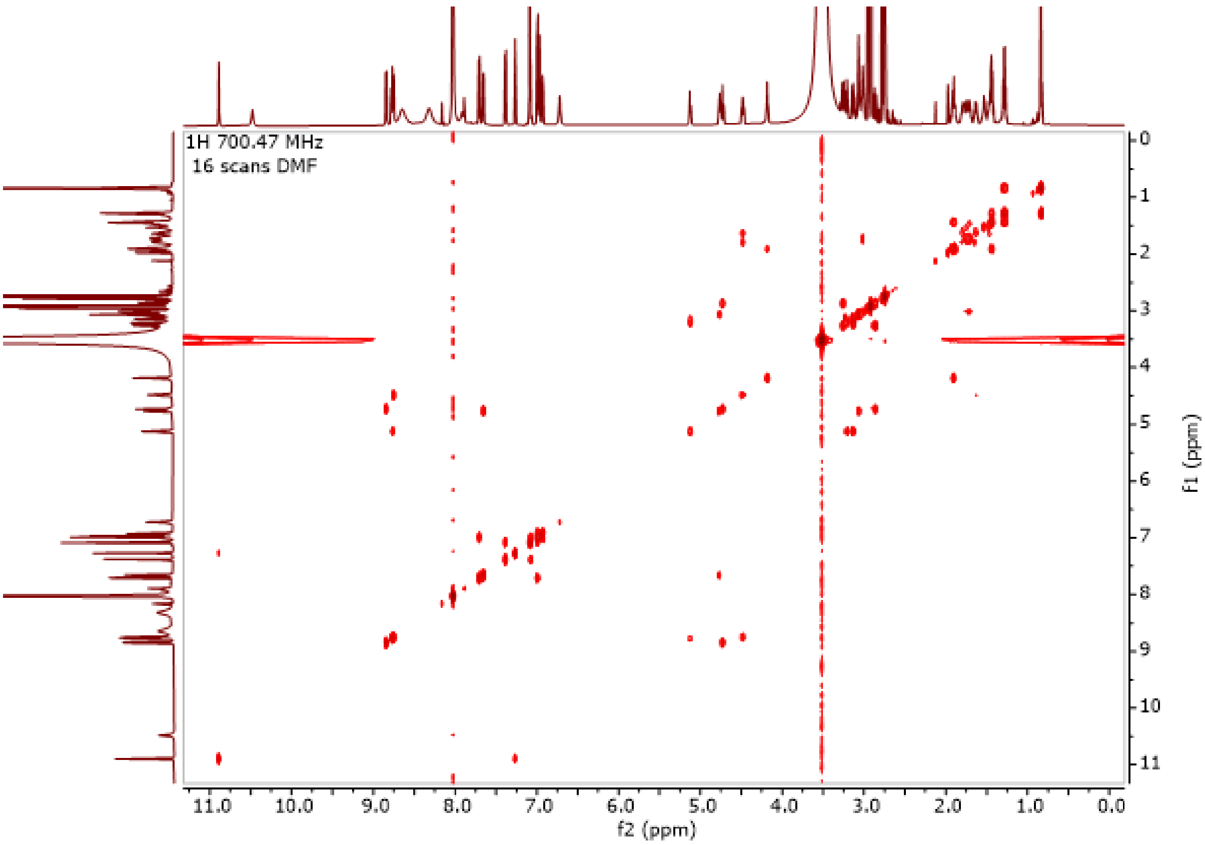

Figure S94. COSY spectrum of **Nle-2** in DMF-d_7

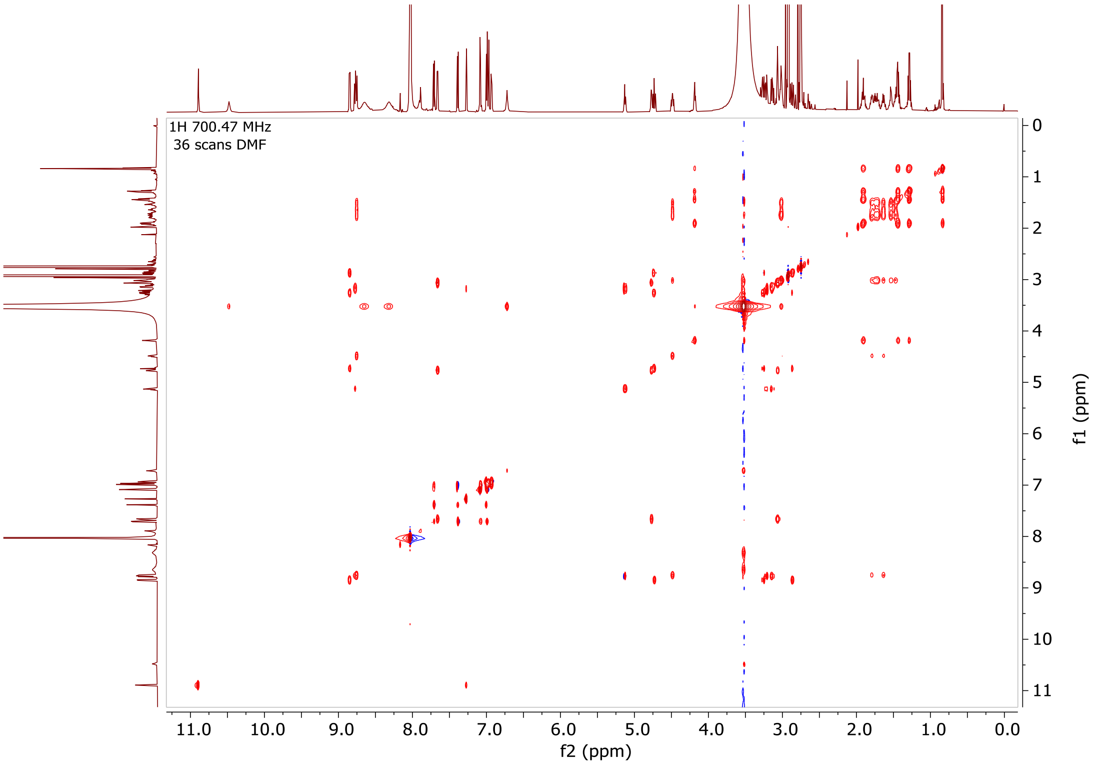

Figure S95. 2D TOCSY spectrum of **Nle-2** in DMF-d_7. Mixing time 80 ms.

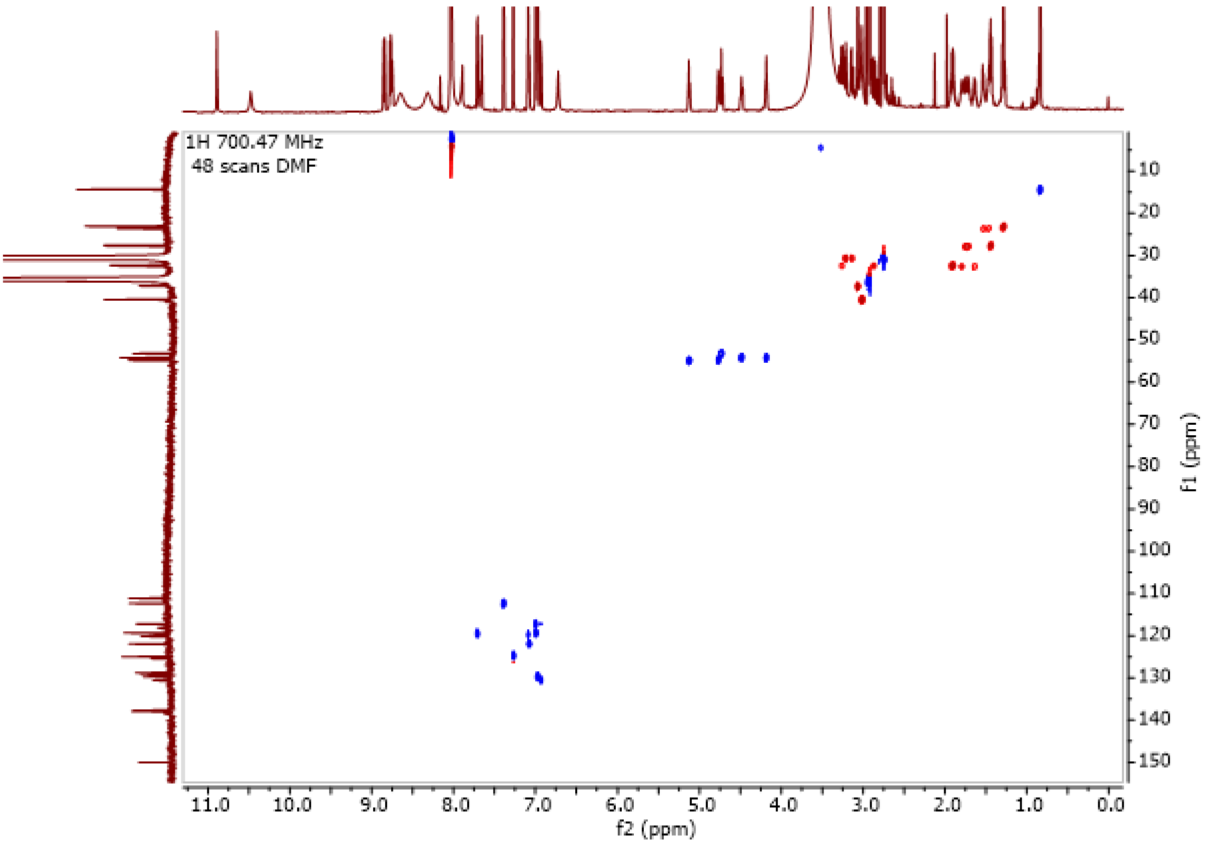

Figure S96. ^{13}C-HSQC Edit spectrum of **Nle-2** in DMF-d_7 optimised for $^{1}J_{CH}$ = 145 Hz.

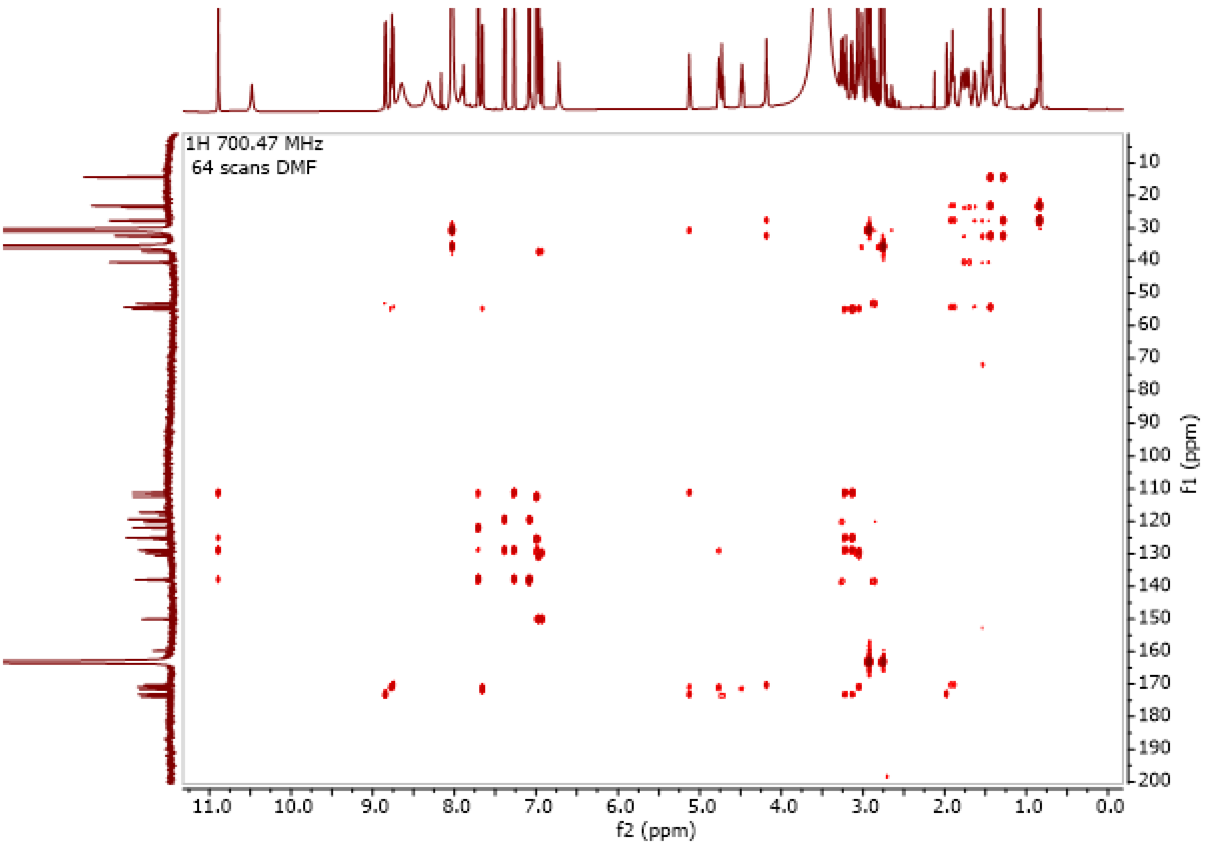

Figure S97. ^{13}C-HMBC spectrum of **Nle-2** in DMF-d_7

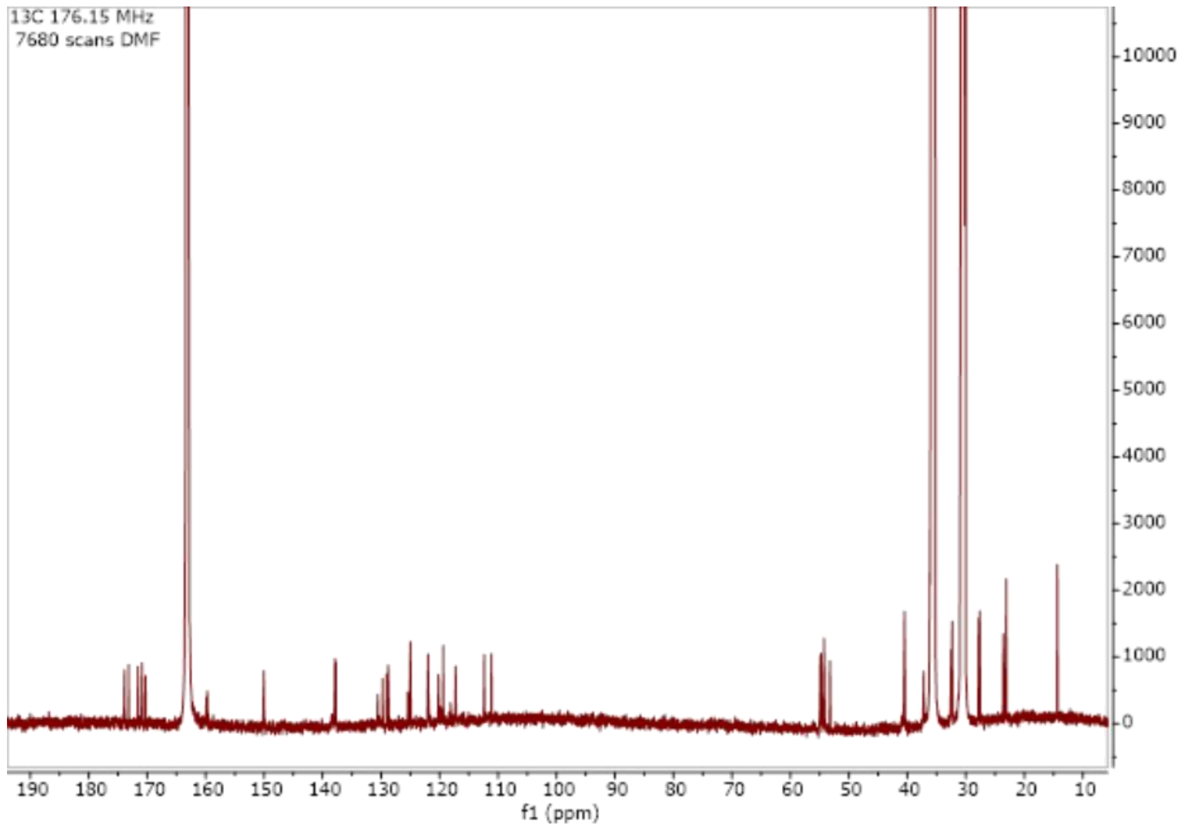

Figure S98. ^{13}C spectrum of **Nle-2** in DMF-d_7

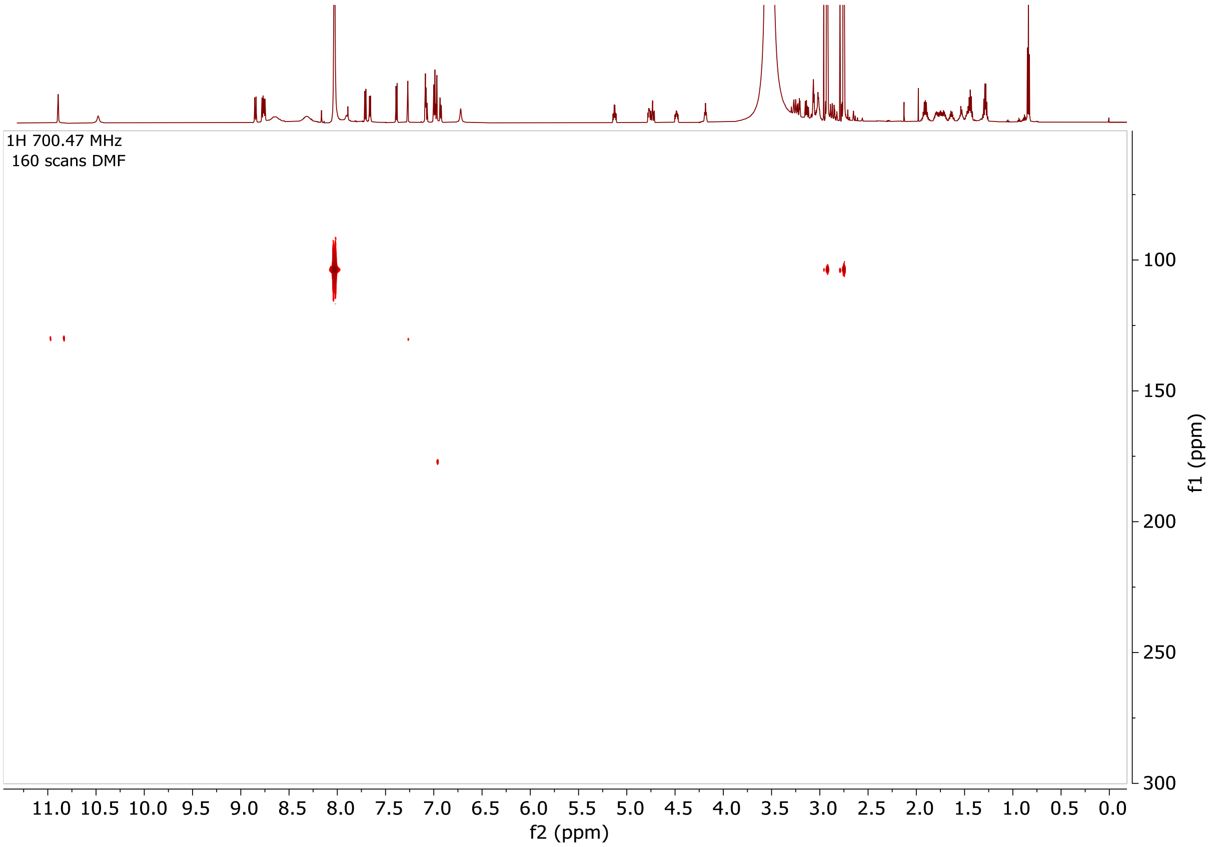

Figure S99. ^{15}N-HMBC spectrum of **Nle-2** in DMF-d_7 optimised for $^3J_{NH}$ = 4 Hz. Note that multiple $^1J_{NH}$ correlations are visible in the spectrum due to the absence of low-pass filter in the pulse sequence.

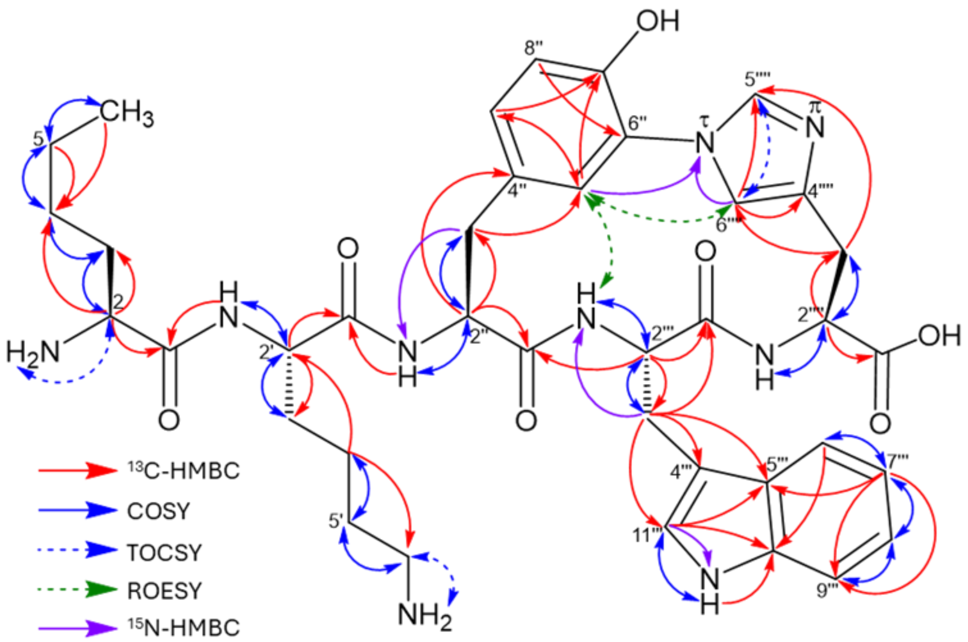

Figure S100. Key NMR correlations used in the characterisation of **Nle-2**.

Table S2. NMR data for crosslinked **Nle-2**

	Obs. DMF-d7<br>700 MHz δ_H (*J*)	176 MHz δ_C	71 MHz δ_N
**Norleucine**			
**1**	-	170.3	
**2**	4.18 (t, 6.5, br)	54.3	
**2-NH**	8.65 (s, br)		Not. Obs. [a]
**3**	1.92 (dq, 14.0, 7.0, ov) [a] 1.89 (dq, 14.0, 7.0, ov)[b]	32.3	
**4**	1.44 (pent, 7.4, ov)	27.6	
**5**	1.28 (sext, 7.4)	23.1	
**6**	0.84 (t, 7.4)	14.4	
**Lysine**			
**1’**	-	171.6	
**2’**	4.48 (td, 8.8, 4.4)	54.2	
**2’-NH**	8.75 (d, 8.8)		120.1
**3’**	1.61 - 1.67 (m), 1.76-1.82 (m, ov)	32.6	
**4’**	1.43 - 1.48 (m, ov), 1.51-5.56 (m)	23.6	
**5’**	1.68 - 1.78 (m, ov)	27.8	
**6’**	2.98 - 3.30 (m)	40.5	
**6’-NH**	8.32 (s, br)		Not Obs.[c]
**Tyrosine**			
**1”**	-	170.9	
**2”**	4.77 (dt, 7.5, 4.4)	54.8	
**2”-NH**	7.65 (d, 7.3)		114.0
**3”**	3.03 -3.08 (m, ov)	37.2	

**4″**	-	129.0	
**5″**	6.97 (d, 2.2)	129.7	
**6″**	-	125.4	
**7″**	-	150.0	
**7″-OH**	10.48 (s)[d]		
**8″**	6.99 (d, ov, 8.2)	117.3	
**9″**	6.93 (dd, 8.2, 2.2)	130.6	
**Tryptophan**			
**1‴**	-	173.1	
**2‴**	5.12 (td, 8.7, 4.9)	54.9	
**2‴-NH**	8.78 (d, 8.7)		121.1
**3‴**	3.22 (dd, 14.7, 4.9), 3.13 (14.7, 8.7)	31.0	
**4‴**	-	111.1	
**5‴**	-	128.8	
**6‴**	7.71 (d, 7.5, br)	119.5	
**7‴**	6.98 (dt, 7.5, 1.0)	119.4	
**8‴**	7.08 (dt, 7.5, 1.0)	121.9	
**9‴**	7.38 (dt, 7.5, 1.0)	112.4	
**10‴**	-	137.7	
**11‴**	7.27 (d, 2.0)	125.0	
**11‴-NH**	10.89 (d, 2.0)		129.9
**Histidine**			
**1⁗**	-	173.9	
**2⁗**	4.73 (ddd, 12.2, 9.5, 2.6)	53.2	
**2⁗-NH**	8.85 (d, 9.5)		119.1
**3⁗**	3.26 (dd, 14.7, 2.6), 2.87 (dd, 4.7, 12.2)	32.3	
**4⁗**	-	137.9	
**5⁗**	7.91 (s, br)	138.2	
**5⁗-N**			177.0
**6⁗**	7.09 (s, br, ov)	120.2	

a. When measured in DMF-d$_7$ + 0.1% trifluoroacetic acid this signal was observed at δ_N 288.0 in a ^{15}N-HMBC experiment optimised for $^3J_{NH}$ = 4 Hz

b. Two overlapped signals. ABX$_2$Y system appearing as a nine-peak signal.

c. When measured in DMF-d$_7$ + 0.1% trifluoroacetic acid this signal was observed at δ_N 282.6 in a ^{15}N-HMBC experiment optimised for $^3J_{NH}$ = 4 Hz

d. No correlations observed, exchangeable proton. Assignment by chemical shift.

Ov indicates an overlapped signal, br indicates a broad signal.

7. Analysis of crosslinking reactions with AciB

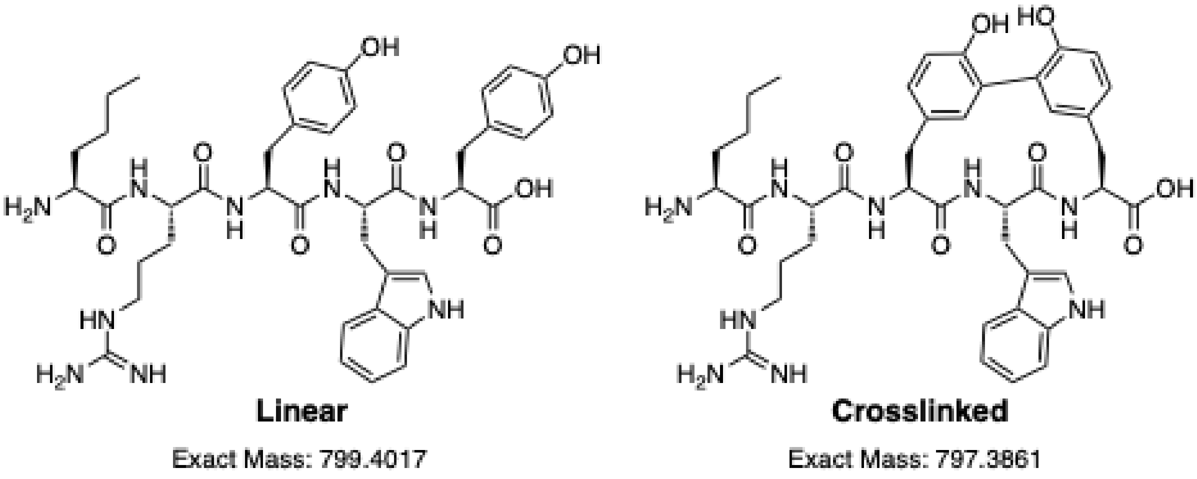

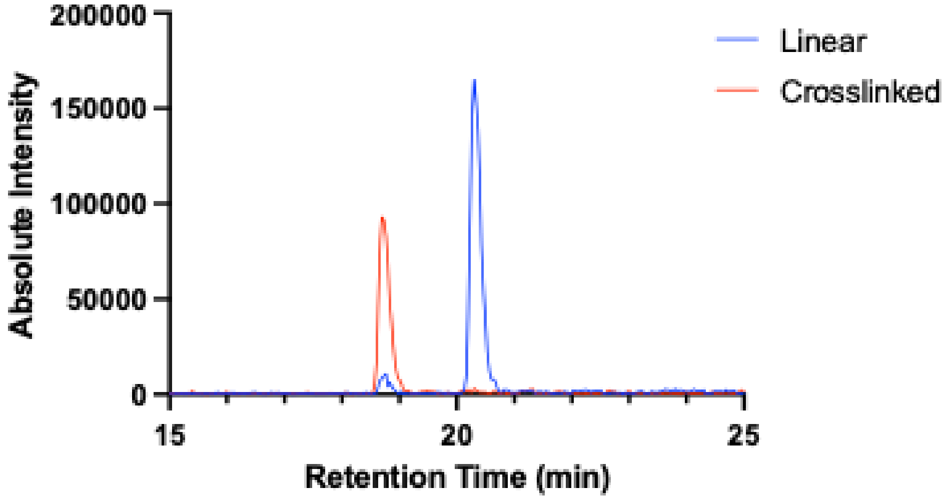

Figure S101. Crosslinking assay data for **Nle-3-(Trp4)** with AciB. Structures of linear and crosslinked peptides shown.

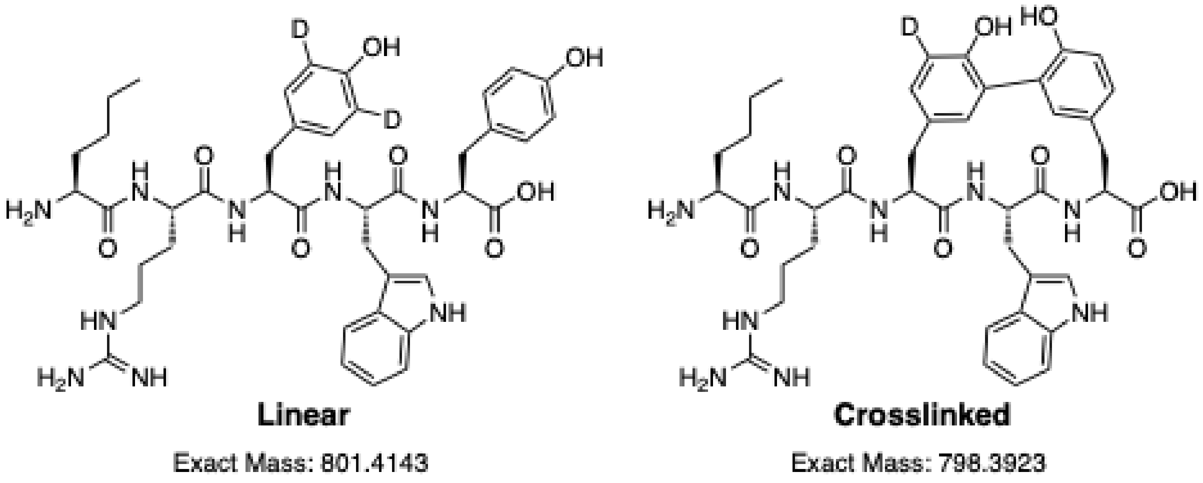

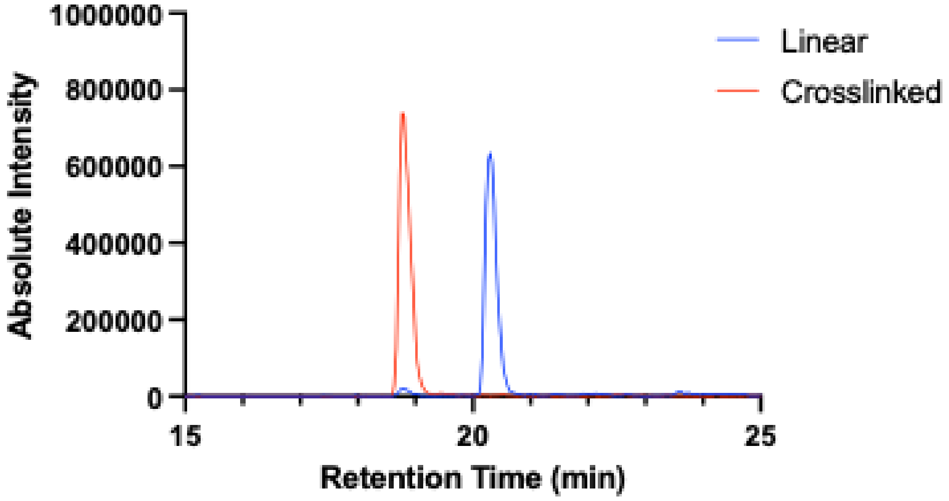

Figure S102. Crosslinking assay data for **Nle-3-(3,5-d$_2$-Tyr3, Trp4)** with AciB. Structures of linear and crosslinked peptides shown.

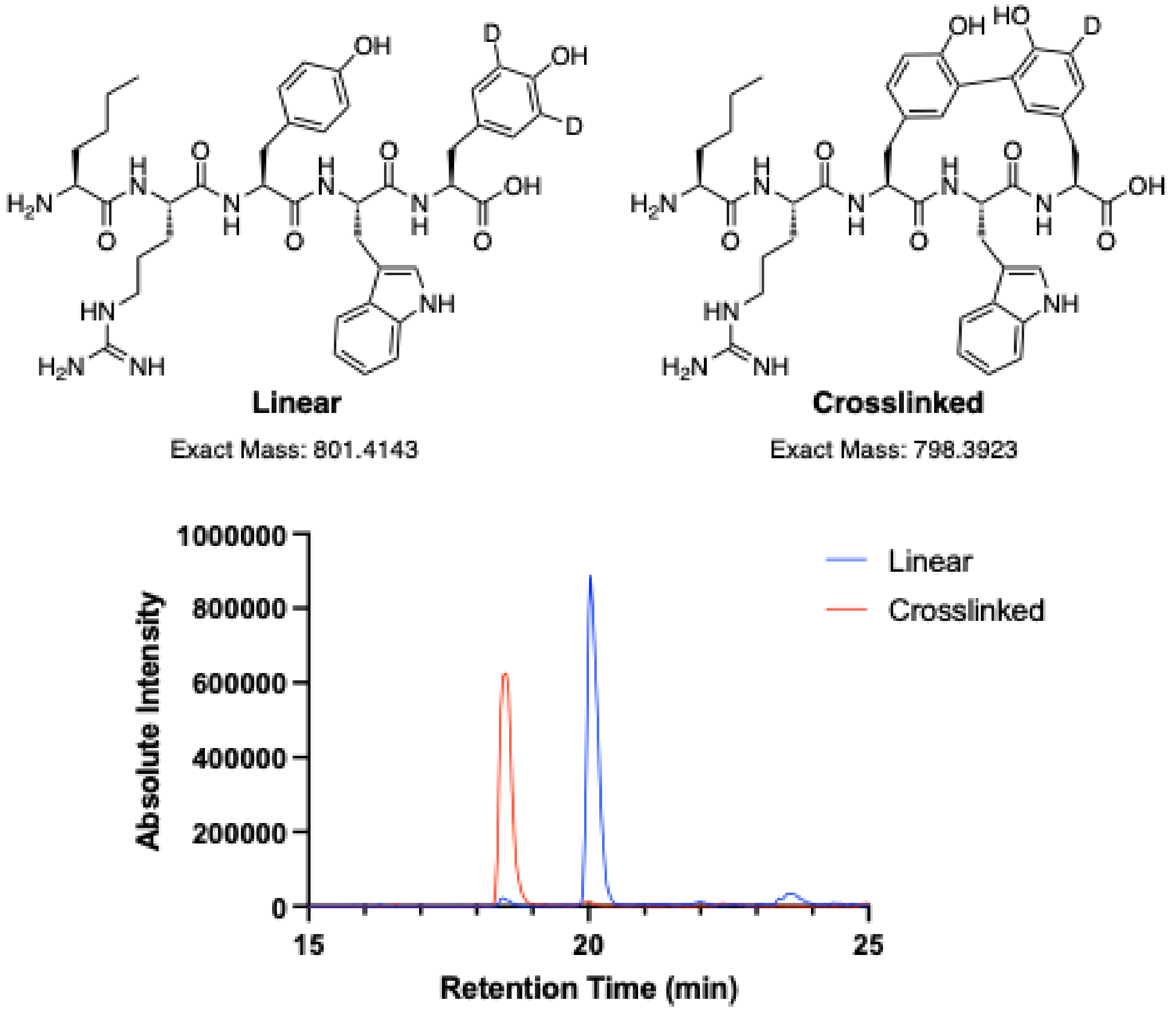

Figure S103. Crosslinking assay data for **Nle-3-(Trp4, 3,5-d$_2$-Tyr5)** with AciB. Structures of linear and crosslinked peptides shown.

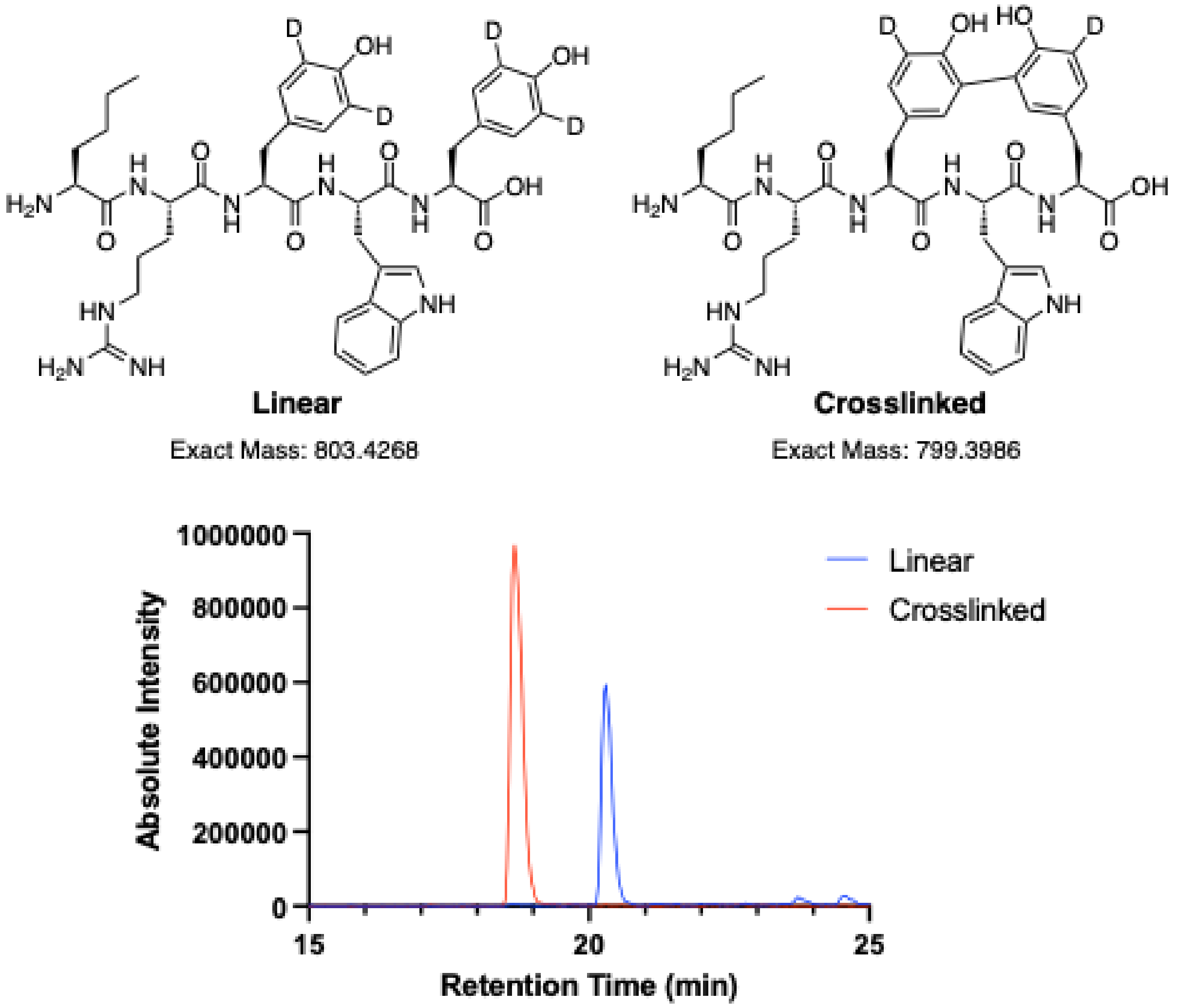

Figure S104. Crosslinking assay data for **Nle-3-(Trp4, 3,5-d$_2$-Tyr3,5)** with AciB. Structures of linear and crosslinked peptides shown.

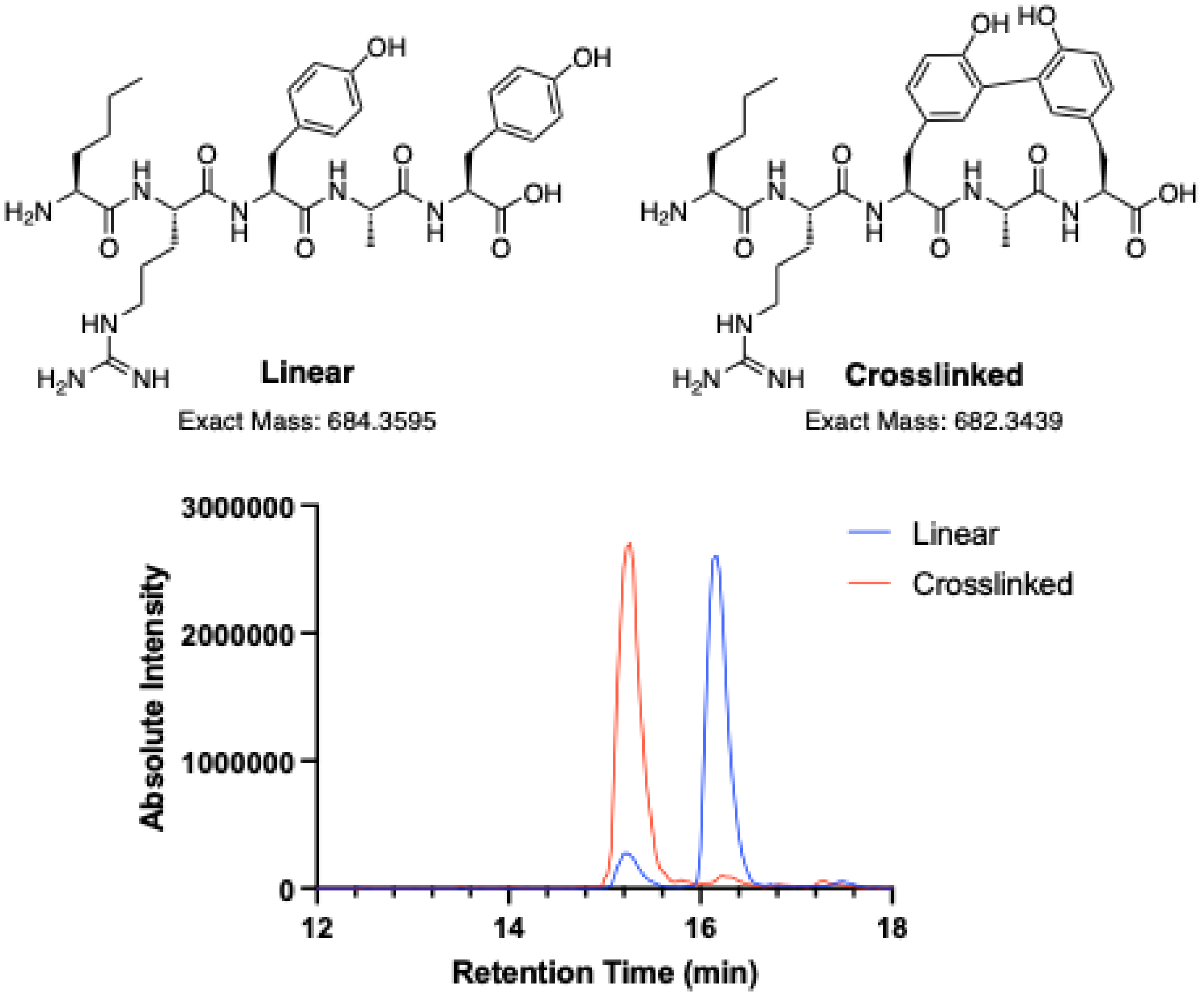

Figure S105. Crosslinking assay data for **Nle-3-(Ala4)** with AciB. Structures of linear and crosslinked peptides shown.

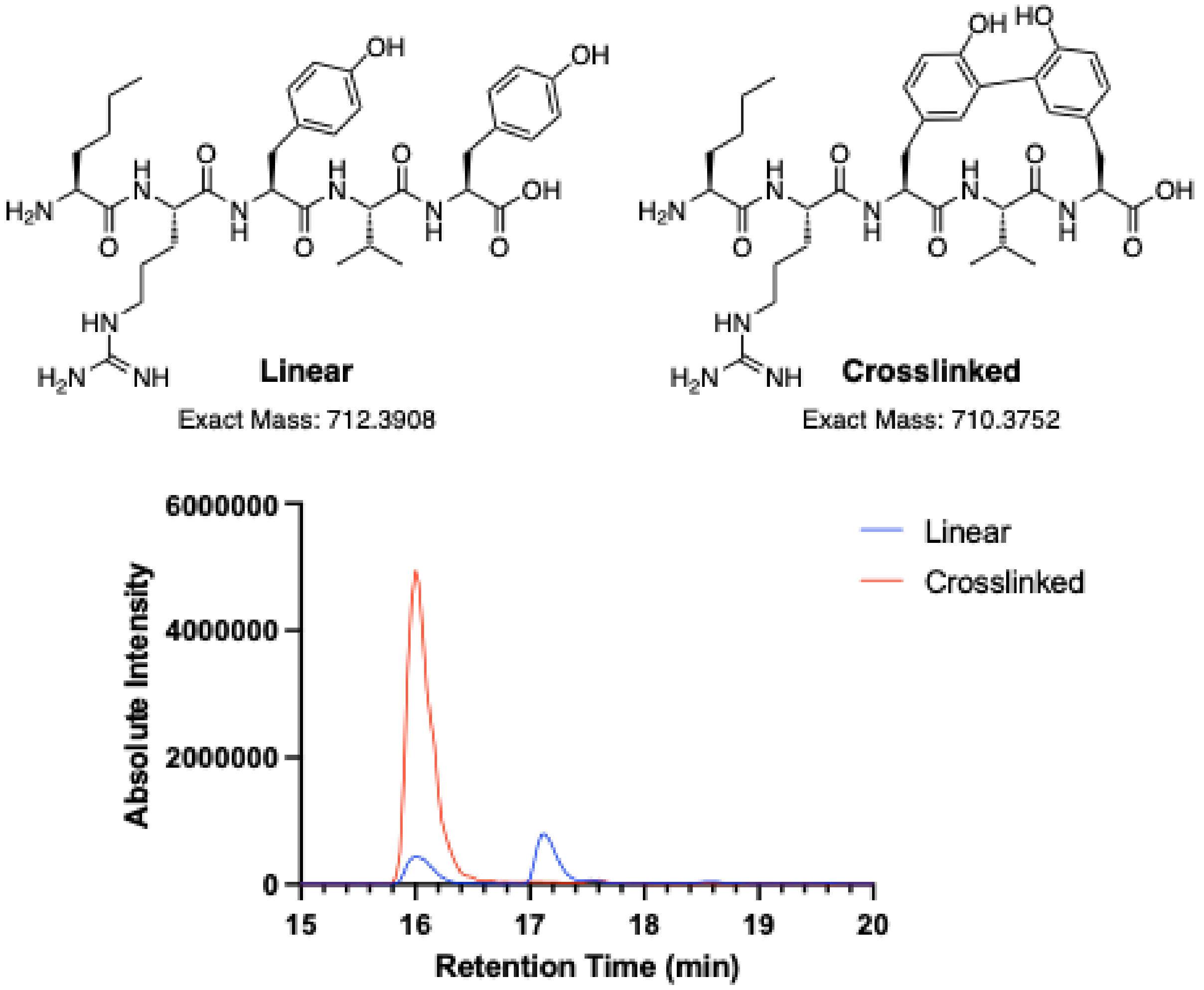

Figure S106. Crosslinking assay data for **Nle-3-(Val4)** with AciB. Structures of linear and crosslinked peptides shown.

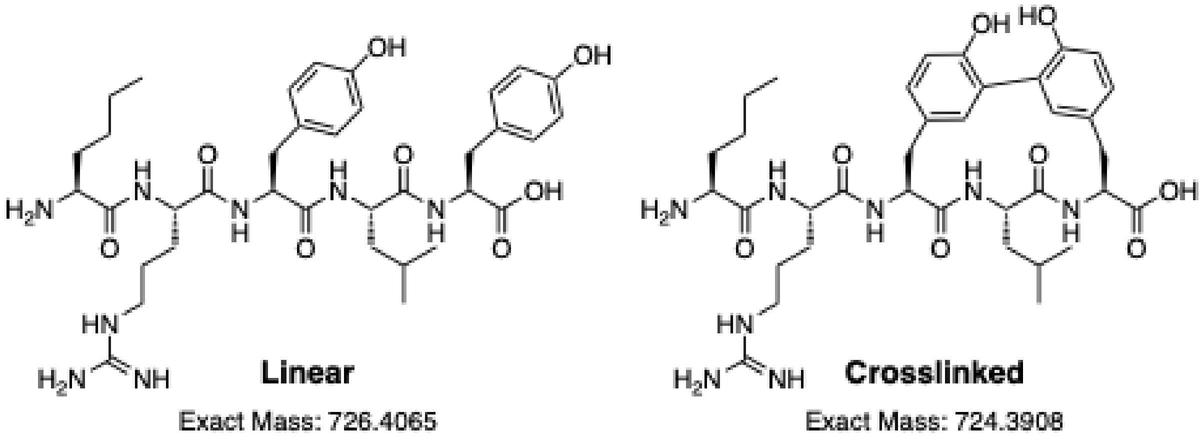

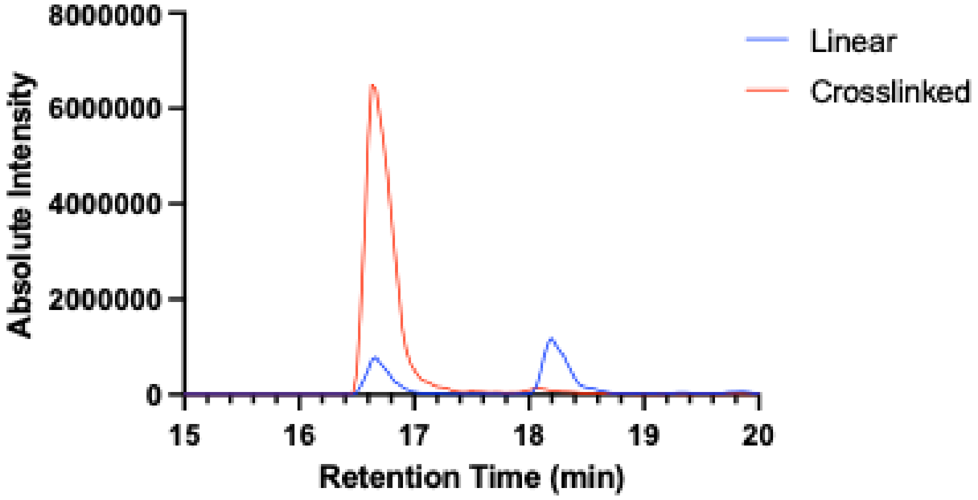

Figure S107. Crosslinking assay data for **Nle-3-(Leu4)** with AciB. Structures of linear and crosslinked peptides shown.

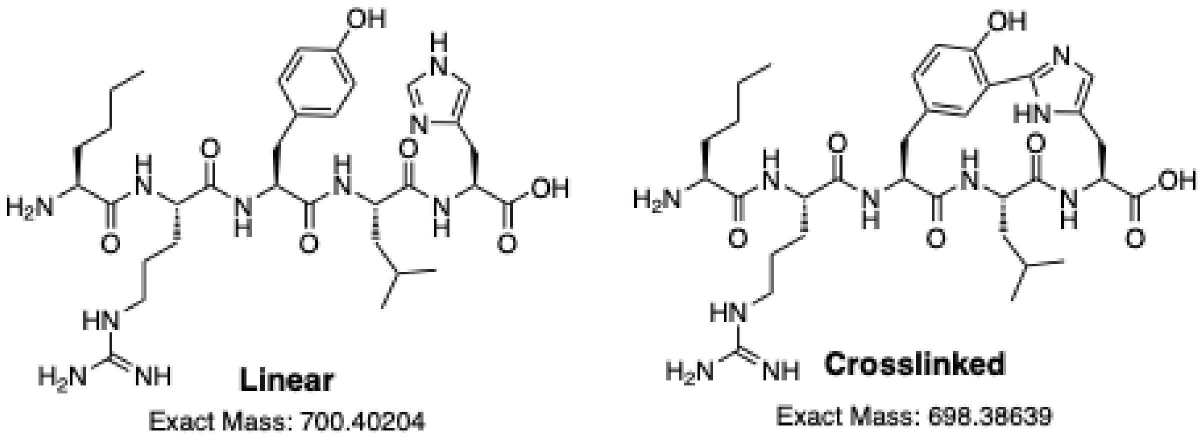

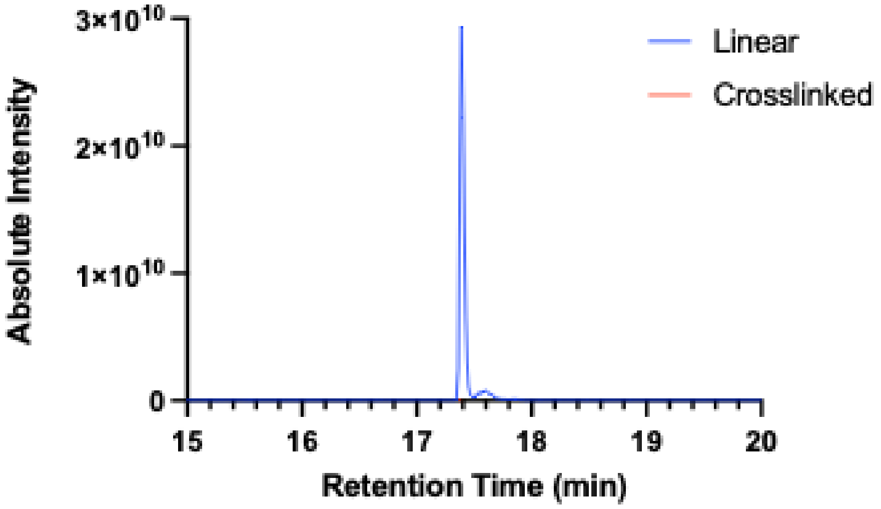

Figure S108. Crosslinking assay data for **Nle-3-(Leu4, His5)** with AciB. Structures of linear and crosslinked peptides shown.

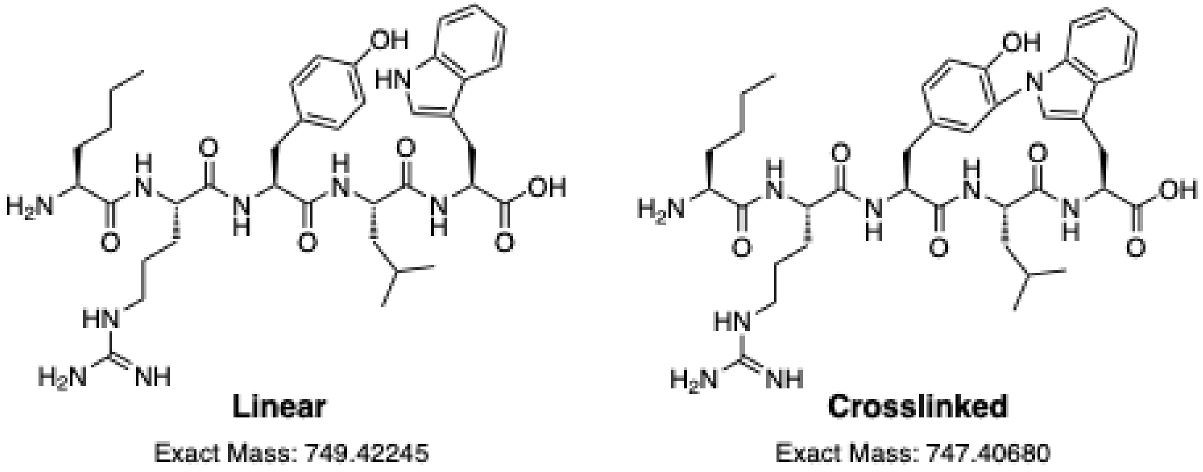

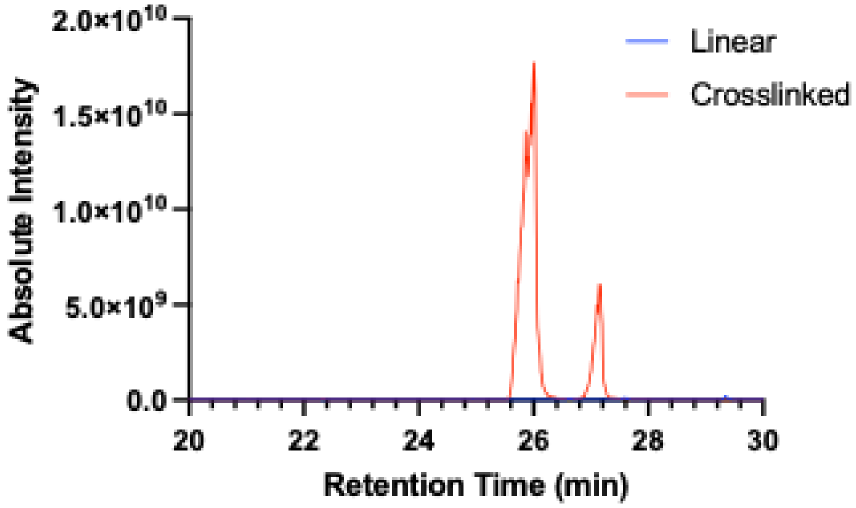

Figure S109. Crosslinking assay data for **Nle-3-(Leu4, Trp5)** with AciB. Structures of linear and crosslinked peptides shown.

8. Analysis of crosslinking reactions with SlyP

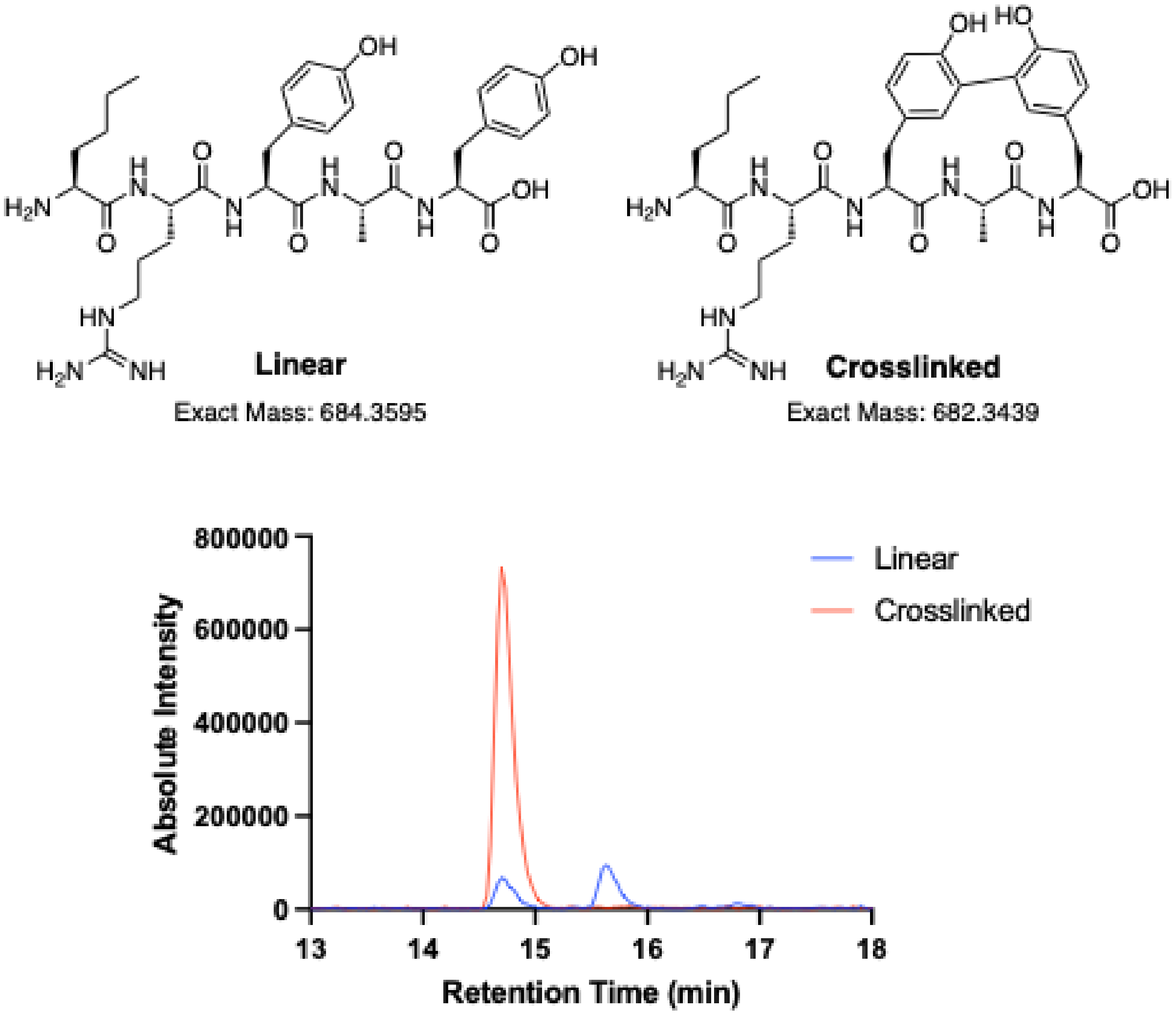

Figure S110. Crosslinking assay data for **Nle-3-(Ala4)** with SlyP. Structures of linear and crosslinked peptides shown.

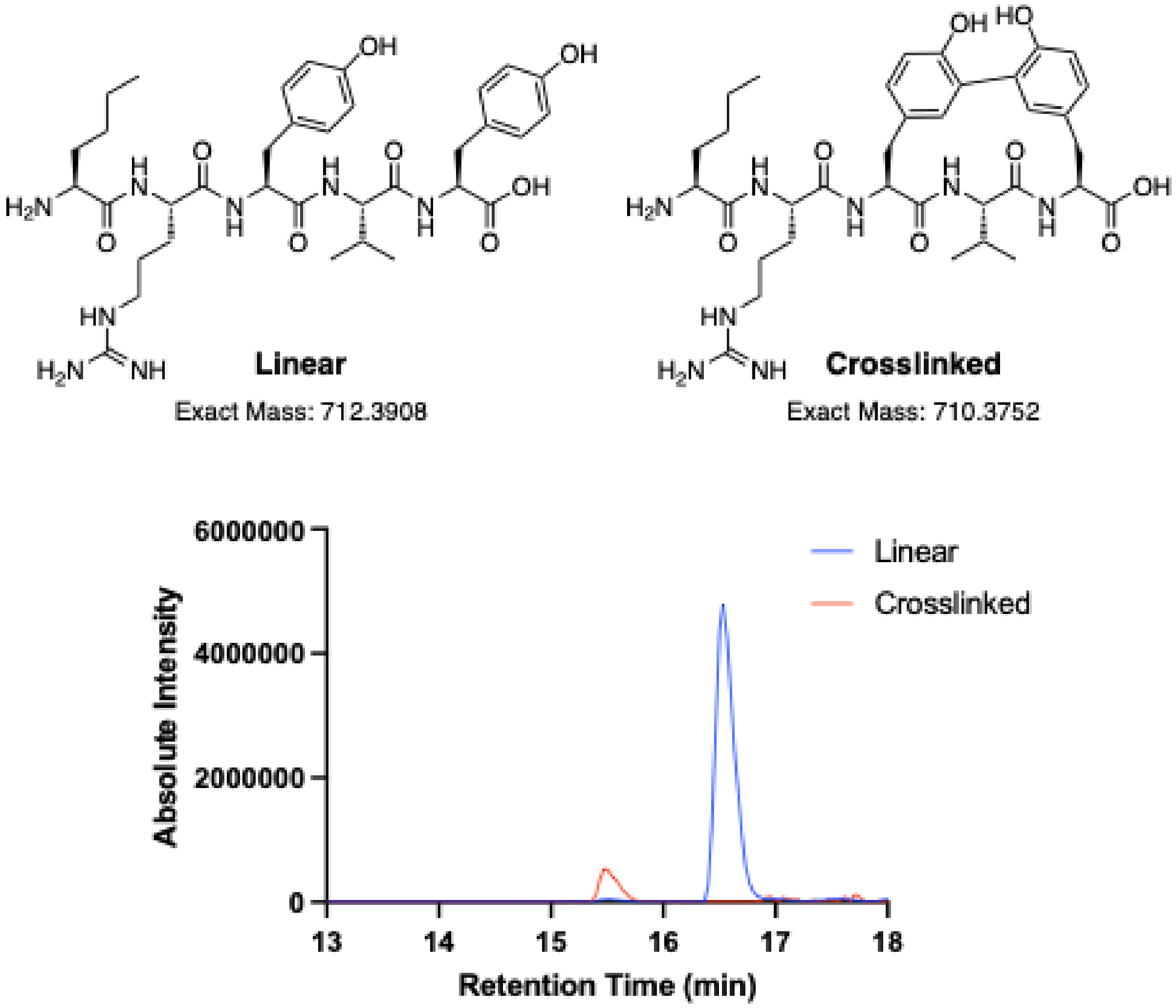

Figure S111. Crosslinking assay data for **Nle-3-(Val4)** with SlyP. Structures of linear and crosslinked peptides shown.

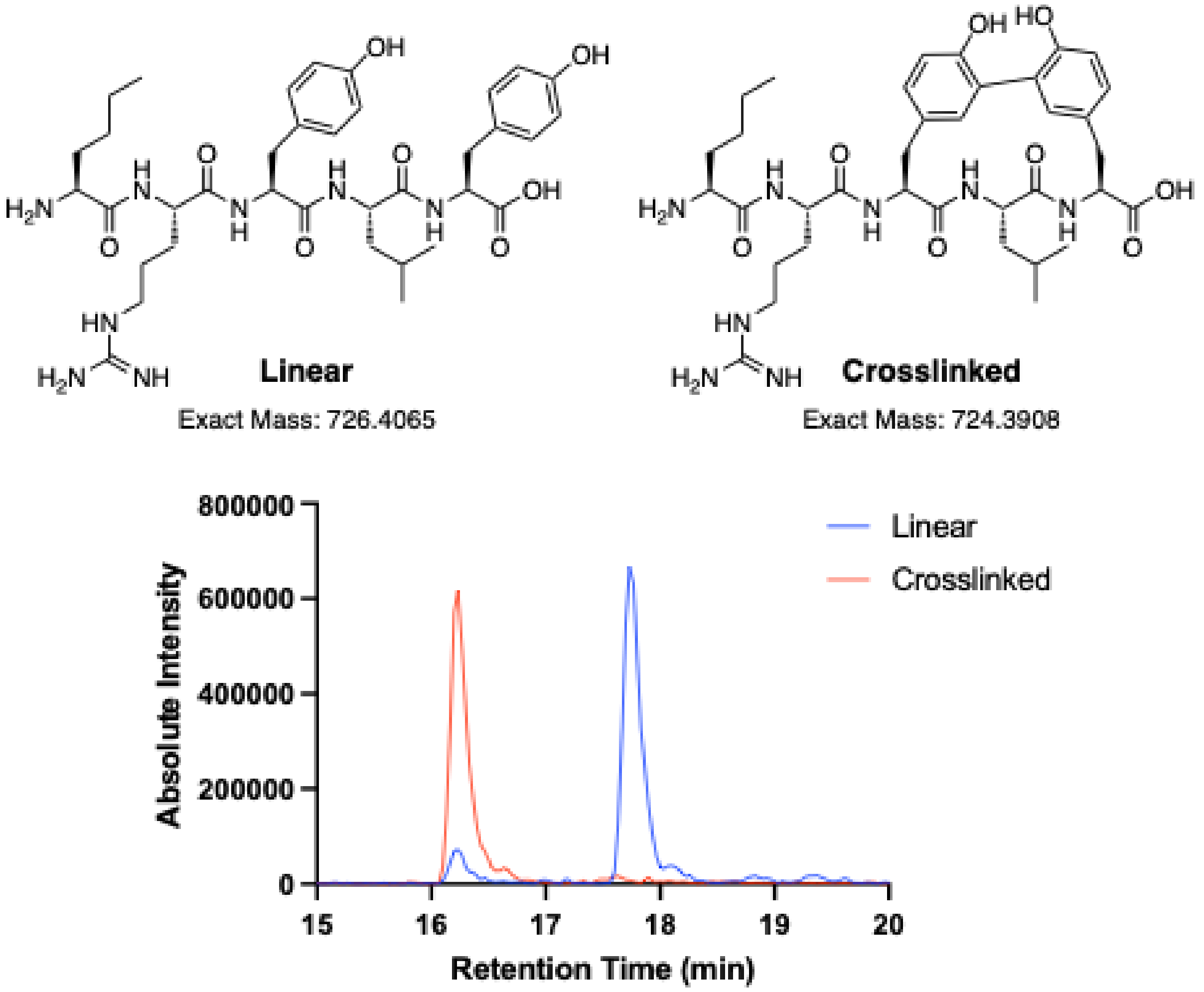

Figure S112. Crosslinking assay data for **Nle-3-(Leu4)** with SlyP. Structures of linear and crosslinked peptides shown.

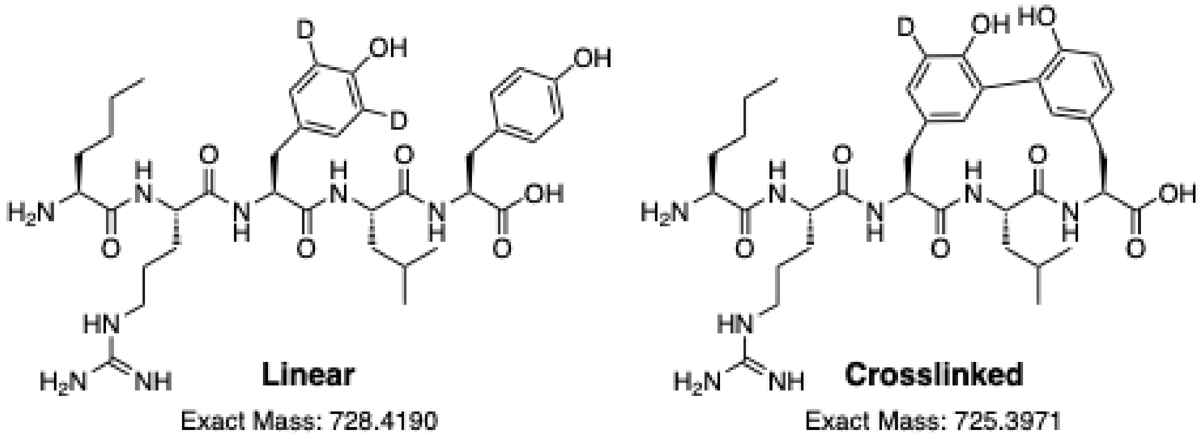

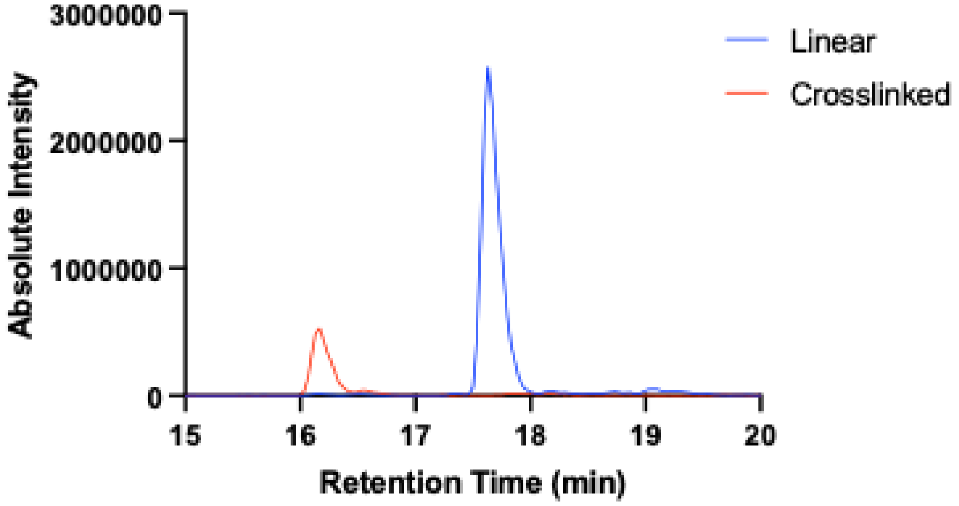

Figure S113. Crosslinking assay data for **Nle-3-(3,5-d$_2$-Tyr3, Leu4)** with SlyP. Structures of linear and crosslinked peptides shown.

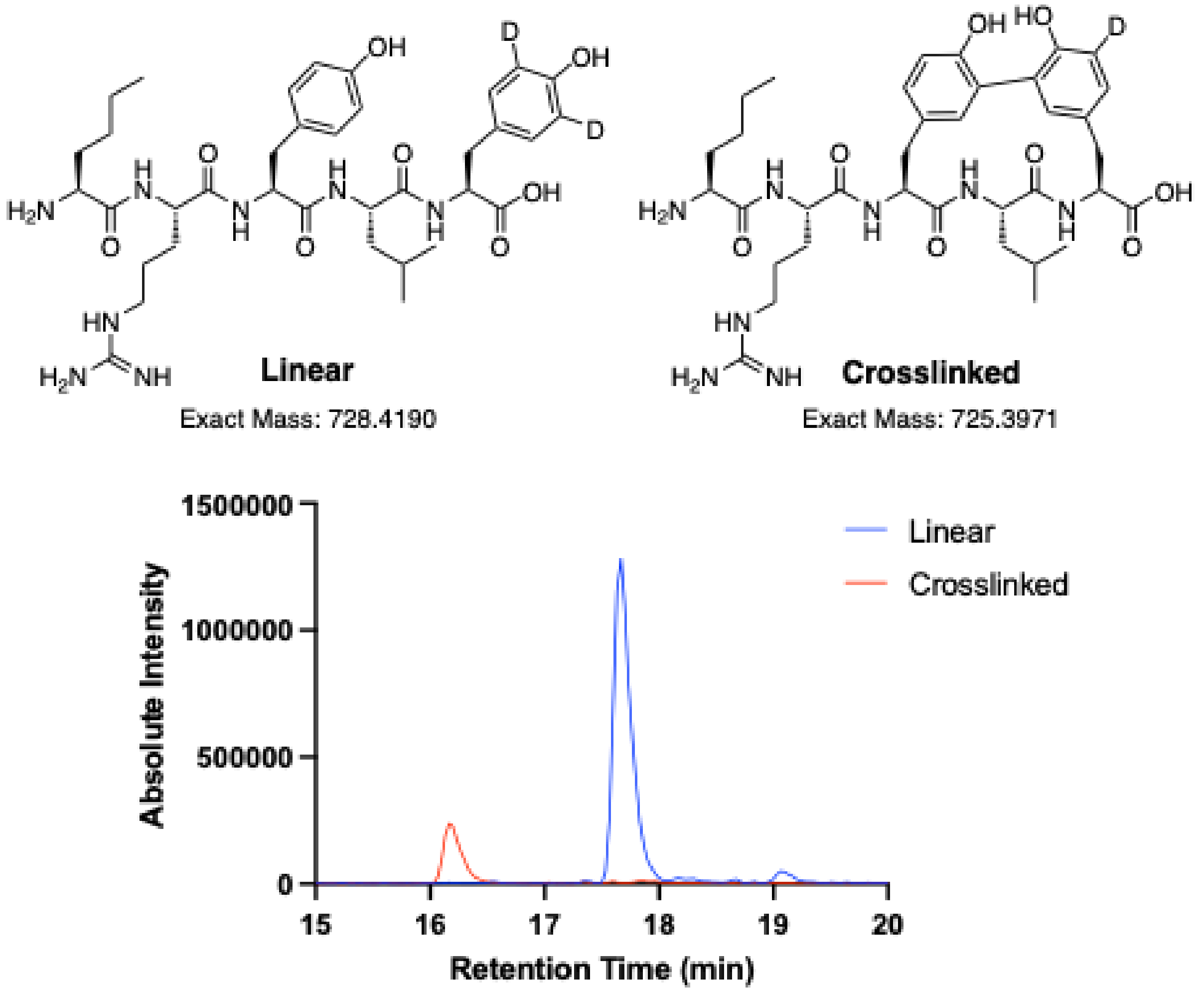

Figure S114. Crosslinking assay data for **Nle-3-(Leu4, 3,5-d$_2$-Tyr5)** with SlyP. Structures of linear and crosslinked peptides shown.

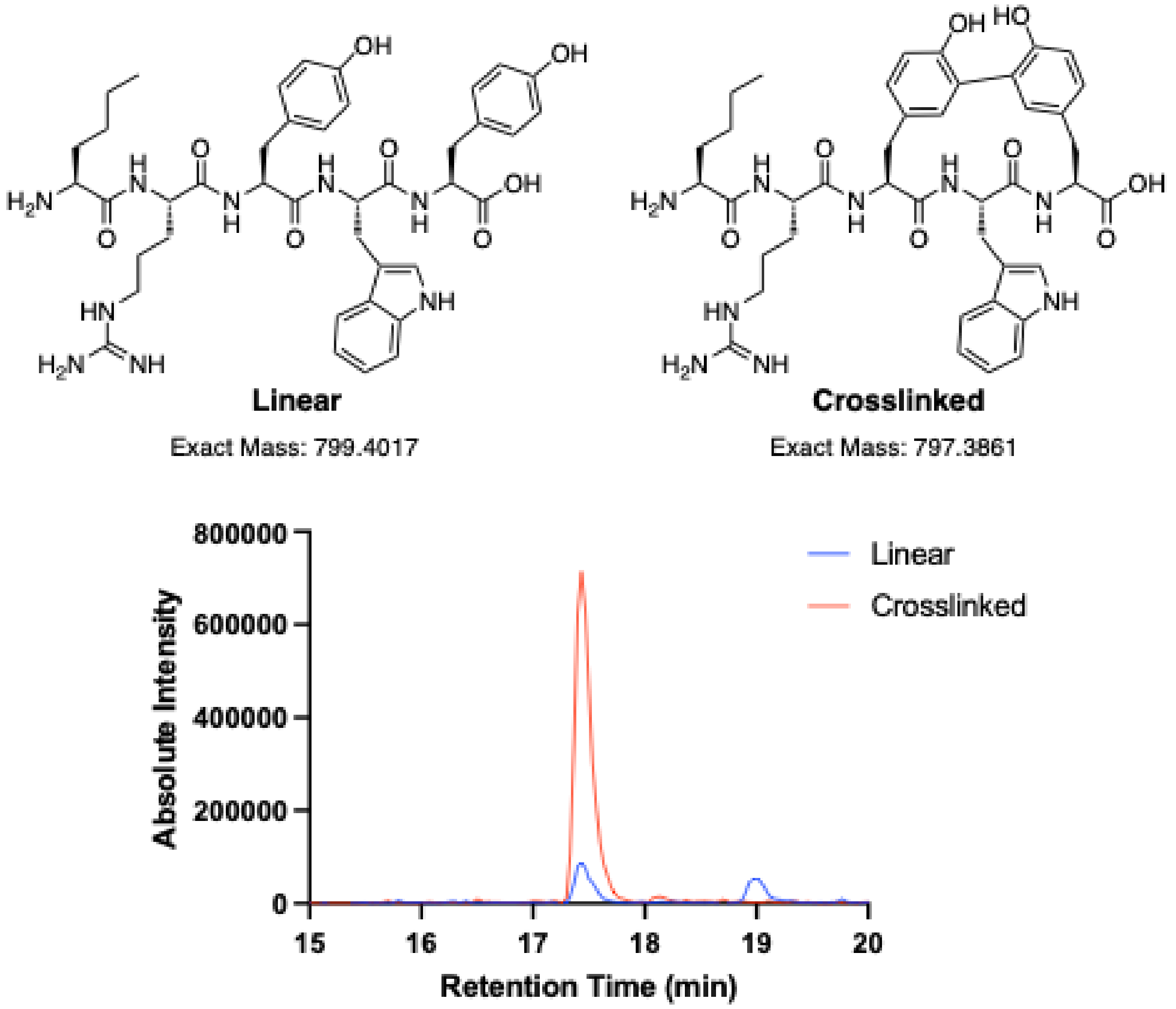

Figure S115. Crosslinking assay data for **Nle-3-(Trp4)** with SlyP. Structures of linear and crosslinked peptides shown.

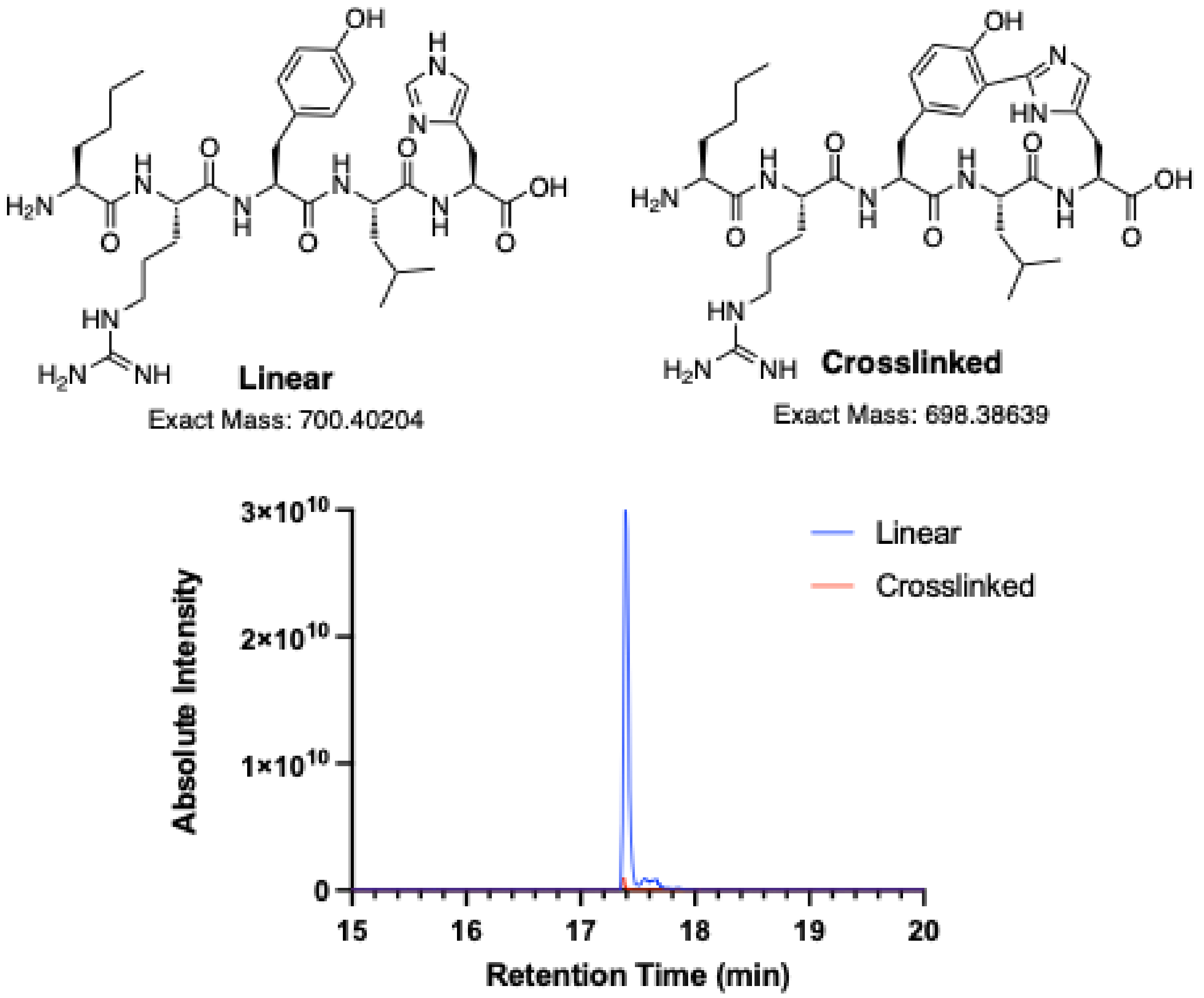

Figure S116. Crosslinking assay data for **Nle-3-(Leu4, His5)** with SlyP. Structures of linear and crosslinked peptides shown.

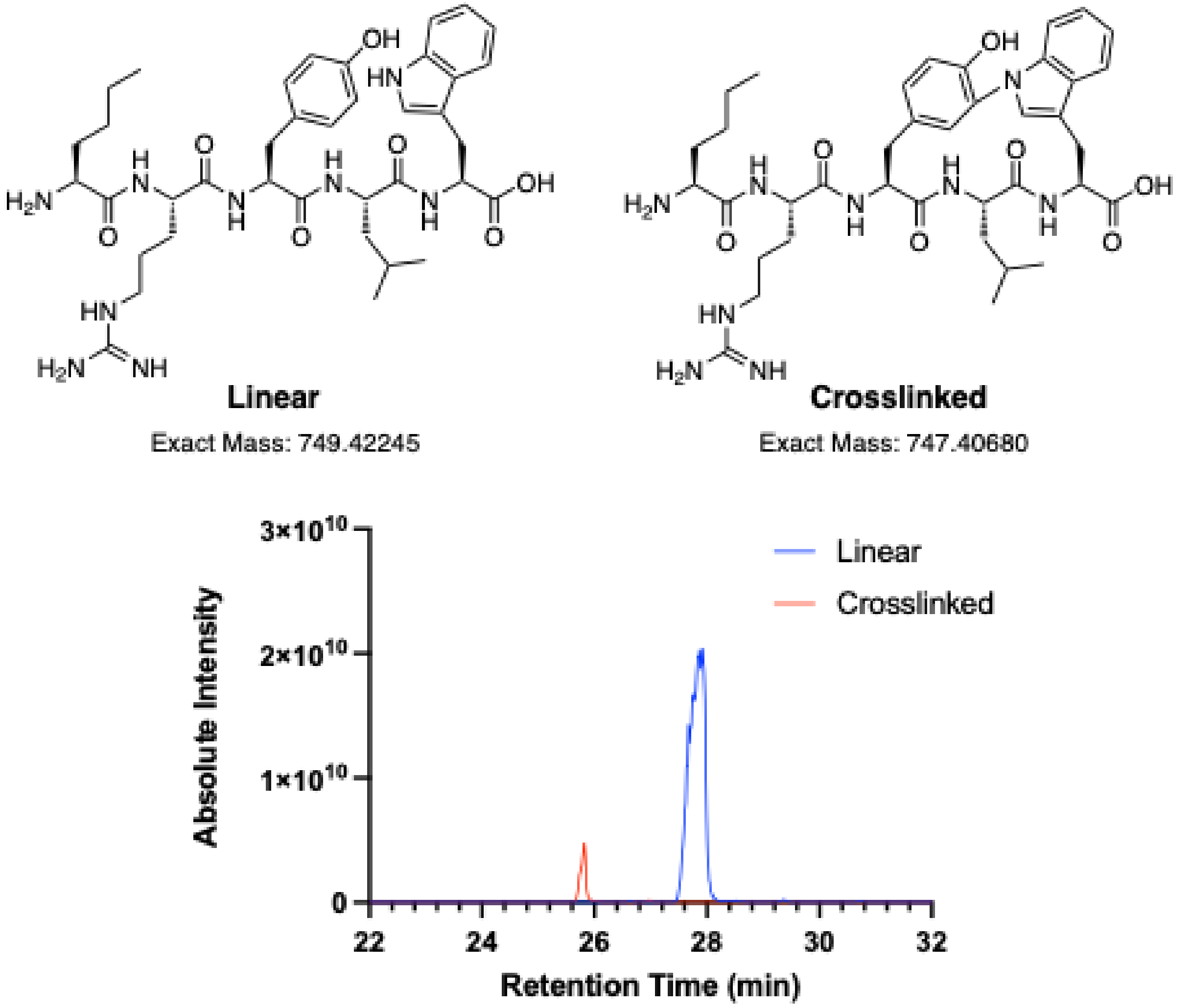

Figure S117. Crosslinking assay data for **Nle-3-(Leu4, Trp5)** with SlyP. Structures of linear and crosslinked peptides shown.

9. Structural Characterisation of Biarylitide YVH (5)

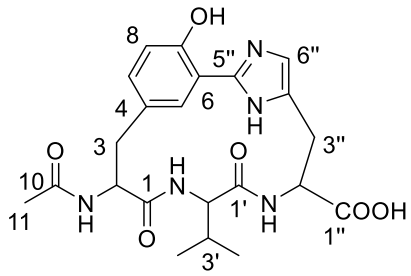

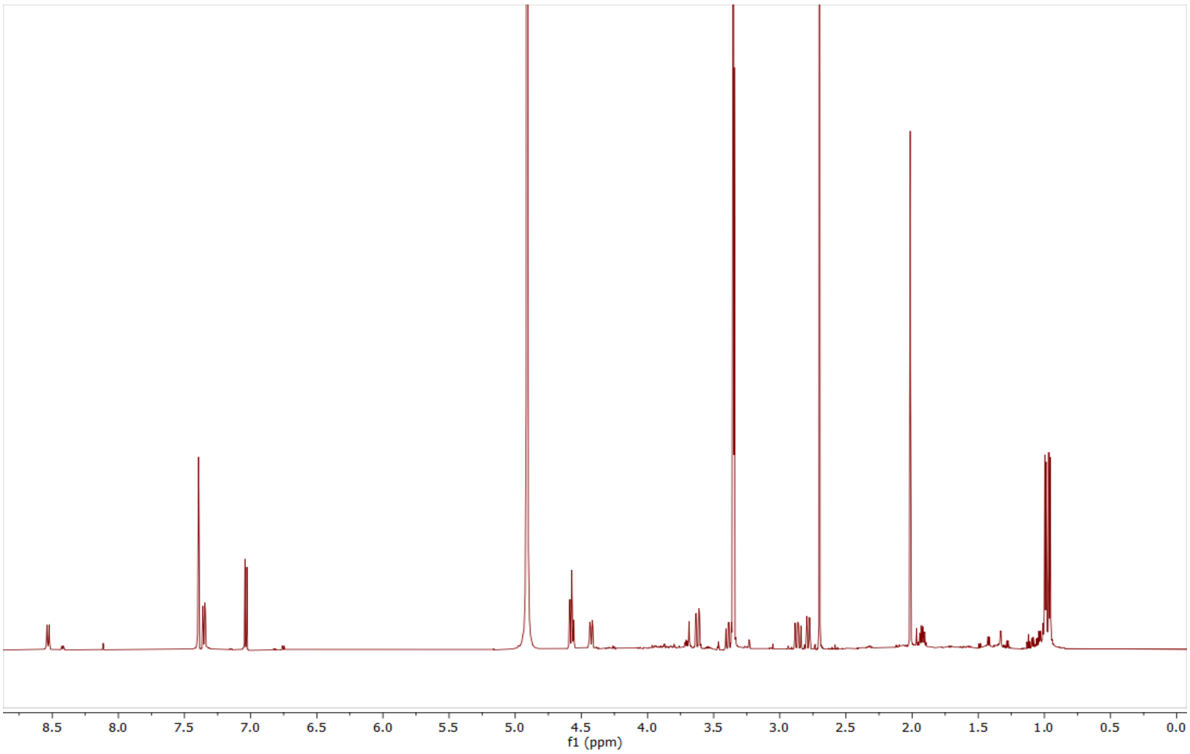

Figure S118. ^{1}H NMR spectrum of **5** in MeOH-d_4 (600 MHz).

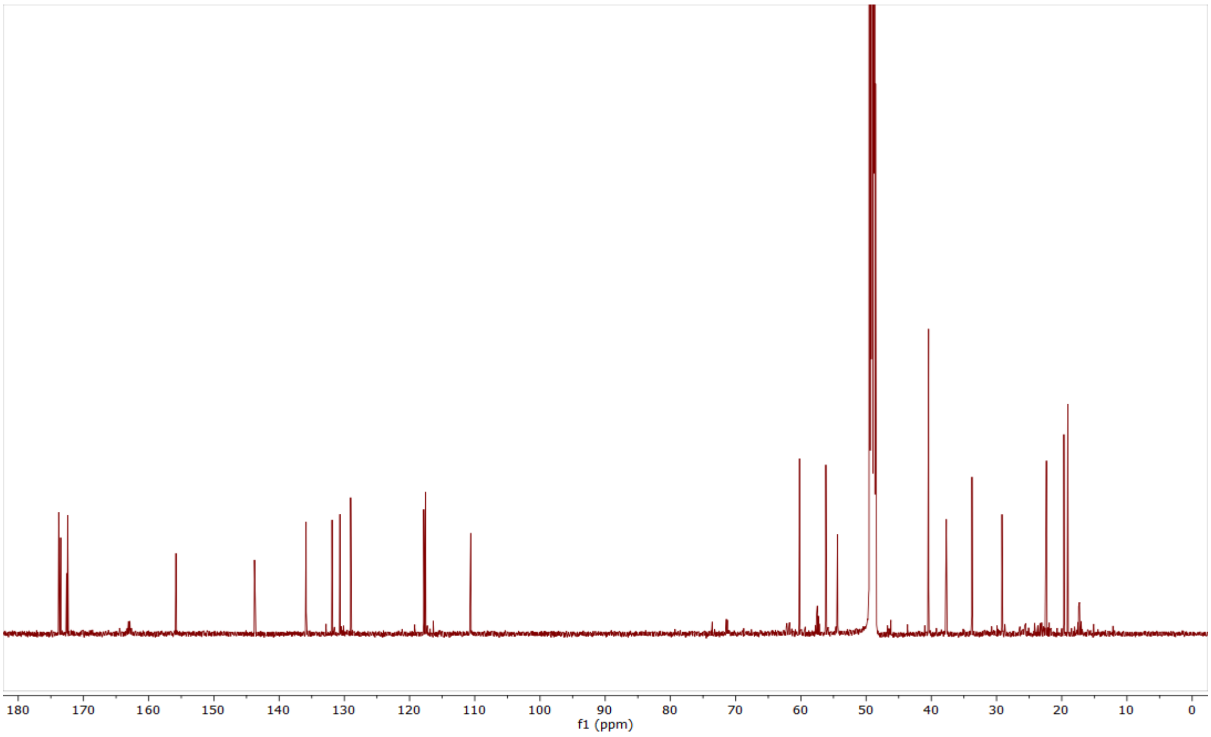

Figure S119. ^{13}C NMR spectrum of **5** in MeOH-d_4 (150 MHz).

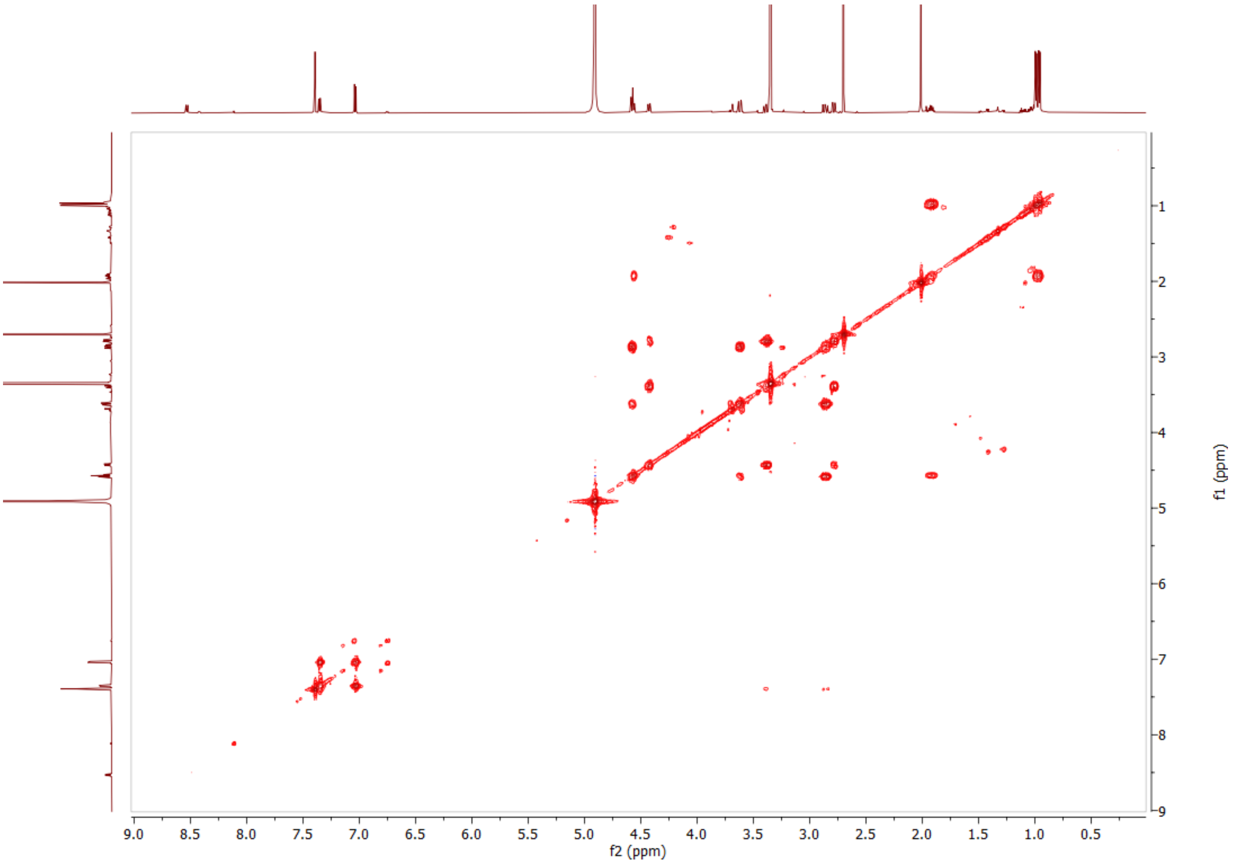

Figure S120. ^{1}H-^{1}H COSY spectrum of **5** in MeOH-d_4 (600 MHz).

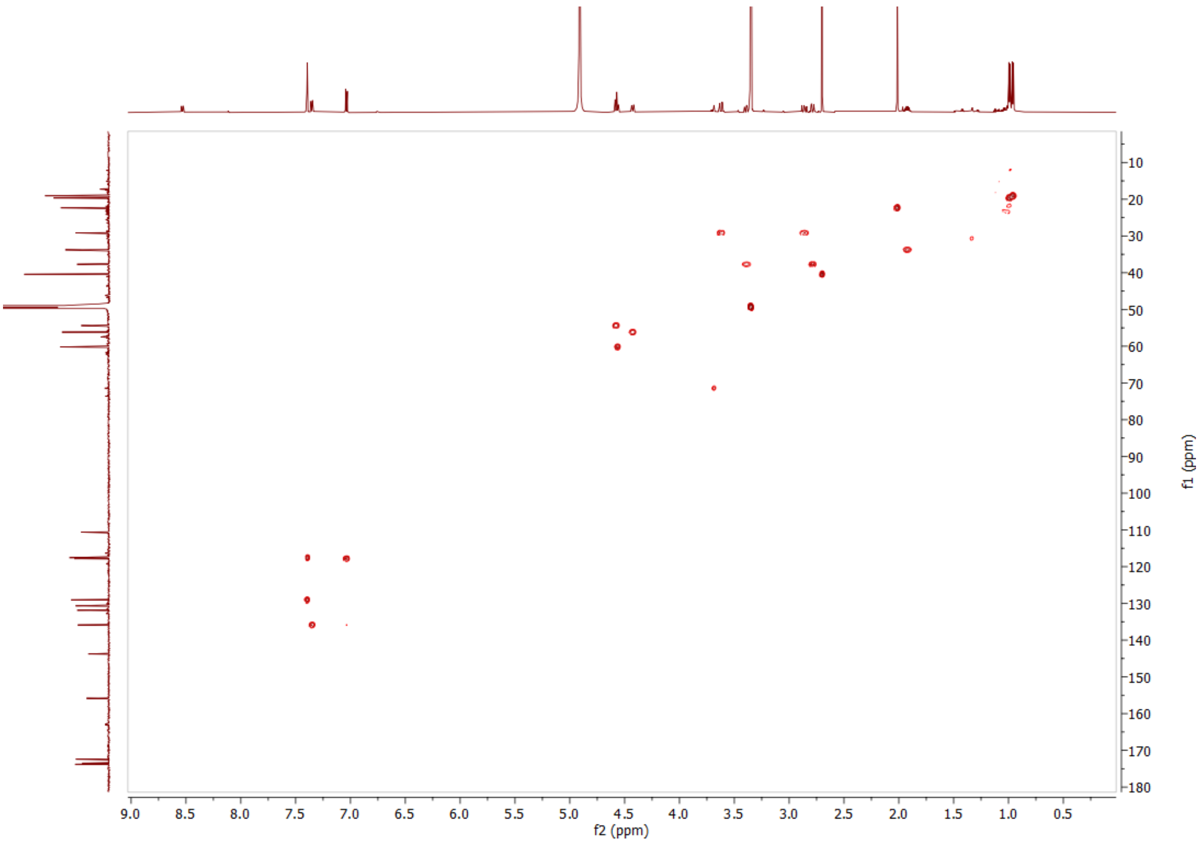

Figure S121. ^{1}H-^{13}C HSQC spectrum of **5** in MeOH-d_4 (600 MHz).

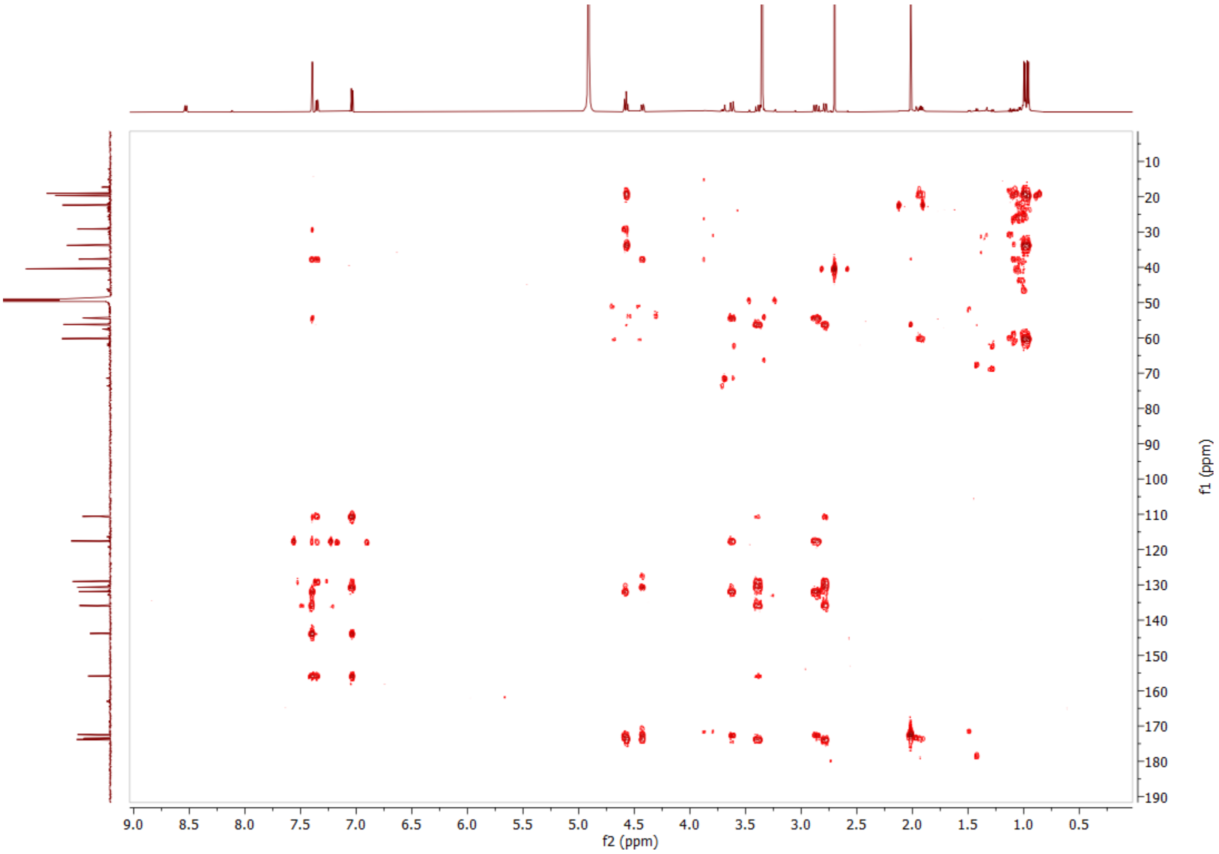

Figure S122. ^{1}H-^{13}C HMBC spectrum of **5** in MeOH-d_4 (600 MHz).

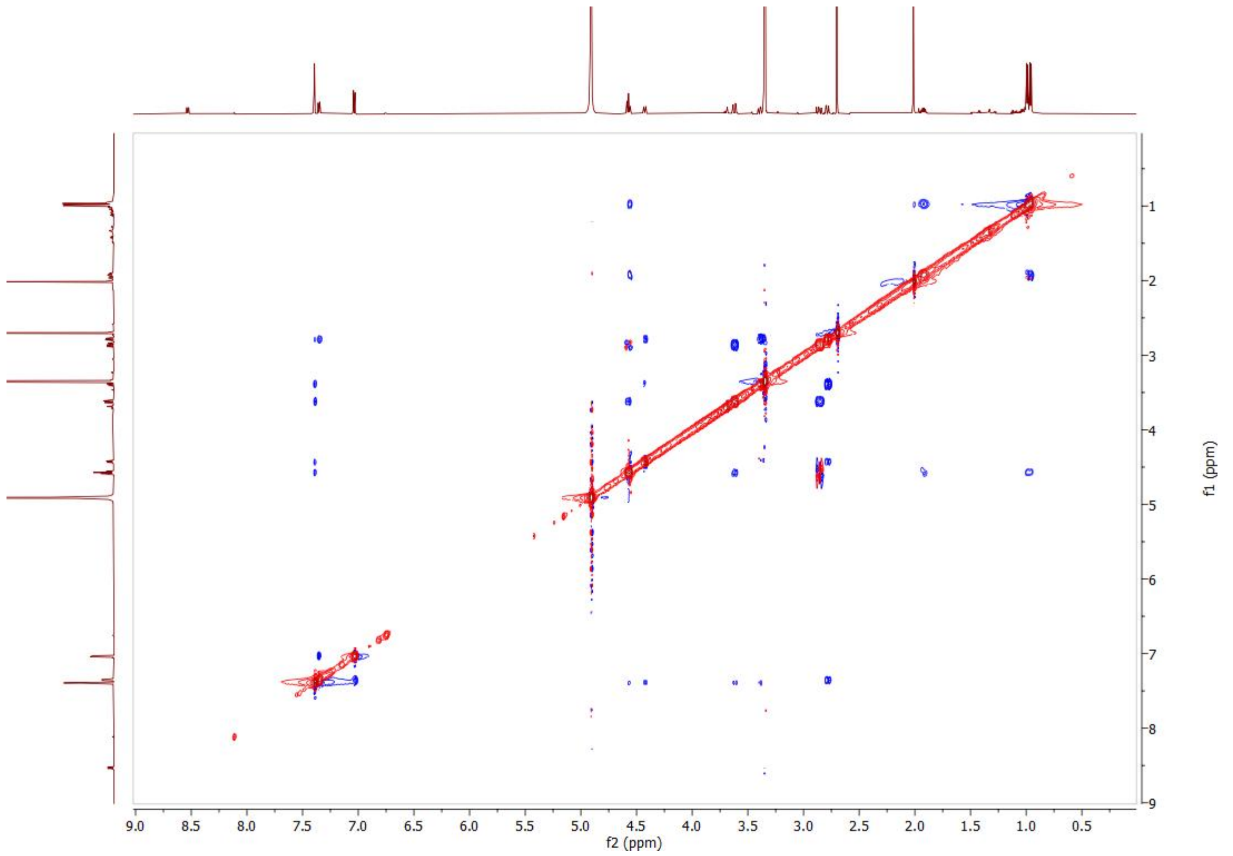

Figure S123. ^{1}H-^{1}H ROESY spectrum of **5** in MeOH-d_4 (600 MHz).

Table S3. NMR Spectroscopic Data for Compound **5** (600 MHz, MeOH-d_4).

	Position	Mult.	δ_H (J in Hz) [a]	δ_C[a]	COSY	HMBC	ROESY
Tyr	1	C		173.8			
	2	CH	4.43, dd (2.5, 11.0)	56.2	3a, b	1, 3, 4, 10	
	3	CH_2	a. 2.79, dd (2.5, 14.0)	37.7	2, 3b	1, 2, 4, 5, 9	2, 3b
			b: 3.39, dd (11.0, 14.0)		2, 3a	1, 2, 4, 5, 9	2, 3a
	4	C		130.7			
	5	CH	7.40, brs	129.0	9	3, 6, 7, 9, 5''	3b
	6	C		110.6			
	7	C		155.8			
	8	CH	7.04, d (8.5)	117.8	9	4, 6, 7, 5''	9
	9	CH	7.35, dd (2.1, 8.5)	135.9	5, 8	3, 5, 7	8
Ac	10	C		172.4			
	11	CH_3	2.01, s	22.4		10	
Val	1'	C		173.5			
	2'	CH	4.56, dd (1.7, 6.7)	60.2	3'	1, 1', 3'	3'
	3'	CH	1.93, m	33.8	2', 4', 5'	1', 2', 5'	2', 4', 5'
	4'	CH_3	0.99, d (6.7)	19.7	3', 5'	2', 3', 5'	3', 5'
	5'	CH_3	0.96, d (6.7)	19.1	3', 4'	2', 3', 4'	3', 4'
His	1''	C		172.6			
	2''	CH	4.57, td (11.0, 2.5)	54.4	3''a,b, NH	1', 1'', 3''	3''a,b
	3''	CH_2	a: 2.86, dd (11.0, 15.0)	29.2	2'', 3''b	1'', 2'', 4'', 6''	2'', 3''b
			b: 3.61, dd (2.5, 15.0)		2'', 3''a	1'', 2'', 4'', 6''	2'', 3''a
	4''	C		131.9			
	5''	C		143.8			
	6''	CH	7.39, s	117.6		3'', 4'', 5''	3''b
		NH	8.52, d (10.2)		2''		

[a] assignments are based on extensive 1D and 2D NMR measurements (HMBC, HSQC, COSY).

10. Analysis of crosslinking reactions with SavB

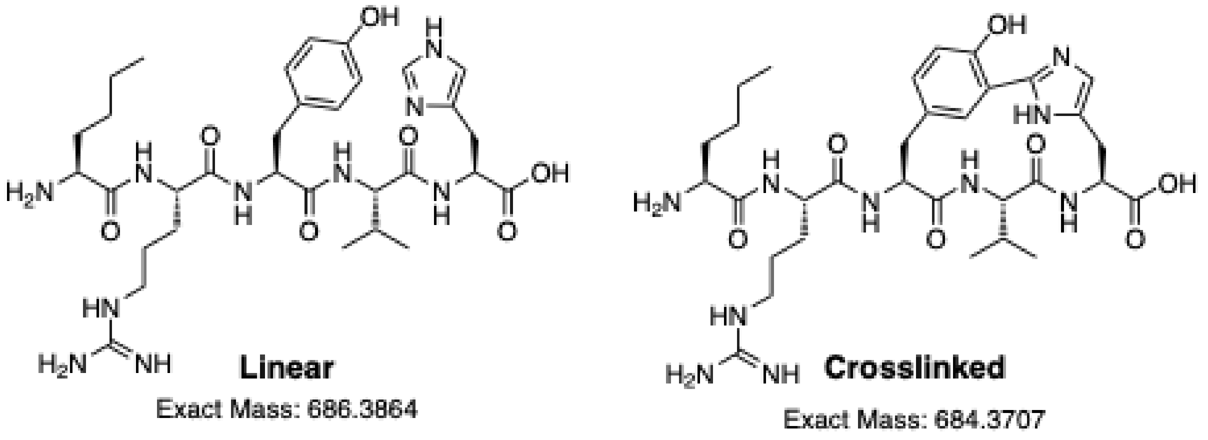

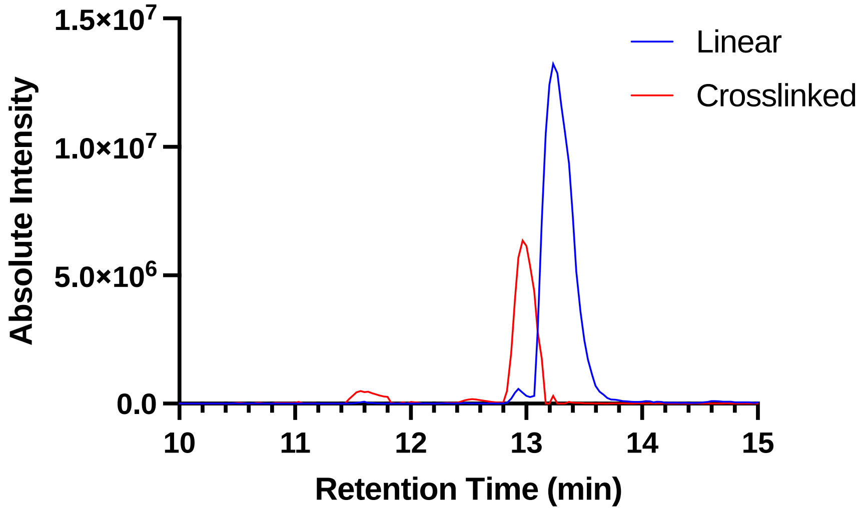

Figure S124. Crosslinking assay data for **Nle-6** with SavB. Structures of linear and crosslinked peptides shown.

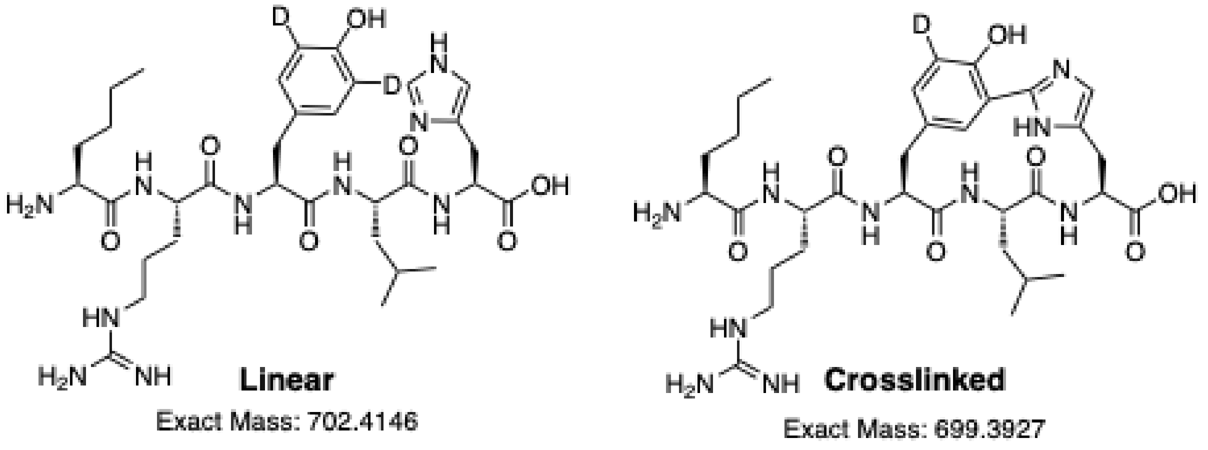

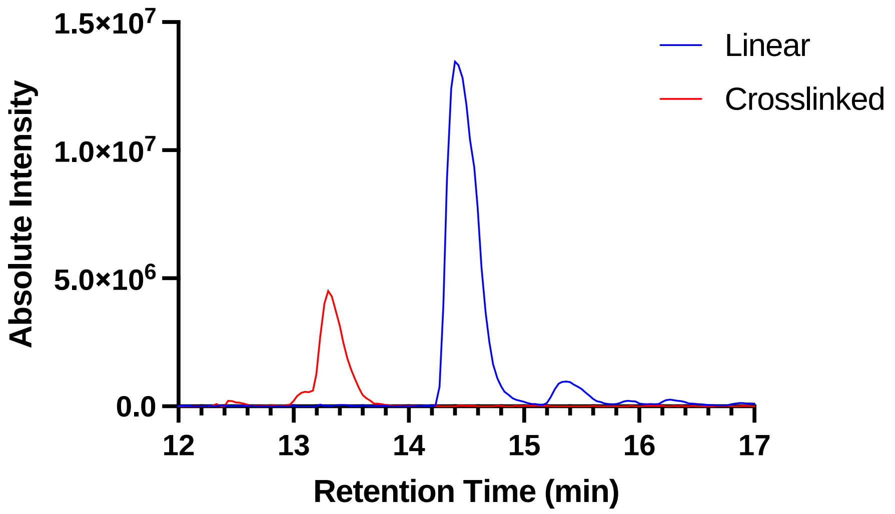

Figure S125. Crosslinking assay data for **Nle-6-(3,5-d$_2$-Tyr3, Leu4)** with SavB. Structures of linear and crosslinked peptides shown.

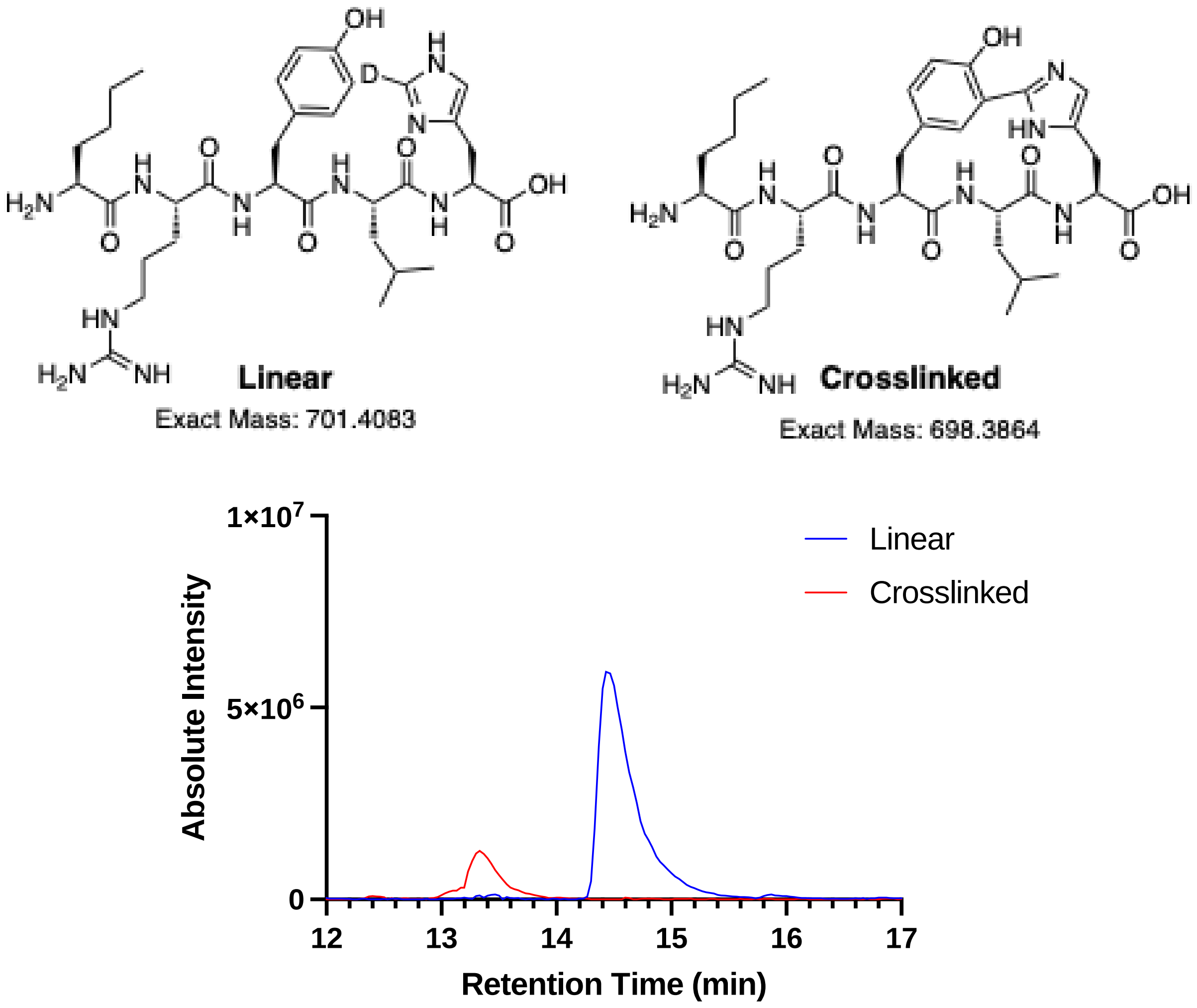

Figure S126. Crosslinking assay data for **Nle-6-(Leu4, 2-d-His5)** with SavB. Structures of linear and crosslinked peptides shown.

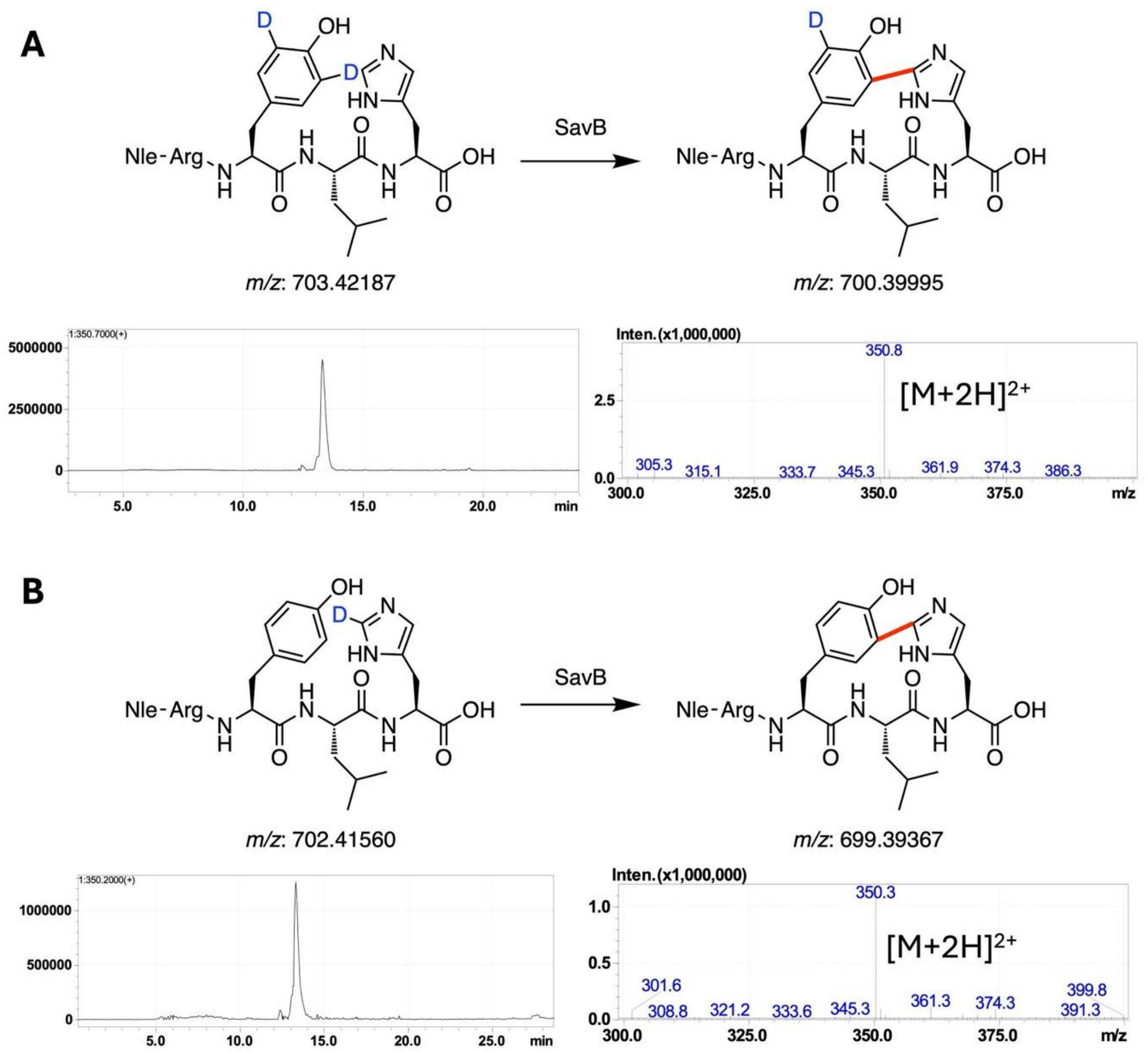

Figure S127. LCMS analysis of products from enzymatic reaction of SavB with **A) Nle-6-(Leu4, 3,5-d2-Tyr5)** and **B) Nle-6-(Leu4, 2-d-His5)**.

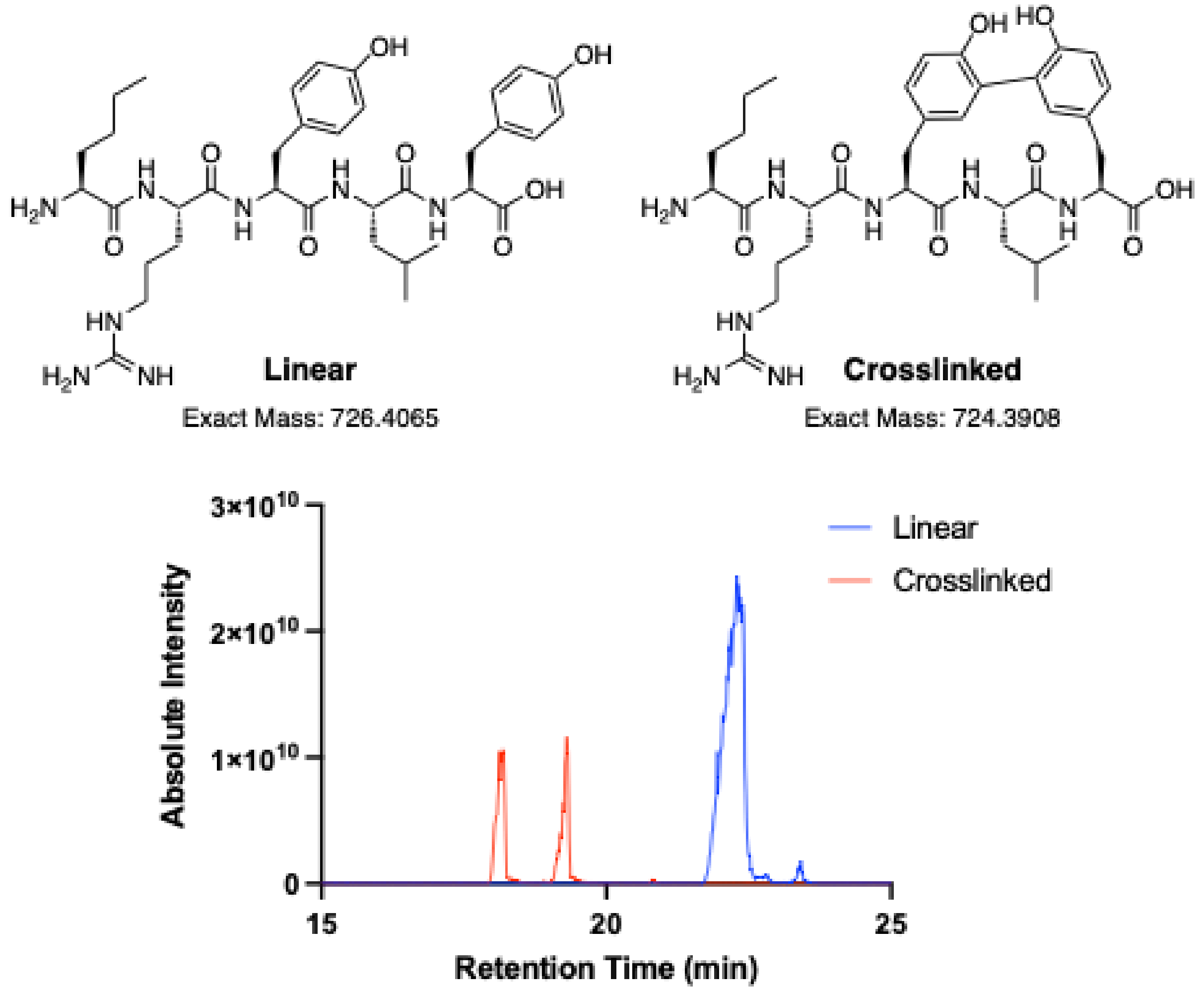

Figure S128. Crosslinking assay data for **Nle-3-(Leu4)** with SavB. Structures of linear and crosslinked peptides shown.

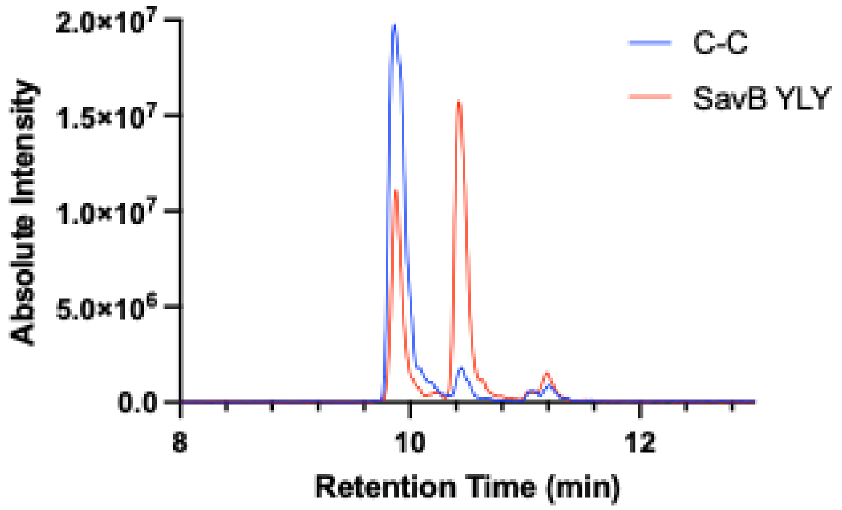

Figure S129. Comparison of products formed from turnover of **Nle-3-(Leu4)** with AciB (confirmed to be C-C crosslink) and SavB. The other product with a retention time of 10.4 min is postulated to be an O-C crosslink.

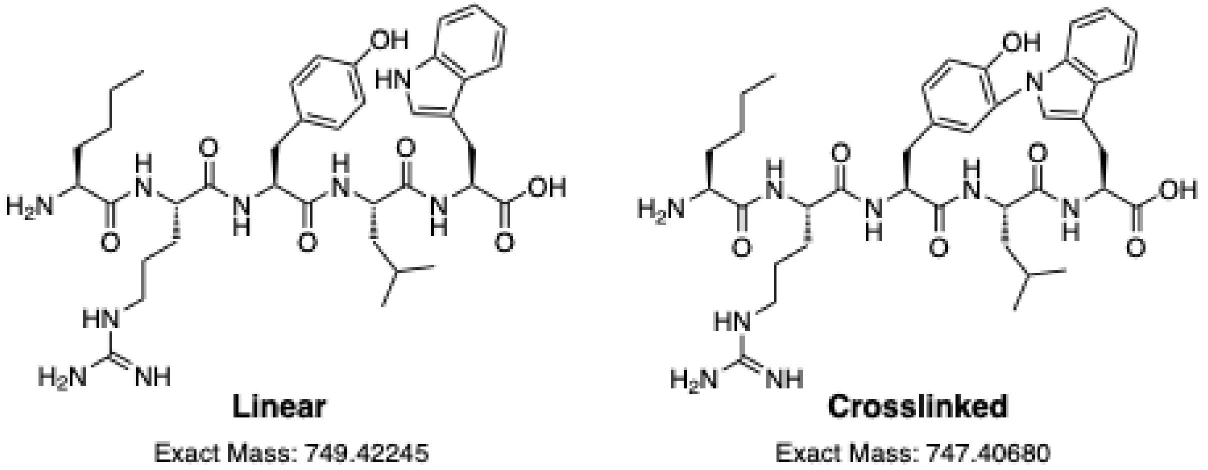

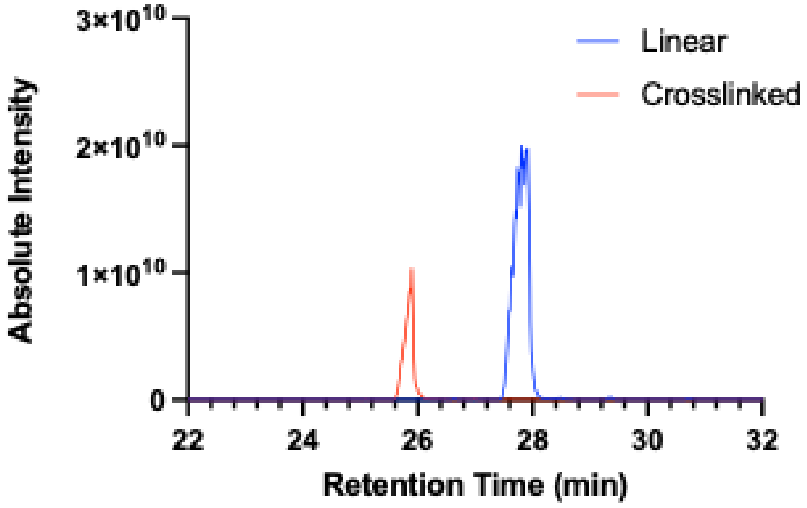

Figure S130. Crosslinking assay data for **Nle-3-(Leu4, Trp5)** with SavB. Structures of linear and crosslinked peptides shown.

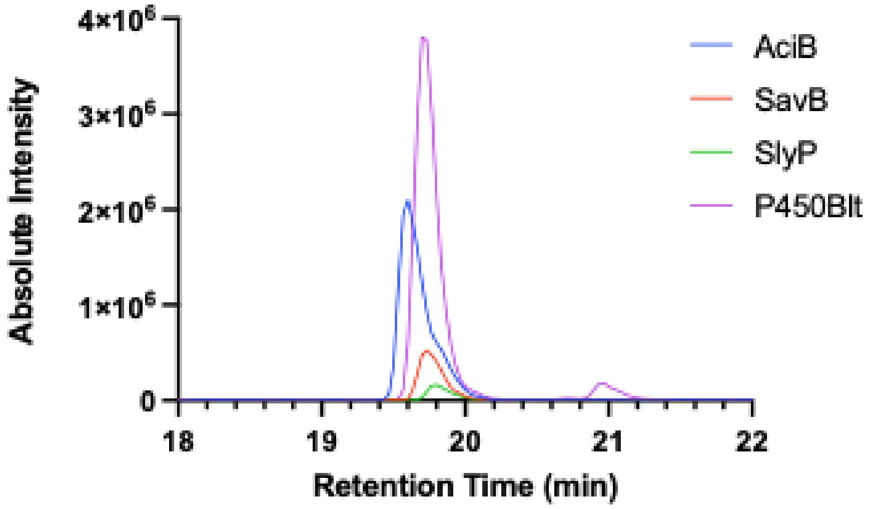

Figure S131. Comparison of products formed from turnover of **Nle-3-(Leu4, Trp5)** with $P450_{Blt}$ (confirmed to be C-N crosslink), SavB, SlyP and AciB. It cannot be determined whether there are alternative crosslinks formed due to the similar retention times of all products.

11. AlphaFold3 predictions of novel P450 motifs

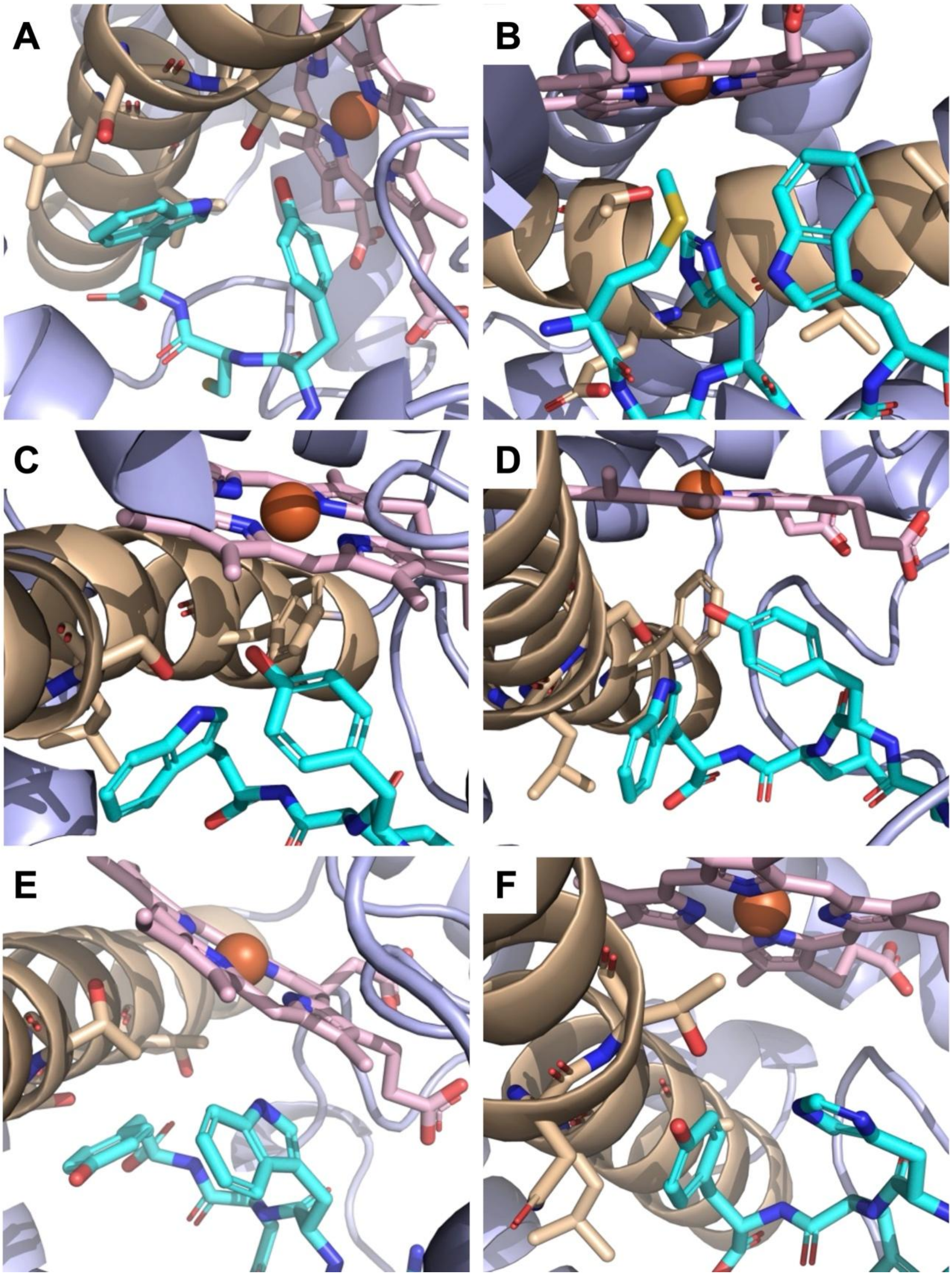

Figure S132. Predicted structures of novel biarylitide P450 motifs folded with AlphaFold3. The I helix is shown in wheat with substrate-interacting residues shown as sticks, the substrate shown as sticks in cyan, the heme shown in light pink and the P450 shown in lilac. **A)** MKYCW P450 from *S. cellulosum*, **B)** MRHRW P450 from *S. lydicus*, **C)** MKYIW P450 from *A. miaoliensis*, **D)** MKYLW P450 from *A. keratinilytica*, **E)** MQWNY P450 from *L. sphaericus*, **F)** MRHYY P450 from *M. polyrhachis*.
